# Three-Dimensional Fully π-Conjugated Macrocycles: When 3D-Aromatic and When 2D-Aromatic-in-3D?

**DOI:** 10.1021/jacs.1c13478

**Published:** 2022-05-06

**Authors:** Ouissam El Bakouri, Dariusz W. Szczepanik, Kjell Jorner, Rabia Ayub, Patrick Bultinck, Miquel Solà, Henrik Ottosson

**Affiliations:** † Department of Chemistry - Ångström Laboratory, Uppsala University, Box 523, Uppsala 751 20, Sweden; ‡ Institut de Química Computacional i Catàlisi (IQCC) and Departament de Química, Universitat de Girona, C/ Maria Aurèlia Capmany 6, Girona, Catalonia 17003, Spain; § K. Guminski Department of Theoretical Chemistry, Faculty of Chemistry, Jagiellonian University, Gronostajowa 2, Kraków 30-387, Poland; ∥ Department of Chemistry, Ghent University, Krijgslaan 281 S3, Gent 9000, Belgium

## Abstract

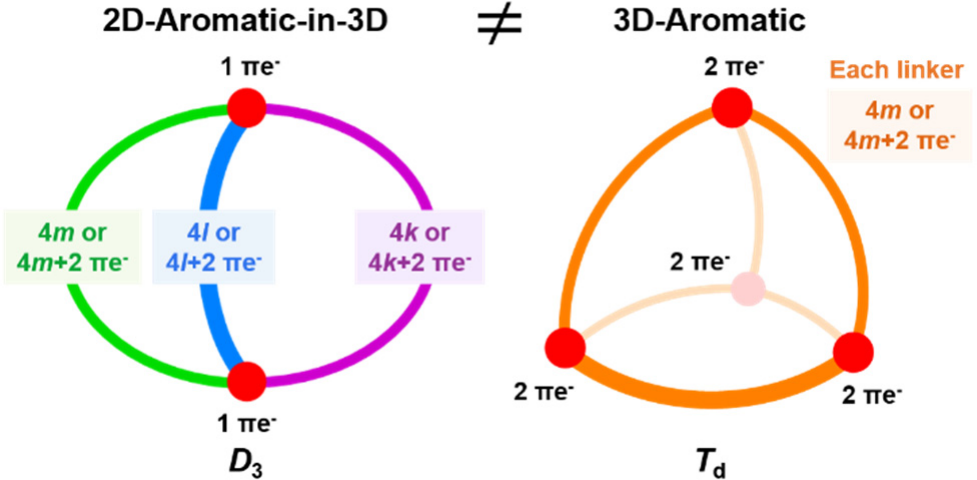

Several fully π-conjugated macrocycles with puckered or cage-type structures were recently found to exhibit aromatic character according to both experiments and computations. We examine their electronic structures and put them in relation to 3D-aromatic molecules (*e.g.*, *closo*-boranes) and to 2D-aromatic polycyclic aromatic hydrocarbons. Using qualitative theory combined with quantum chemical calculations, we find that the macrocycles explored hitherto should be described as 2D-aromatic with three-dimensional molecular structures (abbr. 2D-aromatic-in-3D) and not as truly 3D-aromatic. 3D-aromatic molecules have highly symmetric structures (or nearly so), leading to (at least) triply degenerate molecular orbitals, and for tetrahedral or octahedral molecules, an aromatic closed-shell electronic structure with 6*n* + 2 electrons. Conversely, 2D-aromatic-in-3D structures exhibit aromaticity that results from the fulfillment of Hückel’s 4*n* + 2 rule for each macrocyclic path, yet their π-electron counts are coincidentally 6*n* + 2 numbers for macrocycles with three tethers of equal lengths. It is notable that 2D-aromatic-in-3D macrocyclic cages can be aromatic with tethers of different lengths, *i.e.*, with π-electron counts different from 6*n* + 2, and they are related to naphthalene. Finally, we identify tetrahedral and cubic π-conjugated molecules that fulfill the 6*n* + 2 rule and exhibit significant electron delocalization. Yet, their properties resemble those of analogous compounds with electron counts that differ from 6*n* + 2. Thus, despite the fact that these molecules show substantial π-electron delocalization, they cannot be classified as true 3D-aromatics.

## Introduction

Numerous unconventional forms of aromaticity have been identified experimentally in the last decades: Möbius aromaticity in macrocycles and metallacycles,^1−6^ all-metal σ-aromaticity in the solid state,^7^ aromaticity in electronically excited states,^8−18^ and several other forms.^19−24^ Three-dimensional aromaticity (3D-aromaticity) is an intriguing topic introduced by Aihara in 1978 when he analyzed polyhedral boranes using a Hückel-type molecular orbital theoretical approach.^25^*Closo*-boranes, such as [B_12_H_12_]^2–^ first synthesized in the 1950s,^26^ are highly stable compounds and emblematic 3D-aromatic compounds.^27−31^ Today, 3D-aromaticity is also found in metal clusters and some charged fullerenes,^32−34^ where the aromaticity of the latter is also classified as spherical aromaticity that follows Hirsch’s 2(*n* + 1)^2^ rule.^35^ The tetrahedral P_4_ molecule (white phosphorous) and group 14 element E_4_^4–^ Zintl ions have been labeled as 3D-aromatic,^36,37^ and this also applies to the Zn^I^_8_ (Zn^I^_8_(HL)_4_(L)_8_^12–^, L = tetrazole dianion) metal cluster, which additionally can be described as cubic aromatic since it exhibits an electron delocalization over the entire Zn^I^_8_ cube.^38^

**Figure 1:**
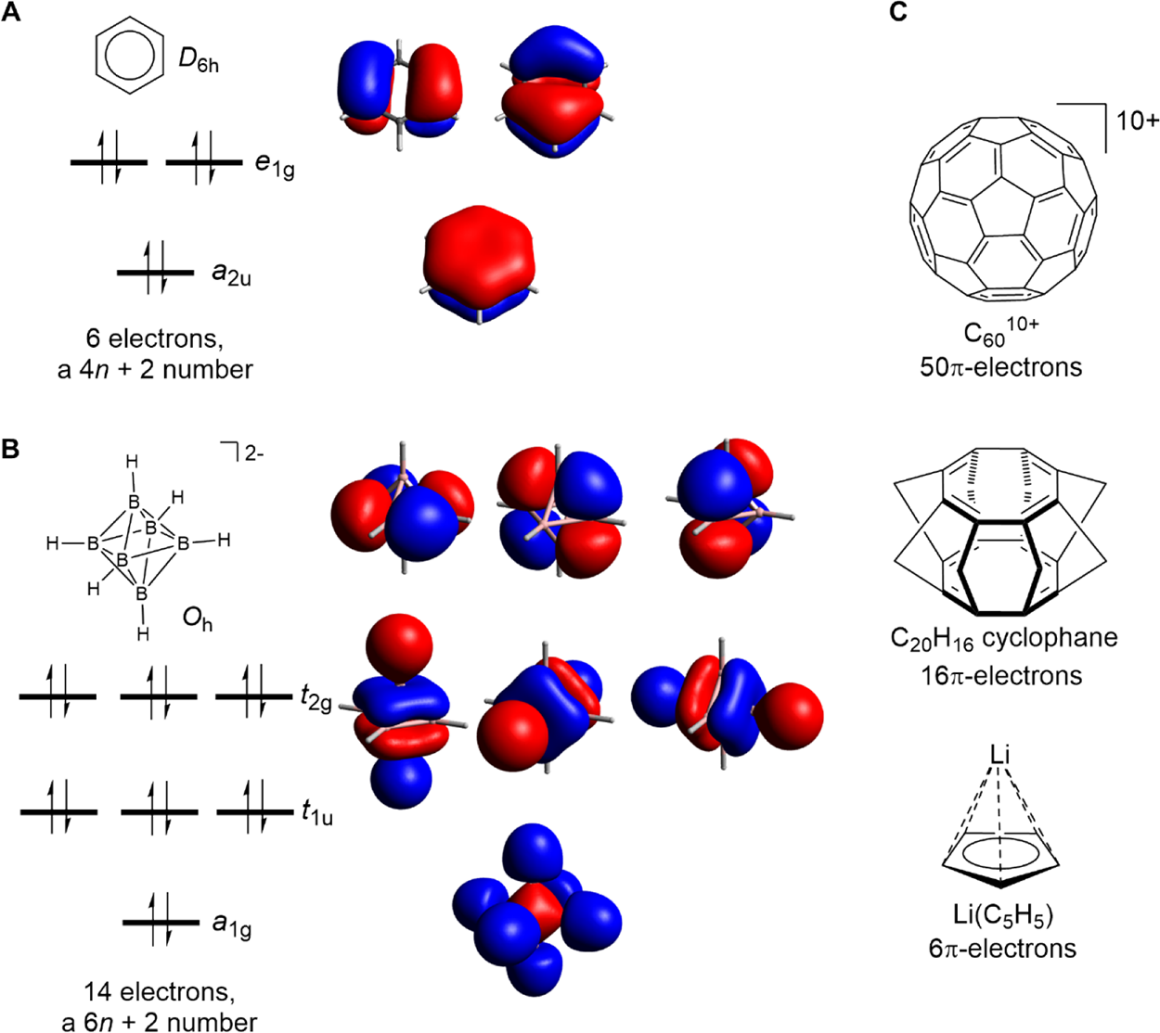
Molecular orbitals of (A) benzene as a 2D-aromatic archetype molecule and (B) the *closo*-borane B_6_H_6_^2−^ as a 3D-aromatic archetype molecule. (C) Examples of other forms of aromaticity that earlier also have been labeled as 3D-aromaticity. Of these, C_60_^10+^ is spherically aromatic and follows Hirsch’s rule, while the C_20_H_12_ cyclophane is face-to-face aromatic and Li(C_5_H_5_) is aromatic with six interstitial electrons. Two species, C_20_H_12_ and Li(C_5_H_5_), have no triply degenerate molecular orbitals. For molecular orbitals and further discussion, see Discussion A and Figures S3–S5 in the Supporting Information.

The unifying feature of these molecules is that they, besides extensive electron delocalization, have a number of degenerate molecular orbital (MO) levels that are at least triply degenerate, including the highest occupied and the lowest unoccupied MOs (HOMO and LUMO). We will henceforth call these molecules truly 3D-aromatic molecules. The MO diagram of a typical 2D-aromatic molecule such as benzene and a truly 3D-aromatic molecule like B_6_H_6_^2–^, as shown in Figure 1A,B, reveals that a closed π-electron shell results with 4*n* + 2 π-electrons in a 2D-aromatic molecule, while a closed shell requires 6*n* + 2 highly delocalized electrons in 3D-aromatic molecules with tetrahedral or octahedral structures. Monocyclic 2D-aromatic molecules with lower symmetries (*e.g.*, *C*_2*v*_ symmetric pyridine) lack the doubly degenerate π-MOs but still have π-MOs that strongly resemble those of the highly symmetric archetypes (see Figure S1 for a comparison between the π-MOs of pyridine and benzene). The same applies to 3D-aromatic carboranes in relation to the highly symmetric *closo*-boranes (see Figure S2 for CB_5_H_6_^–^ and B_6_H_6_^2–^). Indeed, Schleyer and co-workers concluded that the 4-center-2-electron 3D-aromaticity of the 1,3,5,7-bisdehydroadamantane dication manifests itself in a tetrahedral orbital topology, even though the molecule does not belong to the *T*_d_ point group.^39^

3D-Aromatic molecules are traditionally σ-conjugated. Therefore, the search for π-conjugated 3D-aromatic molecules and aromaticity that extends in three dimensions has recently intensified. Indeed, the term 3D-aromaticity has been used to label several different compound classes that exhibit electron delocalization in 3D. One form of aromaticity in three dimensions is the through-space (face-to-face or stacked-ring) aromaticity first observed through computations by Corminboeuf et al. in methano-bridged superphanes involving π-stacked [4*n*]annulenes (Figure 1C)^40^ and further explored both theoretically and experimentally in cyclophanes and hexaphyrin dimers.^41−45^ π-Capped annulenes with six interstitial electrons have also been described as 3D-aromatic.^46,47^ However, here, it should be realized that among the three aromaticity forms in Figure 1C, it is only spherical aromaticity that fulfills the criterion of triply or higher orbital degeneracy, justifying the classification of C_60_^10+^ as 3D-aromatic.

**Figure 2:**
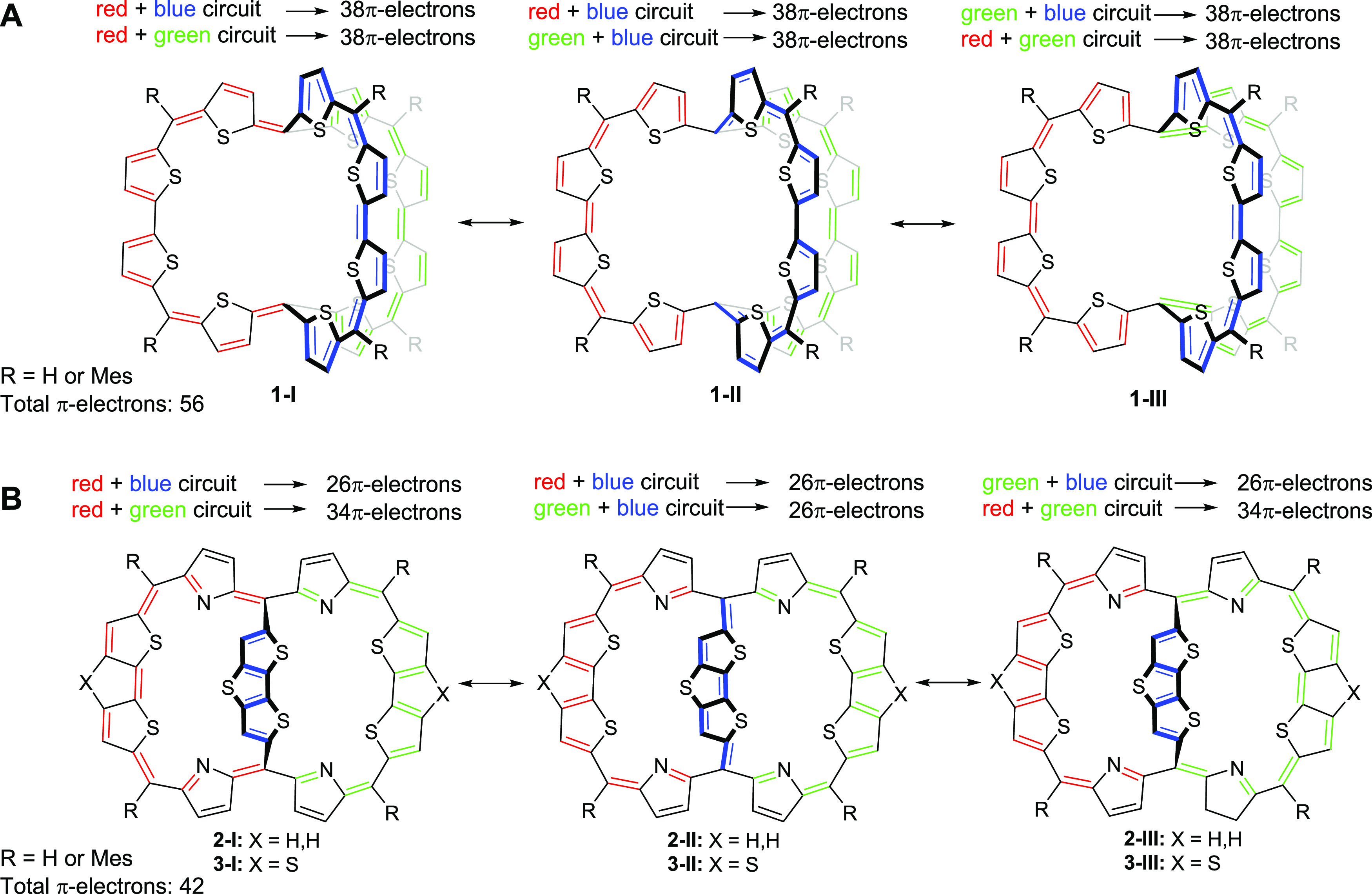
Three resonance structures of (A) π-conjugated molecular cage **1** and (B) non-planar dithienothiophene-bridged [34]octaphyrins **2** and **3** in their neutral forms. The total number of π-electrons in the three circuits of **1** is 56 (a 6*n* + 2 number), while in **2** and **3**, they are 42 (a 4*n* + 2 number) as counted by omitting the sulfurs from the main macrocycle conjugation pathway. The synthetized compounds have R = Mes substitution, yet the computations herein were on the parent compounds (R = H).

New and highly interesting compounds are the π-conjugated cage compound **1** (Figure 2A), its cations up to the hexacation **1^6+^**, and related compounds, which were reported by Wu and co-workers and considered to be “3D globally aromatic”.^48,49^ This bicyclic macrocycle consists of three equally long π-conjugated arms, and in its neutral form, it was computationally found to possess one Hückel aromatic cycle with 38π-electrons, while the lack of aromaticity in the other two cycles is a result of the *C*_2_ symmetric structure and poor π-conjugation in the third bridge. Yet, when the structure of **1** was enforced to *D*_3_ symmetry, it was reasoned that the aromaticity involves the complete molecule. The same was found for **1^6+^**, which has a *D*_3_ symmetric global minimum. Since the macrocycle **1** has 56 π-electrons, *i.e.*, a 6*n* + 2 number (*n* = 9), and exhibits three diatropic ring currents, it was concluded that **1** is 3D-aromatic when it is *D*_3_ symmetric. This also applies to the hexacation, which has 50 π-electrons, a 6*n* + 2 number with *n* = 8. Additionally, Casado and Martín considered **1** and its hexacation **1^6+^** in terms of spherical aromaticity and argued that the hexacation with 50 π-electrons follows Hirsch’s 2(*n* + 1)^2^ rule for spherical aromaticity with *n* = 4.^50^ However, this rule applies to π-electron systems that can be described as uniformly distributed spherical electron gases for which the wave functions are described by the angular momentum number *l* (*l* = 0, 1...) and where each energy level is 2*l* + 1 degenerate. Yet, despite the fact that the π-electron counts of **1** and **1^6+^** in *D*_3_ symmetry are in accord with, respectively, the 6*n* + 2 and 2(*n* + 1)^2^ rules, this symmetry provides for only double degeneracy. Furthermore, in *D*_3_ symmetry, **1** and **1^6+^** must exhibit three equivalent cyclic paths as their electronic structures must be symmetry-adapted.

Earlier, a set of compounds related to **1**, the dithiopheno-bridged octaphyrins **2** and **3** (Figure 2B), were explored by Kim and co-workers.^51^ These compounds showed two diatropic ring currents with 26 and 34 π-electrons, respectively, a feature that the authors described as dual aromaticity. Yet, the authors also used the term bicycloaromaticity, a concept introduced by Goldstein in 1967 to describe through-space aromatic (homoaromatic) interactions in charged bicyclic macrocycles with puckered structures.^52^ Results from ^1^H NMR spectroscopy showed that the ring currents of **2** are diatropic, and the aromatic character was further corroborated by Sundholm and co-workers through computations of magnetically induced current densities.^53^

Clearly, most molecules are three-dimensional, but the mere combination of a 3D molecular geometry along with (aspects of) aromaticity is not a sufficient condition for 3D-aromaticity. As a first example, helicenes are aromatic molecular scaffolds with 3D structures; however, they are not 3D-aromatic. Neither is an octahedral supramolecular scaffold with isolated aromatic compounds. Both are examples of 2D-aromatic systems embedded in 3D scaffolds, *i.e.*, a 3D-aromatic system cannot be reduced to a set of 2D-aromatic moieties. Helicenes do not fulfill the 6*n* + 2 electron count, and the supramolecular scaffold with isolated aromatic compounds (by taking a large distance between them) would also show no delocalization between the individual compounds. From Figures 1 and 2, it is apparent that a variety of compound types have been labeled as 3D-aromatic throughout time, yet is the term meaningful if used that broadly? In our view, there is a high need for a strict definition, and we build on what is known for the *closo*-boranes, labeled by Aihara as 3D-aromatic.^25^ We further relate to what is generally accepted for 2D aromaticity (Figure 1A). Hence, the four necessary conditions for true 3D-aromaticity that all must be fulfilled are (i) (at least) triply degenerate MOs or a closely related orbital topology, which exists for tetrahedral or higher symmetry molecules; (ii) a closed-shell electronic structure, which leads to a 6*n* + 2 electron count for tetrahedral or octahedral molecules (or molecules that are nearly so); (iii) extensive electron delocalization involving the complete molecule leading to resonance stabilization; and (iv) similar (electronic and magnetic) properties in the three *xyz* directions. None of these conditions by themselves is a sufficient condition. Notably, a definition requiring the fulfillment of all of these conditions is in line with Aihara’s original observations for *closo*-boranes as 3D-aromatics.^25^

Now, how do **1**–**3** and **1^6+^** comply with the essential features and the established definitions for 3D-aromaticity and bicycloaromaticity? As the *D*_3_ point group in **1** does not induce triply or higher-order orbital degeneracies, is the aromaticity in **1**–**3** and **1^6+^** instead related to the Hückel-aromaticity of two-dimensional polycyclic aromatic hydrocarbons (PAHs)? Compounds **1**–**3** and **1^6+^** are unusual and intriguing, yet even though their aromatic character is apparent from both experimental and computational observations,^44,48,53^ the cause of this aromaticity has not been analyzed in depth. In particular, there has been no search for macrocycles that potentially disprove the hypothesis that **1** and its hexacation comply with the 6*n* + 2 rule for 3D-aromaticity and Hirsch’s 2(*n* + 1)^2^ rule for spherical aromaticity. In this work, we present a deeper theoretical analysis of **1**–**3** and related compounds, along with a computational analysis to establish the precise nature of the aromaticity of these compounds.

## Results and Discussion

The analyses and discussions of our findings are split in three sections: a first with the qualitative theory on bicycloaromaticity, 3D-aromaticity, and 2D-aromaticity in three-dimensional compounds (briefly, 2D-aromaticity-in-3D); a second with computational results of these compounds discussed within the theoretical framework described in the first section; and a third where we explore species that can be truly π-conjugated 3D-aromatics.

### Qualitative Theoretical Analysis

In line with Coulson’s statement “give us insight, not numbers”,^54^ findings on (macro)molecules that potentially exhibit a new or unusual form of aromaticity must foremost be placed in a qualitative theoretical framework instead of a framework primarily based on computational observations. Such a theoretical framework is given next.

#### On the Alleged Bicycloaromaticity of **2** and **3**

**Figure 3:**
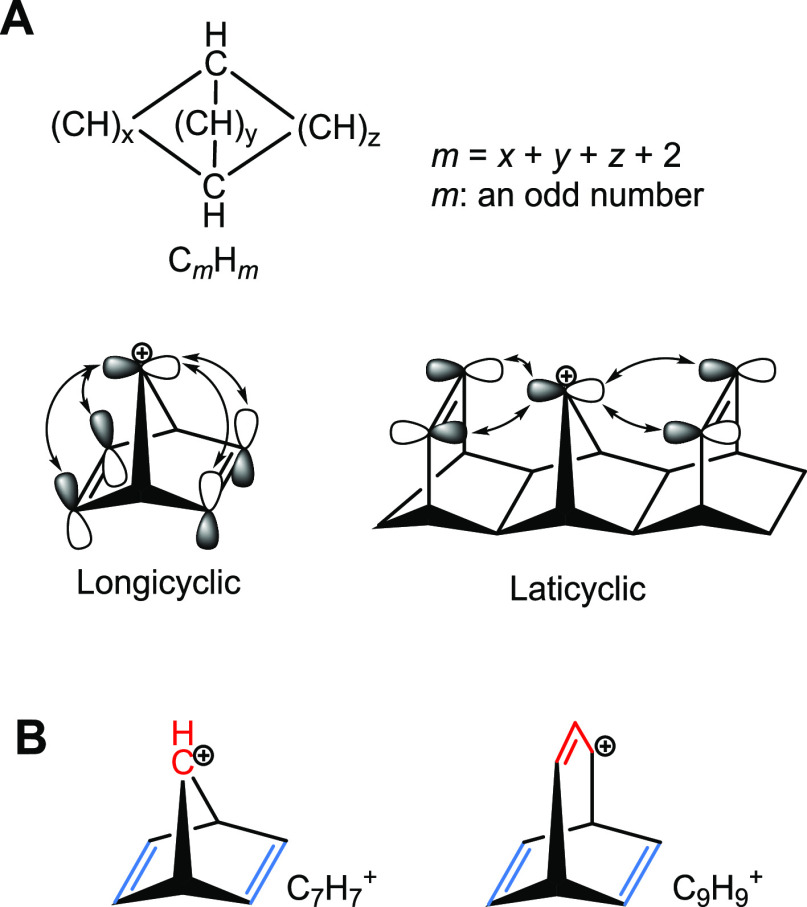
(A) General structure of a hydrocarbon that is bicycloaromatic and orbital interactions leading to bicycloaromatic stabilization in longicyclic and laticyclic topologies. (B) One example of a bicycloaromatic (C_7_H_7_^+^) and a bicycloantiaromatic (C_9_H_9_^+^) species.

We start with the two dithiopheno-bridged octaphyrins **2** and **3** as they link PAHs with the cage-type macrocycles. Macrocycles **2** and **3** were recently labeled as bicycloaromatic,^48^ a form of aromaticity defined by Goldstein as a case where three separate π-conjugated polyene segments (ribbons) in a bicyclic C_*m*_H_*m*_ hydrocarbon interact through-space in either a longicyclic or a laticyclic topology (Figure 3A).^55^ Two criteria must be fulfilled for a bicycloaromatic interaction: (*i*) *m* must be an odd number (*i.e.*, odd number of C and H atoms), and (*ii*) the total number of π-electrons must equal 4*n*. With two isolating (sp^3^ hybridized) bridgehead C atoms, there will be *m* – 2 sp^2^-hybridized C atoms in the polyene segments, and together with the two criteria, this implies that the bicycloaromaticity concept applies to cations and anions but not to neutral all-carbon species. A potentially bicycloaromatic species is the bicyclo[2.2.1]heptadienyl cation (C_7_H_7_^+^) as it has four π-electrons and *m* = 7 (Figure 3B).^52^ Conversely, bicyclo[3.2.2]nonatrienyl (C_9_H_9_^+^) with *m* = 9 should in theory be bicycloantiaromatic as it has in total six π-electrons and the even–odd bridge interaction is destabilizing as it involves four π-electrons.

Based on the original definition of bicycloaromaticity, it is clear that **2** and **3** do not satisfy the criteria for bicycloaromaticity as (i) they possess 42 π-electrons (a 4*n* + 2 number corresponding to bicycloantiaromaticity), (ii) the three bridges (ribbons) are not separate from each other as they all interact conjugatively with the two bridgehead atoms that are sp^2^ instead of sp^3^ hybridized, and (iii) all bridges have even numbers of atoms in the π-conjugated paths (16, 16, and 8) (Figure 2B). With two bridgehead atoms, this implies that they are not C_*m*_H_*m*_ compounds with *m* odd as the sum equals 42 (16 + 16 + 8 + 2). Thus, the description of bicycloaromaticity by Kim and co-workers as a concept where “two (or more) potentially aromatic circuits are contained within the same non-planar molecular framework and share the same π-electrons” is not in line with the original definition (Figure 3).

#### On the Alleged 3D-Aromaticity of **1** and **1^6+^**

**Figure 4:**
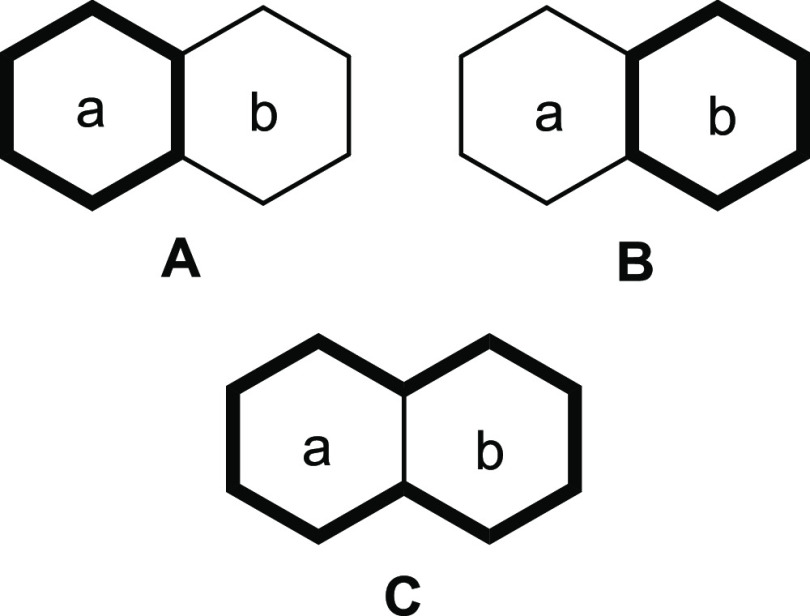
The three circuits of naphthalene. A and B correspond to benzenoid circuits, and C corresponds to the naphthalenic circuit.

Next, we now turn to the claimed 3D-aromaticity of compounds **1** and **1^6+^**. To determine if a compound is 3D-aromatic, one must analyze its electronic structure. As noted in the Introduction, a 3D-aromatic molecule should have (at least) triply degenerate orbitals, but this degeneracy will be lifted when heteroatoms are incorporated in the molecular scaffold (see CB_5_H_6_^–^, Figure S2) or when effects such as bond length alterations lower the symmetry. Compounds **1** and **1^6+^** are aromatic in their *D*_3_-symmetric structures, and they are three-dimensional and have 6*n* + 2 π-electron counts and extensive electron delocalization. We argue that **1**–**3** as well as **1^6+^** are expanded and puckered versions of PAHs, instead of true 3D-aromatics, where the π-electrons are shared between a set of circuits that each fulfills the 4*n* + 2 rule (Figure 4) (for **2** and **3** with different *n*).^56−61^ The total ring-current picture of a PAH is constructed from all the different circuits that can be drawn. Naphthalene has three circuits, where the two hexagons (*a* and *b*) correspond to local six-electron circuits (A and B), while both hexagons are involved in the 10-electron circuit C (Figure 4).^62^ Furthermore, the induced diatropic ring currents of circuits A and B, generated in an external magnetic field, are equivalent and cancel each other in the central C–C bond so that naphthalene exhibits an induced diatropic ring current exclusively along the perimeter. A similar analysis can be made for anthracene and other PAHs.^58,63^ Now, to what extent is the aromaticity of macrocycles **2** and **3** reminiscent of that of naphthalene? Also, can naphthalene be altered/modified to the extent that its ring currents resemble those of the two bicyclic macrocycles **2** and **3**, and should one not consider three aromatic cyclic paths in **2** and **3**?

**Figure 5:**
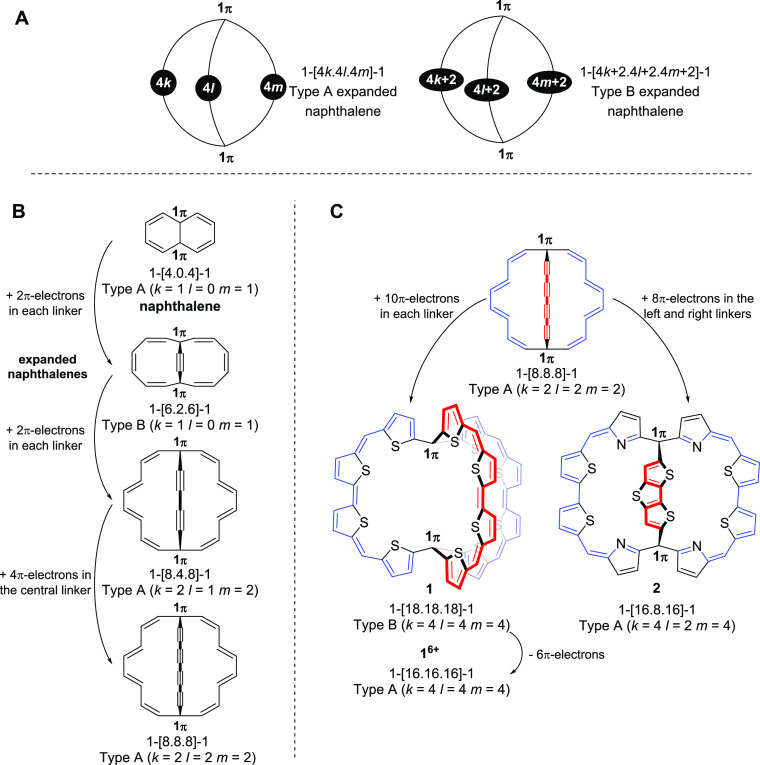
(A) Generalized descriptions of the three-linker bicyclic aromatic hydrocarbons labeled as Type A and Type B expanded naphthalenes. (B) Expansion of naphthalene to gradually larger three-dimensional bicyclic structures. (C) Application of the electron count approach for description of macrocycles **1**, **1^6+^**, and **2**. The numbers of π-electrons are counted by omitting the sulfurs from the main macrocycle conjugation pathway.

Figure 5A shows the two general bicyclic structures to which the three compounds of Wu et al. and Kim and co-workers belong; the difference between the two structures being the total number of π-electrons in the three arms, *i.e.,* 4*n* (Type A) and 4*n* + 2 (Type B). Here, it should be pointed out that the single π-electrons at the two bridgehead C atoms displayed in the generalized structures do not represent radical centers but instead indicate that these π-electrons are involved in π-bonds to either of the three linkers. Indeed, the three-dimensional bicyclic structures can all be viewed as expanded naphthalenes (Figure 5B). Starting at naphthalene, we increase the π-electron count by expanding the six-membered rings through linkers while keeping the topology of Figure 5A to ensure that each circuit allows for 2D Hückel-aromaticity. The 1-[4.0.4]-1 label of naphthalene, used herein, reveals that there is one electron at each of the two bridgehead C atoms, four π-electrons in, respectively, the left and right linkers and none in the central, *i.e.*, naphthalene is a bicyclic Type A compound. Expanding the 1-[8.4.8]-1 Type A compound with a 1,3-butadiyne unit in the central linker gives a 1-[8.8.8]-1 Type A bicycle, from which then compounds **1**–**3** can be designed (Figure 5C). When molecule **1** is forced to *D*_3_ symmetry, it exhibits three diatropic ring currents with 38 π-electrons each, *i.e.*, it is a Type B expanded naphthalene. The hexacation **1^6+^**, a Type A expanded naphthalene, displays similar properties to **1** but with three 34π-electron rings. Both 34 and 38 are 4*n* + 2 numbers so that **1** and **1^6+^** with *D*_3_ symmetry exhibit three Hückel-aromatic circuits similar to naphthalene, although naphthalene is planar and has one 10π- and two 6π-electron circuits.

How do the π-electron counts vary in these bicyclic compounds? Especially, when do they equal 6*n* + 2? For Type A expanded naphthalenes, the π-electron counts generally equal 4*k* + 4*l* + 4*m* + 2, i, which simplifies to 3 × 4*k* + 2 = 6 × 2*k* + 2 when *k* = *l* = *m*. With *n* = 2*k* we get the π-electron count 6*n* + 2. Thus, with three linkers of equal length, the π-electron counts of expanded naphthalenes happen to coincide with the π-electron counts of truly 3D-aromatic molecules. This also applies for the Type B expanded naphthalenes with six extra electrons, still resulting in a 6*n*′ + 2 count (*n*′ = *n* + 1). Among **1**–**3** and **1^6+^**, it is therefore only **1** and **1^6+^** that have 6*n* + 2 total π-electron counts. Yet, will similar bicyclic cages to **1** and **1^6+^** exhibit aromatic character also when *n* ≠ *m* and/or *k*? If the tether lengths are just slightly different, *e.g.*, *k* = *l* + 1 = *m* + 1, then the π-electron count is not a 6*n* + 2 number, although the structure should still allow for strong π-conjugation and Hückel-aromaticity in the three individual macrocyclic paths. Now, if these latter species are calculated to be aromatic, then that will disprove that **1** and **1^6+^** are 3D-aromatics. These two species would instead be three times locally 2D Hückel-aromatic to equal extents, a feature that is unrelated to 3D aromaticity. Moreover, the globally aromatic character simply stems from the symmetry-adapted electronic structure in *D*_3_ symmetry.

#### Design of True π-Conjugated 3D-Aromatics

**Figure 6:**
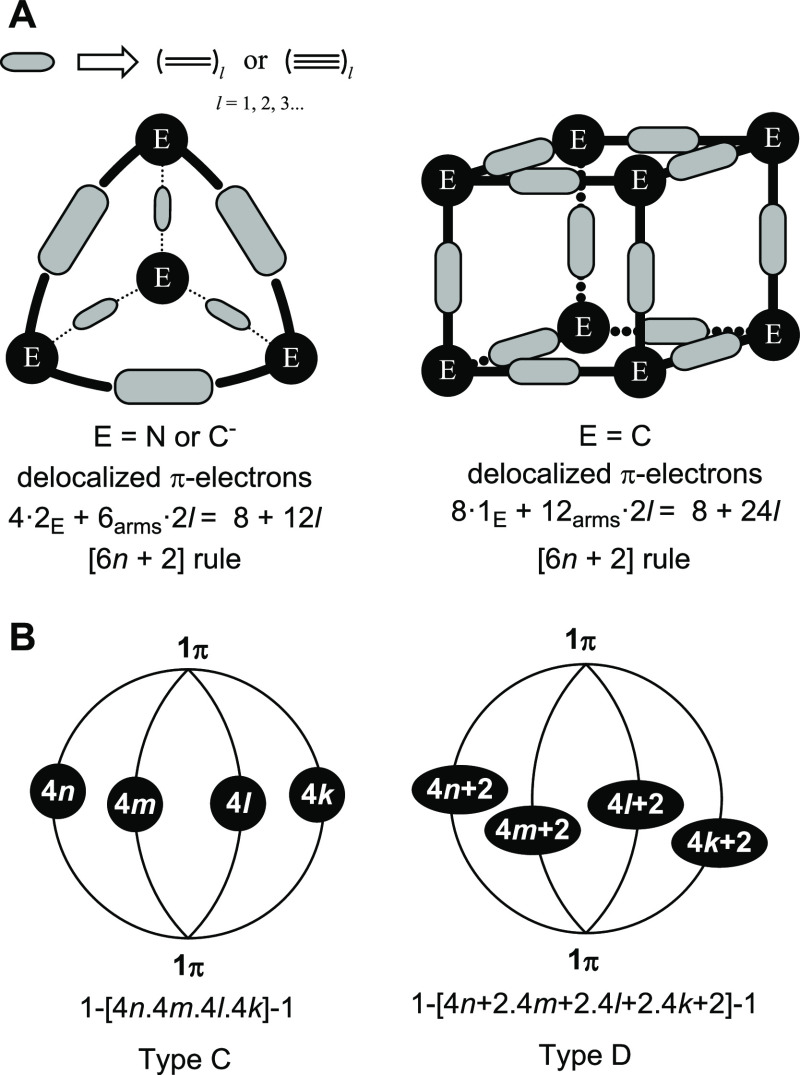
(A) Generalized design of potentially π-conjugated molecules, which can be truly 3D-aromatic. (B) Generalized structures of expanded four-linker (Type C and Type D) polycyclic aromatic hydrocarbons with the number of π-electrons in the arms.

Having refuted the claims of the bicycloaromaticity of **2** and **3** as well as the 3D-aromaticity of **1** and **1^6+^**, how to design π-conjugated (macrocyclic) cage molecules that are truly 3D-aromatic? As it is the higher-order point groups that exhibit irreducible representations with triple (or higher) degeneracies, leading to species that possibly can be 3D-aromatic, a π-conjugated macrocycle that is 3D-aromatic must have (approximate) tetrahedral, octahedral or icosahedral, or even higher symmetry. Importantly, the local π-orbitals of the π-conjugated linkers must be oriented radially outward if they are to interact with the local p_π_ orbitals at the vertex atoms. We have also analyzed such tetrahedral and cubic species through computations (Figure 6A and *vide infra*). These species must have radial orientations of their π-orbitals similar to charged fullerenes C_60_^10+^ and C_20_^2+^, which have been explored computationally and found to follow Hirsch’s 2(*n* + 1)^2^ rule as they are spherically aromatic.^35,64^ Yet, Hirsch’s rule has a limitation as it seems applicable only to species with 50 π-electrons or less.^65^ A further caveat with regard to the cubic species is that each of the faces has 4*m* π-electrons (*m* ≠ *n*), meaning that these can be Hückel-antiaromatic. Hence, the cubes can in theory be both globally 3D-aromatic and six-fold locally 2D-antiaromatic.

#### 3D-Baird-Aromaticity

Another difference between true 3D-aromaticity and 2D-aromaticity-in-3D occurs for open-shell species that can adopt a Baird-aromatic character. Compounds that are Baird-aromatic follow Baird’s rule, which tells that the lowest ππ* triplet state of [4*n*]annulenes is aromatic, and it applies to electronically excited states and to open-shell ground states.^66,67,13−16^ For a (macro)cyclic two-dimensional molecule, which is Hückel-aromatic, one can achieve a Baird-aromatic triplet state by either removal or addition of two electrons,^13^ exemplified by, respectively, the benzene dication and dianion for which there are derivatives shown experimentally to have either triplet ground states or low-lying triplet states.^68−70^ Similar to the 2D-Baird-aromatic benzene dication and dianion, true 3D-Baird-aromaticity will occur for the trication and trianion as their π-electron occupancies allow for half-filled triply degenerate orbitals with an electron count of 6*n* – 1. Hence, true open-shell 3D-Baird-aromaticity will not occur for the triplet dication even though **1^2+^**, which has a triplet ground state, has an NICS(0) value of −2.6 ppm,^48^ which (at best) suggests a modest Baird-aromatic character. Instead, the triplet dication of a true 3D-aromatic species would have an electron configuration with four π-electrons in the triply degenerate HOMOs, an electron configuration that due to the Jahn–Teller effect should lead to a distortion away from the high symmetry. Now, what is the electron count for Baird-aromaticity in a bicyclic macrocycle that is 2D-aromatic-in-3D? If one goes by simple π-electron counts, then the quartet trication of both Type A and Type B expanded naphthalenes with *k* = *l* = *m* can be Baird-aromatic as each linker will have 4*k* – 1 π-electrons for Type A and 4*k* + 1 π-electrons for Type B providing for three Baird-aromatic 4*n* circuits (*n* = 2*k*). Additionally, the triplet dication can be Baird-aromatic, as found by Wu and co-workers.^48^ For 3D-aromatic species, in contrast, only the trication in its quartet state can exhibit Baird-aromaticity.

#### Tetra-tethered 2D-Aromatics-in-3D

A final feature of 2D-aromatic-in-3D structures is that they can be expanded to (hypothetical) macrocyclic compounds with additional arms (Types C and D, Figure 6B). Here, it is noteworthy that a macrocyclic cage molecule with four tethers, yet with π-conjugated (aromatic) Ni-porphyrin units at its two poles, was recently reported by Wu and co-workers.^71^ It was argued that the dication of this species “discloses the close correlation between 3D global aromaticity and 2D Hückel-aromaticity”. Yet, does it? When the molecules of Figure 5B have four linkers with equal numbers of π-electrons, one has gedanken molecules that are 2D-aromatic-in-3D with six Hückel-aromatic 4*n* + 2 cycles and with total π-electron counts of 8*n* + 2, a π-electron count that is not in line with true 3D-aromaticity. As pointed out in the Introduction, 3D-aromaticity cannot be reduced to a set of 2D aromatic moieties. This also becomes obvious *via* the origins of, respectively, the 4*n* + 2 and 6*n* + 2 electron counts (Figure 1A,B) as the electron counts stem from the different orbital degeneracies in the two aromaticity types.

### Computational Analysis

Here, we use quantum chemical calculations to probe the conceptual theories described above. We start by examining the electronic structure and aromaticity of naphthalene (**4**), puckered naphthalene (**5**), and the benzocyclooctatetraene dication (**6**) as 10π-electron bicyclic molecules, and we connect these to the non-planar dithiopheno-bridged octaphyrins **2** and **3**, which have one short and two long bridges. Subsequently, we study bicyclic macrocycles with three bridges of (approximately) equal lengths whereby these compounds adopt cage-type structures. We especially analyze compounds that allow us to contest the presumption that **1** and **1^6+^** are 3D-aromatics that follow the 6*n* + 2 and 2(*n* + 1)^2^ rules. At the end, we explore tetrahedral and cubic π-conjugated compounds (Figure 6A) with potentials to be 3D-aromatics.

It should be stressed that the study is aimed at establishing the type of aromaticity, not the quantitative extent of aromaticity. We utilize primarily the B3LYP functional^72−74^ with the 6-311G(d,p) basis set. This functional is known to exaggerate aromaticity in macrocycles when compared to long-range corrected functionals (*e.g.*, CAM-B3LYP),^75^ which are recommended for such molecules^76^ and for aromatic compounds in general.^77^ Calculations with CAM-B3LYP are, however, performed on selected species (see Tables S4 and S5 in the SI). Using B3LYP entails that we may overestimate the extent of aromaticity. This is appropriate here because if even this functional fails to reveal a high degree of aromaticity, then this conclusion holds even stronger for the other functionals.

Aromaticity analyses are performed through the electron density of delocalized bond (EDDB) function,^78,79^ nucleus independent chemical shifts (NICS),^80−83^ anisotropy of induced current density (ACID),^84^ and current densities and strengths of the integrated current densities calculated perturbatively with the GIMIC program.^85,86^ The EDDB function discloses electron delocalization and, hence, electron density that cannot be attributed exclusively to a particular chemical bond.^78,79^ The EDDB reveals that in archetypical Hückel’s 4*n* + 2 aromatics, the cyclic (Kekuléan) delocalization predominates and is very effective (82–89% for 6π-systems), while in the case of Hückel’s 4*n* antiaromatics, cyclic delocalization almost entirely vanishes, although some local resonance effects still remain in their π-systems (see Discussion B in the Supporting Information and Figure S6 for further examples). This clarifies the wide span in the electron delocalization between aromatic and antiaromatic annulenes. As we are aware that electron-sharing and magnetic response properties are not always connected,^87−90^ we follow the common practice of using a set of descriptors to characterize the aromatic character of a given species. It should, however, be noted that we emphasize results on electron delocalization as provided by the EDDB.

#### Non-planar Dithiopheno-Bridged Octaphyrins as Expanded Naphthalenes

**Figure 7:**
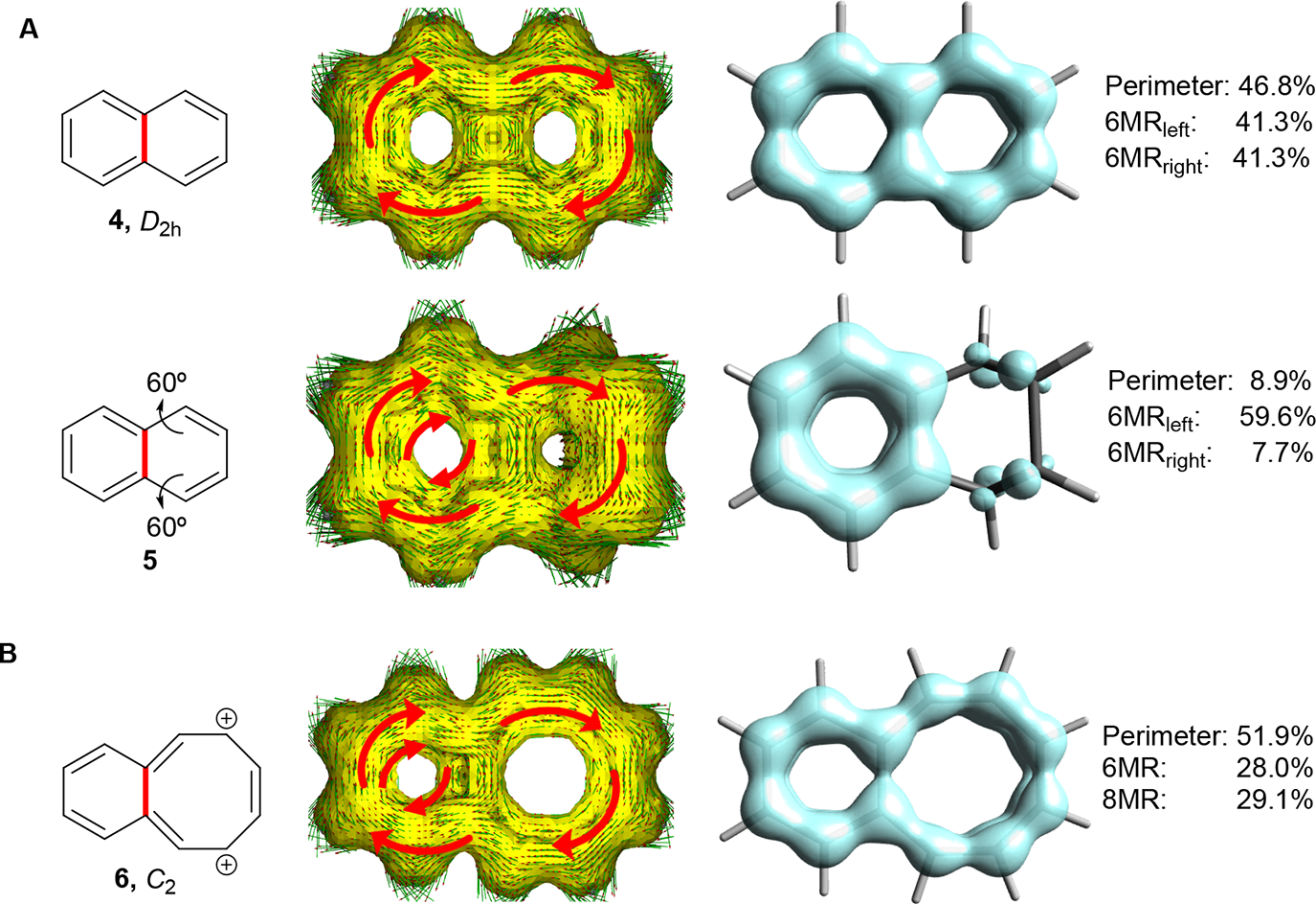
(A) Naphthalene in its planar (**4**) and puckered (**5**) structures and the corresponding ACID and EDDB plots. (B) Molecular structure and symmetry of benzoCOT dication (**6**). *n*MR = *n*-membered ring. For full-scale images of the ACID plots for **4**–**6** as well as naphthalene at other distortion angles, see Figures S7 and S8.

The fact that naphthalene exhibits two circuits with six π-electrons and one with 10 becomes apparent through electronic aromaticity indices. The EDDB reveals delocalization through both the perimeter and the central CC bond (**4**, Figure 7A), and as the electronic structure is a superposition of the π-dectet and the two π-sextet resonance forms, the EDDB-based percentage effectiveness of cyclic delocalization of π-electrons in each cycle is similar.^61^ In contrast, and as noted above, magnetic indices give an obscured picture since the induced ring currents from the two hexagons circuits of **4** cancel each other perfectly in the central CC bond (Figure 7A).^56^ This is also clear from the current densities obtained with GIMIC as the average current strength in the perimeter bonds is ∼13 nAT^–1^ (diatropic), while the current strength in the central CC bond is nil (for a definition of the strengths of the integrated current densities, see ref (86)). Yet, when gradually distorting one of the hexagons leading to a puckered *C_s_* symmetric structure (**5**, Figure 7A), the ring current in the puckered hexagon is attenuated whereby the two 6π-electron ring currents do not cancel anymore and a current density in the inter-ring CC bond emerges (for current densities of gradually more distorted naphthalenes, see Tables S1 and S2). One can also see that the π-electron delocalization is very attenuated in the puckered hexagon while it is enhanced in the planar hexagon (Figure 7A).

Yet, it is also possible to achieve differentiation in the 6π-electron currents, and a current density in the inter-ring CC bond, by going to the nearly planar benzocyclooctatetraene dication (**6**, Figure 7B). For **6**, an NICS-*XY* scan^83^ shows that the local 6π-electron aromaticity in the hexagon dominates over that in the octagon (Figure S7), whereby the resulting current density in the central CC bond is 4.7 nAT^–1^. Clearly, a structural differentiation between the two 6π-electron cycles, achieved either by distortion or altered ring size, provides for a differentiation in the ring currents of the two cycles, leading to an imperfect cancellation of the currents in the inter-ring CC bond. In **2** and **3**, the dissimilarities between the two 26π-electron cycles come about because of the orientation of the dithienothiophene (DTT) bridge, which leads to a better π-conjugation with one half of the octaphyrin than with the other half, reflected in C–C–C–C dihedral angles to the DTT bridge, which are 7 and 33°, respectively. This provides a better electron delocalization in one 26π-electron cycle than in the other (41.7 *vs* 35.6%), although the best delocalization is along the perimeter (42.5%).

Now, can one instead turn the DTT-bridged octaphyrins into compounds with similar features as planar naphthalene, that is, can they be turned into symmetric (and near-planar) molecules with no current densities on the central bridge? The [34]octaphyrin, *i.e.*, **2** without the DTT bridge, adopts a modestly helical structure 0.2 kcal/mol lower in energy than a planar *C*_2*v*_ symmetric structure (a first-order saddle point). However, the DTT bridge is too long by ∼0.9 Å to fit into a [34]octaphyrin (see Figure S9A), and its incorporation leads to the strongly puckered compound. Indeed, a planar *C*_2*v*_ symmetric structure of **2** is a higher-order saddle point 87.0 kcal/mol above the minimum. The importance of the non-planarity for the observations made by Kim and co-workers becomes obvious through an ACID plot of the planar *C*_2*v*_ structure because now the two 26π-electron ring currents are of similar weights and cancel on the bridge, whereas the current through the perimeter remains (Figure S10). In the planar structure of **2**, there is a slight reduction in the difference in the extent of delocalization between the two 26π-electron cycles (34 *vs* 39%) (Table S3). Hence, it is the non-equivalence of the two 26π-electron resonance structures of **2** and **3**, effectuated through the non-planar structures and the larger C–C–C–C dihedrals within one macrocyclic path than within the other, that leads to the observation of two strong ring currents with, respectively, 34 and 26 π-electrons and one with weakened strength (Figure S9B). It is apparent that these macrocycles resemble distorted naphthalene.

**Figure 8:**
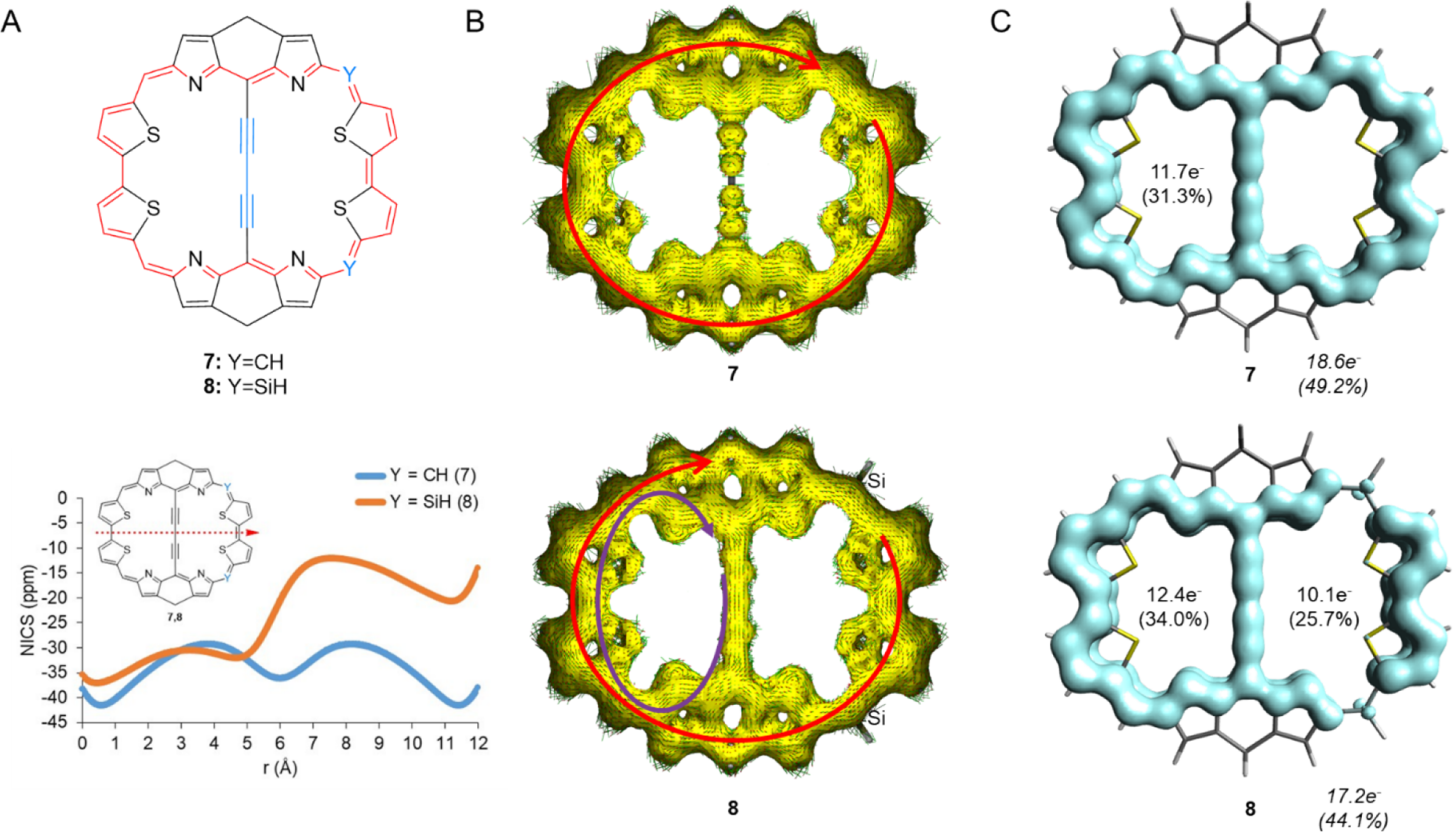
(A) Expanded naphthalenes modeled from **2** and NICS-*XY* scans for the expanded naphthalenes **7** (blue) and **8** (dark orange), (B) ACID plots with the induced current densities of **7** and **8**, and (C) EDDB plots of **7** and **8**. For full-scale images of the ACID plots, see Figures S11 and S12. For additional EDDB details regarding **8**, see Discussion C, Supporting Information.

To achieve a planar octaphyrin-based macrobicyclic species, the DTT bridge was replaced by a shorter butadiyne bridge and the two pyrrole rings adjacent to the bridgehead C atoms were linked pairwise via methylene bridges. This leads to the *D*_2*h*_ symmetric molecule **7** (Figure 8A), a compound that displays only a perimetric ring current with current densities in the range 23–33 nAT^–1^ (Figure 8B). To achieve a differentiation between the two 22π-electron macromonocycles in a near-planar macrobicycle, we replaced two CH moieties on one side by two SiH moieties, leading to **8** (Figure 8A). According to ACID, two clockwise ring currents can be detected for this species: one over the perimeter and one over one of the 22π-electron cycles (Figure 8B). As seen visually, the 22π-electron cycle with the stronger ring current is the ring with CH units. The NICS-*XY* scans also show that the aromatic character of one of the two macromonocycles decreases when going from **7** to **8**. Hence, there is an imperfect cancellation of the two 22π-electron ring currents in the butadiyne-bridge of **8**, analogous to the situation in **6** (Figure 7). GIMIC reveals current strengths of 11.7–14.4 nAT^–1^ at this bridge. Interestingly, the EDDB reveals that the extents of delocalization in the two macromonocycles of **8** are, respectively, larger and smaller than in the two equivalent macromonocycles of **7** (Figure 8C).

**Figure 9:**
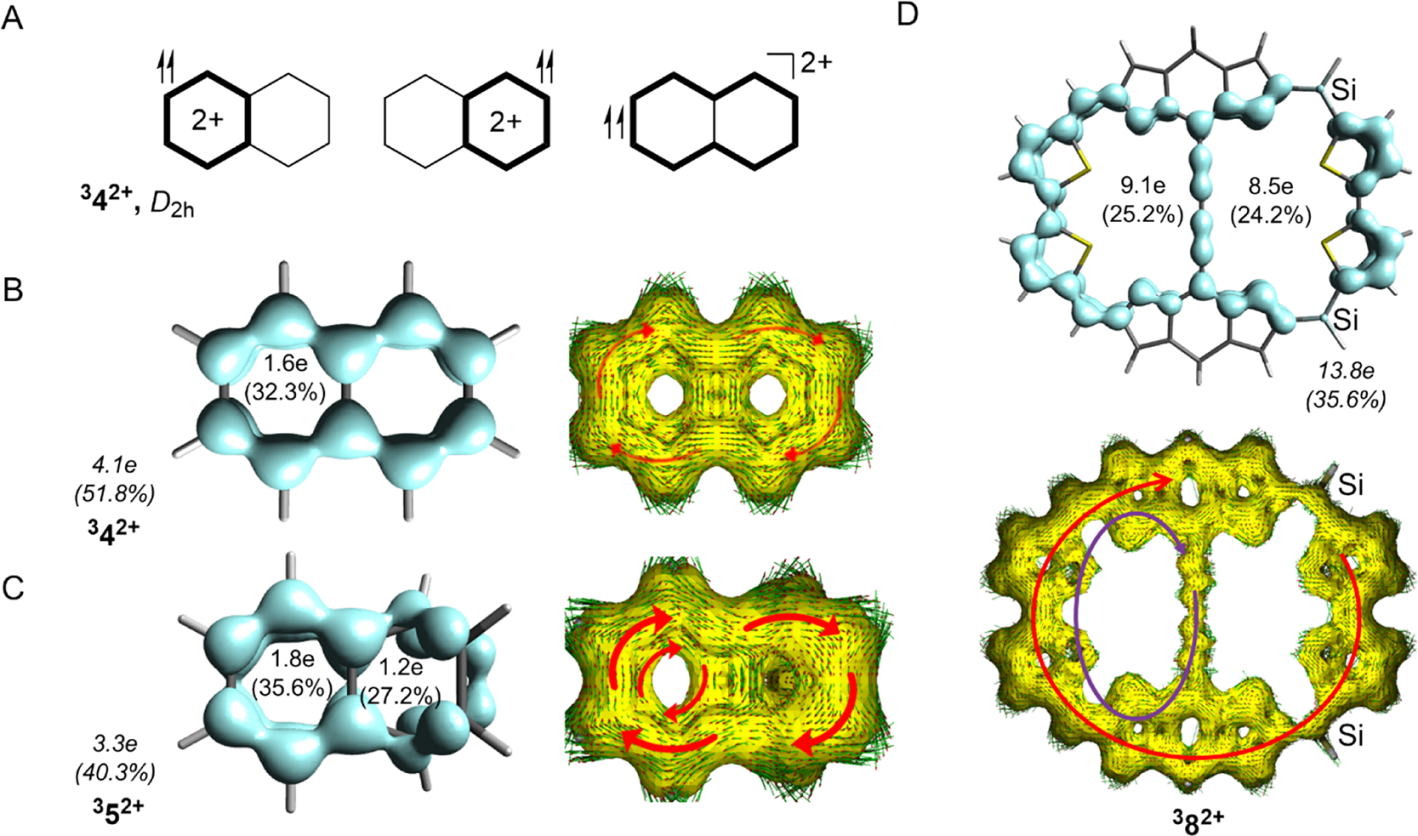
(A) Schematic resonance structures of available 4*n* π-electron circuits in naphthalene dications. Triplet state naphthalene dication in the (B) planar (**^3^4^2+^**) and (C) distorted (**^3^5^2+^**) structures, both ACID and EDDB plots, and (D) EDDB and ACID plots of **8^2+^**. For full-scale images of the ACID plots for **^3^4^2+^**, **8^2+^** and different distortions of **^3^4^2+^**, see Figures S13–S15. For the ACID plot for **7^2+^**, see Figure S16.

Furthermore, Kim and co-workers argued that the triplet state of the dication of **2** (**^3^2^2+^**) can be described as having one 33π-electron cycle in the perimeter and one 25π-electron circuit in one of the individual macrocycles.^48^ Based on the conceptual theory above, we instead reason that **^3^2^2+^** is a distorted expanded naphthalene dication, which is triplet state 2D-Baird-aromatic in the conventional sense. The naphthalene dication in its triplet state (**^3^4^2+^**) is described by three Baird-aromatic resonance structures: two with 4π-electron circuits in either of the hexagons and one with an 8π-electron perimeter (Figure 9A,B). Upon distortion leading to **^3^5^2+^**, the two 4π-electron cycles become inequivalent: one remains unaltered, while the other is weakened (Figure 9C). Similarly, **^3^2^2+^** should be described primarily by two conventional 4*n*π-electron Baird-aromatic resonance structures: one with a 32 π-electron perimetric circuit and one with a 24π-electron circuit in one of the two macromonocycles. The weakened cyclic conjugation in the ring with two Si atoms is also apparent in the EDDB plot (Figure 9D).

Next, when the middle tether of **2** and **3** is extended so that the three tethers become equal, the expanded naphthalenes turn into cage (macro)molecules such as **1**.

#### On the Aromaticity of Fully π-Conjugated Cage Macrocycles

Despite the obvious three-dimensional structures of **1** and **1^6+^** and the fact that the globally aromatic characters of the *D*_3_ symmetric molecular cages may seem apparent, they are not 3D-aromatic and do not follow the 6*n* + 2 rule. Instead their aromaticity can be understood in terms of the conventional Hückel-aromaticity of polycyclic aromatic hydrocarbons, which in most cases is two-dimensional. We therefore label these compounds as 2D-aromatic-in-3D. Also, **1** and **1^6+^** can be viewed as expanded naphthalenes (Figure 5B,C) where the middle tether has been elongated so that the π-electron counts in the three macrocyclic paths become equal. To check the aromatic character of these macrocycles, we use an electronic index, *i.e.*, EDDB, over other descriptors, considering that electron delocalization is a necessary condition for 3D-aromatics.

**Chart 1:**
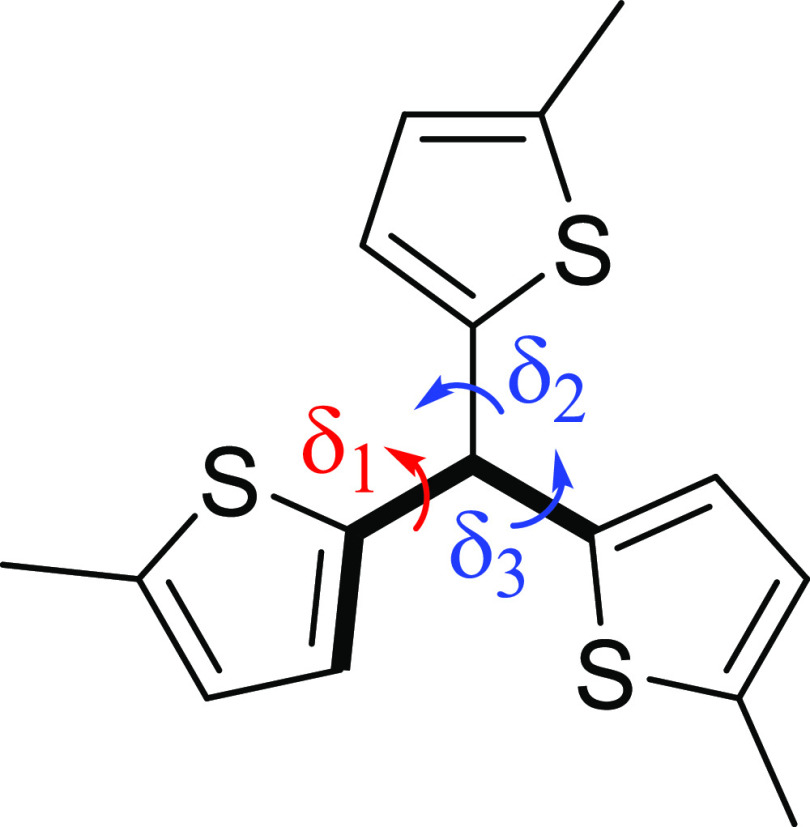
Dihedral angles (δ) at the Bridgeheads in the Molecular Cages

As noted above, the *D*_3_ symmetry does not provide the compounds with the required orbital degeneracy for 3D-aromaticity. Furthermore, in neutral **1**, the dihedral angles between the tethers at the bridgehead atoms (δ_1_, δ_2_, and δ_3_, Chart 1) are 26, 46, and 48°, which reveals that only one of the three cyclic paths is significantly π-conjugated over the two bridgeheads as the π-orbital overlap between two p_π_ AO scales as cos θ with θ being the angle between the two AOs.^91^ Yet, with the other dihedral angles in that (aromatic) cycle being 154–171°, its π-conjugation is also attenuated. It was earlier reported that when it is *D*_3_ symmetric, there is an equal aromaticity in the three rings, giving **1** aromatic character that seemingly extends over the complete molecule. However, as concluded above, the equal extent of aromaticity in the three local rings is a necessary result of the symmetry-adapted electronic structure and is not due to 3D-aromaticity. Thus, it is also not appropriately described as an aromaticity of global character. Furthermore, the *D*_3_ symmetric structure of **1^6+^** maximizes the possibility to mutually separate six positive charges. Accordingly, its enhanced aromatic character is a byproduct of charge repulsion, which is in line with earlier observations on oxidized macrocycles when compared to the corresponding neutral macrocycles.^92^ In this context, it is notable that **1^6+^** has δ_1_ = δ_2_ = δ_3_ = 41°, and with two such dihedrals in each macrocyclic ring, the π-conjugation will still be attenuated since [cos(41°)]^2^ = 0.57.

**Figure 10:**
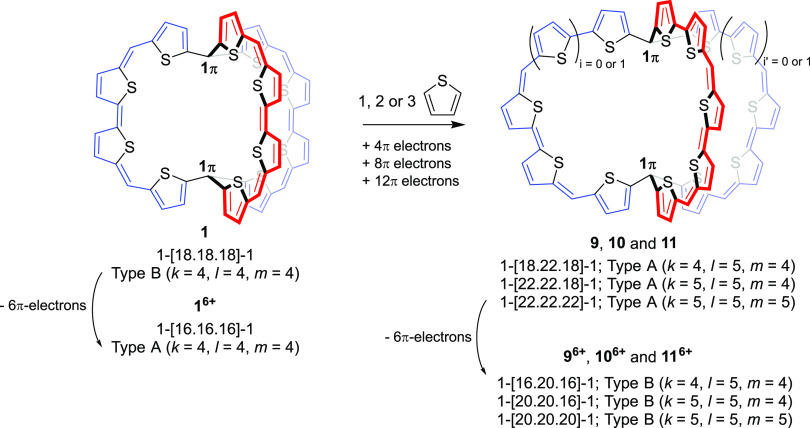
Stepwise insertion of one more thiopheno ring in each tether of molecular cage **1** leads to species with total π-electron counts of 60 (**9, *i* = *i*′ = 0**), 64 (**10, *i* + *i*′ = 1**), and 68 π-electrons (**11, *i* = *i*′ = 1**) and their hexacations.

As **1^6+^** has a π-electron count of 50, Casado and Martín described it as spherically aromatic fulfilling Hirsch’s 2(*n* + 1)^2^ rule with *n* = 4.^50^ Here, it is noteworthy that Wu and co-workers recently concluded that a similar *C*_2_ symmetric cage-type macrocycle with four tethers does not follow this rule.^71^ Now, to probe if **1^6+^** complies with Hirsch’s rule, or if the π-electron count is merely coincidental, we analyzed the next larger analogue of **1^6+^**, leading to **11^6+^** with five instead of four thiopheno rings in each tether (Figure 10). According to EDDB this hexacation exhibits slightly more π-electron delocalization than **1^6+^** (60.0% in **11^6+^** and 59.5% in **1^6+^**) and would be equally aromatic. Yet, **11^6+^** has 62 π-electrons, a number that is not a 2(*n* + 1)^2^ number (the next is 72 when *n* = 5). Instead, the slightly enhanced π-electron delocalization of **11^6+^** seems to be a result of the longer tethers, which allow for better π-orbital overlap at the bridgehead C atoms because the δ_1_ – δ_3_ values are lower in **11^6+^** than in **1^6+^** (36° *vs* 41°). Thus, it is a coincidence that the π-electron count of **1^6+^** is a 2(*n* + 1)^2^ number.

**Figure 11:**
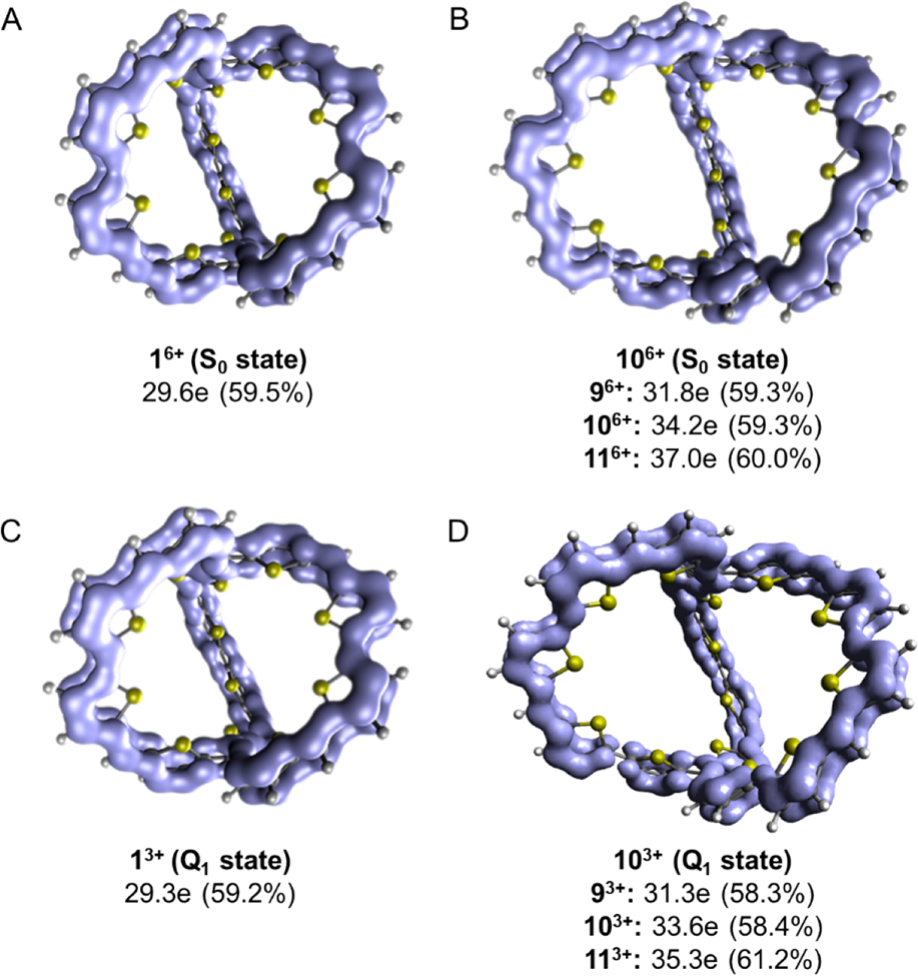
EDDB results of (A) **1^6+^** and (B) **9^6+^**, **10^6+^** (EDDB plot shown), and **11^6+^** in their closed-shell singlet ground states and (C) **1^3+^** and (D) **9^3+^**, **10^3+^** (EDDB plot shown), and **11^3+^** in their lowest open-shell quartet states. For the percentage delocalized electrons per cycle, see Table S4 in the SI.

The π-electron count of **11^6+^** is a 6*n* + 2 number. Yet, also this π-electron count does not expose 3D-aromaticity since a π-electron count of 6*n* + 2 results coincidentally for a bicyclic molecule with three equal tethers, as described in the qualitative theory section. To confirm this, we examined if cage macrocycles where one linker has four more π-electrons (or four less) than the other two linkers are similarly aromatic as **1^6+^** and **11^6+^**. We tested this through **9^6+^** and **10^6+^** (Figure 10) with total π-electron counts of 54 and 58, *i.e.*, counts that are not 6*n* + 2 numbers. We now find that the extent of π-electron delocalization according to the EDDB is similar in the four hexacations (Figure 11), revealing that it is not the specific 6*n* + 2 π-electron count in **1^6+^** and **11^6+^**, which leads to the high delocalization in these species. Instead, all four species (**1^6+^**, **9^6+^**, **10^6+^**, and **11^6+^**) are 2D-aromatic-in-3D, which should be the reason for their high electron delocalization. Notably, similar results were calculated with CAM-B3LYP as with B3LYP (see Table S4). Furthermore, by starting at **11** and shortening one of the tethers by either two or three thiopheno rings, we arrive at cage compounds **12** and **13** where the extent of delocalization starts to differ between the macrocyclic paths (see Table S4). In **12**, the electron delocalization in one circuit reaches 52%, while it is 41–42% in the other two, and in **13**, these numbers are 54 and 38%, respectively. This is even a stronger differentiation than that seen in **2**.

Finally, it was earlier argued that the dication of **1** in its triplet state (**^3^1^2+^**) is Baird-aromatic in one of the cycles since it has an NICS(0) value of −2.6 ppm,^48^ a value that suggests a modest Baird-aromaticity (if any). Following the argumentation in the section on qualitative theory, we tested if Baird-aromaticity is also achieved in the quartet state of the trication and found that **^4^1^3+^** is *D*_3_ symmetric at its global minimum having an electron delocalization of 59% (EDDB), and a similar delocalization is found in the triplet dication (**^3^1^2+^**) (62%). Furthermore, as *D*_3_ symmetry does not provide for triple degeneracy, one may ask, what do the half-filled MOs that support the high symmetry of **^4^1^3+^** look like? An inspection of the three highest α-SOMOs reveals a pair of doubly degenerate MOs and a non-degenerate totally symmetric MO (see Figure S17), leading to an orbital occupancy of the quartet trication that complies with *D*_3_ symmetry. This leads to a symmetric distribution of the unpaired electrons and explains why the trication in its quartet state can keep *D*_3_ symmetry despite the fact that there are no triply degenerate sets of orbitals.

### Search for Truly 3D-Aromatic π-Conjugated Cage Molecules

Now, can one design π-conjugated molecules that have the necessary highly symmetric structures (*e.g.*, *T_d_* or *O_h_*) and which are true 3D-aromatics? Four conditions should be satisfied according to the definition given in the Introduction. Both the 6*n* + 2 electron count and the orbital topology requirements must be fulfilled in such molecules, and the molecular properties should be similar in all three directions. Furthermore, they should exhibit extensive electron delocalization in radially oriented π-orbitals, and it should be larger than the delocalization for analogous species with other electron counts than 6*n* + 2. In the theory section, we outlined the design of tetrahedral and cubic π-conjugated molecules, which may be true 3D-aromatics (see Figure 6A). The design starts at the tetrahedral E_4_^4–^ and cubic E_8_ species, and we insert, respectively, six and twelve π-conjugated linkers between the vertex atoms in the two structures. Carbon atoms were chosen at the vertices as they provide for stronger π-conjugation than the heavier Group 14 elements, which are found in tetrahedral Zintl ions earlier labeled as 3D-aromatic.^36^ For the tetrahedral species, we also considered N atoms at the vertices but they lowered the π-electron delocalization (see Figure S18). With four anionic sp^3^-hybridized C atoms (tetrahedral species) or eight neutral sp^2^-hybridized C atoms (cubic species), we formally have, in both cases, eight radially oriented electrons at the vertices, which can conjugate with the electrons in the radial π-orbitals of the linkers. Both polyene and polyyne linkers were considered, yet we start with the rigid polyyne linkers butadiynyl, hexatriynyl, or octatetraynyl, which contribute with four, six, or eight electrons to radially oriented π-orbital frameworks.

**Figure 12:**
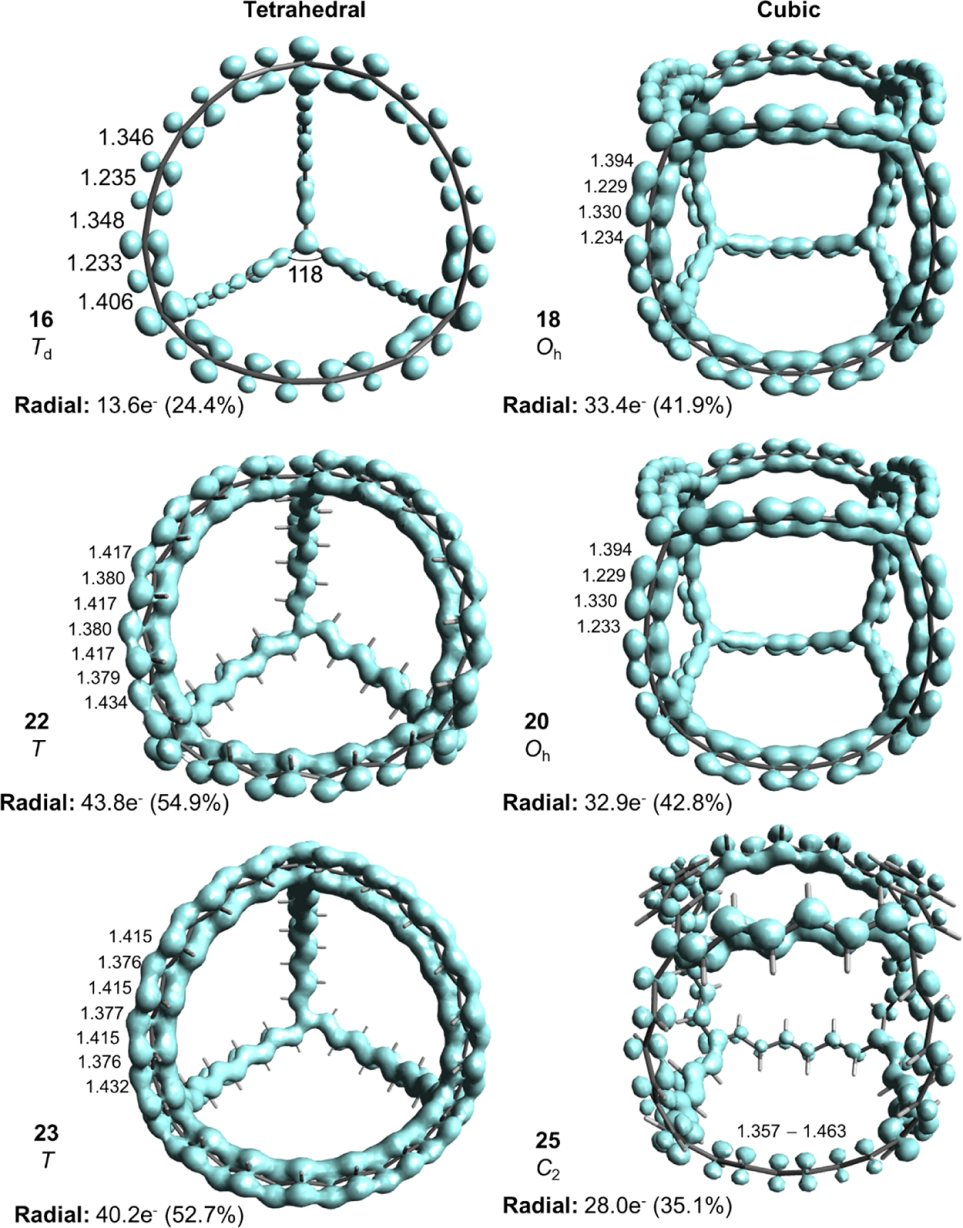
Structures displaying the bond lengths as well as the EDDB plots of C_4_(C_8_)_6_^4–^ (**16**), C_4_(C_12_H_12_)_6_^4–^ (**22**), C_4_(C_12_H_12_)_6_^–^ (**23**) (quartet state), C_8_(C_6_)_12_ (**18**), C_8_(C_6_)_12_^3+^ (quartet state, **20**), and C_8_(C_6_H_6_)_12_ (**25**). Distances are in Å, and angles are in deg. The EDDB results based on all radial π-MOs and on all MOs are both given. Essential molecular orbitals are found in Figures S21 and S22 of the SI.

The C_4_(C_*q*_)_6_^4–^ species (*q* = 4 (**14**), 6 (**15**), or 8 (**16**)) are *T_d_* symmetric, and the π-electron delocalization increases with the linker length from 14% in **14** to 24% in **16**. Thus, the delocalization in the radial π-orbital framework of **16** (Figure 12), which is bent, is slightly lower than that of planar furan (27%) but modest when compared to that of pyrrole (48%). When calculated with CAM-B3LYP, the delocalization in **16** decreases to 15%, although the molecule keeps its tetrahedral structure. We checked the possible multiconfigurational character of these tetrahedral and cubic systems by computing the *T*_1_ diagnostic values for **14** and **15** at the CCSD(T)/6-311G(d,p)//B3LYP/6-311G(d,p) level. The values obtained were below 0.02, which is the threshold for single-configurational character of closed-shell species,^93^ justifying our use of single-reference methods. Interestingly, for the cubes C_8_(C_4_)_12_ (**17**) and C_8_(C_6_)_12_ (**18**), the extents of delocalization of the radial π-electrons are higher at 43 and 42%, respectively. Based on the CC bond lengths within the hexatriyne segments of **18** (1.229–1.330 Å) as compared to those of 1,3,5-hexatriyne (1.209–1.356 Å), it is furthermore clear that there is some degree of bond length equalization in **18** indicative of enhanced bond delocalization, which may suggest aromaticity.

Zintl ions such as Si_4_^4–^ and white phosphorous P_4_ have earlier been concluded to be 3D-aromatic based on NICS(0) values computed in the tetrahedron centers.^36^ Yet, as the electron delocalization decreases with shorter tethers, it should be the lowest in the E_4_^4–^ species. Indeed, according to the EDDB, these species are devoid of electron delocalization in the valence subshell because merely 0.2041 and 0.2075e are delocalized in P_4_ and Si_4_^4–^, respectively, corresponding to 1% of the total number of valence electrons in the two molecules (see Discussion D in the Supporting Information). This is a clear case where electron indices and magnetic descriptors are not connected. Since substantial electron delocalization is a necessary condition for a 3D-aromatic compound, the EDDB results indicate that these compounds should not be labeled as such. The reason for the highly negative NICS values observed earlier^36^ requires a detailed computational study, which is outside the scope of the present work. The lack of electron delocalization in P_4_ and Si_4_^4–^ is in contrast to *closo*-boranes, which exhibit extensive delocalization (see Discussion E in the Supporting Information) and in contrast to certain small charged fullerenes.^29,65^

**Figure 13:**
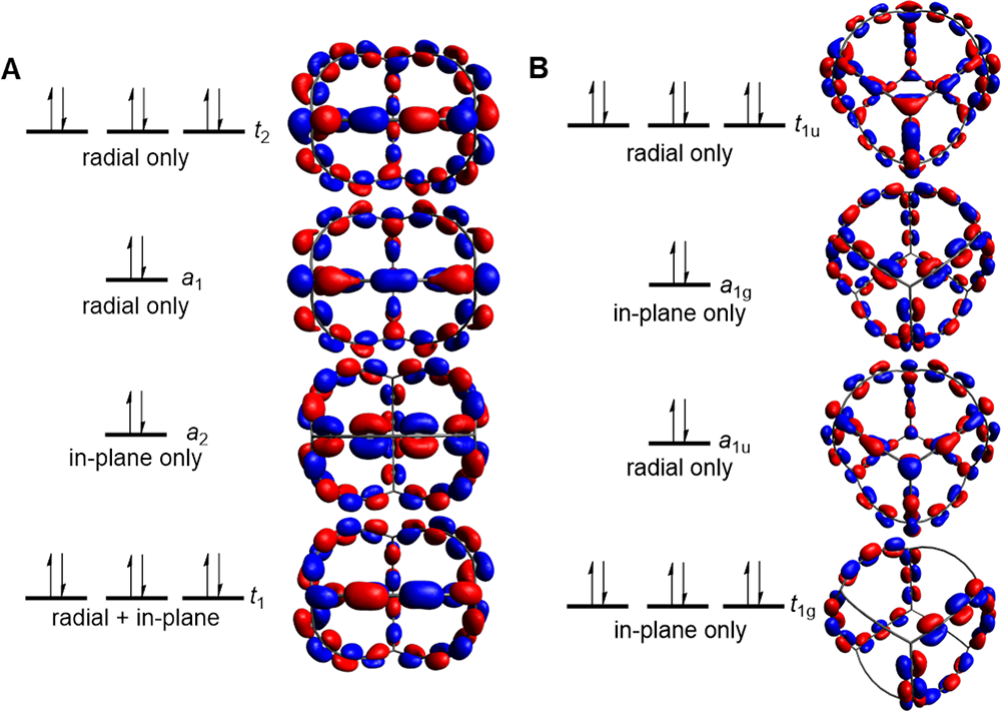
Highest few occupied molecular orbitals (A) C_4_(C_8_)_6_^4–^ (**16**) (*T_d_* symmetric) and (B) C_8_(C_6_)_12_ (**18**) (*O_h_* symmetric) at the optimal B3LYP/6-311G(d,p) geometries. The symmetry of the orbitals is also specified. For full-image orbital plots, see Figures S21 and S22.

As **16**, **17**, and **18** formally have 6*n* + 2 π-electrons that can be assigned to radially oriented π-MOs, and since they exhibit weak electron delocalization, one may ask if the highest occupied MO levels all are triply degenerate and radially oriented. A visual inspection shows that this is not the case for **16** but for **17** and **18** (Figure 13). Although the HOMO of **16** is triply degenerate and described purely by radially oriented p_π_ AOs, HOMO-1 and HOMO-2 are non-degenerate, and HOMO-3 is a mix of radially and in-plane oriented p_π_. Thus, **16** should not be labeled as a 3D-aromatic that fulfills the 6*n* + 2 rule for tetrahedral or octahedral molecules, and the same applies to **14** and **15**. Clearly, as the local bond orbitals with either in-plane or radial orientations mix for the tetrahedra, the polyyne linkers are not suitable for construction of tetrahedral π-conjugated 3D-aromatics. In contrast, in the cubic **18**, combinations of radially and in-plane oriented p_π_ AOs are only found in orbitals of very low energy (HOMO-59 and HOMO-67).

Instead of polyyne linkers in the tetrahedra, we turned to all-*E*-polyenes in their all-s-*trans* conformations, leading to the *T* symmetric C_4_(C_10_H_10_)_6_^4–^ (**21**) and C_4_(C_12_H_12_)_6_^4–^ (**22**). These two compounds only have radially oriented π-orbitals. Moreover, the EDDB reveals that **21** and **22** have significantly stronger radial π-electron delocalization (41 and 55%, respectively) compared to those with polyyne linkers. Interestingly, for the cubic species such as C_8_(C_4_H_4_)_8_ (**24**) and C_8_(C_6_H_6_)_8_ (**25**), the polyene linkers lead to structures that are distorted (*C*_2_ symmetric) with a weaker delocalization (35%) than that found for the corresponding polyyne-linked species (Figure 12). Furthermore, they exhibit significant bond length alternations as seen for **25**.

Now, if the polyene-linked C_4_^4–^ and the polyyne-linked C_8_ species are truly 3D-aromatic, then similar compounds but with electron counts that differ from 6*n* + 2 should be non-aromatic and exhibit lower electron delocalization. Yet, this is not the case for neither of the species. Only a minutely decreased delocalization is calculated when going from the tetrahedral **22** to C_4_(C_12_H_12_)_4_(C_10_H_10_)_2_^4–^ (**26**) (55% in **22** and 54% in **26**). The same is observed for the cubic species, and sometimes the delocalization is even minutely increased (*e.g.*, 41% in **18** and 42% in C_8_(C_4_)_8_(C_6_)_4_ (**27**)).

Finally, we explored if 3D-Baird-aromatic structures can be reached by removal of three electrons from **22** leading to the quartet state ^4^C_4_(C_12_H_12_)_6_^–^ (**23**) and from **17** and **18** to the quartet state ^4^C_8_(C_4_)_12_^3+^ (**19**) and ^4^C_8_(C_6_)_12_^3+^ (**20**) (Figure 12). These three species adopt, respectively, *T* and *O_h_* symmetric minima, while the triplet dications, as predicted above, drift away from the high symmetries due to Jahn–Teller distortions. Furthermore, the EDDB reveals that **23** has a very similar extent of delocalization (53%) to the closed-shell **22** (55%). To check if **23** has some true 3D-Baird-aromatic character, we examined two similar compounds, C_4_(C_12_H_12_)_4_(C_10_H_10_)_2_^–^ (**28**) and C_4_(C_12_H_12_)_2_(C_10_H_10_)_4_^–^ (**29**), for which the electron counts differ from 6*n* – 1. However, similar extents of delocalization (53% for both) as for **23** were calculated. Next, in the case of the tricationic cubes in their quartet state (**20–22**), the delocalization is very similar to their respective neutral analogues in the singlet ground state. In cubes that do not follow the rule (such as C_8_(C_4_)_4_(C_6_)_8_^3+^ (**30**) and C_8_(C_6_)_4_(C_4_)_8_^3+^ (**31**)), the delocalization remains almost unchanged (42 and 41%, respectively) compared to that of **20** (43%). Consequently, it becomes clear that also the potential 3D-Baird-aromatic character of **23** in its quartet state is unlikely.

Taken together, we have revealed the sincere difficulty to design π-conjugated molecules (neutral or charged) that are 3D-aromatic in the true sense.

## Conclusions

Using qualitative theory combined with quantum chemical calculations, we came up with key points that help in discerning between regular 2D-aromaticity, albeit in a 3D molecular structure, and true 3D-aromaticity in three-dimensional π-conjugated (cage) (macro)molecules. Our study revealed that compounds **1^6+^** and **2** (and **3**) should not be labeled as, respectively, 3D-aromatic and bicycloaromatic. The basic prerequisite for 3D-aromaticity, besides 6*n* + 2 π-electron counts and high π-electron delocalization, is a highly symmetric structure with at least triply degenerate MOs, features that do not exist in **1^6+^** (also not in approximate sense). Yet, there are clear limitations for tetrahedral and cubic cage compounds that formally fulfill the 3D-aromaticity requirements because there are negligible differences in electron delocalization between these cage compounds and near-tetrahedral or near-cubic compounds that have π-electron counts that deviate from 6*n* + 2. Hence, there seems to be a size limitation for 3D-aromaticity, similar as has been observed earlier for spherical aromaticity.^65^

Likewise, the main features of bicycloaromatic species do not exist in **2** and **3**. Instead, we find that the three macrocyclic molecules **1**–**3** and **1^6+^** are better described as 2D-aromatic-in-3D since their aromatic character originates from the presence of various 2D Hückel-aromatic circuits in three-dimensional molecular scaffolds. In that regard, we showed that there is not only a direct connection between PAHs (*e.g.*, naphthalene) and **1^6+^** and **2** (as well as **3**) but also a connection between the three macrocycles. It becomes clear that they all can be described as expanded naphthalenes.

Building on these findings, we further attempted to design 3D-aromatic species that are exclusively π-conjugated and identified caveats that make their design difficult. First is the difficulty in getting a sufficient number of triply degenerate MOs with only radial orientation. Ideally, in a highly symmetric structure, the 3D-aromatic character would only come from radial π-MOs, whereas if such orbitals exist in combination with other MO types, then the molecule is not a true 3D-aromatic. A second weakness is the fact that the extent of electron delocalization in species that fulfill the 6*n* + 2 electron count barely differs from those that do not fulfill this count.

In conclusion, no 3D-aromatic π-conjugated molecule has, to our knowledge so far, been generated experimentally as a long-lived species. At this point, we want to stress that, although we bring a different rationalization of the aromaticity in **1**–**3** and **1^6+^** than provided earlier,^48−51^ it is truly important that new compounds that stretch and provoke our understanding of chemical bonding phenomena are designed and analyzed. Healthy discussions on their chemical bonding bring chemistry forward as a science. Today, the term 3D-aromaticity is used for a number of different bonding patterns that involve aromatic interaction in three dimensions. Yet, if insufficiently well defined, it will lose its utility as a meaningful concept for chemical bonding analyses and the applications that rest upon such analyses. In our view, it is time the term is put on a solid foundation, which applies to both σ- and π-bonded systems. This description of 3D-aromaticity should comply with Aihara’s original finding on the compounds, which he labeled as 3D-aromatic.^25^

## Experimental Section

### Computational Methods

All geometry optimizations were performed with Gaussian 16^94^ at the B3LYP/6-311G(d,p) level,^74−86^ although selected species were also calculated with CAM-B3LYP.^75^ Aromaticity was evaluated in terms of electronic and magnetic indicators at the same level of theory. The electronic delocalization was evaluated by the electron density of delocalization bond (EDDB_H_),^78,79^ and magnetic properties were assessed using the nucleus-independent chemical shift (NICS),^80,81^ anisotropy of the induced current density (ACID)^84^ plots, and gauge-including magnetically induced currents (GIMIC).^85^ Regarding EDDB_H_ computations, NBO 3.1^95^ and Multiwfn have been employed, where the former together with Avogadro 1.2^96,97^ was used in obtaining EDDB_H_ surfaces. NICS-*XY* scans were performed using the Aroma package,^98^ and ACID plots were produced using the AICD 2.0.0 program.^84,99^

## Supporting Information Available

High-resolution ACID plots; table with current strengths and percentage of delocalization; EDDB plots; full image of the highest few occupied molecular orbitals; aromaticity analysis of P_4_, Si_4_^2–^, *closo*-boranes, and archetypal aromatic and antiaromatic compounds; and Cartesian coordinates and absolute energies (PDF)
