# Supplementary material for: Three-Dimensional Fully π-Conjugated Macrocycles: When 3D-Aromatic and When 2D-Aromatic-in-3D?
Source: J Am Chem Soc. 2022 May 6;144(19):8560–75. doi: 10.1021/jacs.1c13478 (PMC9121391; doi:10.1021/jacs.1c13478)
Supplement: Supplementary file 1 — ja1c13478_si_001.pdf [file ja1c13478_si_001.pdf]

# SUPPORTING INFORMATION

## Three-Dimensional Fully π-Conjugated Macrocycles: When 3D-Aromatic and when 2D-Aromatic-in-3D?

Ouissam El Bakouri [a,b], Dariusz W. Szczepanik [b,c], Kjell Jorner [a], Rabia Ayub [a], Patrick Bultinck [d], Miquel Solà*[b], and Henrik Ottosson*[a]

[a] Department of Chemistry - Ångström Laboratory, Uppsala University, Box 523, 751 20 Uppsala, Sweden. [b] Institut de Química Computacional i Catàlisi (IQCC) and Departament de Química, Universitat de Girona, C/ Maria Aurèlia Capmany 6, 17003 Girona, Catalonia, Spain. [c] K. Guminski Department of Theoretical Chemistry, Faculty of Chemistry, Jagiellonian University, Gronostajowa 2, Kraków 30-387, Poland. [d] Department of Chemistry, Ghent University, Krijgslaan 281 S3, 9000 Gent, Belgium.

## TABLE OF CONTENTS

**Figure S1.** Molecular orbitals of (A) benzene as a 2D-aromatic archetype molecule and (B) pyridine........ 4

**Figure S2**. Molecular orbitals of (A) the $B_6H_6^{2-}$ *closo*-borane as a 3D-aromatic archetype molecule and (B) the $CB_5H_6^-$ *closo*-borane........ 5

Discussion A. MO-diagrams of panel C compounds and their potential 3D-aromaticity ........ 6

**Figure S3**. Molecular orbitals of $C_{60}^{10+}$ as a spherical aromatic molecule........ 6

**Figure S4**. Molecular orbitals of the $C_{24}H_{16}$ cyclophane as a face-to-face aromatic molecule . 7

**Figure S5**. Molecular orbitals of $LiC_5H_5$ as a six-interstitial electrons aromatic molecule ...... 7

Discussion B. π-electron delocalization in archetypical aromatic compounds........ 8

**Figure S6**. The global ($EDDB_H$) and cyclic delocalization of electrons ($EDDB_P$) visualized and quantified by the corresponding EDDB functions for selected archetypical hydrocarbon aromatic and antiaromatic monocycles. The effectiveness of electron delocalization (in %) given in brackets was calculated assuming the formal number of π-electrons given above the structures. The numbers of dominating resonance forms (NDRF) were taken from the Natural Resonance Theory (NRT) analysis as implemented in the NBO7 software........ 9

**Figure S7**. ACID (isosurface: 0.025) plots of **4**, **5** and **6** in the $S_0$ state, and NICS-*XY* scan of **4** and **6** in the $S_0$ state ........ 10

**Figure S8.** ACID (isosurface: 0.025) and EDDB (isosurface: 0.015) plots of distorted naphthalenes in the $S_0$ state ........ 11

**Table S1.** Current strengths of the distorted naphthalenes in the $S_0$ state obtained from GIMIC ($nAT^{-1}$)........ 12

**Figure S9.** (A) Molecular structure of **2** without the DTP bridge and DTP optimized at the B3LYP/6-311G(d,p) level. (B) ACID plot of **2**. ........ 13

**Figure S10**. ACID plot (isosurface: 0.025) and EDDB plots (isosurface: 0.015) of **2** in its planar $C_{2v}$ structure in the $S_0$ state. ........ 14

**Figure S11**. ACID plot (isosurface: 0.025) of **7** in the $S_0$ state. ........ 15

**Figure S12**. ACID plot (isosurface: 0.025) of **8** in the $S_0$ state. ........ 15

Discussion C. Compound 8........ 16

**Figure S13**. ACID (isosurface: 0.025) plots of $\mathbf{4^{2+}}$ and $\mathbf{5^{2+}}$ in the $T_1$ state........ 17

**Figure S14**. ACID (isosurface: 0.025) and EDDB (isosurface: 0.015) plots of distorted naphthalenes dication in the $T_1$ state ........ 18

**Table S2**. Current strengths of the distorted naphthalenes in the $T_1$ state obtained from GIMIC ($nAT^{-1}$)........ 19

**Figure S15**. ACID plot (isosurface: 0.025) and EDDB plots (isosurface: 0.015) of $\mathbf{8^{2+}}$ in the $T_1$ state........ 20

**Figure S16.** ACID plot (isosurface: 0.025) and EDDB plot (isosurface: 0.015) of $\mathbf{7^{2+}}$ in the $T_1$ state........ 21

**Table S3.** Percentage of π-delocalization of **2** and its derivatives at the B3LYP/6-311G(d,p) level. ..... 22

**Table S4.** Percentage of π-delocalization computed with the EDDB method of 1 and analogous cage macromolecules 9 - 13 at the B3LYP and CAM-B3LYP level. ..... 23

**Figure S17.** Three highest α-SOMOs of $\mathbf{1^{3+}}$ in its quartet state. ..... 24

**Figure S18.** EDDB plots (isosurface: 0.015) of tetrahedral species considering N atoms in the corner. ..... 25

**Figure S19.** EDDB plots (isosurface: 0.015) of **14** and **15**. ..... 26

Discussion D. Aromaticity of $Si_4^{2-}$ and $P_4$ ..... 27

**Figure S20**: The $EDDB_G$ values (violet numbers) and contours at different isovalues for $P_4$ and $Si_4^{4-}$. The default (recommended) isovalue used in the EDDB analyses is in the range 0.010 - 0.020. ..... 27

**Figure S21**. Full-image of the highest few occupied molecular orbitals (A) $C_4(C_8)_6^{4-}$ (**16**) ($T_h$ symmetric) ..... 28

**Figure S22.** Full-image of the highest few occupied molecular orbitals of $C_8(C_6)_{12}$ (**18**) ($O_h$ symmetric) ..... 29

**Figure S23.** EDDB plots (isosurface: 0.015) of **17**, **19**, **21**, **24**, **25** and **26**. Percentage of π-delocalization in parenthesis. ..... 30

**Figure S24.** EDDB plots (isosurface: 0.015) of **27** - **31**. Percentage of π-delocalization in parenthesis. ..... 31

**Table S5.** Percentage of π-delocalization computed with the EDDB method of cage macromolecules **16**, **18**, **20**, **22**, **23** and **25** at the CAM-B3LYP level. ..... 32

Discussion E. Electron delocalization in *closo*-borane $B_{12}H_{12}^{2-}$ and its derivatives ..... 33

**Figure S25.** EDDB plots of *closo*-borane $B_{12}H_{12}^{2-}$, $CB_{11}H_{12}^{-}$ and *o*-$C_2B_{10}H_{12}^{2-}$. Percentage of π-delocalization. ..... 33

Description of EDDB ..... 34

**Figure S26**. The renormalized percentage contributions of the π-resonance forms in the aromatic $C_7H_7^+$ (blue numbers) and formally antiaromatic $C_7H_7^-$ obtained from the Natural Resonance Theory (NRT[7]) module implemented in the NBO7 software. ..... 36

References ..... 37

XYZ coordinates ..... 38

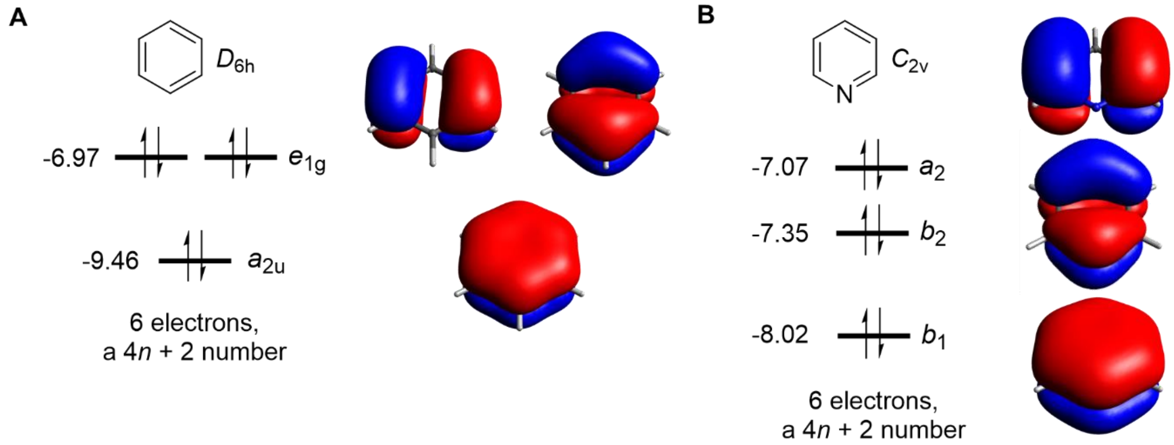


**Figure S1.** Molecular orbitals of (A) benzene as a 2D-aromatic archetype molecule and (B) pyridine. Orbital energies in eV.

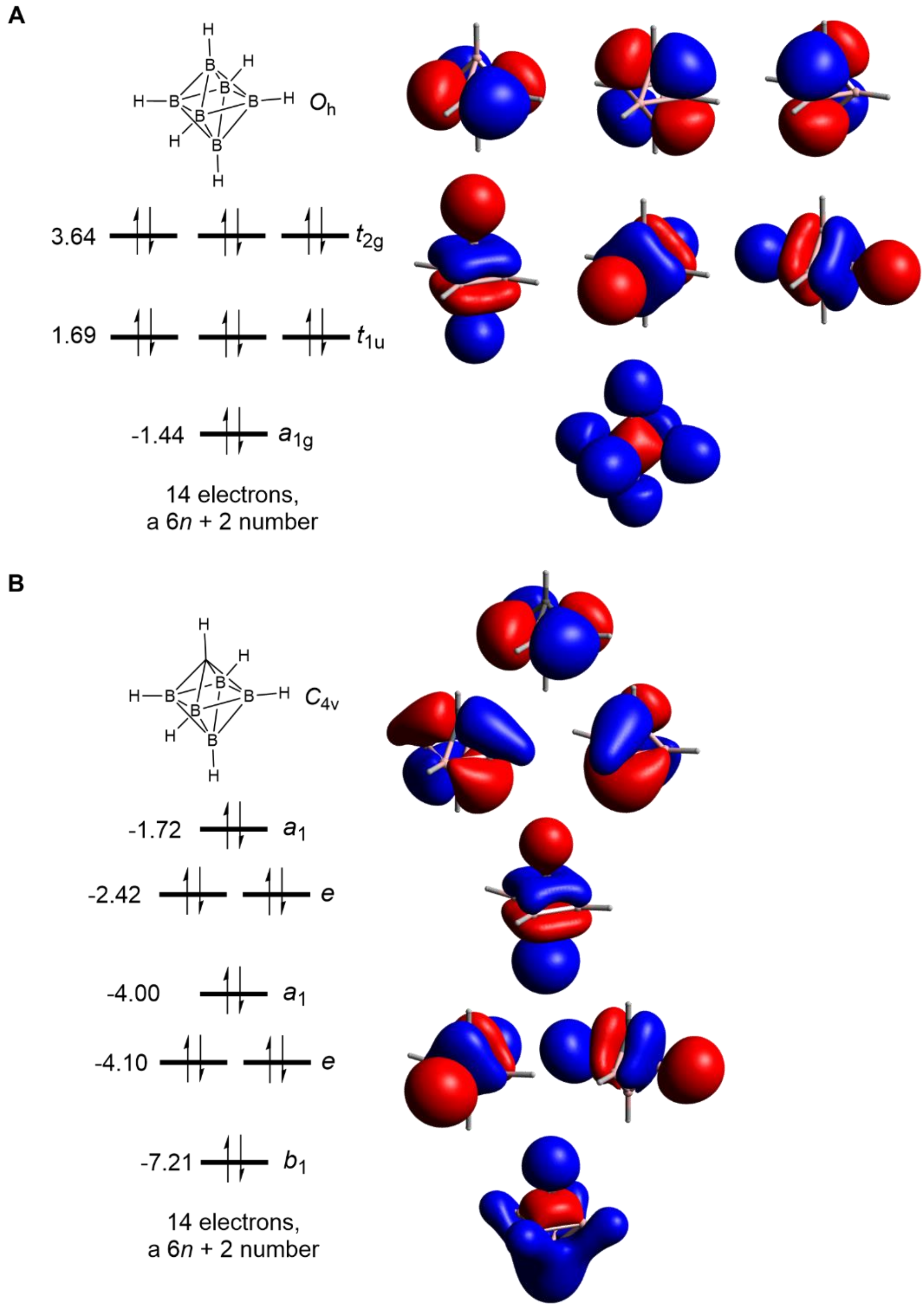


**Figure S2**. Molecular orbitals of (A) the $B_6H_6^{2-}$ *closo*-borane as a 3D-aromatic archetype molecule and (B) the $CB_5H_6^-$ *closo*-borane. Orbital energies in eV.

## Discussion A. MO-diagrams of panel C compounds and their potential 3D-aromaticity

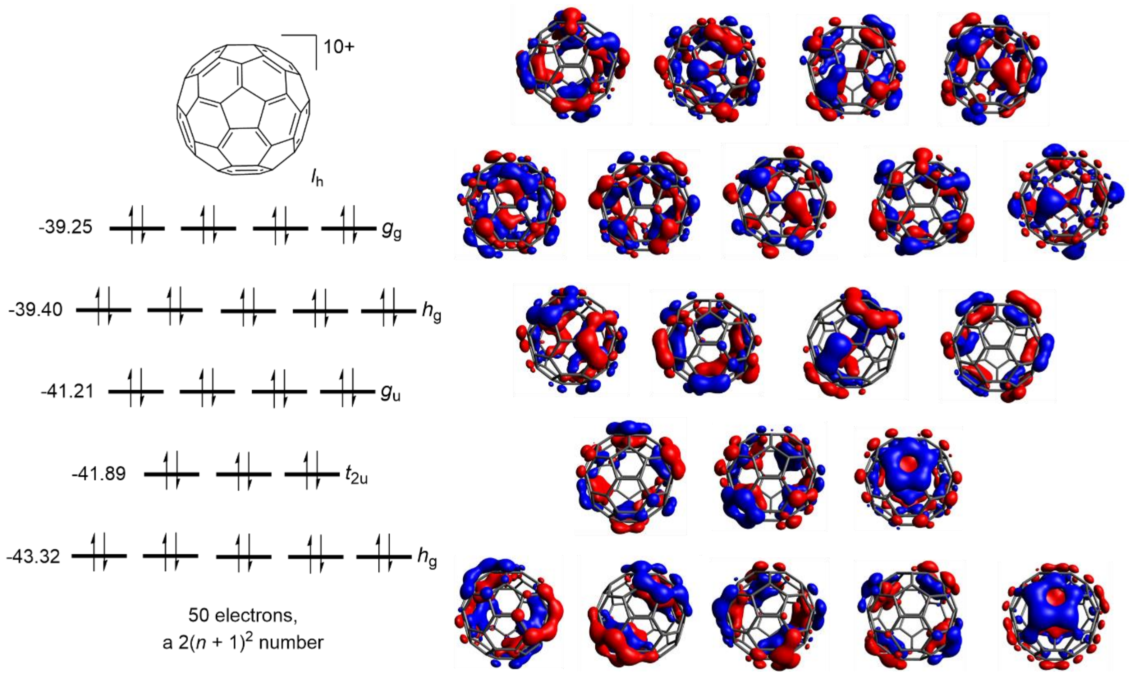


**Figure S3**. Molecular orbitals of $C_{60}^{10+}$ as a spherical aromatic molecule. Orbital energies in eV.

The spherical aromatic character of $C_{60}^{10+}$ comes from the fact that it obeys Hirsch's rule as it has 50 $\pi$-electrons, *i.e.*, a $2(n+1)^2$ number, and that it exhibits a high degree of electron delocalization over the complete molecule and it adopts a highly symmetric structure ($I_h$) with triply, quadruply and quintuply degenerate molecular orbitals as seen above for the highest few occupied MOs.

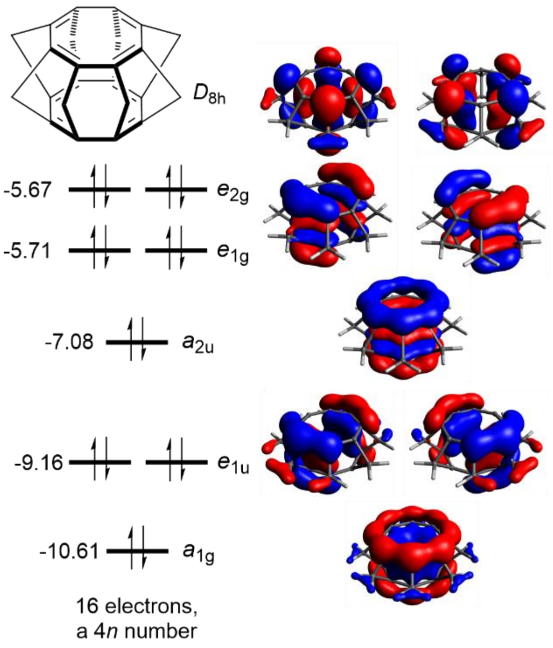


**Figure S4**. Molecular orbitals of the $C_{24}H_{16}$ cyclophane as a face-to-face aromatic molecule. Orbital energies in eV.

This compound has 16 π-electrons and it is constructed by bridging two [8]annulenes, *i.e.*, cyclooctatetraene molecules. Such a structure allows significant transannular orbital interactions leading to a through-space aromatic character in three dimensions. This leads to a stabilization despite the fact that the molecule has a total of 4*n* π-electrons. However, these features are not sufficient to be labeled as a 3D-aromatic species as there also needs to be at least triply degenerate molecular orbitals, a characteristic that cyclophanes do not have. Thus, such compounds do not satisfy all the conditions for 3D-aromaticity.

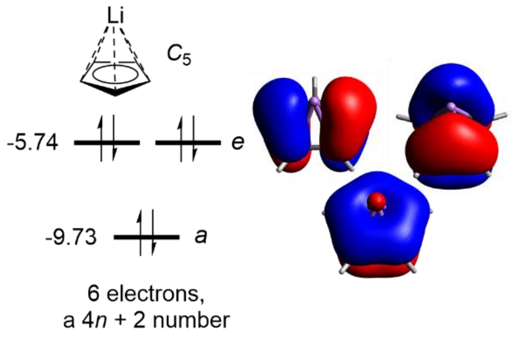


**Figure S5**. Molecular orbitals of $LiC_5H_5$ as a six-interstitial electrons aromatic molecule. Orbital energies in eV.

This is a case where an annulene interacts with capping groups or atoms (a metal in this case). This was earlier described as a form of 3D-aromaticity,[1] however, apart from not having 6*n* + 2 electrons, these molecules have no triply degenerate orbitals. Consequently, these species should not be labelled as 3D-aromatics.

**Discussion B. π-electron delocalization in archetypical aromatic compounds**

Figure S6 present the results of the $EDDB_H$ and $EDDB_P$ analyses of the pi-electron delocalization in archetypical aromatic compounds, $C_4H_4^{2+}$, $C_5H_5^-$, $C_6H_6$, and $C_7H_7^+$, and the corresponding antiaromatics, $C_4H_4$, $C_5H_5^+$, $C_6H_6^{2+}$, and $C_7H_7^-$. Even at first sight it is clear that in aromatic compounds the electrons are distributed uniformly over the entire ring, while in antiaromatic system the Jahn-Teller distortion dramatically disrupts the electron delocalization, and, depending on the symmetry of the system, the number of dominating resonance forms (NDRF) is significantly reduced from 4-7 to 1-3. Thus, the antiaromatics may still reveal some of the local resonance effects, which is particularly evident in in $C_7H_7^-$ where the close degeneracy of 3 dominating resonance forms results in the electron delocalization between 5 carbon atoms. However, in the cases of all 4*n* rings the $EDDB_P$ function clearly shows the lack of cyclic delocalization marking them not aromatic. It should be noted that in the aromatic systems containing the *n*c-2e bonds (like $C_4H_4^{2+}$) the EDDB method accounts only the kekulean delocalization (1.000e), the cross-ring delocalization (0.500e), but by definition it does not take into account the resonance between forms having no bond (for a single π-MO in the cyclobutadiene dication we have in total four possible resonance forms with a lone-pair localized on one carbon atom). These forms are necessary to describe completely the wavefunction but they do not contribute to the electron delocalization. In fact, the 50% effectiveness of cyclic delocalization in $C_4H_4^{2+}$ is not enough to hold the system planar and it experiences noticeable distortion from planarity at its equilibrium ground-state geometry.

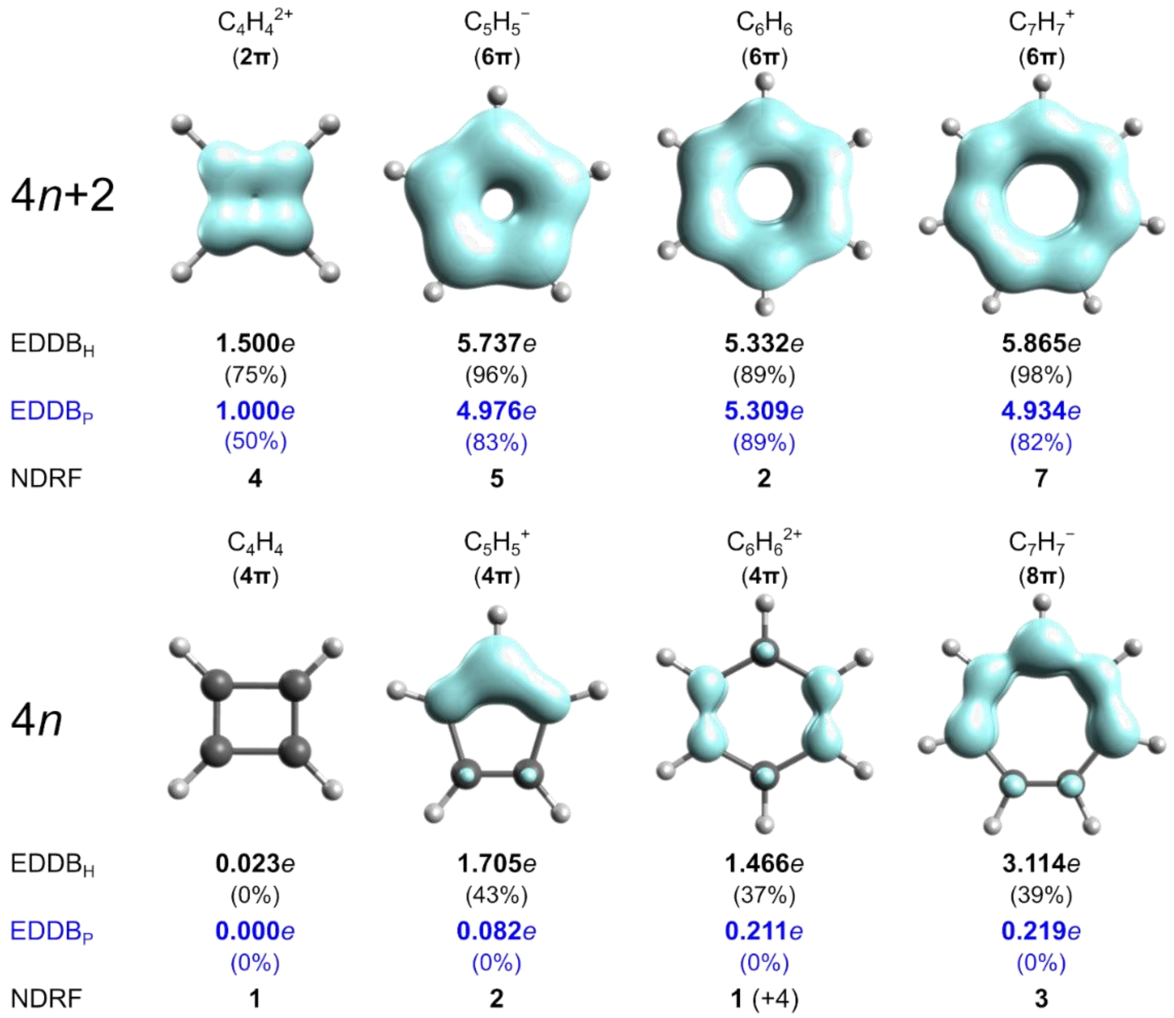


**Figure S6**. The global ($EDDB_H$) and cyclic delocalization of electrons ($EDDB_P$) visualized and quantified by the corresponding EDDB functions for selected archetypical hydrocarbon aromatic and antiaromatic monocycles. The effectiveness of electron delocalization (in %) given in brackets was calculated assuming the formal number of π-electrons given above the structures. The numbers of dominating resonance forms (NDRF) were taken from the Natural Resonance Theory (NRT) analysis as implemented in the NBO7 software. Method: B3LYP/6-311G(d,p).

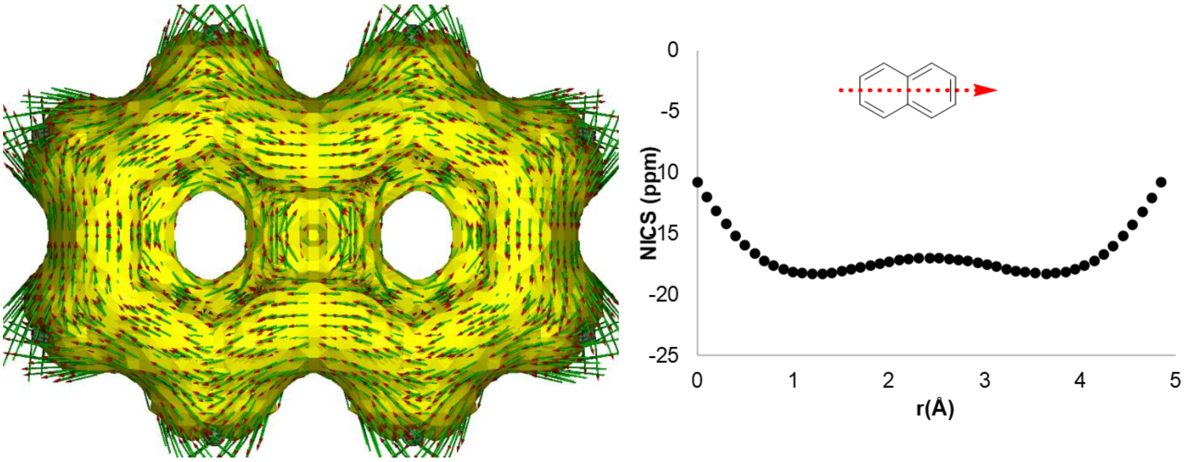


Naphthalene (**4**)

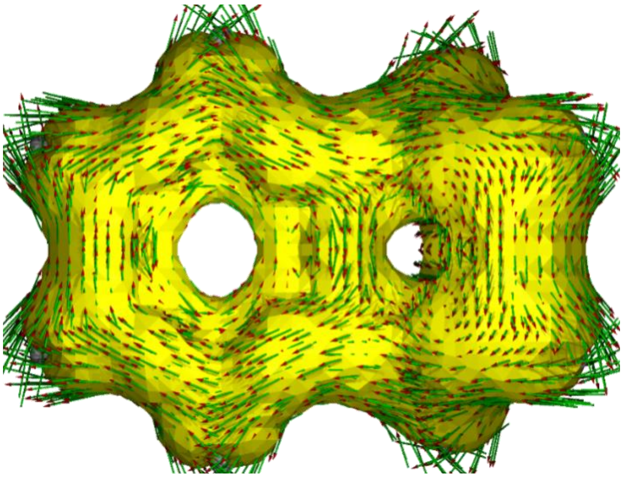

Distorted naphthalene (**5**, 60 degrees)

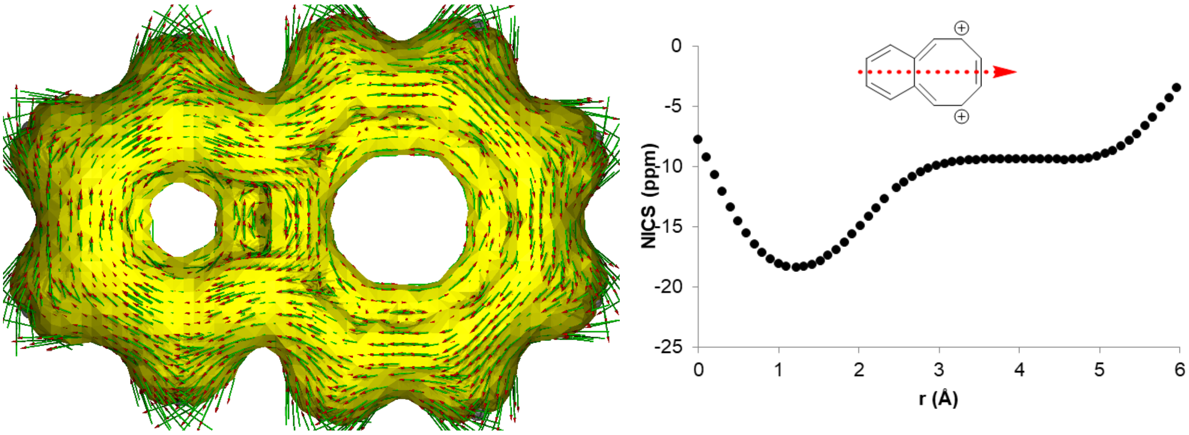


BenzoCOT dication (**6**)

**Figure S7**. ACID (isosurface: 0.025) plots of **4**, **5** and **6** in the $S_0$ state, and NICS-*XY* scan of **4** and **6** in the $S_0$ state at the B3LYP/6-311G(d,p) level.

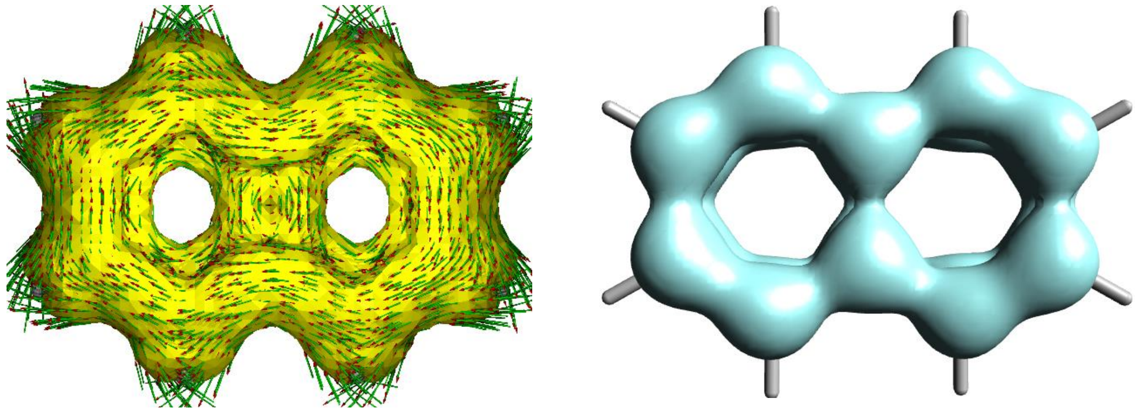

Naphthalene (15º degrees)

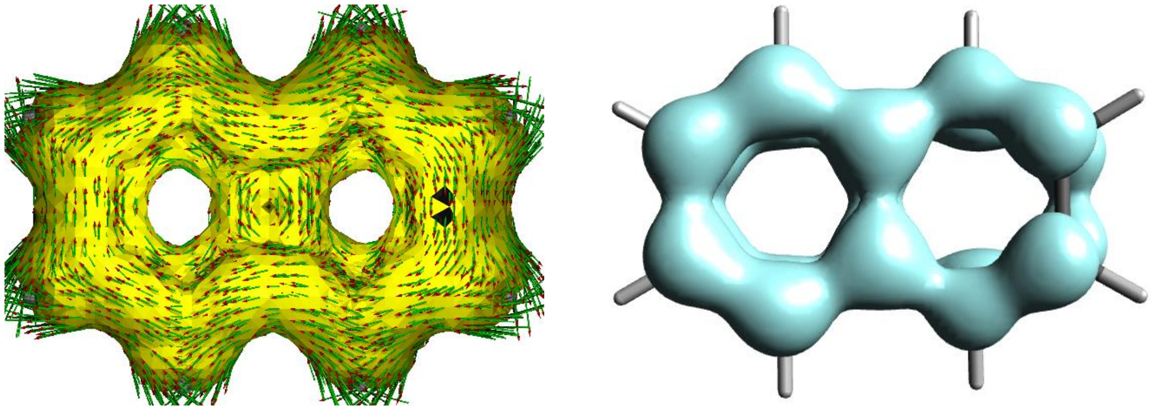

Naphthalene (30º degrees)

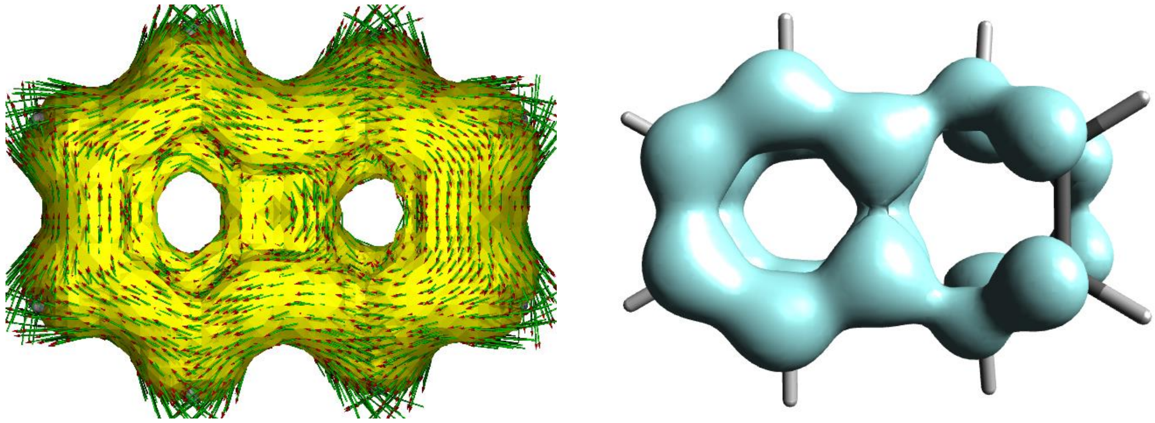

Naphthalene (45º degrees)

**Figure S8.** ACID (isosurface: 0.025) and EDDB (isosurface: 0.015) plots of distorted naphthalenes in the $S_0$ state at the B3LYP/6-311G(d,p) level.

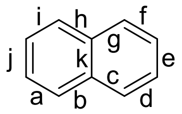


**Table S1.** Current strengths of the distorted naphthalenes in the $S_0$ state obtained from GIMIC ($nAT^{-1}$).

| *$S_0$* | *0º* | *15º* | *30º* | *45º* | *60º* |
|---|---|---|---|---|---|
| *a* | 11.7 | 12.3 | 11.8 | 12.4 | 12.3 |
| *b* | 12.1 | 12.5 | 12.1 | 12.4 | 12.2 |
| *c* | 12.1 | 11.9 | 9.9 | 8.1 | 6.5 |
| *d* | 11.6 | 11.7 | 9.6 | 8.0 | 5.4 |
| *e* | 19.3 | 19.3 | 18.8 | 18.2 | 16.4 |
| *f* | 11.7 | 8.4 | 3.1 | 1.5 | -0.8 |
| *g* | 12.1 | 12.1 | 10.6 | 9.1 | 7.1 |
| *h* | 12.1 | 12.4 | 11.8 | 11.8 | 11.3 |
| *i* | 11.6 | 12.2 | 11.8 | 12.4 | 12.3 |
| *j* | 19.3 | 19.3 | 18.7 | 17.8 | 15.2 |
| *k* | 0.0 | 0.2 | 0.8 | 1.9 | 4.9 |

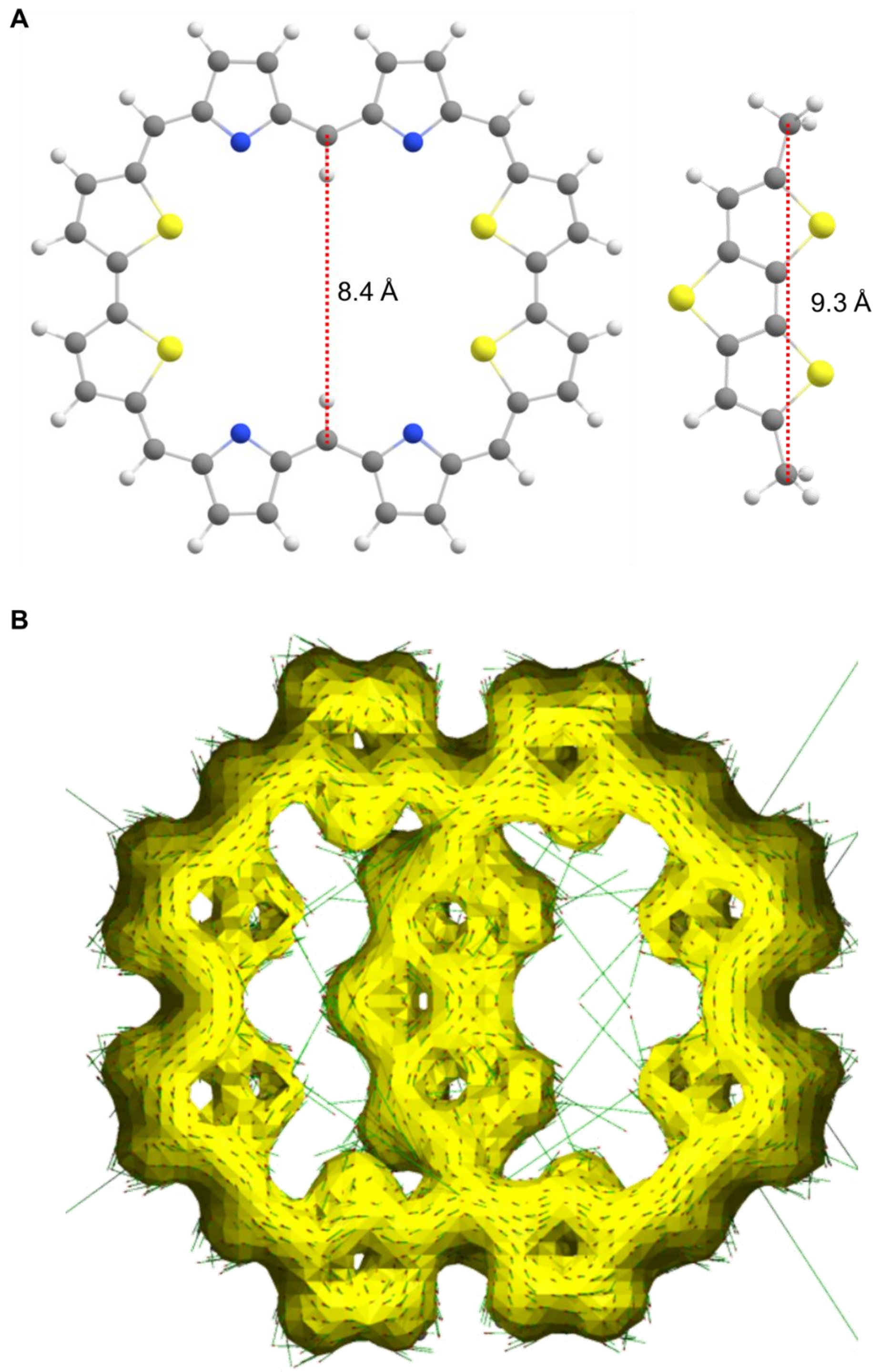


**Figure S9.** (A) Molecular structure of **2** without the DTP bridge and DTP optimized at the B3LYP/6-311G(d,p) level. (B) ACID plot of **2**.

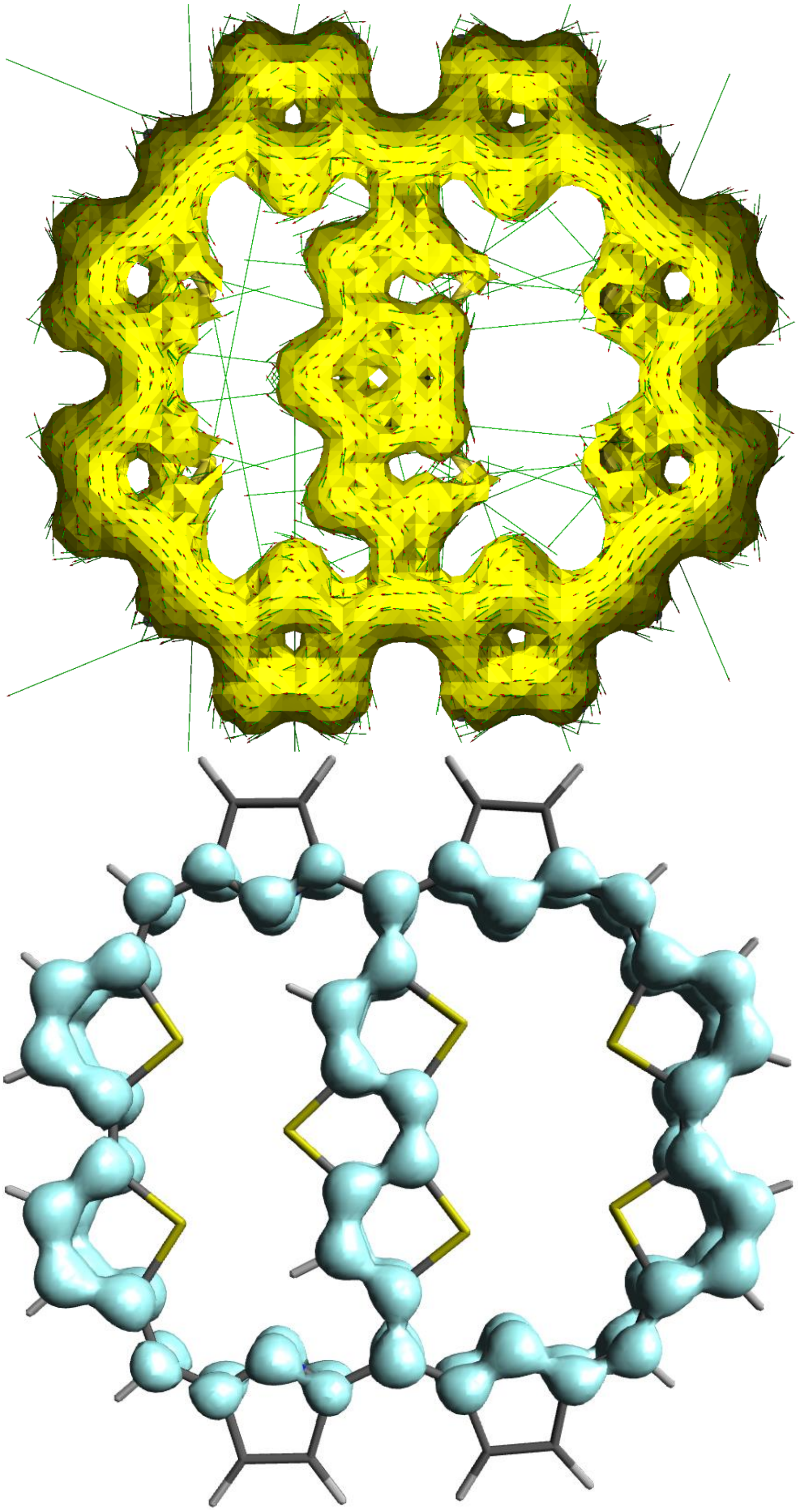

2 ($C_{2v}$, $S_0$)

**Figure S10.** ACID plot (isosurface: 0.025) and EDDB plots (isosurface: 0.015) of **2** in its planar $C_{2v}$ structure in the $S_0$ state.

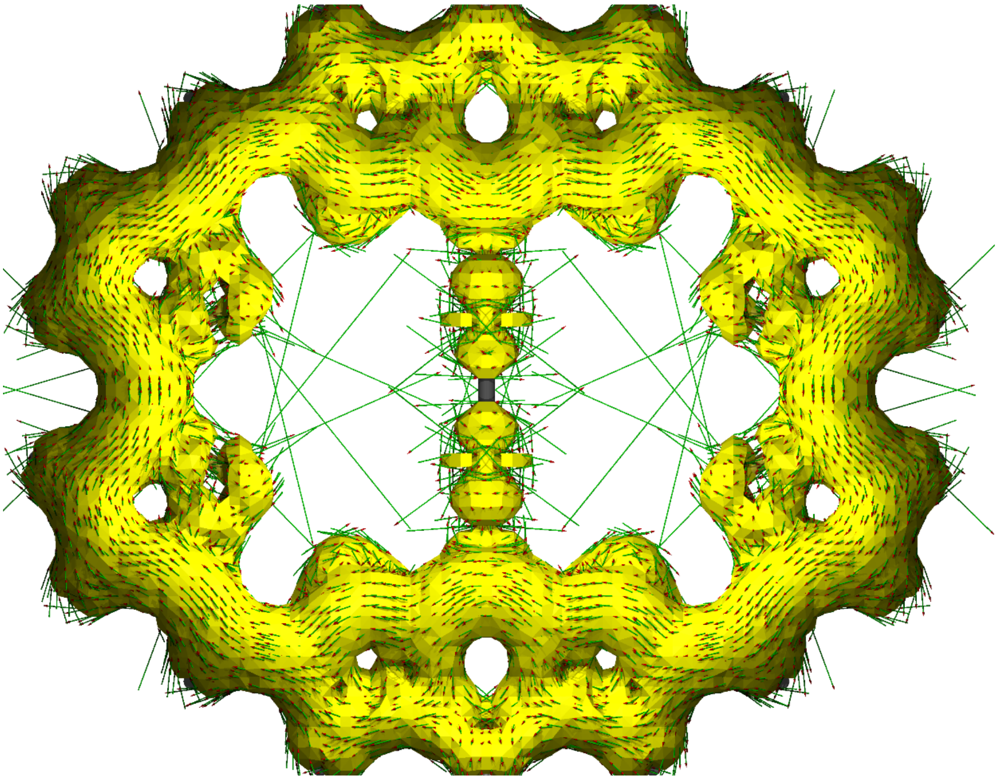

**Figure S11**. ACID plot (isosurface: 0.025) of **7** in the $S_0$ state.

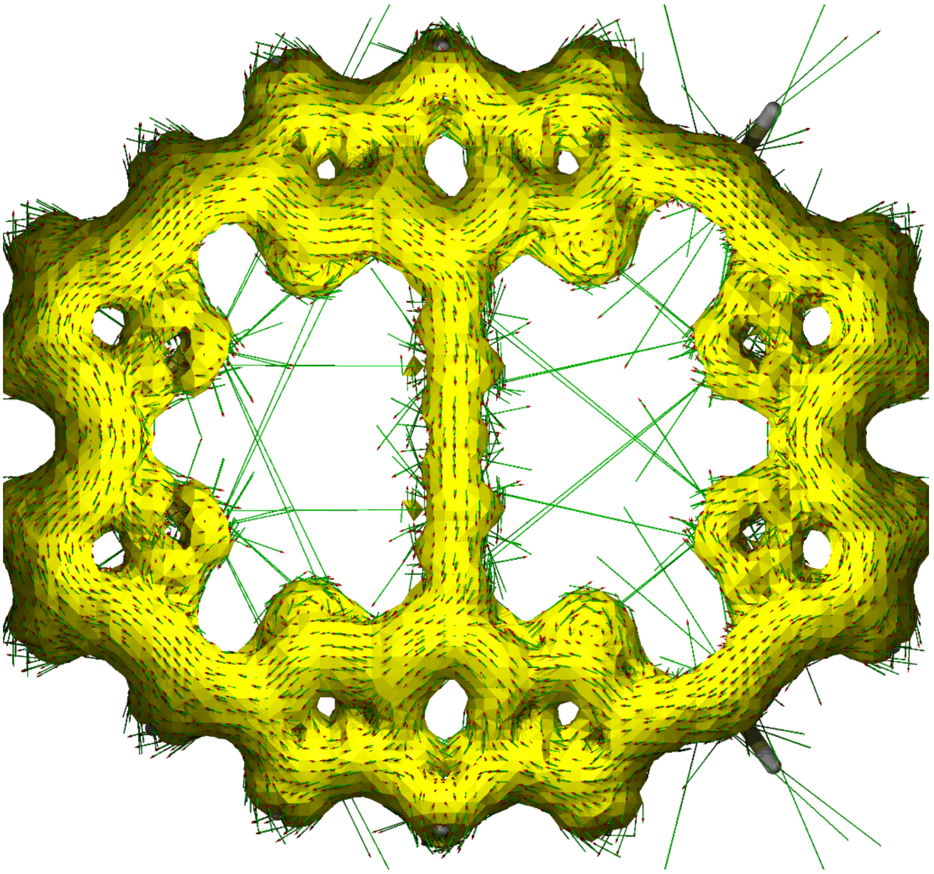

**Figure S12**. ACID plot (isosurface: 0.025) of **8** in the $S_0$ state.

**Discussion C. Compound 8**

EDDB shows a clear attenuation in the electron delocalization at the Si atoms and the C-Si bonds since EDDB is more sensitive than ACID. At higher isovalues this is observed as two discontinuities in the plots, and at lower isovalues as smaller contributions at the Si atoms. This indicates that the π-conjugation in compound **8** with Si=C bonds is weaker than in **7**, a result of attenuated $p_\pi$-orbital overlap between the 3p(Si) and 2p(C) atomic orbitals.

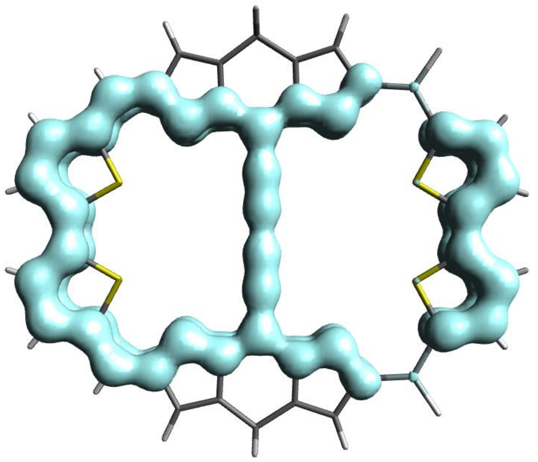

0.012

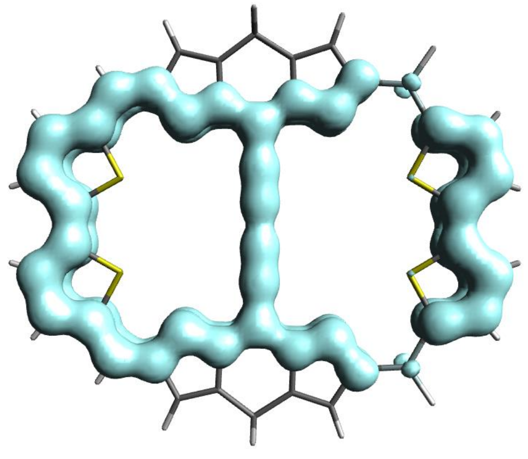

0.010

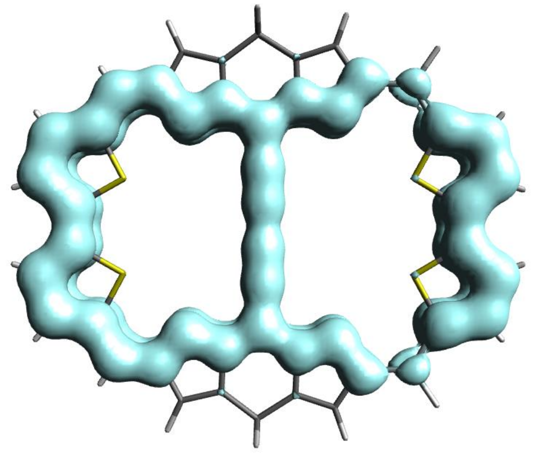

0.008

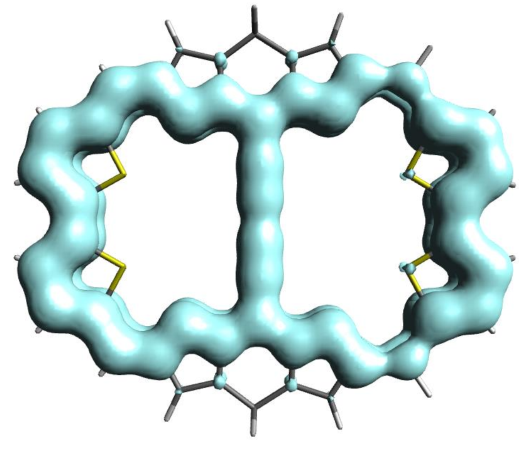

0.006

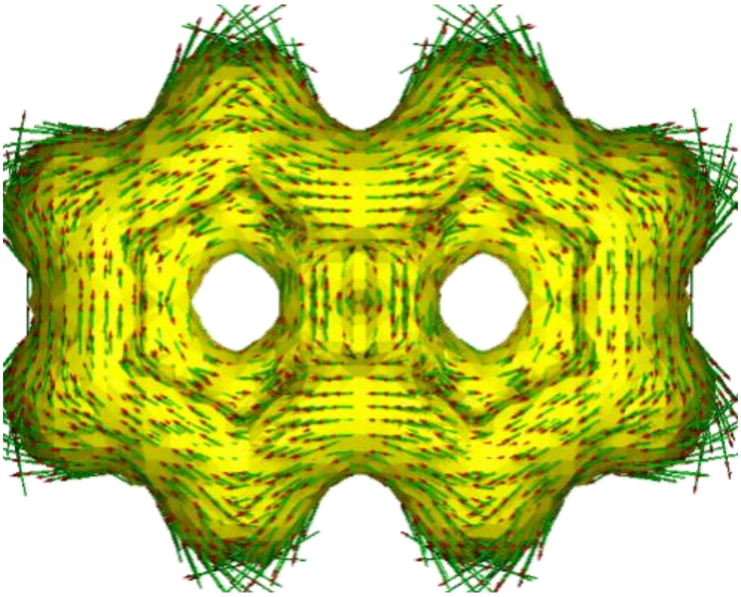

Naphthalene dication (**4**$^{2+}$)

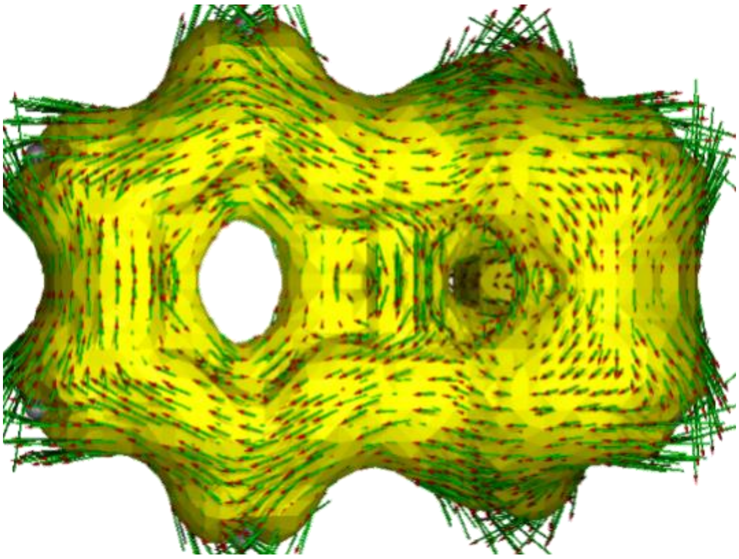

Distorted naphthalene dication (**5**$^{2+}$, 60 degrees)

**Figure S13**. ACID (isosurface: 0.025) plots of **4**$^{2+}$ and **5**$^{2+}$ in the $T_1$ state at the B3LYP/6-311G(d,p) level.

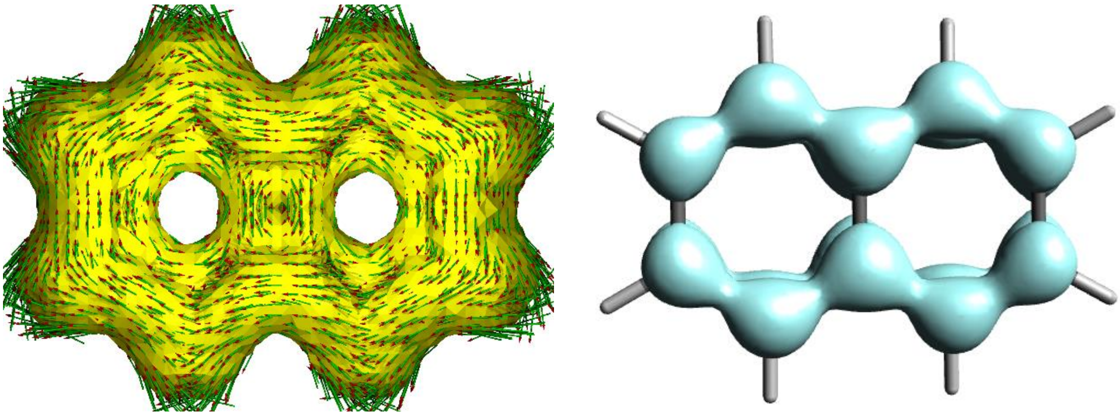

Naphthalene dication (15º degrees)

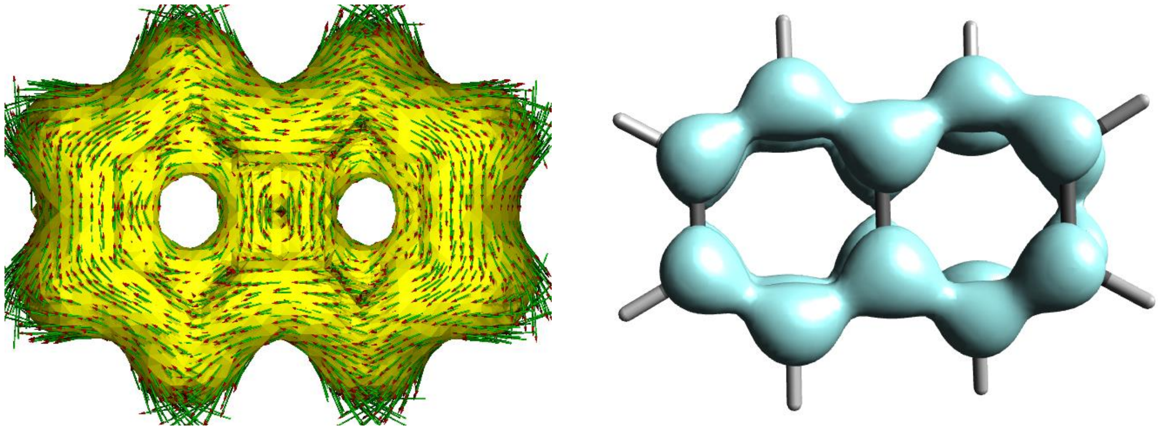

Naphthalene dication (30º degrees)

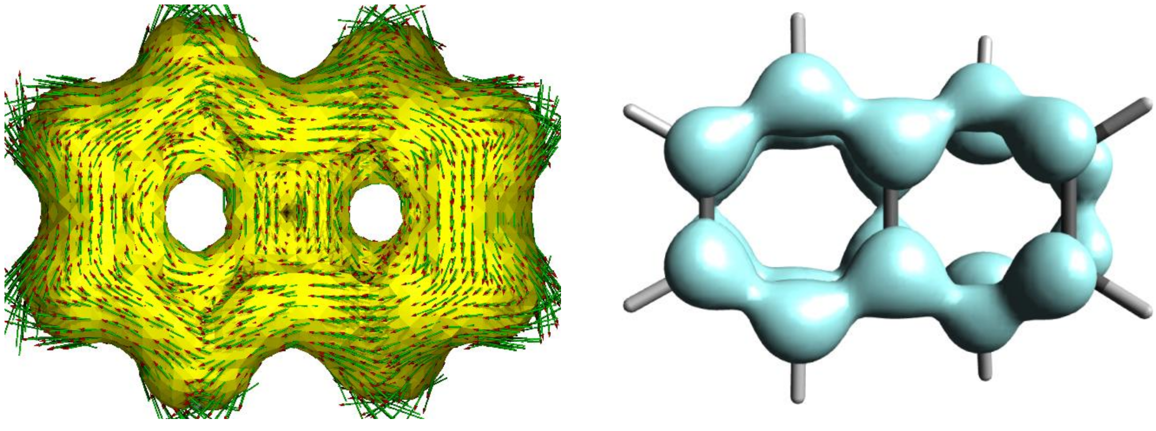

Naphthalene dication (45º degrees)

**Figure S14**. ACID (isosurface: 0.025) and EDDB (isosurface: 0.015) plots of distorted naphthalenes dication in the $T_1$ state at the B3LYP/6-311G(d,p) level.

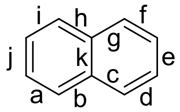


**Table S2**. Current strengths of the distorted naphthalenes in the $T_1$ state obtained from GIMIC ($nAT^{-1}$).

| *$T_1$* | *0º* | *15º* | *30º* | *45º* | *60º* |
|---|---|---|---|---|---|
| *a* | 0.0 | 0.0 | 0.1 | 0.4 | 0.9 |
| *b* | 1.2 | 1.2 | 1.2 | 1.3 | 1.5 |
| *c* | 1.2 | 1.2 | 1.2 | 1.2 | 1.3 |
| *d* | 0.0 | 0.1 | 0.4 | 0.6 | 1.0 |
| *e* | 1.6 | 1.6 | 1.7 | 2.0 | 3.0 |
| *f* | 0.0 | 0.0 | -0.2 | -0.3 | 0.0 |
| *g* | 1.2 | 1.3 | 1.3 | 1.4 | 1.5 |
| *h* | 1.2 | 1.2 | 1.2 | 1.2 | 1.4 |
| *i* | 0.0 | 0.0 | 0.2 | 0.4 | 1.0 |
| *j* | 1.6 | 1.6 | 1.4 | 1.2 | 0.7 |
| *k* | 0.0 | -0.1 | -0.3 | -0.3 | 0.1 |

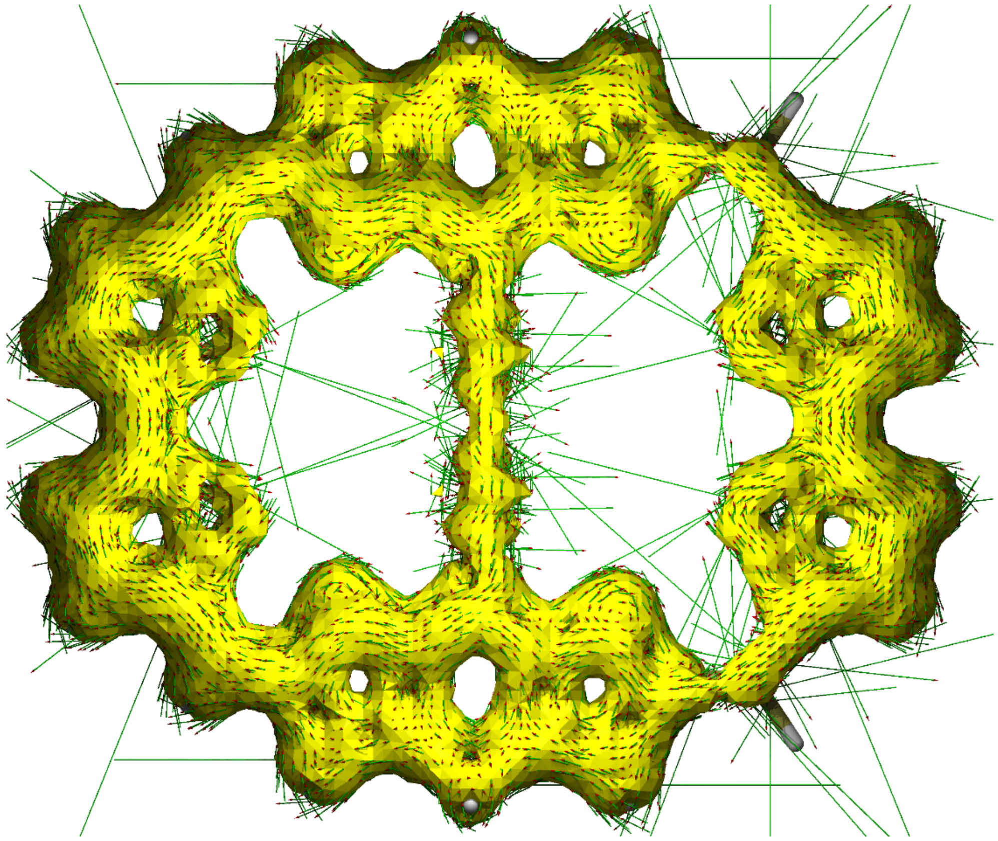

**Figure S15**. ACID plot (isosurface: 0.025) and EDDB plots (isosurface: 0.015) of $\mathbf{8^{2+}}$ in the $T_1$ state.

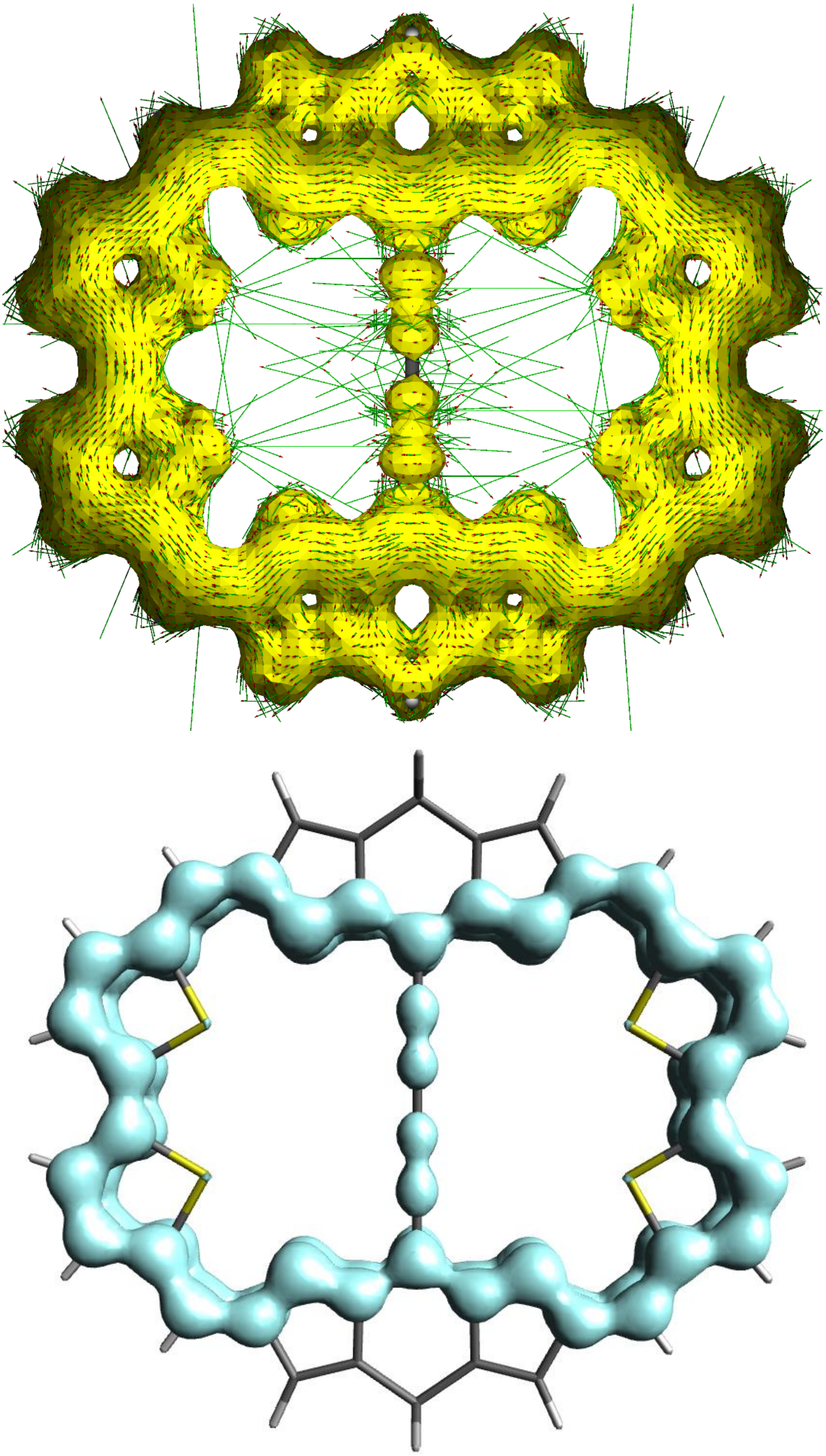

$7^{2+}$ ($T_1$)

**Figure S16.** ACID plot (isosurface: 0.025) and EDDB plot (isosurface: 0.015) of $\mathbf{7^{2+}}$ in the $T_1$ state.

**Table S3.** Percentage of π-delocalization of **2** and its derivatives at the B3LYP/6-311G(d,p) level.

| *B3LYP* | *All* | *Monocycle 1* | *Monocycle 2* | *Monocycle 3* |
|---|---|---|---|---|
| *2 ($S_0$)* | 55.3 | 35.6 | 42.5 | 41.7 |
| *2 ($C_{2v}$, $S_0$)* | 53.1 | 34.4 | 45.2 | 39.0 |
| *$2^{6+}$ ($S_0$)* | 43.2 | 26.4 | 35.4 | 30.6 |
| *7 ($S_0$)* | 50.9 | 49.2 | 31.3 | 31.3 |
| *8 ($S_0$)* | 44.9 | 44.1 | 25.7 | 34.0 |
| *$2^{2+}$ ($T_1$)* | 53.5 | 48.7 | 36.8 | 42.5 |
| *$7^{2+}$ ($T_1$)* | 46.8 | 47.0 | 30.0 | 29.9 |
| *$8^{2+}$ ($T_1$)* | 38.6 | 35.6 | 25.2 | 24.2 |

**Table S4.** Percentage of π-delocalization computed with the EDDB method of 1 and analogous cage macromolecules 9 - 13 at the B3LYP/6-311G(d,p) and CAM-B3LYP/6-311G(d,p) level.

| *B3LYP* | *All* | *Cycle 1* | *Cycle 2* | *Cycle 3* |
|---|---|---|---|---|
| *1* | 60.0 | 51.1 | 51.1 | 51.1 |
| $1^{6+}$ | 59.5 | 46.5 | 46.5 | 46.5 |
| $9^{6+}$ | 59.3 | 42.4 | 48.0 | 48.1 |
| $10^{6+}$ | 59.3 | 44.9 | 44.6 | 50.2 |
| $11^{6}$ | 60.0 | 47.5 | 47.5 | 47.5 |
| $12^{6+}$ | 56.5 | 51.6 | 40.8 | 41.8 |
| $13^{6+}$ | 55.8 | 38.0 | 54.1 | 38.0 |
| $1^{3+}$ *quartet* | 59.2 | 50.0 | 50.0 | 50.0 |
| $1^{2+}$ *triplet* | 62.0 | 51.5 | 43.4 | 43.4 |
| $9^{3+}$ *quartet* | 58.3 | 45.0 | 49.6 | 50.8 |
| $10^{3+}$ *quartet* | 58.4 | 47.8 | 47.1 | 51.7 |
| $11^{3+}$ *quartet* | 61.2 | 51.0 | 51.0 | 51.0 |

| *CAM-B3LYP* | *All* | *Cycle 1* | *Cycle 2* | *Cycle 3* |
|---|---|---|---|---|
| $1^{6+}$ | 59.1 | 46.2 | 46.2 | 46.2 |
| $9^{6+}$ | 47.8 | 30.7 | 41.5 | 39.1 |
| $10^{6+}$ | 52.0 | 36.2 | 37.1 | 47.3 |
| $11^{6+}$ | 50.5 | 42.6 | 42.6 | 42.6 |

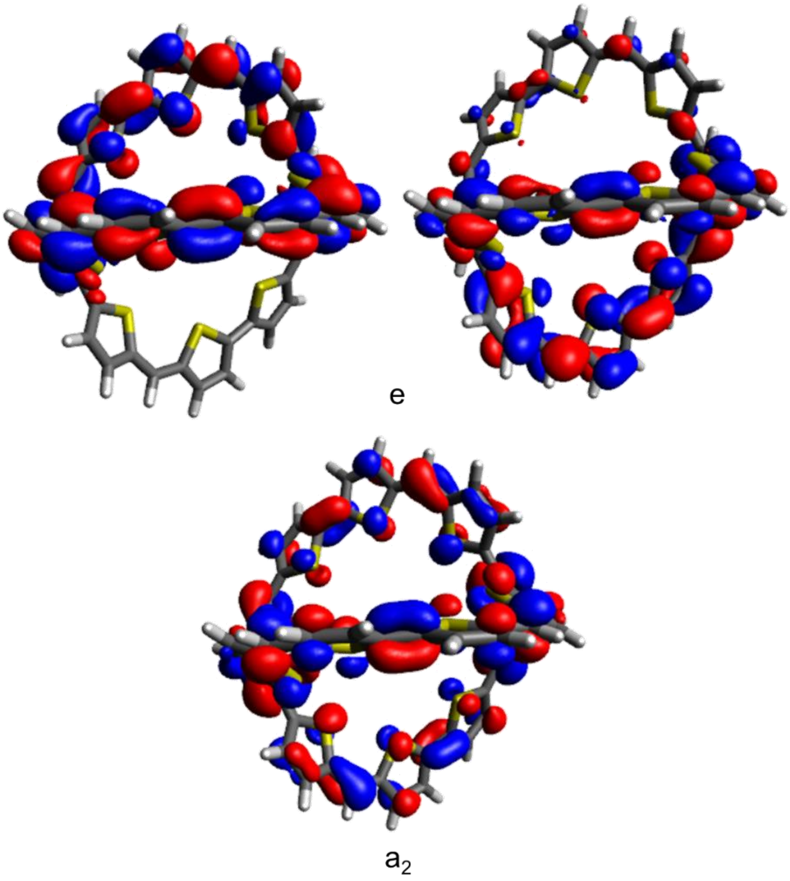


**Figure S17.** Three highest α-SOMOs of $\mathbf{1}^{3+}$ in its quartet state.

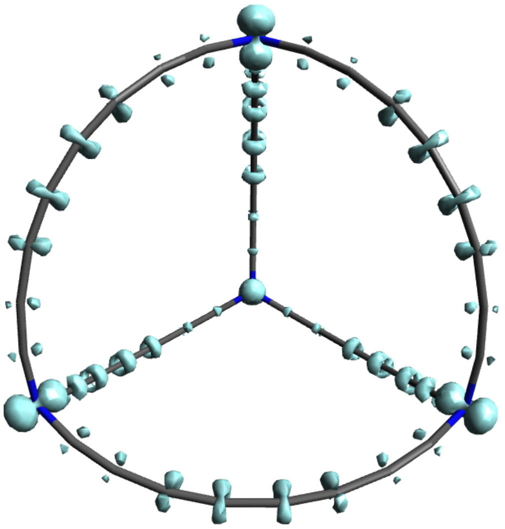

$N_4(C_8)_6$
(9.4%)

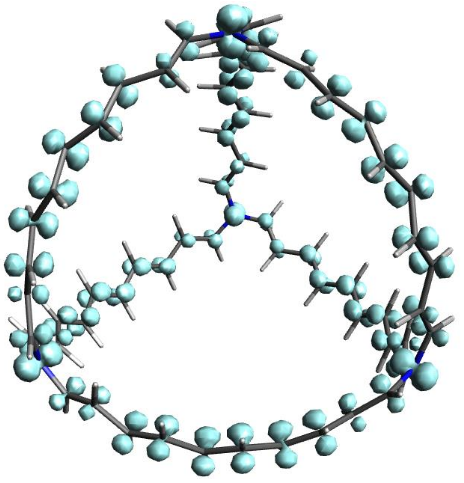

$N_4(C_{10}H_{10})_6$
(22.3%)

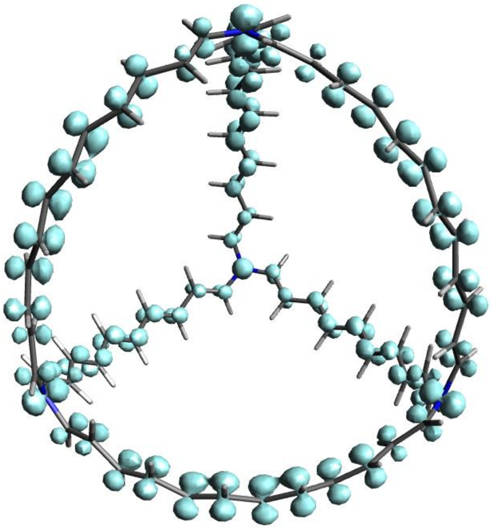

$N_4(C_{12}H_{12})_6$
(24.3%)

**Figure S18.** EDDB plots (isosurface: 0.015) of tetrahedral species considering N atoms in the corner.

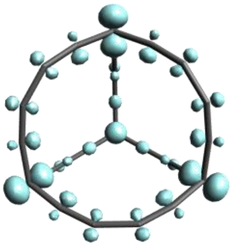

$C_4(C_4)_6^{4-}$
(14.8%)
**14**

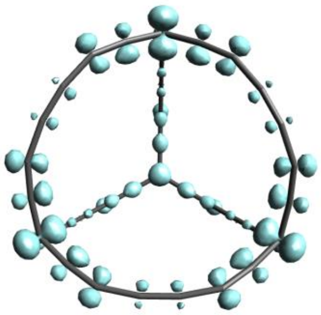

$C_4(C_6)_6^{4-}$
(17.9%)
**15**

**Figure S19.** EDDB plots (isosurface: 0.015) of **14** and **15**.

**Discussion D. Aromaticity of $Si_4^{2-}$ and $P_4$**

For $P_4$ and $Si_4^{4-}$ the global electron density of delocalized bonds function, $EDDB_G(r)$, predicts that the total population of delocalized electrons is 0.2041e and 0.2075e, respectively, which means that the effectiveness of electron delocalization in the valence-shell of both these systems approaches 1.0%. In other words, 99% of electrons in the valence shells of both species is localized, whereby they according to EDDB should be considered as nonaromatic.

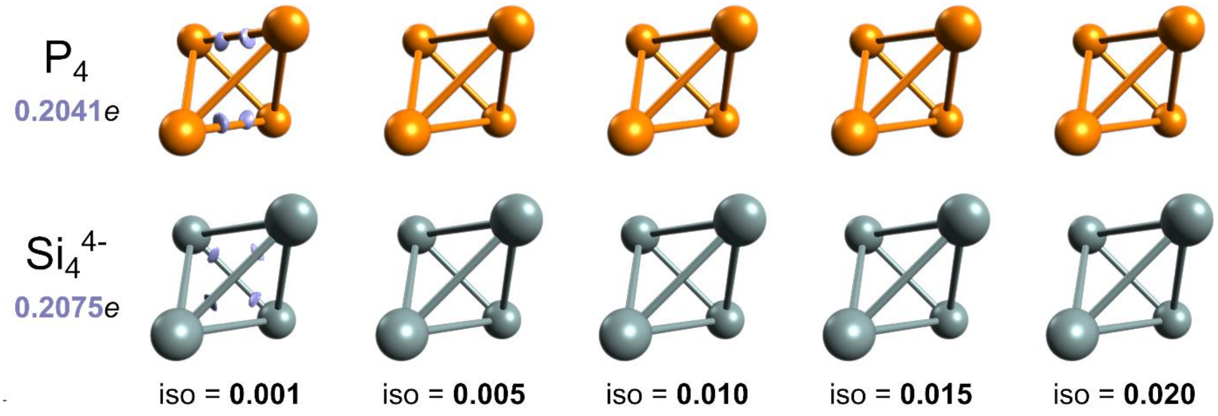


**Figure S20**: The $EDDB_G$ values (violet numbers) and contours at different isovalues for $P_4$ and $Si_4^{4-}$. The default (recommended) isovalue used in the EDDB analyses is in the range 0.010 - 0.020.

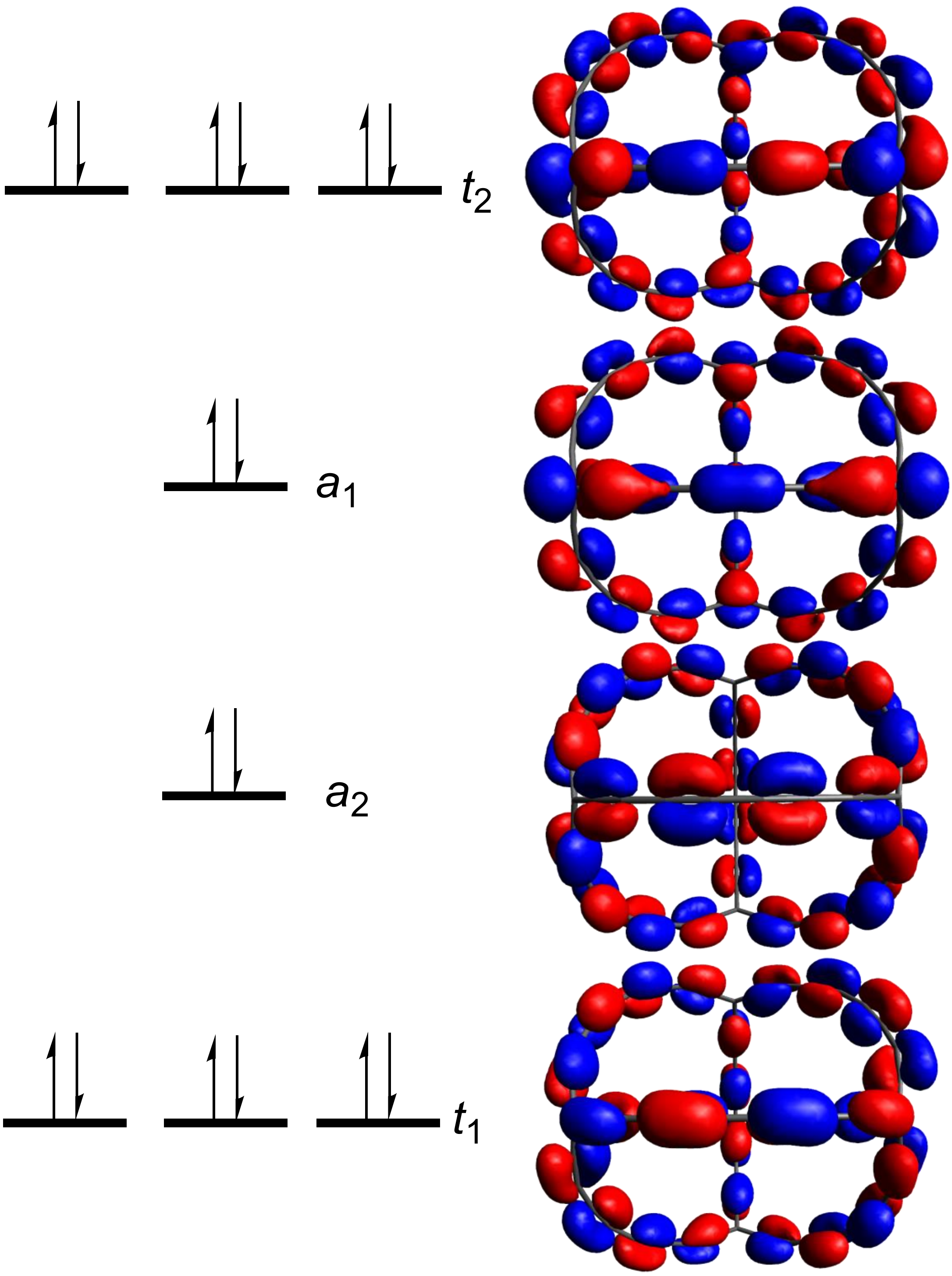


**Figure S21**. Full-image of the highest few occupied molecular orbitals (A) $C_4(C_8)_6^{4-}$ (**16**) ($T_h$ symmetric) at the optimal B3LYP/6-311G(d,p) geometries.

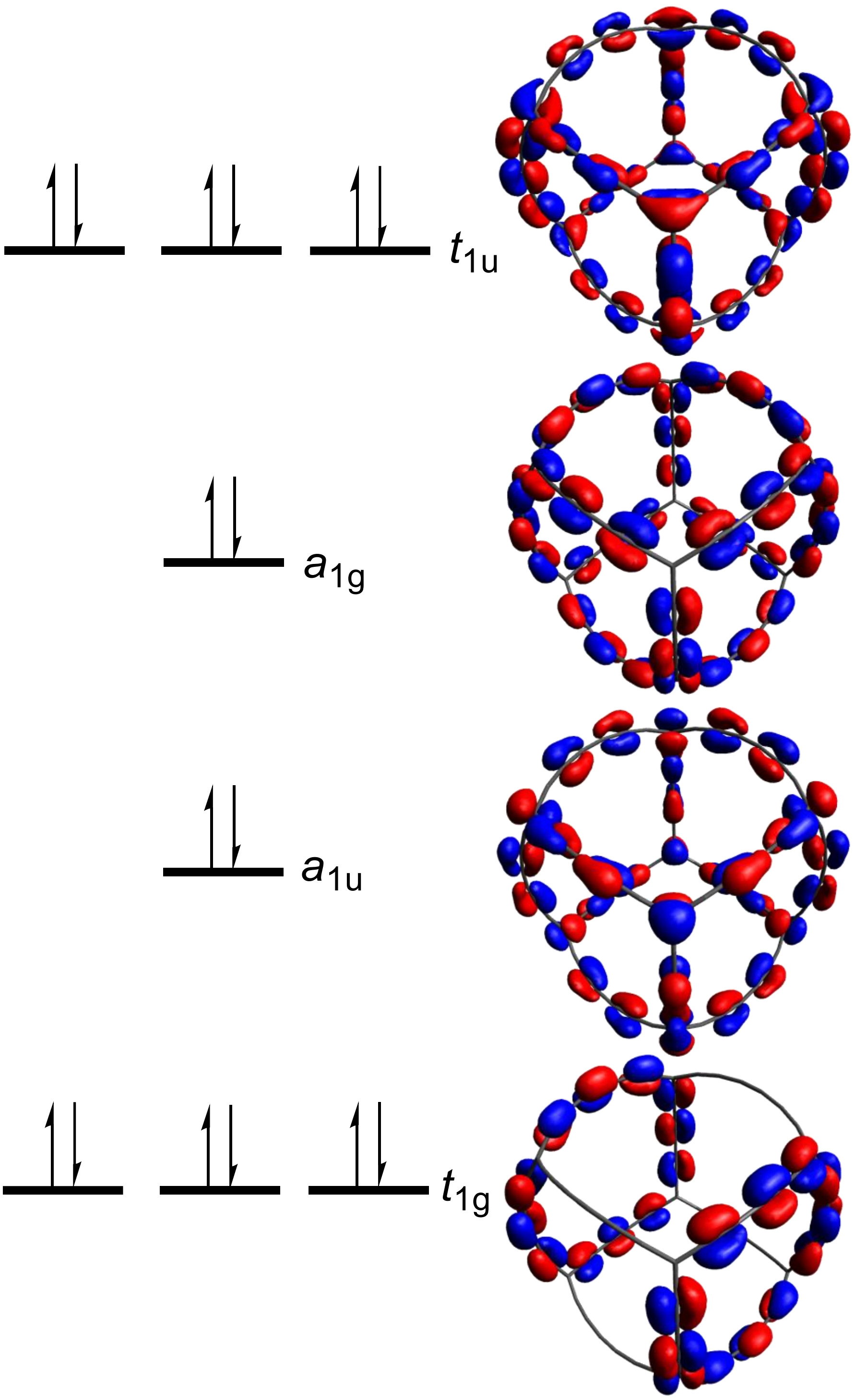


**Figure S22.** Full-image of the highest few occupied molecular orbitals of $C_8(C_6)_{12}$ (**18**) ($O_h$ symmetric) at the optimal B3LYP/6-311G(d,p) geometries.

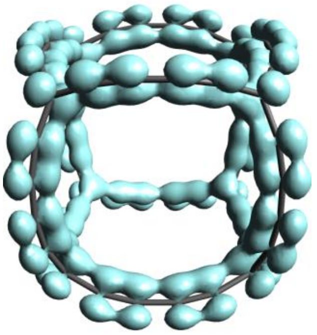

$C_8(C_4)_{12}$
(42.5%)
**17**

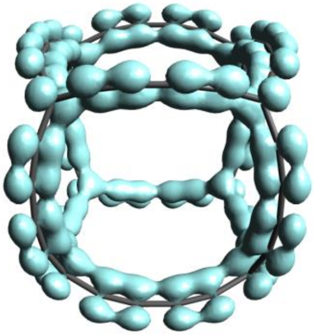

$C_8(C_4)_{12}{}^{3+}$ Q1
(43.7%)
**19**

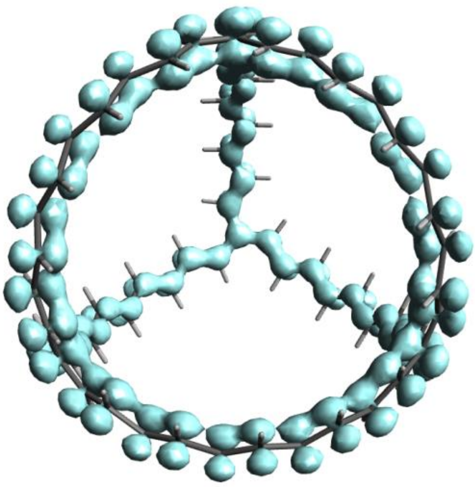

$C_4(C_{10}H_{10})_6{}^{4-}$
(41.1%)
**21**

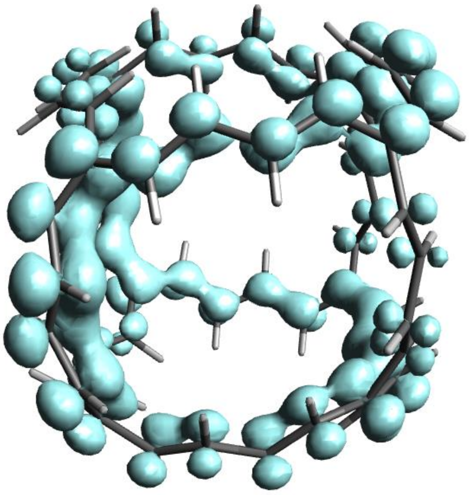

$C_8(C_4H_4)_{12}$
(39.4%)
**24**

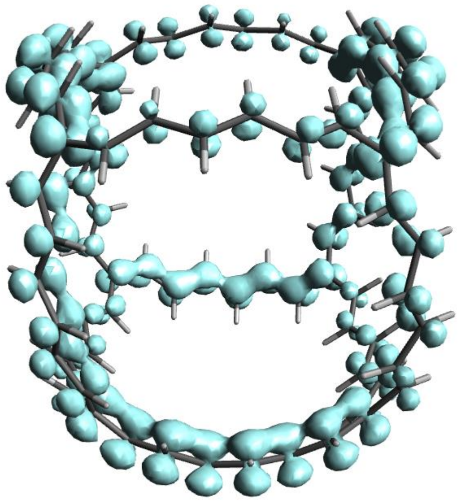

$C_8(C_6H_6)_{12}$
(35.1)
**25**

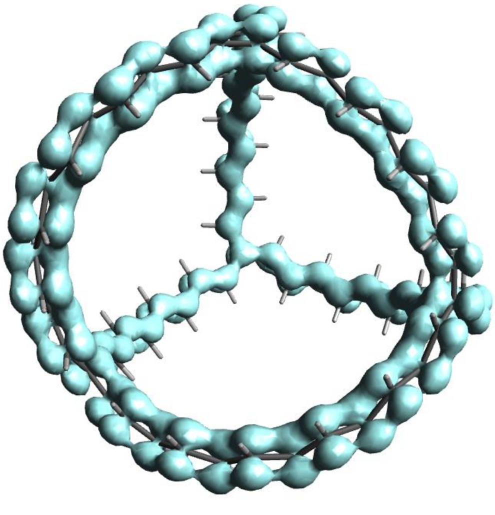

$C_4(C_{12}H_{12})_2(C_{10}H_{10})_4{}^{4-}$
(52.6%)
**26**

**Figure S23.** EDDB plots (isosurface: 0.015) of **17**, **19**, **21**, **24**, **25** and **26**. Percentage of π-delocalization in parenthesis.

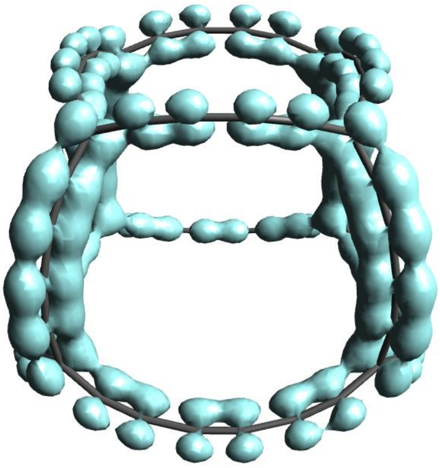

$C_8(C_4)_8(C_6)_4$
(42.2%)
**27**

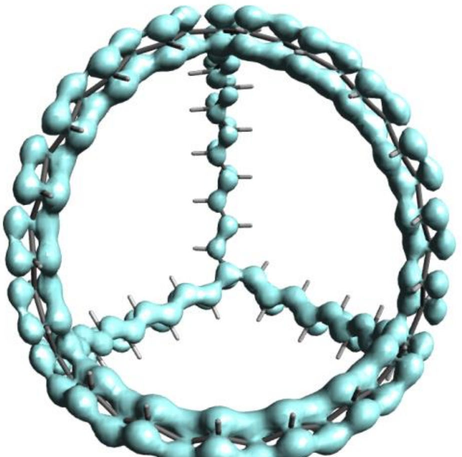

$C_4(C_{12}H_{12})_4(C_{10}H_{10})_2^-$ Q1
(52.9%)
**28**

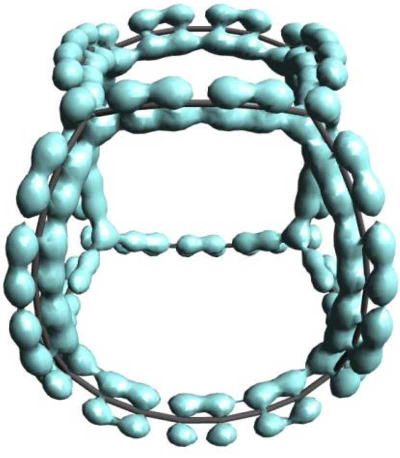

$C_8(C_4)_4(C_6)_8^{3+}$ Q1
(42.4%)
**30**

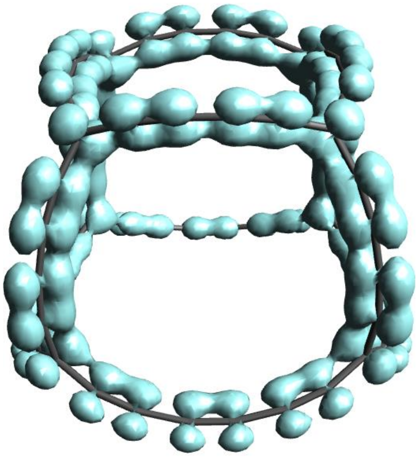

$C_8(C_6)_4(C_4)_8^{3+}$ Q1
(40.8%)
**31**

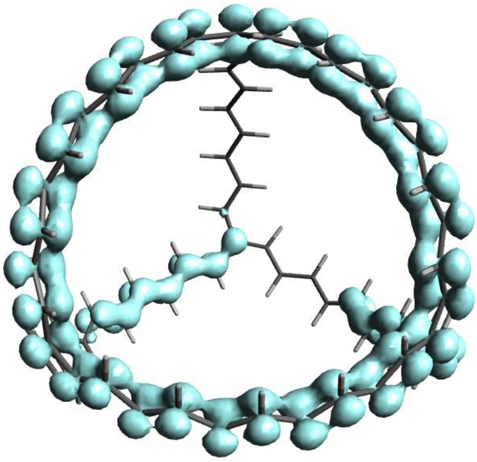

$C_4(C_{12}H_{12})_2(C_{10}H_{10})_4^-$ Q1
(49.2%)
**29**

**Figure S24.** EDDB plots (isosurface: 0.015) of **27** - **31**. Percentage of π-delocalization in parenthesis.

**Table S5.** Percentage of π-delocalization computed with the EDDB method of cage macromolecules **16**, **18**, **20**, **22**, **23** and **25** at the CAM-B3LYP/6-311G(d,p) level.

| | *B3LYP* | *CAM-B3LYP* |
|---|---|---|
| *16* | 24.4 | 15.3 |
| *18* | 41.9 | 38.5[a] |
| *20* | 42.8 | 38.8 |
| *22* | 54.9 | 35.7 |
| *23* | 52.7 | 38.8 |
| *25* | 35.1 | 18.5 |

[a] Third-order saddle point.

## Discussion E. Electron delocalization in *closo*-borane $B_{12}H_{12}^{2-}$ and its derivatives

The full (relaxed) Natural Bond Orbital (NBO) analysis of the parent *closo*-borane $B_{12}H_{12}^{2-}$ reveals 12 degenerated core orbitals with occupation number 2.000*e* and orbital energy -6.413 a.u., 12 B–H degenerated bond orbitals with occupation number 1.980*e* and orbital energy -0.070 a.u., 24 (Lewis) and 12 (non-Lewis) degenerated unhybridized 2p orbitals with the occupation numbers 0.763*e* and 0.617*e*, and orbital energies 0.342 a.u. and 0.410 a.u., respectively. This picture strongly suggests that the resonance electronic structure of *closo*-borane $B_{12}H_{12}^{2-}$ and the resulting electron delocalization is determined by the lone-pair rather than σ-bond position isomerism. The global electron density of delocalized bonds function, $EDDB_H(r)$, predicts that about half of the electron population assigned to the B atoms (excluding the electrons in core and the B–H bonds), which gives about 1*e* delocalized per each B atom (comparable o benzene). Interestingly, diagonalization of the $EDDB_H$ matrix gives 12 natural orbitals for bond delocalization (NOBD) with the occupation numbers close to 1*e*, which is typical for the lone-pair isomerism (in benzene the occupation numbers of three π-type NOBDs are close to 1.8*e*). Interestingly, the effectiveness of electron delocalization slightly increases for the carbon derivatives of $B_{12}H_{12}^{2-}$ but their resonance electronic structure remain pretty much the same.

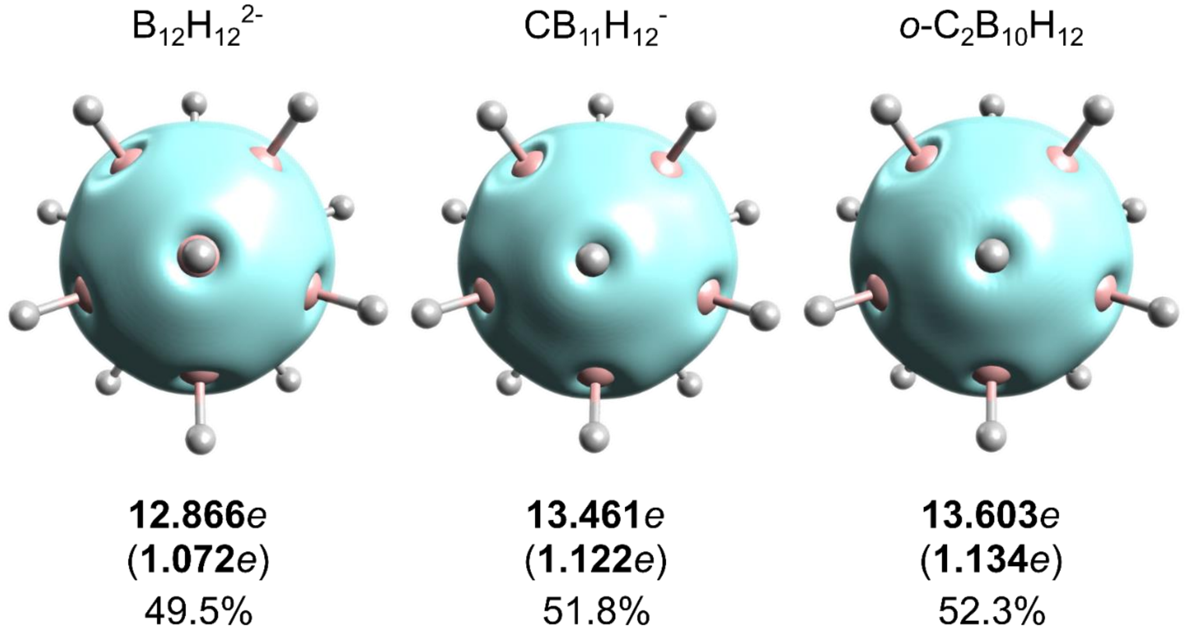


**Figure S25.** EDDB plots of *closo*-borane $B_{12}H_{12}^{2-}$, $CB_{11}H_{12}^{-}$ and *o*-$C_2B_{10}H_{12}^{2-}$. Percentage of π-delocalization.

## Description of EDDB

Electron density of delocalized bonds is a part of the original method of one-electron density (ED) decomposition into 'layers' representing different levels of electron delocalization.[2] In the basis of natural atomic orbitals (NAO), or any other representation of well-localized orthonormalized atomic orbitals, the spinless *global* electron density of delocalized bonds function, $\mathrm{EDDB_G}(r)$, for a single-determinant molecular wavefunction is defined as follows:[3,4]

$$\mathrm{EDDB_G}(r) = \sum_{\mu,\nu} \chi_\mu^\dagger(r) \mathcal{D}_{\mu,\nu}^{\Omega_\mathrm{G}} \chi_\nu(r), \tag{1}$$

where

$$\boldsymbol{\mathcal{D}}^{\Omega_\mathrm{G}} = 2 \sum_{\sigma=\alpha,\beta} \mathbf{P}^\sigma \left[ \sum_{a,b}^{\Omega_\mathrm{G}} \boldsymbol{C}_{a,b}^\sigma \boldsymbol{\varepsilon}_{a,b}^{\Omega_\mathrm{G},\sigma} \left(\boldsymbol{\lambda}_{a,b}^\sigma\right)^2 \boldsymbol{C}_{a,b}^{\sigma\,\dagger} \right] \mathbf{P}^\sigma. \tag{2}$$

In the above equation, $\mathrm{P}^\sigma$ ($\sigma = \alpha,\beta$) stands for the σ spin-resolved charge and bond-order (CBO) matrix, $\boldsymbol{C}_{a,b}^\sigma$ is the matrix of linear coefficients of the appropriately orthogonalized σ spin-resolved two-center bond-order orbitals (2cBO) of the chemical bond $X_a$–$X_b$ (obtained by diagonalization of the appropriate off-diagonal blocks of the CBO matrix), $\boldsymbol{\lambda}_{a,b}^\sigma$ represents the diagonal matrix collecting the corresponding 2cBO eigenvalues (occupation numbers), $\boldsymbol{\varepsilon}_{a,b}^{\Omega_\mathrm{G},\sigma}$ is a diagonal matrix of the σ-spin bond-conjugation factors, and for an n-atomic molecular system, $\Omega_\mathrm{G}$ represents the set of all $n(n\text{-}1)/2$ possible atomic pairs (regardless of whether the atoms are formally bonded or not). The definition of the key matrix $\boldsymbol{\varepsilon}_{a,b}^{\Omega_\mathrm{G},\sigma}$ is based on the bond-orbital projection (BOP) criterion developed by one of the authors, which relies on sophisticated orbital projection cascades involving 2cBOs, their 3-center counterparts (3cBO), and canonical MOs.[5,6] According to BOP, for a typical well-localized (Lewis-like) bond $X_a$–$X_b$, all diagonal elements of the $\boldsymbol{\varepsilon}_{a,b}^{\Omega_\mathrm{G},\sigma}$ matrix are close to zero, which means that the 2cBOs associated with this bond do not form effectively linear combinations with 2cBOs of all other bonds in a molecule. On the other hand, when the $X_a$–$X_b$ bond is effectively conjugated with any other adjacent bond in the system, the $\boldsymbol{\varepsilon}_{a,b}^{\Omega_\mathrm{G},\sigma}$ matrix has at least one element on its diagonal that approaches 1 (for systems with double and higher multifaceted aromaticity, the number of non-zero diagonal elements is equal to the number of delocalization 'channels').

The trace of such defined $\boldsymbol{\mathcal{D}}^{\Omega_\mathrm{G}}$ matrix can be straightforwardly interpreted as the population of electrons delocalized through the system of all conjugated bonds in a molecule, and as such, it can be used as a 'measure' of *global* aromaticity.[3,4] However, one of the most distinctive features of the BOP technique is that one can easily restrict the set bonds/atomic pairs in $\Omega_\mathrm{G}$ giving rise to a series of different variants of *global* and *local* EDDB functions. In the case of the $\mathrm{EDDB_H}(r)$ function, $\Omega_\mathrm{H}$ contains all possible atomic pairs in the molecule excluding hydrogen atoms from consideration. Thus, $\mathrm{EDDB_H}(r)$ is also a *global* function that can be especially useful in the analysis of global π-aromaticity in organic molecules since the 2cBO involving hydrogen atoms tend to conjugate with the adjacent σ-bond orbitals noticeably increasing the delocalization in the σ-subsystem, which may sometimes lead to less precise conclusions. In the case of the $\mathrm{EDDB_F}(r)$ function, $\Omega_\mathrm{F}$ contains all possible atomic pairs in the selected molecular fragment (usually cyclic unit without H atoms), which in the case of a single 6-membered ring (6MR) gives rise to $6 \cdot 5 / 2 = 15$ atomic pairs (the same number of linear

combinations of 2cBO has to be considered within the BOP procedure). In turn, six chemical bonds are considered within the definition of the congeneric *local* $EDDB_P(r)$ function, which 'measures' electron delocalization along the selected *pathway* of adjacent bonds. Thus, in contrast to $EDDB_F(r)$, the $EDDB_P(r)$ function does not take into account the cross-ring delocalization effects, which for 6MR are associated with the resonance involving Dewar structures. The $EDDB_P(r)$ function and the corresponding electron population (denoted simply by $EDDB_P$) can be used to visualize and quantify *local* aromaticity in a wide range of molecular rings regardless of their size and topology, but also to assess *macrocyclic* aromaticity associated with particular delocalization pathways in larger structures like expanded porphyrins. Each of the EDDB($r$) functions can easily be dissected into σ-, π- and higher-symmetry components by diagonalization of the corresponding density matrix defined in Eq. 2. The resulting eigenfunctions, called the natural orbitals for bond delocalization (NOBD), can be particularly useful in the identification of non-planar multifaceted aromatic compounds because they do not tend toward the mixing of different symmetry types like the canonical MOs.

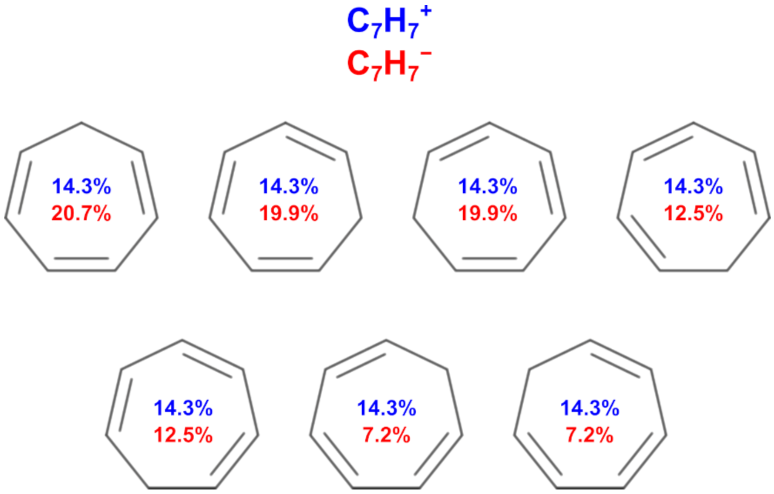


**Figure S26**. The renormalized percentage contributions of the π-resonance forms in the aromatic $C_7H_7^+$ (blue numbers) and formally antiaromatic $C_7H_7^-$ obtained from the Natural Resonance Theory (NRT[7]) module implemented in the NBO7 software. Method: B3LYP/6-311G(d,p).

## References


(1) Jemmis, E. D.; Schleyer, P. von R. Aromaticity in Three Dimensions. 4. Influence of Orbital Compatibility on the Geometry and Stability of Capped Annulene Rings with Six Interstitial Electrons. *J. Am. Chem. Soc.* **1982**, *104*, 4781–4788.

(2) Szczepanik, D. W. A New Perspective on Quantifying Electron Localization and Delocalization in Molecular Systems. *Comput. Theor. Chem.* **2016**, *1080*, 33–37.

(3) Szczepanik, D. W.; Andrzejak, M.; Dyduch, K.; Żak, E.; Makowski, M.; Mazur, G.; Mrozek, J. A Uniform Approach to the Description of Multicenter Bonding. *Phys. Chem. Chem. Phys.* **2014**, *16* (38), 20514–20523.

(4) Szczepanik, D. W.; Solà, M. The Electron Density of Delocalized Bonds (EDDBs) as a Measure of Local and Global Aromaticity. In *Aromaticity*; Elsevier, 2021; pp 259–284.

(5) Szczepanik, D. W.; Żak, E.; Dyduch, K.; Mrozek, J. Electron Delocalization Index Based on Bond Order Orbitals. *Chem. Phys. Lett.* **2014**, *593*, 154–159.

(6) Szczepanik, D. W. On the Three-Center Orbital Projection Formalism within the Electron Density of Delocalized Bonds Method. *Comput. Theor. Chem.* **2017**, *1100*, 13–17.

(7) Glendening, E. D.; Landis, C. R.; Weinhold, F. Resonance Theory Reboot. *J. Am. Chem. Soc.* **2019**, *141*, 4156–4166..

**XYZ coordinates**

Naphthalene ($C_{10}H_{10}$)

State = S0
Symmetry = $D_{2h}$
E = -385.984908818 a.u.

| | | | |
|---|---|---|---|
| C | 2.429787 | 0.707363 | 0.000014 |
| C | 1.243662 | 1.399995 | 0.000002 |
| C | -0.000000 | 0.715662 | -0.000003 |
| C | 0.000000 | -0.715662 | 0.000004 |
| C | 1.243662 | -1.399995 | 0.000016 |
| C | 2.429787 | -0.707363 | 0.000021 |
| H | -1.242235 | 2.485222 | -0.000021 |
| H | 3.372172 | 1.243570 | 0.000018 |
| H | 1.242235 | 2.485222 | -0.000003 |
| C | -1.243662 | 1.399995 | -0.000016 |
| C | -1.243662 | -1.399995 | -0.000002 |
| H | 1.242235 | -2.485222 | 0.000021 |
| H | 3.372172 | -1.243570 | 0.000030 |
| C | -2.429787 | -0.707363 | -0.000014 |
| C | -2.429787 | 0.707363 | -0.000021 |
| H | -1.242235 | -2.485222 | 0.000004 |
| H | -3.372172 | -1.243569 | -0.000018 |
| H | -3.372172 | 1.243570 | -0.000031 |

Dihedral angle = 15 degrees
State = S0
Symmetry = *C*s
E = -385.977882961 a.u.

| | | | |
|---|---|---|---|
| C | -0.050225 | 2.425385 | 0.706375 |
| C | 0.007047 | 1.243769 | 1.409586 |
| C | 0.034760 | -0.000383 | 0.737904 |
| C | 0.034760 | -0.000383 | -0.737904 |
| C | 0.007047 | 1.243769 | -1.409586 |
| C | -0.050225 | 2.425385 | -0.706375 |
| H | 0.196391 | -1.262102 | 2.482122 |
| H | -0.089148 | 3.369214 | 1.238674 |
| H | 0.017843 | 1.253130 | 2.494896 |
| C | 0.122002 | -1.249974 | 1.399008 |
| C | 0.122002 | -1.249974 | -1.399008 |
| H | 0.017843 | 1.253130 | -2.494896 |
| H | -0.089148 | 3.369214 | -1.238674 |
| C | -0.104637 | -2.418829 | -0.708447 |
| C | -0.104637 | -2.418829 | 0.708447 |
| H | 0.196391 | -1.262102 | -2.482122 |
| H | -0.178760 | -3.360057 | -1.241468 |
| H | -0.178760 | -3.360057 | 1.241468 |

Dihedral angle = 30 degrees
State = S0
Symmetry = *C*s
E = -385.959701409 a.u.

| | | | |
|---|---|---|---|
| C | 2.415035 | 0.705648 | -0.101978 |
| C | 1.234438 | 1.403504 | 0.015546 |
| C | -0.000918 | 0.715762 | 0.074962 |
| C | -0.000708 | -0.715894 | 0.074966 |
| C | 1.231472 | -1.402900 | 0.015545 |
| C | 2.413580 | -0.707226 | -0.101964 |
| H | -1.273478 | 2.436713 | 0.396291 |
| H | 3.355429 | 1.239336 | -0.182180 |
| H | 1.236605 | 2.488239 | 0.037703 |
| C | -1.259727 | 1.360950 | 0.245237 |
| C | -1.260156 | -1.360431 | 0.245266 |
| H | 1.233019 | -2.487702 | 0.037833 |
| H | 3.352877 | -1.242809 | -0.182145 |
| C | -2.386400 | -0.715652 | -0.215346 |
| C | -2.386220 | 0.716461 | -0.215358 |
| H | -1.274329 | -2.436190 | 0.396404 |
| H | -3.316427 | -1.252693 | -0.362521 |
| H | -3.316073 | 1.253773 | -0.362640 |

Dihedral angle = 45 degrees
State = S0
Symmetry = *C*s
E = -385.919736389 a.u.

| | | | |
|---|---|---|---|
| C | 2.397307 | 0.704483 | -0.146333 |
| C | 1.224619 | 1.416134 | 0.025479 |
| C | -0.003861 | 0.737904 | 0.107557 |
| C | -0.003861 | -0.737904 | 0.107557 |
| C | 1.224619 | -1.416134 | 0.025479 |
| C | 2.397307 | -0.704483 | -0.146333 |
| H | -1.331440 | 2.372159 | 0.607073 |
| H | 3.335354 | 1.235240 | -0.265097 |
| H | 1.240390 | 2.500558 | 0.059712 |
| C | -1.285429 | 1.311806 | 0.365565 |
| C | -1.285429 | -1.311806 | 0.365565 |
| H | 1.240389 | -2.500558 | 0.059711 |
| H | 3.335354 | -1.235241 | -0.265098 |
| C | -2.331371 | -0.731760 | -0.329797 |
| C | -2.331370 | 0.731760 | -0.329798 |
| H | -1.331440 | -2.372159 | 0.607074 |
| H | -3.251896 | -1.265168 | -0.536512 |
| H | -3.251895 | 1.265169 | -0.536514 |

Dihedral angle = 60 degrees
State = S0
Symmetry = *C*s
E = -385.849437684 a.u.

| | | | |
|---|---|---|---|
| C | -0.187274 | 2.374562 | 0.700343 |
| C | 0.038215 | 1.205126 | 1.422995 |
| C | 0.157949 | -0.001294 | 0.737904 |
| C | 0.157949 | -0.001294 | -0.737904 |
| C | 0.038215 | 1.205126 | -1.422995 |
| C | -0.187274 | 2.374562 | -0.700343 |
| H | 0.840240 | -1.411355 | 2.253994 |
| H | -0.346268 | 3.306765 | 1.230900 |
| H | 0.082728 | 1.227779 | 2.506719 |
| C | 0.469825 | -1.328216 | 1.230226 |
| C | 0.469825 | -1.328216 | -1.230226 |

| | | | |
|---|---|---|---|
| H | 0.082728 | 1.227779 | -2.506719 |
| H | -0.346268 | 3.306765 | -1.230900 |
| C | -0.461621 | -2.240424 | -0.789257 |
| C | -0.461621 | -2.240424 | 0.789257 |
| H | 0.840240 | -1.411355 | -2.253994 |
| H | -0.679262 | -3.181718 | -1.281880 |
| H | -0.679262 | -3.181718 | 1.281880 |

Naphthalene dication ($C_{10}H_{10}^{2+}$)

State = T1
Symmetry = $D_{2h}$
E = -385.193483400 a.u.

| | | | |
|---|---|---|---|
| C | 2.437340 | -0.716295 | -0.000014 |
| C | 1.231458 | -1.428766 | 0.000001 |
| C | 0.000001 | -0.737904 | 0.000007 |
| C | 0.000001 | 0.737904 | -0.000003 |
| C | 1.231458 | 1.428766 | -0.000016 |
| C | 2.437340 | 0.716295 | -0.000026 |
| H | -1.244561 | -2.514045 | 0.000038 |
| H | 3.383986 | -1.247925 | -0.000016 |
| H | 1.244568 | -2.514045 | 0.000010 |
| C | -1.231462 | -1.428766 | 0.000022 |
| C | -1.231462 | 1.428766 | 0.000001 |
| H | 1.244568 | 2.514045 | -0.000018 |
| H | 3.383986 | 1.247925 | -0.000044 |
| C | -2.437338 | 0.716296 | 0.000017 |
| C | -2.437338 | -0.716296 | 0.000010 |
| H | -1.244561 | 2.514045 | -0.000006 |
| H | -3.383985 | 1.247925 | 0.000046 |
| H | -3.383986 | -1.247924 | -0.000009 |

Dihedral angle = 15 degrees
State = T1
Symmetry = *C*s
E = -385.189516883 a.u.

| | | | |
|---|---|---|---|
| C | 2.431271 | 0.716295 | -0.066935 |
| C | 1.228667 | 1.429629 | 0.014693 |
| C | -0.001790 | 0.737904 | 0.050322 |
| C | -0.001790 | -0.737904 | 0.050323 |
| C | 1.228667 | -1.429629 | 0.014694 |
| C | 2.431271 | -0.716295 | -0.066933 |
| H | -1.255917 | 2.499971 | 0.232697 |
| H | 3.376565 | 1.248132 | -0.115303 |
| H | 1.243135 | 2.514531 | 0.041691 |
| C | -1.235848 | 1.419648 | 0.128543 |
| C | -1.235848 | -1.419648 | 0.128543 |
| H | 1.243135 | -2.514531 | 0.041693 |
| H | 3.376565 | -1.248132 | -0.115297 |
| C | -2.423023 | -0.716804 | -0.113069 |
| C | -2.423023 | 0.716803 | -0.113066 |
| H | -1.255917 | -2.499971 | 0.232697 |
| H | -3.359441 | -1.251652 | -0.240440 |
| H | -3.359442 | 1.251652 | -0.240430 |

Dihedral angle = 30 degrees
State = T1
Symmetry = *C*s
E = -385.176241684 a.u.

| | | | |
|---|---|---|---|
| C | 2.412358 | 0.716295 | -0.129862 |
| C | 1.219820 | 1.433518 | 0.028912 |
| C | -0.006981 | 0.737904 | 0.098610 |
| C | -0.006981 | -0.737904 | 0.098610 |
| C | 1.219820 | -1.433518 | 0.028913 |
| C | 2.412358 | -0.716295 | -0.129861 |
| H | -1.293748 | 2.452146 | 0.473598 |
| H | 3.355475 | 1.246013 | -0.222838 |
| H | 1.238114 | 2.517248 | 0.081325 |
| C | -1.249947 | 1.388828 | 0.257119 |
| C | -1.249947 | -1.388828 | 0.257119 |
| H | 1.238113 | -2.517248 | 0.081325 |
| H | 3.355475 | -1.246013 | -0.222835 |
| C | -2.377895 | -0.716386 | -0.230022 |
| C | -2.377895 | 0.716386 | -0.230021 |
| H | -1.293748 | -2.452146 | 0.473598 |
| H | -3.283967 | -1.260898 | -0.480646 |
| H | -3.283969 | 1.260898 | -0.480642 |

Dihedral angle = 45 degrees
State = T1
Symmetry = *C*s
E = -385.148611509 a.u.

| | | | |
|---|---|---|---|
| C | -0.181109 | 2.382113 | 0.716295 |
| C | 0.041643 | 1.204317 | 1.440826 |
| C | 0.140283 | -0.016317 | 0.737904 |
| C | 0.140283 | -0.016317 | -0.737904 |
| C | 0.041643 | 1.204317 | -1.440826 |
| C | -0.181109 | 2.382113 | -0.716295 |
| H | 0.728162 | -1.365200 | 2.354879 |
| H | -0.312247 | 3.322515 | 1.242644 |
| H | 0.113167 | 1.227427 | 2.522982 |
| C | 0.383262 | -1.275941 | 1.327905 |
| C | 0.383262 | -1.275941 | -1.327905 |
| H | 0.113167 | 1.227427 | -2.522982 |
| H | -0.312247 | 3.322515 | -1.242644 |
| C | -0.352940 | -2.298817 | -0.716774 |
| C | -0.352940 | -2.298817 | 0.716774 |
| H | 0.728162 | -1.365200 | -2.354879 |
| H | -0.715913 | -3.156867 | -1.277160 |
| H | -0.715913 | -3.156867 | 1.277160 |

Dihedral angle = 60 degrees
State = T1
Symmetry = *C*s
E = -385.093310373 a.u.

| | | | |
|---|---|---|---|
| C | -2.345692 | -0.716295 | -0.208715 |
| C | -1.184173 | -1.454568 | 0.051271 |
| C | 0.027014 | -0.737903 | 0.166136 |
| C | 0.027014 | 0.737903 | 0.166136 |
| C | -1.184173 | 1.454568 | 0.051271 |

| | | | |
|---|---|---|---|
| C | -2.345692 | 0.716295 | -0.208715 |
| H | 1.483696 | -2.161883 | 1.002269 |
| H | -3.285073 | -1.236980 | -0.366734 |
| H | -1.209952 | -2.535397 | 0.129903 |
| C | 1.315370 | -1.212361 | 0.496016 |
| C | 1.315370 | 1.212361 | 0.496016 |
| H | -1.209952 | 2.535397 | 0.129903 |
| H | -3.285073 | 1.236979 | -0.366734 |
| C | 2.189928 | 0.716294 | -0.479082 |
| C | 2.189928 | -0.716294 | -0.479082 |
| H | 1.483697 | 2.161883 | 1.002269 |
| H | 2.996647 | 1.299412 | -0.919195 |
| H | 2.996647 | -1.299412 | -0.919195 |

Octaphyrin [2a removing DTP]
State = S0
Symmetry = C1
E = -3273.40293154 a.u.

| | | | |
|---|---|---|---|
| C | -3.408955 | -4.829160 | -0.381891 |
| C | -2.936766 | -6.207398 | -0.497365 |
| C | -1.592393 | -6.154718 | -0.366471 |
| C | -1.252759 | -4.739334 | -0.171973 |
| C | 1.252758 | -4.739334 | 0.171974 |
| C | 1.592392 | -6.154718 | 0.366472 |
| C | 2.936765 | -6.207398 | 0.497365 |
| C | 3.408954 | -4.829160 | 0.381891 |
| C | 4.748839 | 4.439379 | -0.435764 |
| C | -4.748839 | -4.439380 | -0.435763 |
| C | 3.408955 | 4.829159 | -0.381891 |
| C | 2.936767 | 6.207398 | -0.497365 |
| C | 1.592395 | 6.154719 | -0.366470 |
| C | 1.252759 | 4.739335 | -0.171974 |
| C | -5.271933 | -3.153615 | -0.324524 |
| C | -6.651577 | -2.848154 | -0.355226 |
| C | -6.937856 | -1.510395 | -0.214837 |
| H | -7.398659 | -3.621622 | -0.479742 |
| C | -5.790403 | -0.700233 | -0.075463 |
| H | 5.482950 | 5.228514 | -0.569476 |
| H | -5.482949 | -5.228515 | -0.569476 |
| C | -1.252759 | 4.739335 | 0.171973 |
| C | -1.592394 | 6.154719 | 0.366471 |
| C | -2.936766 | 6.207398 | 0.497366 |
| C | -3.408954 | 4.829160 | 0.381891 |
| C | -5.790403 | 0.700234 | 0.075464 |
| C | -6.937856 | 1.510395 | 0.214839 |
| C | -6.651576 | 2.848154 | 0.355227 |
| C | -5.271932 | 3.153616 | 0.324524 |
| H | -7.398658 | 3.621622 | 0.479745 |
| C | -4.748838 | 4.439380 | 0.435765 |
| H | -5.482948 | 5.228515 | 0.569479 |
| C | -0.000000 | -4.137808 | 0.000000 |
| C | 0.000000 | 4.137810 | -0.000001 |
| C | 5.271933 | 3.153615 | -0.324523 |
| C | 6.651577 | 2.848153 | -0.355225 |
| C | 6.937856 | 1.510394 | -0.214836 |
| C | 5.790403 | 0.700232 | -0.075463 |
| H | 7.398659 | 3.621621 | -0.479741 |
| C | 6.937856 | -1.510396 | 0.214836 |
| C | 6.651576 | -2.848155 | 0.355224 |
| C | 5.271932 | -3.153616 | 0.324523 |
| C | 5.790403 | -0.700234 | 0.075463 |
| H | 7.398659 | -3.621623 | 0.479740 |
| C | 4.748838 | -4.439380 | 0.435763 |
| H | 5.482948 | -5.228516 | 0.569475 |
| N | 2.378411 | 3.976049 | -0.202370 |
| N | -2.378410 | 3.976049 | 0.202368 |
| N | 2.378410 | -3.976048 | 0.202368 |
| N | -2.378411 | -3.976048 | -0.202368 |
| S | 4.328782 | -1.681631 | 0.114306 |
| S | 4.328782 | 1.681630 | -0.114304 |
| S | -4.328783 | -1.681630 | -0.114306 |
| S | -4.328782 | 1.681631 | 0.114302 |
| H | -0.904041 | -6.979810 | -0.436801 |
| H | -3.554875 | -7.077847 | -0.667047 |
| H | 0.904039 | -6.979810 | 0.436803 |
| H | 3.554874 | -7.077847 | 0.667047 |
| H | 3.554877 | 7.077846 | -0.667047 |
| H | 0.904043 | 6.979812 | -0.436799 |
| H | -0.904041 | 6.979812 | 0.436800 |
| H | -3.554875 | 7.077847 | 0.667048 |
| H | -7.941115 | -1.108077 | -0.223009 |
| H | -7.941115 | 1.108077 | 0.223011 |
| H | 7.941115 | 1.108076 | -0.223007 |
| H | 7.941115 | -1.108078 | 0.223006 |
| H | -0.000000 | -3.053026 | -0.000000 |
| H | 0.000000 | 3.053028 | -0.000001 |

DTP
State = S0
Symmetry = $C_{2V}$
E = -1580.60560824 a.u.

| | | | |
|---|---|---|---|
| C | 0.000000 | 3.216779 | -0.215795 |
| C | 0.000000 | 0.709873 | -0.277169 |
| C | 0.000000 | 1.244575 | 1.002523 |
| C | 0.000000 | 2.667742 | 1.034733 |
| C | -0.000000 | -0.709873 | -0.277169 |
| C | -0.000000 | -1.244575 | 1.002523 |
| S | 0.000000 | -0.000000 | 2.244564 |
| S | -0.000000 | -1.976301 | -1.473358 |
| C | -0.000000 | -3.216779 | -0.215795 |
| C | -0.000000 | -2.667742 | 1.034733 |
| H | 0.000000 | 3.264080 | 1.937510 |
| H | -0.000000 | -3.264080 | 1.937510 |
| S | 0.000000 | 1.976301 | -1.473358 |
| C | 0.000000 | 4.666516 | -0.590319 |
| H | 0.882863 | 4.935816 | -1.178447 |
| H | -0.882863 | 4.935816 | -1.178447 |
| H | 0.000000 | 5.280124 | 0.312772 |
| C | -0.000000 | -4.666516 | -0.590319 |
| H | -0.882863 | -4.935816 | -1.178447 |

| | | | |
|---|---|---|---|
| H | 0.882863 | -4.935816 | -1.178447 |
| H | -0.000000 | -5.280124 | 0.312772 |

2
State = S0
Symmetry = CS
E = -4772.93799316 a.u.

| | | | |
|---|---|---|---|
| C | -0.346423 | -3.354276 | -4.993625 |
| C | -0.210366 | -2.936196 | -6.382236 |
| C | 0.152727 | -1.627782 | -6.346920 |
| C | 0.252232 | -1.267448 | -4.930235 |
| C | 0.087061 | 1.250207 | -4.846221 |
| C | -0.533548 | 1.502965 | -6.146242 |
| C | -0.824864 | 2.827490 | -6.174170 |
| C | -0.444394 | 3.359964 | -4.872579 |
| C | -0.707534 | 4.661158 | 4.456138 |
| C | -0.749058 | -4.589607 | -4.535287 |
| C | -0.444394 | 3.359964 | 4.872579 |
| C | -0.824864 | 2.827490 | 6.174170 |
| C | -0.533548 | 1.502965 | 6.146242 |
| C | 0.087061 | 1.250207 | 4.846221 |
| C | -0.910939 | -5.041842 | -3.203044 |
| C | -1.319747 | -6.337853 | -2.874100 |
| C | -1.405266 | -6.583823 | -1.510153 |
| H | -1.544300 | -7.074226 | -3.635384 |
| C | -1.064263 | -5.483503 | -0.717741 |
| H | -1.073210 | 5.335295 | 5.225977 |
| H | -0.981390 | -5.337716 | -5.288464 |
| C | 0.252232 | -1.267448 | 4.930235 |
| C | 0.152727 | -1.627782 | 6.346920 |
| C | -0.210366 | -2.936196 | 6.382236 |
| C | -0.346423 | -3.354276 | 4.993625 |
| C | -1.064263 | -5.483503 | 0.717741 |
| C | -1.405266 | -6.583823 | 1.510153 |
| C | -1.319747 | -6.337853 | 2.874100 |
| C | -0.910939 | -5.041842 | 3.203044 |
| H | -1.544300 | -7.074226 | 3.635384 |
| C | -0.749058 | -4.589607 | 4.535287 |
| H | -0.981390 | -5.337716 | 5.288464 |
| C | 0.587335 | 0.017213 | -4.322929 |
| C | 0.587335 | 0.017213 | 4.322929 |
| C | -0.646039 | 5.233788 | 3.177035 |
| C | -0.965897 | 6.574561 | 2.887066 |
| C | -0.953084 | 6.888990 | 1.541092 |
| C | -0.629370 | 5.802572 | 0.709470 |
| H | -1.215904 | 7.278838 | 3.670460 |
| C | -0.953084 | 6.888990 | -1.541092 |
| C | -0.965897 | 6.574561 | -2.887066 |
| C | -0.646039 | 5.233788 | -3.177035 |
| C | -0.629370 | 5.802572 | -0.709470 |
| H | -1.215904 | 7.278838 | -3.670460 |
| C | -0.707534 | 4.661158 | -4.456138 |
| H | -1.073210 | 5.335295 | -5.225977 |
| N | 0.118549 | 2.379217 | 4.112581 |
| N | -0.039025 | -2.311689 | 4.155284 |
| N | 0.118549 | 2.379217 | -4.112581 |
| N | -0.039025 | -2.311689 | -4.155284 |
| S | -0.300053 | 4.375266 | -1.681116 |
| S | -0.300053 | 4.375266 | 1.681116 |
| S | -0.635663 | -4.111231 | -1.732008 |
| S | -0.635663 | -4.111231 | 1.732008 |
| H | 0.389969 | -0.992972 | -7.185849 |
| H | -0.363178 | -3.557401 | -7.253432 |
| H | -0.727832 | 0.781404 | -6.921515 |
| H | -1.289991 | 3.385835 | -6.974194 |
| H | -1.289991 | 3.385835 | 6.974194 |
| H | -0.727832 | 0.781404 | 6.921515 |
| H | 0.389969 | -0.992972 | 7.185849 |
| H | -0.363178 | -3.557401 | 7.253432 |
| C | 1.261923 | -0.030380 | -3.080035 |
| C | 1.969313 | -1.142053 | -2.568902 |
| S | 1.157377 | 1.276778 | -1.884346 |
| C | 2.300754 | -0.994869 | -1.239001 |
| H | 2.139349 | -2.029686 | -3.153750 |
| C | 1.876707 | 0.238267 | -0.691566 |
| C | 1.261923 | -0.030380 | 3.080035 |
| C | 1.969313 | -1.142053 | 2.568902 |
| S | 1.157377 | 1.276778 | 1.884346 |
| C | 2.300754 | -0.994869 | 1.239001 |
| H | 2.139349 | -2.029686 | 3.153750 |
| C | 1.876707 | 0.238267 | 0.691566 |
| S | 2.849661 | -2.131748 | -0.000000 |
| H | -1.705412 | -7.536528 | -1.097887 |
| H | -1.705412 | -7.536528 | 1.097887 |
| H | -1.192199 | 7.870185 | 1.154928 |
| H | -1.192199 | 7.870185 | -1.154928 |

2 (planar)
State = S0
Symmetry = $C_{2V}$
E = -4772.79937303 a.u.

| | | | |
|---|---|---|---|
| C | 0.000000 | 5.305696 | 3.585907 |
| C | 0.000000 | 6.712711 | 3.155420 |
| C | 0.000000 | 6.704657 | 1.787309 |
| C | 0.000000 | 5.293277 | 1.351677 |
| N | 0.000000 | 4.551015 | 2.462830 |
| C | 0.000000 | 4.655518 | -0.005313 |
| C | 0.000000 | 4.717616 | 4.859751 |
| C | 0.000000 | 3.342229 | 5.318240 |
| C | 0.000000 | 4.541086 | -4.814071 |
| C | 0.000000 | 3.215615 | -5.345071 |
| C | 0.000000 | 2.938728 | -6.726719 |
| C | 0.000000 | 1.589084 | -7.070098 |
| C | 0.000000 | 0.720263 | -5.970204 |
| S | 0.000000 | 1.668190 | -4.491787 |
| C | -0.000000 | -1.589084 | -7.070098 |
| C | -0.000000 | -0.720263 | -5.970204 |
| C | -0.000000 | -2.938728 | -6.726719 |
| C | -0.000000 | -3.215615 | -5.345071 |
| S | -0.000000 | -1.668190 | -4.491787 |

| | | | |
|---|---|---|---|
| S | 0.000000 | 1.870749 | 4.345974 |
| C | 0.000000 | 0.740510 | 5.726309 |
| C | 0.000000 | 1.545196 | 6.878327 |
| C | 0.000000 | 2.930575 | 6.662491 |
| C | -0.000000 | -0.740510 | 5.726309 |
| C | -0.000000 | -1.545196 | 6.878327 |
| S | -0.000000 | -1.870749 | 4.345974 |
| C | -0.000000 | -3.342229 | 5.318240 |
| C | -0.000000 | -2.930575 | 6.662491 |
| C | -0.000000 | -4.717616 | 4.859751 |
| C | -0.000000 | -4.541086 | -4.814071 |
| C | -0.000000 | -5.305696 | 3.585907 |
| C | 0.000000 | -5.093643 | -3.530746 |
| N | -0.000000 | -4.551015 | 2.462830 |
| C | -0.000000 | -5.293277 | 1.351677 |
| C | -0.000000 | -6.704657 | 1.787309 |
| C | -0.000000 | -6.712711 | 3.155420 |
| C | 0.000000 | -6.524749 | -3.206236 |
| C | 0.000000 | -6.624226 | -1.846490 |
| C | 0.000000 | -5.256178 | -1.330496 |
| N | 0.000000 | -4.398887 | -2.363168 |
| C | 0.000000 | 3.234928 | -0.043357 |
| C | 0.000000 | 0.698177 | -0.422972 |
| C | 0.000000 | 1.160224 | 0.910697 |
| C | 0.000000 | 2.476032 | 1.110147 |
| C | -0.000000 | -0.698177 | -0.422972 |
| C | -0.000000 | -1.160224 | 0.910697 |
| S | 0.000000 | -0.000000 | 2.154610 |
| S | 0.000000 | -2.130285 | -1.469964 |
| C | -0.000000 | -3.234928 | -0.043357 |
| C | -0.000000 | -2.476032 | 1.110147 |
| C | -0.000000 | -4.655518 | -0.005313 |
| H | 0.000000 | 7.586485 | 3.792125 |
| H | 0.000000 | 5.416930 | 5.693126 |
| H | 0.000000 | 5.284392 | -5.608289 |
| H | 0.000000 | 3.735813 | -7.460031 |
| H | -0.000000 | -3.735813 | -7.460031 |
| H | 0.000000 | 3.643574 | 7.478369 |
| H | -0.000000 | -3.643574 | 7.478369 |
| H | -0.000000 | -5.416930 | 5.693126 |
| H | -0.000000 | -5.284392 | -5.608289 |
| H | -0.000000 | -7.586485 | 3.792125 |
| H | 0.000000 | -7.337334 | -3.919471 |
| H | 0.000000 | 2.931014 | 2.069239 |
| H | -0.000000 | -2.931014 | 2.069239 |
| C | 0.000000 | 5.256178 | -1.330496 |
| C | 0.000000 | 6.624226 | -1.846490 |
| N | 0.000000 | 4.398887 | -2.363168 |
| C | 0.000000 | 6.524749 | -3.206236 |
| C | 0.000000 | 5.093643 | -3.530746 |
| H | 0.000000 | 7.337334 | -3.919471 |
| S | 0.000000 | 2.130285 | -1.469964 |
| H | 0.000000 | -7.533548 | -1.264502 |
| H | 0.000000 | -7.575930 | 1.148590 |
| H | 0.000000 | 7.533548 | -1.264502 |
| H | 0.000000 | 7.575930 | 1.148590 |
| H | 0.000000 | 1.240152 | -8.094279 |
| H | -0.000000 | -1.240152 | -8.094279 |
| H | 0.000000 | -1.126619 | 7.876010 |
| H | 0.000000 | 1.126619 | 7.876010 |

$2^{6+}$

State = S0

Symmetry = $C_S$

E = -4770.24464202 a.u.

| | | | |
|---|---|---|---|
| C | -0.315055 | -3.360292 | -4.934240 |
| C | -0.187666 | -2.970465 | -6.350207 |
| C | 0.176466 | -1.674171 | -6.349484 |
| C | 0.296166 | -1.286932 | -4.920700 |
| C | 0.143146 | 1.262294 | -4.867768 |
| C | -0.302935 | 1.577226 | -6.244828 |
| C | -0.595928 | 2.890906 | -6.245119 |
| C | -0.383872 | 3.360727 | -4.858914 |
| C | -0.655177 | 4.676100 | 4.435779 |
| C | -0.743992 | -4.605150 | -4.481646 |
| C | -0.383872 | 3.360727 | 4.858914 |
| C | -0.595928 | 2.890906 | 6.245119 |
| C | -0.302935 | 1.577226 | 6.244828 |
| C | 0.143146 | 1.262294 | 4.867768 |
| C | -0.936079 | -5.065693 | -3.156208 |
| C | -1.376854 | -6.398765 | -2.872600 |
| C | -1.494513 | -6.669816 | -1.535564 |
| H | -1.589610 | -7.115454 | -3.658604 |
| C | -1.149509 | -5.554287 | -0.700264 |
| H | -0.905944 | 5.373478 | 5.232591 |
| H | -0.969256 | -5.340231 | -5.250957 |
| C | 0.296166 | -1.286932 | 4.920700 |
| C | 0.176466 | -1.674171 | 6.349484 |
| C | -0.187666 | -2.970465 | 6.350207 |
| C | -0.315055 | -3.360292 | 4.934240 |
| C | -1.149509 | -5.554287 | 0.700264 |
| C | -1.494513 | -6.669816 | 1.535564 |
| C | -1.376854 | -6.398765 | 2.872600 |
| C | -0.936079 | -5.065693 | 3.156208 |
| H | -1.589610 | -7.115454 | 3.658604 |
| C | -0.743992 | -4.605150 | 4.481646 |
| H | -0.969256 | -5.340231 | 5.250957 |
| C | 0.595994 | 0.008238 | -4.314552 |
| C | 0.595994 | 0.008238 | 4.314552 |
| C | -0.687555 | 5.235901 | 3.144696 |
| C | -0.965397 | 6.622058 | 2.898447 |
| C | -1.000910 | 6.953420 | 1.565833 |
| C | -0.763758 | 5.836255 | 0.705455 |
| H | -1.129155 | 7.330406 | 3.703946 |
| C | -1.000910 | 6.953420 | -1.565833 |
| C | -0.965397 | 6.622058 | -2.898447 |
| C | -0.687555 | 5.235901 | -3.144696 |
| C | -0.763758 | 5.836255 | -0.705455 |
| H | -1.129155 | 7.330406 | -3.703946 |
| C | -0.655177 | 4.676100 | -4.435779 |

| | | | |
|---|---|---|---|
| H | -0.905944 | 5.373478 | -5.232591 |
| N | 0.079975 | 2.348870 | 4.071499 |
| N | 0.020071 | -2.308027 | 4.102368 |
| N | 0.079975 | 2.348870 | -4.071499 |
| N | 0.020071 | -2.308027 | -4.102368 |
| S | -0.477476 | 4.370446 | -1.642766 |
| S | -0.477476 | 4.370446 | 1.642766 |
| S | -0.691272 | -4.153005 | -1.685910 |
| S | -0.691272 | -4.153005 | 1.685910 |
| H | 0.410875 | -1.067712 | -7.210229 |
| H | -0.346211 | -3.615713 | -7.204376 |
| H | -0.360488 | 0.908876 | -7.087507 |
| H | -0.938610 | 3.495304 | -7.074595 |
| H | -0.938610 | 3.495304 | 7.074595 |
| H | -0.360488 | 0.908876 | 7.087507 |
| H | 0.410875 | -1.067712 | 7.210229 |
| H | -0.346211 | -3.615713 | 7.204376 |
| C | 1.252586 | -0.021222 | -3.045619 |
| C | 2.056205 | -1.110079 | -2.564727 |
| S | 1.072542 | 1.269292 | -1.869220 |
| C | 2.366532 | -0.967993 | -1.228497 |
| H | 2.359926 | -1.931098 | -3.196152 |
| C | 1.835971 | 0.245571 | -0.684372 |
| C | 1.252586 | -0.021222 | 3.045619 |
| C | 2.056205 | -1.110079 | 2.564727 |
| S | 1.072542 | 1.269292 | 1.869220 |
| C | 2.366532 | -0.967993 | 1.228497 |
| H | 2.359926 | -1.931098 | 3.196152 |
| C | 1.835971 | 0.245571 | 0.684372 |
| S | 3.041470 | -2.042636 | 0.000000 |
| H | -1.813559 | -7.630449 | -1.150767 |
| H | -1.813559 | -7.630449 | 1.150767 |
| H | -1.193976 | 7.957058 | 1.206636 |
| H | -1.193976 | 7.957058 | -1.206636 |

7
State = S0
Symmetry = $C_1$
E = -3500.82506405 a.u.

| | | | |
|---|---|---|---|
| C | 3.299165 | -4.570160 | 0.000088 |
| C | 2.562687 | -5.830512 | 0.000217 |
| C | 1.243713 | -5.498747 | 0.000127 |
| C | 1.216311 | -4.029068 | -0.000056 |
| C | -1.216032 | -4.029113 | -0.000070 |
| C | -1.243370 | -5.498791 | 0.000171 |
| C | -2.562329 | -5.830615 | 0.000155 |
| C | -3.298864 | -4.570297 | 0.000054 |
| C | -4.672689 | 4.382830 | 0.000130 |
| C | 4.672685 | -4.382834 | 0.000104 |
| C | -3.299161 | 4.570157 | 0.000139 |
| C | -2.562682 | 5.830510 | 0.000394 |
| C | -1.243710 | 5.498742 | 0.000063 |
| C | -1.216307 | 4.029064 | -0.000033 |
| C | 5.321581 | -3.133943 | 0.000056 |
| C | 6.712015 | -2.921254 | 0.000100 |
| C | 7.083789 | -1.584477 | 0.000057 |
| H | 7.414605 | -3.745232 | 0.000148 |
| C | 5.987057 | -0.709870 | -0.000004 |
| H | -5.313764 | 5.259094 | 0.000219 |
| H | 5.313761 | -5.259097 | 0.000182 |
| C | 1.216035 | 4.029121 | -0.000106 |
| C | 1.243374 | 5.498800 | 0.000094 |
| C | 2.562335 | 5.830621 | 0.000179 |
| C | 3.298869 | 4.570303 | 0.000043 |
| C | 5.987009 | 0.710160 | -0.000014 |
| C | 7.083682 | 1.584840 | 0.000046 |
| C | 6.711820 | 2.921592 | 0.000075 |
| C | 5.321371 | 3.134191 | 0.000021 |
| H | 7.414357 | 3.745616 | 0.000122 |
| C | 4.672399 | 4.383043 | 0.000064 |
| H | 5.313429 | 5.259341 | 0.000148 |
| C | 0.000118 | -3.277214 | -0.000142 |
| C | -0.000126 | 3.277216 | -0.000188 |
| C | -5.321575 | 3.133941 | 0.000045 |
| C | -6.712019 | 2.921249 | 0.000078 |
| C | -7.083790 | 1.584480 | 0.000019 |
| C | -5.987053 | 0.709863 | -0.000036 |
| H | -7.414605 | 3.745231 | 0.000133 |
| C | -7.083685 | -1.584846 | -0.000006 |
| C | -6.711825 | -2.921590 | 0.000035 |
| C | -5.321367 | -3.134192 | 0.000009 |
| C | -5.987005 | -0.710156 | -0.000040 |
| H | -7.414357 | -3.745619 | 0.000062 |
| C | -4.672403 | -4.383041 | 0.000052 |
| H | -5.313427 | -5.259342 | 0.000086 |
| N | -2.438833 | 3.507271 | -0.000029 |
| N | 2.438572 | 3.507373 | -0.000131 |
| N | -2.438583 | -3.507373 | -0.000103 |
| N | 2.438824 | -3.507268 | -0.000042 |
| S | -4.473027 | -1.598101 | 0.000001 |
| S | -4.473133 | 1.597906 | -0.000008 |
| S | 4.473136 | -1.597910 | -0.000024 |
| S | 4.473028 | 1.598101 | -0.000052 |
| H | 3.000612 | -6.819267 | 0.000306 |
| H | -3.000197 | -6.819395 | 0.000101 |
| H | -3.000595 | 6.819271 | 0.000665 |
| H | 3.000218 | 6.819395 | 0.000230 |
| C | 0.000103 | -1.895177 | -0.000383 |
| C | 0.000034 | -0.666285 | -0.000524 |
| C | -0.000085 | 1.895176 | -0.000519 |
| C | -0.000041 | 0.666285 | -0.000484 |
| H | 8.111219 | -1.245844 | 0.000050 |
| H | 8.111135 | 1.246276 | 0.000051 |
| H | -8.111219 | 1.245844 | 0.000003 |
| H | -8.111135 | -1.246277 | -0.000033 |
| C | -0.000192 | 6.346362 | 0.000028 |
| C | 0.000182 | -6.346361 | 0.000121 |
| H | 0.000190 | -7.009154 | 0.874203 |
| H | 0.000176 | -7.009038 | -0.874054 |
| H | -0.000230 | 7.009177 | 0.874092 |

| | | | |
|---|---|---|---|
| H | -0.000206 | 7.009017 | -0.874165 |

8
State = S0
Symmetry = $C_1$
E = -4003.57671571 a.u.

| | | | |
|---|---|---|---|
| C | 2.946879 | -4.789861 | -0.000799 |
| C | 2.141706 | -5.990863 | -0.000389 |
| C | 0.846446 | -5.568802 | 0.000120 |
| C | 0.899716 | -4.108466 | -0.000130 |
| N | 2.159091 | -3.659291 | -0.000646 |
| C | -0.295752 | -3.288483 | 0.000082 |
| C | -5.019517 | -4.337774 | 0.000962 |
| C | -5.696750 | -3.112885 | 0.000860 |
| C | -7.093005 | -2.924434 | 0.000990 |
| C | -7.483496 | -1.595582 | 0.000908 |
| C | -6.396256 | -0.706693 | 0.000719 |
| S | -4.869457 | -1.570200 | 0.000564 |
| C | -7.483682 | 1.594802 | 0.000797 |
| C | -6.396333 | 0.706038 | 0.000670 |
| C | -7.093365 | 2.923700 | 0.000769 |
| C | -5.697132 | 3.112309 | 0.000616 |
| S | -4.869645 | 1.569739 | 0.000471 |
| C | -5.020027 | 4.337253 | 0.000626 |
| C | 2.946265 | 4.790717 | -0.000493 |
| C | -3.641213 | 4.501363 | 0.000507 |
| N | 2.158769 | 3.659879 | -0.000484 |
| C | 0.899277 | 4.108772 | -0.000179 |
| C | 0.845698 | 5.569099 | 0.000120 |
| C | 2.140893 | 5.991437 | -0.000200 |
| C | -2.938208 | 5.773436 | 0.000615 |
| C | -1.616179 | 5.472141 | 0.000489 |
| C | -1.542667 | 4.000758 | 0.000219 |
| N | -2.762958 | 3.453937 | 0.000280 |
| C | -0.250576 | -1.903021 | -0.000169 |
| C | -0.238362 | -0.668297 | -0.000467 |
| C | -0.238499 | 0.668452 | -0.000160 |
| C | -0.250892 | 1.903174 | -0.000276 |
| C | -0.296088 | 3.288639 | -0.000074 |
| H | 2.504780 | -7.009510 | -0.000440 |
| H | -5.635311 | -5.231980 | 0.001176 |
| H | -7.780898 | -3.760654 | 0.001165 |
| H | -7.781354 | 3.759841 | 0.000874 |
| H | -5.635904 | 5.231404 | 0.000771 |
| H | 2.503731 | 7.010169 | -0.000182 |
| H | -3.398079 | 6.752162 | 0.000800 |
| C | -1.542196 | -4.000780 | 0.000450 |
| C | -1.615471 | -5.472187 | 0.000736 |
| N | -2.762585 | -3.454179 | 0.000515 |
| C | -2.937453 | -5.773691 | 0.000965 |
| C | -3.640679 | -4.501733 | 0.000828 |
| H | -3.397163 | -6.752492 | 0.001208 |
| H | -8.514788 | -1.268707 | 0.001013 |
| H | -8.514928 | 1.267782 | 0.000927 |
| C | -0.419352 | 6.369068 | 0.000600 |
| C | -0.418510 | -6.368951 | 0.000800 |
| H | -0.446921 | 7.030750 | 0.875544 |
| H | -0.447206 | 7.031388 | -0.873846 |
| H | -0.446373 | -7.031367 | -0.873571 |
| H | -0.445902 | -7.030543 | 0.875818 |
| S | 4.756343 | 1.608523 | -0.001466 |
| S | 4.756865 | -1.608747 | -0.001081 |
| C | 5.599208 | -3.136004 | -0.001200 |
| C | 6.255214 | -0.717292 | -0.000845 |
| C | 6.254927 | 0.717768 | -0.000709 |
| C | 5.598607 | 3.136574 | -0.000761 |
| C | 6.978064 | -2.928723 | -0.000960 |
| C | 7.347215 | -1.578876 | -0.000796 |
| C | 7.346814 | 1.579526 | 0.000085 |
| C | 6.977447 | 2.929295 | 0.000075 |
| H | 8.373245 | -1.236782 | -0.000679 |
| H | 7.692316 | -3.742712 | -0.000939 |
| H | 8.372886 | 1.237563 | 0.000725 |
| H | 7.691624 | 3.743362 | 0.000674 |
| Si | 4.704381 | -4.699760 | -0.000918 |
| H | 5.538507 | -5.916100 | -0.001613 |
| Si | 4.704215 | 4.700020 | -0.000380 |
| H | 5.539187 | 5.915792 | -0.000721 |

$2^{2+}$
State = T1
Symmetry = $C_1$
E = -4772.40615518 a.u.

| | | | |
|---|---|---|---|
| C | -3.325411 | 4.984035 | -0.331127 |
| C | -2.931938 | 6.378242 | -0.162856 |
| C | -1.632975 | 6.357682 | 0.224551 |
| C | -1.258722 | 4.943220 | 0.312008 |
| N | -2.283608 | 4.150010 | -0.011635 |
| C | 0.019101 | 4.330641 | 0.653834 |
| C | -4.547916 | 4.518094 | -0.776018 |
| C | -4.981845 | 3.187586 | -0.966448 |
| C | 4.636615 | 4.455057 | -0.711739 |
| C | 5.185072 | 3.162970 | -0.692138 |
| C | 6.532143 | 2.877647 | -1.022880 |
| C | 6.838708 | 1.536101 | -1.029695 |
| C | 5.737348 | 0.707166 | -0.713351 |
| S | 4.313277 | 1.676388 | -0.373115 |
| C | 6.838706 | -1.536110 | -1.029697 |
| C | 5.737347 | -0.707174 | -0.713352 |
| C | 6.532138 | -2.877655 | -1.022885 |
| C | 5.185067 | -3.162977 | -0.692143 |
| S | 4.313275 | -1.676394 | -0.373117 |
| S | -4.053516 | 1.721430 | -0.678695 |
| C | -5.413549 | 0.715245 | -1.149760 |
| C | -6.513213 | 1.507158 | -1.519614 |
| C | -6.272658 | 2.865064 | -1.416738 |
| C | -5.413550 | -0.715236 | -1.149758 |
| C | -6.513217 | -1.507147 | -1.519609 |
| S | -4.053521 | -1.721424 | -0.678689 |
| C | -4.981851 | -3.187577 | -0.966441 |

| | | | |
|---|---|---|---|
| C | -6.272665 | -2.865053 | -1.416731 |
| C | -4.547925 | -4.518085 | -0.776011 |
| C | 4.636608 | -4.455063 | -0.711745 |
| C | -3.325421 | -4.984030 | -0.331123 |
| C | 3.343989 | -4.882212 | -0.419793 |
| N | -2.283615 | -4.150006 | -0.011630 |
| C | -1.258731 | -4.943220 | 0.312008 |
| C | -1.632985 | -6.357679 | 0.224550 |
| C | -2.931950 | -6.378237 | -0.162852 |
| C | 2.833838 | -6.221849 | -0.710229 |
| C | 1.522471 | -6.204983 | -0.387436 |
| C | 1.255759 | -4.864962 | 0.159487 |
| N | 2.361080 | -4.104265 | 0.120122 |
| C | -0.034281 | 3.080379 | 1.316538 |
| C | 0.206134 | 0.694068 | 1.886478 |
| C | -1.016867 | 1.235913 | 2.335122 |
| C | -1.147998 | 2.581092 | 2.028432 |
| C | 0.206134 | -0.694069 | 1.886478 |
| C | -1.016868 | -1.235914 | 2.335122 |
| S | -2.145251 | 0.000001 | 2.897866 |
| S | 1.250450 | -1.871618 | 1.170955 |
| C | -0.034285 | -3.080381 | 1.316537 |
| C | -1.148003 | -2.581093 | 2.028431 |
| C | 0.019096 | -4.330642 | 0.653833 |
| H | -3.562825 | 7.241626 | -0.315263 |
| H | -1.021584 | 7.204022 | 0.492138 |
| H | -5.296041 | 5.269037 | -1.013121 |
| H | 5.322877 | 5.226453 | -1.048243 |
| H | 7.239636 | 3.662768 | -1.255423 |
| H | 7.239631 | -3.662776 | -1.255428 |
| H | -7.002777 | 3.627912 | -1.653666 |
| H | -7.002784 | -3.627901 | -1.653658 |
| H | -5.296051 | -5.269027 | -1.013115 |
| H | 5.322868 | -5.226460 | -1.048250 |
| H | -1.021593 | -7.204022 | 0.492132 |
| H | -3.562838 | -7.241621 | -0.315257 |
| H | 3.403007 | -7.037755 | -1.130353 |
| H | 0.818880 | -7.011044 | -0.500715 |
| H | -2.009988 | 3.187724 | 2.251143 |
| H | -2.009993 | -3.187722 | 2.251139 |
| C | 1.255766 | 4.864960 | 0.159490 |
| C | 1.522481 | 6.204982 | -0.387432 |
| N | 2.361086 | 4.104261 | 0.120126 |
| C | 2.833848 | 6.221845 | -0.710224 |
| H | 0.818890 | 7.011043 | -0.500712 |
| C | 3.343996 | 4.882208 | -0.419787 |
| H | 3.403018 | 7.037750 | -1.130347 |
| S | 1.250453 | 1.871615 | 1.170954 |
| H | -7.456614 | 1.096258 | -1.847719 |
| H | -7.456616 | -1.096246 | -1.847716 |
| H | 7.819172 | -1.148820 | -1.267344 |
| H | 7.819173 | 1.148811 | -1.267343 |

$7^{2+}$
State = T1
Symmetry = $C_1$
E = -3500.28362611 a.u.

| | | | |
|---|---|---|---|
| C | -3.283474 | 4.569535 | -0.000049 |
| C | -2.555974 | 5.840317 | -0.000156 |
| C | -1.238774 | 5.517843 | 0.000056 |
| C | -1.206016 | 4.041110 | 0.000253 |
| N | -2.428820 | 3.509077 | 0.000051 |
| C | 0.000852 | 3.289599 | 0.000364 |
| C | -4.664801 | 4.371862 | -0.000221 |
| C | -5.296228 | 3.119942 | -0.000249 |
| C | 4.666790 | 4.370299 | 0.000128 |
| C | 5.297723 | 3.118230 | -0.000007 |
| C | 6.699615 | 2.907331 | -0.000176 |
| C | 7.065477 | 1.577868 | -0.000276 |
| C | 5.953649 | 0.706509 | -0.000204 |
| S | 4.441306 | 1.591162 | 0.000008 |
| C | 7.064763 | -1.580411 | -0.000241 |
| C | 5.953310 | -0.708536 | -0.000223 |
| C | 6.698303 | -2.909683 | -0.000194 |
| C | 5.296289 | -3.119982 | -0.000115 |
| S | 4.440567 | -1.592483 | -0.000096 |
| S | -4.440485 | 1.592523 | -0.000033 |
| C | -5.953248 | 0.708500 | -0.000157 |
| C | -7.064676 | 1.580377 | -0.000474 |
| C | -6.698201 | 2.909676 | -0.000533 |
| C | -5.953565 | -0.706504 | -0.000054 |
| C | -7.065415 | -1.577884 | -0.000057 |
| S | -4.441218 | -1.591206 | 0.000040 |
| C | -5.297666 | -3.118250 | 0.000130 |
| C | -6.699569 | -2.907331 | 0.000041 |
| C | -4.666787 | -4.370376 | 0.000195 |
| C | 4.664846 | -4.371757 | -0.000089 |
| C | -3.285492 | -4.568496 | 0.000197 |
| C | 3.283362 | -4.569364 | -0.000057 |
| N | -2.430559 | -3.508313 | 0.000126 |
| C | -1.207897 | -4.040734 | 0.000055 |
| C | -1.241119 | -5.517463 | 0.000221 |
| C | -2.558415 | -5.839529 | 0.000135 |
| C | 2.555917 | -5.840215 | -0.000254 |
| C | 1.238708 | -5.517794 | 0.000092 |
| C | 1.205883 | -4.041099 | -0.000071 |
| N | 2.428852 | -3.509036 | 0.000049 |
| C | 0.000606 | 1.893505 | 0.000023 |
| C | 0.000031 | 0.672125 | 0.000295 |
| C | -0.000470 | -0.672115 | 0.000242 |
| C | -0.000646 | -1.893510 | 0.000113 |
| C | -0.000817 | -3.289608 | -0.000098 |
| H | -3.003824 | 6.823547 | -0.000344 |
| H | -5.311494 | 5.243266 | -0.000369 |
| H | 5.313739 | 5.241514 | 0.000147 |
| H | 7.401570 | 3.731110 | -0.000185 |
| H | 7.399883 | -3.733782 | -0.000190 |
| H | -7.399795 | 3.733761 | -0.000703 |
| H | -7.401536 | -3.731100 | 0.000096 |
| H | -5.313790 | -5.241551 | 0.000209 |

| | | | |
|---|---|---|---|
| H | 5.311438 | -5.243236 | -0.000127 |
| H | -3.006573 | -6.822619 | 0.000105 |
| H | 3.003809 | -6.823426 | -0.000414 |
| C | 1.207792 | 4.040738 | 0.000301 |
| C | 1.241062 | 5.517423 | 0.000246 |
| N | 2.430576 | 3.508289 | 0.000311 |
| C | 2.558364 | 5.839454 | 0.000296 |
| C | 3.285422 | 4.568386 | 0.000248 |
| H | 3.006549 | 6.822532 | 0.000263 |
| H | -8.090246 | 1.238195 | -0.000623 |
| H | -8.090820 | -1.235212 | -0.000084 |
| H | 8.090320 | -1.238195 | -0.000327 |
| H | 8.090883 | 1.235201 | -0.000434 |
| C | 0.001286 | 6.367171 | 0.000083 |
| C | -0.001320 | -6.367168 | 0.000237 |
| H | -0.001458 | -7.029155 | -0.873325 |
| H | -0.001372 | -7.029059 | 0.873870 |
| H | 0.001315 | 7.029209 | 0.873602 |
| H | 0.001457 | 7.029009 | -0.873593 |

$8^{2+}$
State = T1
Symmetry = $C_1$
E = -4003.05615603 a.u.

| | | | |
|---|---|---|---|
| C | -2.922377 | 4.780026 | -0.000025 |
| C | -2.122847 | 5.993505 | -0.000004 |
| C | -0.829206 | 5.580448 | 0.000011 |
| C | -0.876785 | 4.115326 | -0.000007 |
| N | -2.160581 | 3.662222 | -0.000030 |
| C | 0.286290 | 3.299453 | -0.000004 |
| C | -5.578626 | 3.127179 | -0.000086 |
| C | 5.012576 | 4.335724 | 0.000029 |
| C | 5.668210 | 3.095109 | 0.000011 |
| C | 7.075770 | 2.908415 | 0.000007 |
| C | 7.459527 | 1.588314 | -0.000007 |
| C | 6.354990 | 0.699647 | -0.000013 |
| S | 4.828527 | 1.568064 | -0.000013 |
| C | 7.459602 | -1.588069 | -0.000024 |
| C | 6.355023 | -0.699453 | -0.000019 |
| C | 7.075909 | -2.908188 | -0.000020 |
| C | 5.668358 | -3.094943 | -0.000013 |
| S | 4.828601 | -1.567942 | -0.000021 |
| S | -4.732044 | 1.602973 | -0.000040 |
| C | -6.227861 | 0.718629 | -0.000073 |
| C | -7.327642 | 1.575134 | -0.000146 |
| C | -6.963184 | 2.922083 | -0.000152 |
| C | -6.227828 | -0.718837 | -0.000039 |
| C | -7.327570 | -1.575391 | -0.000004 |
| S | -4.731969 | -1.603112 | -0.000045 |
| C | -5.578482 | -3.127358 | 0.000027 |
| C | -6.963050 | -2.922323 | 0.000033 |
| C | 5.012772 | -4.335579 | 0.000005 |
| C | -2.922163 | -4.780108 | 0.000069 |
| C | 3.640296 | -4.514113 | 0.000016 |
| N | -2.160398 | -3.662282 | 0.000031 |
| C | -0.876585 | -4.115345 | 0.000036 |
| C | -0.828964 | -5.580464 | 0.000087 |
| C | -2.122593 | -5.993560 | 0.000098 |
| C | 2.944930 | -5.791103 | 0.000054 |
| C | 1.626102 | -5.496740 | 0.000068 |
| C | 1.548468 | -4.011726 | 0.000021 |
| N | 2.749223 | -3.457057 | -0.000002 |
| C | 0.246140 | 1.901263 | -0.000013 |
| C | 0.241256 | 0.673414 | 0.000011 |
| C | 0.241053 | -0.673395 | -0.000019 |
| C | 0.246263 | -1.901247 | -0.000034 |
| C | 0.286466 | -3.299435 | 0.000004 |
| H | -2.493020 | 7.008797 | 0.000002 |
| H | 5.641628 | 5.219496 | 0.000042 |
| H | 7.762675 | 3.744530 | 0.000018 |
| H | 7.762852 | -3.744271 | -0.000017 |
| H | -7.679582 | 3.733785 | -0.000197 |
| H | -7.679411 | -3.734057 | 0.000070 |
| H | 5.641850 | -5.219333 | 0.000017 |
| H | -2.492736 | -7.008863 | 0.000131 |
| H | 3.412988 | -6.764629 | 0.000076 |
| C | 1.548268 | 4.011786 | 0.000012 |
| C | 1.625864 | 5.496800 | 0.000033 |
| N | 2.749041 | 3.457153 | 0.000012 |
| C | 2.944687 | 5.791196 | 0.000040 |
| C | 3.640093 | 4.514227 | 0.000030 |
| H | 3.412719 | 6.764734 | 0.000053 |
| H | -8.352706 | 1.231878 | -0.000191 |
| H | -8.352650 | -1.232183 | 0.000004 |
| H | 8.488717 | -1.256220 | -0.000026 |
| H | 8.488659 | 1.256515 | -0.000008 |
| C | 0.429244 | 6.388062 | 0.000039 |
| C | 0.429509 | -6.388038 | 0.000121 |
| H | 0.455268 | -7.049638 | -0.873816 |
| H | 0.455279 | -7.049538 | 0.874134 |
| H | 0.454979 | 7.049595 | 0.874028 |
| H | 0.454996 | 7.049630 | -0.873922 |
| Si | -4.710508 | -4.691255 | 0.000081 |
| H | -5.523714 | -5.916774 | 0.000145 |
| Si | -4.710714 | 4.691114 | -0.000056 |
| H | -5.523967 | 5.916601 | -0.000052 |

1
State = S0
Symmetry = $D_3$
E = -6930.89457247 a.u.

| | | | |
|---|---|---|---|
| C | -0.650187 | 0.256725 | 6.311259 |
| C | -1.404683 | 0.787948 | 7.381970 |
| H | -1.018168 | 0.829342 | 8.391991 |
| C | -2.619852 | 1.314545 | 7.006285 |
| H | -3.307606 | 1.795426 | 7.690484 |
| C | -2.919508 | 1.192335 | 5.622750 |
| C | -4.038037 | 1.685057 | 4.968020 |
| C | -4.448232 | 1.478093 | 3.634549 |
| C | -5.324857 | 2.295588 | 2.902292 |

| | | | |
|---|---|---|---|
| H | -5.777049 | 3.178018 | 3.337556 |
| C | -5.523767 | 1.891281 | 1.588511 |
| H | -6.161974 | 2.410434 | 0.886345 |
| C | -4.870245 | 0.699902 | 1.248761 |
| C | -4.883257 | -0.000000 | 0.000000 |
| C | -4.870245 | 0.731508 | -1.230514 |
| C | -5.523767 | 0.430051 | -2.432153 |
| H | -6.161974 | -0.437619 | -2.530670 |
| C | -5.324857 | 1.365665 | -3.439184 |
| H | -5.777049 | 1.301399 | -4.421023 |
| C | -4.448232 | 2.408565 | -3.097341 |
| C | -4.870245 | -1.431410 | -0.018247 |
| C | -5.523767 | -2.321332 | 0.843641 |
| H | -6.161974 | -1.972815 | 1.644324 |
| C | -5.324857 | -3.661253 | 0.536891 |
| H | -5.777049 | -4.479418 | 1.083466 |
| C | -4.448232 | -3.886659 | -0.537208 |
| C | -4.038037 | -5.144960 | -1.024708 |
| C | -2.919508 | -5.465612 | -1.778783 |
| C | -2.619852 | -6.724893 | -2.364713 |
| H | -3.307606 | -7.557868 | -2.290357 |
| C | -1.404683 | -6.786947 | -3.008602 |
| H | -1.018168 | -7.682348 | -3.477764 |
| C | -0.650187 | -5.594073 | -2.933299 |
| C | 0.650187 | -5.337349 | -3.377960 |
| C | 1.404683 | -5.999000 | -4.373368 |
| H | 1.018168 | -6.853006 | -4.914226 |
| C | 2.619852 | -5.410348 | -4.641572 |
| H | 3.307606 | -5.762442 | -5.400127 |
| C | 2.919508 | -4.273277 | -3.843967 |
| C | 4.038037 | -3.459903 | -3.943313 |
| S | -1.574293 | 0.374233 | 4.813013 |
| S | -3.932725 | 0.127483 | 2.628342 |
| S | -3.932725 | 2.212469 | -1.424574 |
| S | -3.932725 | -2.339952 | -1.203768 |
| S | -1.574293 | -4.355308 | -2.082411 |
| S | 1.574293 | -3.981075 | -2.730602 |
| C | 0.650187 | -0.256725 | 6.311259 |
| C | 1.404683 | -0.787948 | 7.381970 |
| H | 1.018168 | -0.829342 | 8.391991 |
| C | 2.619852 | -1.314545 | 7.006285 |
| H | 3.307606 | -1.795426 | 7.690484 |
| C | 2.919508 | -1.192335 | 5.622750 |
| C | 4.038037 | -1.685057 | 4.968020 |
| C | 4.448232 | -1.478093 | 3.634549 |
| C | 5.324857 | -2.295588 | 2.902292 |
| H | 5.777049 | -3.178018 | 3.337556 |
| C | 5.523767 | -1.891281 | 1.588511 |
| H | 6.161974 | -2.410434 | 0.886345 |
| C | 4.870245 | -0.699902 | 1.248761 |
| C | 4.883257 | -0.000000 | 0.000000 |
| C | 4.870245 | -0.731508 | -1.230514 |
| C | 5.523767 | -0.430051 | -2.432153 |
| H | 6.161974 | 0.437619 | -2.530670 |
| C | 5.324857 | -1.365665 | -3.439184 |
| H | 5.777049 | -1.301399 | -4.421023 |
| C | 4.448232 | -2.408565 | -3.097341 |
| C | 4.870245 | 1.431410 | -0.018247 |
| C | 5.523767 | 2.321332 | 0.843641 |
| H | 6.161974 | 1.972815 | 1.644324 |
| C | 5.324857 | 3.661253 | 0.536891 |
| H | 5.777049 | 4.479418 | 1.083466 |
| C | 4.448232 | 3.886659 | -0.537208 |
| C | 4.038037 | 5.144960 | -1.024708 |
| C | 2.919508 | 5.465612 | -1.778783 |
| C | 2.619852 | 6.724893 | -2.364713 |
| H | 3.307606 | 7.557868 | -2.290357 |
| C | 1.404683 | 6.786947 | -3.008602 |
| H | 1.018168 | 7.682348 | -3.477764 |
| C | 0.650187 | 5.594073 | -2.933299 |
| C | -0.650187 | 5.337349 | -3.377960 |
| C | -1.404683 | 5.999000 | -4.373368 |
| H | -1.018168 | 6.853006 | -4.914226 |
| C | -2.619852 | 5.410348 | -4.641572 |
| H | -3.307606 | 5.762442 | -5.400127 |
| C | -2.919508 | 4.273277 | -3.843967 |
| C | -4.038037 | 3.459903 | -3.943313 |
| S | 1.574293 | -0.374233 | 4.813013 |
| S | 3.932725 | -0.127483 | 2.628342 |
| S | 3.932725 | -2.212469 | -1.424574 |
| S | 3.932725 | 2.339952 | -1.203768 |
| S | 1.574293 | 4.355308 | -2.082411 |
| S | -1.574293 | 3.981075 | -2.730602 |
| H | 4.664883 | -3.627350 | -4.815660 |
| H | 4.664883 | -2.356809 | 5.549207 |
| H | 4.664883 | 5.984159 | -0.733547 |
| H | -4.664883 | -5.984159 | -0.733547 |
| H | -4.664883 | 2.356809 | 5.549207 |
| H | -4.664883 | 3.627350 | -4.815660 |

$1^{6+}$
State = S0
Symmetry = $D_3$
E = -6928.58500496 a.u.

| | | | |
|---|---|---|---|
| C | 0.689784 | -3.199553 | -5.499309 |
| C | 1.471670 | -3.428606 | -6.650749 |
| H | 1.092408 | -3.917023 | -7.539114 |
| C | 2.746519 | -2.902929 | -6.554415 |
| H | 3.478845 | -2.947626 | -7.352215 |
| C | 3.024638 | -2.290040 | -5.304197 |
| C | 4.203623 | -1.616335 | -4.984863 |
| C | 4.637575 | -1.052659 | -3.766047 |
| C | 5.579858 | -0.004300 | -3.678768 |
| H | 6.104107 | 0.378294 | -4.546521 |
| C | 5.733401 | 0.501961 | -2.396963 |
| H | 6.412236 | 1.307366 | -2.148483 |
| C | 5.008119 | -0.210629 | -1.424214 |
| C | 5.012897 | 0.001135 | -0.000239 |
| C | 5.007841 | 1.340182 | 0.528378 |
| C | 5.733082 | 1.826416 | 1.631882 |

| | | | |
|---|---|---|---|
| H | 6.412175 | 1.208686 | 2.205006 |
| C | 5.579150 | 3.189535 | 1.834547 |
| H | 6.103313 | 3.749787 | 2.599777 |
| C | 4.636625 | 3.789140 | 0.970443 |
| C | 5.008552 | -1.126202 | 0.895064 |
| C | 5.734065 | -2.324786 | 0.764073 |
| H | 6.412833 | -2.512051 | -0.057779 |
| C | 5.580771 | -3.181948 | 1.843251 |
| H | 6.105173 | -4.124680 | 1.945558 |
| C | 4.638432 | -2.733731 | 2.794921 |
| C | 4.204614 | -3.507755 | 3.892312 |
| C | 3.025531 | -3.447958 | 4.635289 |
| C | 2.747504 | -4.224566 | 5.790980 |
| H | 3.480013 | -4.892939 | 6.228578 |
| C | 1.472515 | -4.045735 | 6.294202 |
| H | 1.093299 | -4.571190 | 7.161194 |
| C | 0.690417 | -3.163203 | 5.520157 |
| C | -0.688378 | -2.838948 | 5.693900 |
| C | -1.470231 | -2.993757 | 6.857699 |
| H | -1.090800 | -3.423850 | 7.775648 |
| C | -2.745253 | -2.475848 | 6.727759 |
| H | -3.477585 | -2.469405 | 7.526779 |
| C | -3.023575 | -1.944752 | 5.440700 |
| C | -4.202783 | -1.293416 | 5.078708 |
| S | 1.609855 | -2.385335 | -4.258864 |
| S | 4.059884 | -1.488066 | -2.166747 |
| S | 4.059130 | 2.621791 | -0.206369 |
| S | 4.060454 | -1.130992 | 2.372731 |
| S | 1.610480 | -2.495468 | 4.195077 |
| S | -1.608749 | -2.106565 | 4.403641 |
| C | -0.688886 | -3.512575 | -5.304942 |
| C | -1.470551 | -4.443652 | -6.020290 |
| H | -1.091076 | -5.023899 | -6.851493 |
| C | -2.745464 | -4.590182 | -5.506512 |
| H | -3.477639 | -5.285812 | -5.899959 |
| C | -3.023853 | -3.740652 | -4.403410 |
| C | -4.202954 | -3.752822 | -3.658143 |
| C | -4.637148 | -2.909839 | -2.612951 |
| C | -5.579621 | -3.296063 | -1.634626 |
| H | -6.103852 | -4.243514 | -1.676181 |
| C | -5.733355 | -2.371232 | -0.612883 |
| H | -6.412319 | -2.505325 | 0.219140 |
| C | -5.008041 | -1.183453 | -0.820597 |
| C | -5.012893 | -0.000762 | 0.000278 |
| C | -5.007940 | -0.120339 | 1.434902 |
| C | -5.733228 | 0.653373 | 2.359806 |
| H | -6.412255 | 1.440948 | 2.060010 |
| C | -5.579443 | 0.230857 | 3.671558 |
| H | -6.103674 | 0.668505 | 4.512899 |
| C | -4.636984 | -0.809550 | 3.826131 |
| C | -5.008536 | 1.301434 | -0.613525 |
| C | -5.734103 | 1.715244 | -1.745965 |
| H | -6.412893 | 1.061526 | -2.278064 |
| C | -5.580869 | 3.062565 | -2.035988 |
| H | -6.105363 | 3.572127 | -2.835652 |
| C | -4.638528 | 3.717006 | -1.212417 |
| C | -4.204851 | 5.043855 | -1.419746 |
| C | -3.025838 | 5.683434 | -1.036738 |
| C | -2.747957 | 7.063687 | -1.220398 |
| H | -3.480528 | 7.752186 | -1.625483 |
| C | -1.473036 | 7.435598 | -0.836891 |
| H | -1.093940 | 8.445740 | -0.923406 |
| C | -0.690842 | 6.350587 | -0.389011 |
| C | 0.687903 | 6.362705 | -0.021123 |
| C | 1.469519 | 7.474541 | 0.356452 |
| H | 1.089941 | 8.487969 | 0.378044 |
| C | 2.744515 | 7.128550 | 0.763258 |
| H | 3.476664 | 7.841929 | 1.123580 |
| C | 3.023043 | 5.739493 | 0.668494 |
| C | 4.202262 | 5.126352 | 1.091974 |
| S | -1.609241 | -2.761006 | -4.025777 |
| S | -4.059480 | -1.283183 | -2.294598 |
| S | -4.059345 | -1.347043 | 2.258164 |
| S | -4.060406 | 2.628158 | 0.037074 |
| S | -1.610704 | 4.866699 | -0.378139 |
| S | 1.608464 | 4.881593 | 0.063207 |
| H | -4.873886 | -1.070670 | 5.906593 |
| H | -4.873935 | -4.581348 | -3.878850 |
| H | -4.876223 | 5.649185 | -2.026556 |
| H | 4.875901 | -4.320090 | 4.165937 |
| H | 4.874708 | -1.447153 | -5.825343 |
| H | 4.873211 | 5.769727 | 1.658792 |

$9^{6+}$
State = S0
Symmetry = $C_2$
E = -7480.53034941 a.u.

| | | | |
|---|---|---|---|
| C | 2.551893 | -1.925022 | 5.790978 |
| C | 2.244781 | -2.537193 | 7.022168 |
| H | 2.833188 | -2.394003 | 7.919117 |
| C | 1.079119 | -3.281793 | 6.985145 |
| H | 0.665372 | -3.796271 | 7.844172 |
| C | 0.464303 | -3.323704 | 5.710922 |
| C | -0.753179 | -3.957974 | 5.425608 |
| C | -1.395004 | -4.199054 | 4.204087 |
| C | -2.775898 | -4.499483 | 4.081862 |
| H | -3.414032 | -4.632380 | 4.947237 |
| C | -3.218622 | -4.584656 | 2.777973 |
| H | -4.239379 | -4.817975 | 2.506807 |
| C | -2.183972 | -4.466980 | 1.821507 |
| C | -2.278178 | -4.614421 | 0.403928 |
| C | -3.458893 | -4.198323 | -0.311988 |
| C | -4.069500 | -4.879075 | -1.378769 |
| H | -3.689115 | -5.812854 | -1.770683 |
| C | -5.258114 | -4.301498 | -1.794539 |
| H | -5.896547 | -4.720309 | -2.563052 |
| C | -5.569453 | -3.093968 | -1.134073 |
| C | -1.191224 | -5.240112 | -0.319578 |
| C | -0.521492 | -6.422884 | 0.039896 |
| H | -0.764952 | -6.982815 | 0.933159 |

| | | | |
|---|---|---|---|
| C | 0.382604 | -6.857170 | -0.916028 |
| H | 0.944414 | -7.780605 | -0.841834 |
| C | 0.538641 | -5.964154 | -1.999419 |
| C | 1.498828 | -6.134882 | -3.010736 |
| C | 2.094640 | -5.213491 | -3.879021 |
| C | 2.962400 | -5.548456 | -4.945808 |
| H | 3.176536 | -6.575359 | -5.217307 |
| C | 3.502000 | -4.451807 | -5.596107 |
| H | 4.197213 | -4.529396 | -6.421857 |
| C | 3.117673 | -3.225351 | -5.022583 |
| C | 3.593844 | -1.923501 | -5.363835 |
| C | 4.198786 | -1.519614 | -6.572613 |
| H | 4.379730 | -2.194433 | -7.399006 |
| C | 4.471911 | -0.166912 | -6.617285 |
| H | 4.905307 | 0.333066 | -7.475551 |
| C | 4.137761 | 0.530379 | -5.424977 |
| C | 4.265236 | 1.902559 | -5.238645 |
| S | 1.388859 | -2.368046 | 4.561213 |
| S | -0.646562 | -4.152028 | 2.613645 |
| S | -4.367838 | -2.753341 | 0.103282 |
| S | -0.593551 | -4.624624 | -1.847773 |
| S | 1.992971 | -3.465002 | -3.707825 |
| S | 3.440757 | -0.588116 | -4.252264 |
| C | 3.610277 | -1.015760 | 5.514602 |
| C | 4.795300 | -0.823648 | 6.261460 |
| H | 5.011402 | -1.360615 | 7.175757 |
| C | 5.672825 | 0.059119 | 5.670311 |
| H | 6.647420 | 0.304349 | 6.075726 |
| C | 5.189482 | 0.637257 | 4.462710 |
| C | 5.908060 | 1.502384 | 3.648833 |
| C | 5.515741 | 2.214357 | 2.488666 |
| C | 6.414322 | 2.650737 | 1.495308 |
| H | 7.486824 | 2.530730 | 1.589449 |
| C | 5.791502 | 3.204559 | 0.384761 |
| H | 6.327702 | 3.590336 | -0.472401 |
| C | 4.398658 | 3.319279 | 0.524984 |
| C | 3.476797 | 3.924221 | -0.401449 |
| C | 3.640550 | 3.690939 | -1.816061 |
| C | 3.489374 | 4.610762 | -2.865118 |
| H | 3.251753 | 5.653597 | -2.701182 |
| C | 3.783312 | 4.074555 | -4.113303 |
| H | 3.774777 | 4.646331 | -5.033451 |
| C | 4.066892 | 2.695064 | -4.083733 |
| C | -1.184284 | 6.279865 | 0.505414 |
| C | -1.696109 | 7.480001 | 1.021613 |
| H | -1.069889 | 8.275412 | 1.403637 |
| C | -3.076664 | 7.565431 | 0.931384 |
| H | -3.649899 | 8.426374 | 1.254399 |
| C | -3.689266 | 6.410280 | 0.393730 |
| C | -5.075018 | 6.233851 | 0.304307 |
| C | -5.857246 | 5.110575 | 0.003369 |
| C | -7.264837 | 5.140469 | -0.124692 |
| H | -7.837699 | 6.052362 | -0.005007 |
| C | -7.828682 | 3.910281 | -0.418673 |
| H | -8.892675 | 3.756580 | -0.539557 |
| C | -6.882380 | 2.873721 | -0.501432 |
| C | -7.146179 | 1.494865 | -0.773711 |
| C | -8.333636 | 0.965379 | -1.314730 |
| H | -9.200830 | 1.568926 | -1.546902 |
| C | -8.254645 | -0.387945 | -1.579304 |
| H | -9.056793 | -0.962222 | -2.027394 |
| C | -7.016472 | -0.975716 | -1.217051 |
| C | -6.687425 | -2.307171 | -1.470251 |
| S | 3.585147 | 0.000750 | 4.094811 |
| S | 3.872145 | 2.644396 | 2.059337 |
| S | 4.093217 | 2.112085 | -2.430401 |
| S | -2.459461 | 5.236545 | -0.067918 |
| S | -5.263586 | 3.471996 | -0.233026 |
| S | -5.948120 | 0.246859 | -0.531191 |
| H | 4.514487 | 2.465895 | -6.136077 |
| H | 6.957245 | 1.617392 | 3.914699 |
| H | -5.652253 | 7.127768 | 0.532662 |
| H | 1.886480 | -7.148661 | -3.095028 |
| H | -1.312431 | -4.278192 | 6.302474 |
| H | -7.418211 | -2.830257 | -2.084326 |
| C | 0.195641 | 5.882221 | 0.460732 |
| C | 1.186046 | 6.251443 | 1.381537 |
| S | 0.824676 | 4.766212 | -0.721391 |
| C | 2.391220 | 5.584367 | 1.192426 |
| H | 1.011037 | 6.954121 | 2.185691 |
| C | 2.395767 | 4.729231 | 0.077214 |
| H | 3.266469 | 5.751526 | 1.806104 |

$10^{6+}$
State = S0
Symmetry = $C_2$
E = -8032.46839098 a.u.

| | | | |
|---|---|---|---|
| C | 5.462321 | 3.726916 | -3.677041 |
| C | 5.595745 | 4.855606 | -4.502718 |
| H | 6.395519 | 4.973825 | -5.221271 |
| C | 4.568099 | 5.769581 | -4.348108 |
| H | 4.485831 | 6.685503 | -4.920514 |
| C | 3.617519 | 5.405594 | -3.368268 |
| C | 2.466911 | 6.153711 | -3.087765 |
| C | 1.471879 | 6.036058 | -2.110879 |
| C | 0.247909 | 6.744816 | -2.167620 |
| H | 0.032622 | 7.454647 | -2.957029 |
| C | -0.623692 | 6.450632 | -1.137968 |
| H | -1.589616 | 6.921882 | -1.019613 |
| C | -0.088473 | 5.564533 | -0.181666 |
| C | -0.697395 | 5.152034 | 1.051136 |
| C | -2.141037 | 5.037543 | 1.147504 |
| C | -2.932129 | 5.560023 | 2.182001 |
| H | -2.511290 | 6.083449 | 3.029759 |
| C | -4.293013 | 5.447516 | 1.948924 |
| H | -5.053630 | 5.851284 | 2.606048 |
| C | -4.616055 | 4.747185 | 0.767961 |
| C | 0.097334 | 4.952802 | 2.224307 |
| C | 1.218781 | 5.712617 | 2.633683 |
| H | 1.601670 | 6.540845 | 2.053407 |

| | | | |
|---|---|---|---|
| C | 1.678430 | 5.386010 | 3.890017 |
| H | 2.479702 | 5.910188 | 4.396586 |
| C | 1.016919 | 4.276346 | 4.479131 |
| C | 1.383628 | 3.719851 | 5.704597 |
| C | 1.101418 | 2.465392 | 6.274450 |
| C | 1.377498 | 2.105589 | 7.611311 |
| H | 1.782337 | 2.809358 | 8.328233 |
| C | 1.078867 | 0.786686 | 7.910991 |
| H | 1.241610 | 0.344643 | 8.885191 |
| C | 0.616195 | 0.056504 | 6.801146 |
| C | 0.355514 | -1.339633 | 6.739088 |
| C | 0.097037 | -2.211911 | 7.822549 |
| H | 0.076601 | -1.881915 | 8.852690 |
| C | -0.190610 | -3.498400 | 7.427951 |
| H | -0.446460 | -4.297764 | 8.113207 |
| C | -0.118115 | -3.706006 | 6.020170 |
| C | -0.393554 | -4.901127 | 5.378185 |
| S | 4.053929 | 3.859700 | -2.649341 |
| S | 1.515701 | 5.032719 | -0.664913 |
| S | -3.143038 | 4.306687 | -0.092905 |
| S | -0.292791 | 3.735638 | 3.430255 |
| S | 0.478257 | 1.082621 | 5.389083 |
| S | 0.301135 | -2.189604 | 5.215470 |
| C | 6.306599 | 2.572624 | -3.681997 |
| C | 7.588862 | 2.476722 | -4.251321 |
| H | 8.084463 | 3.303941 | -4.741125 |
| C | 8.168553 | 1.232344 | -4.081925 |
| H | 9.159416 | 0.975751 | -4.437055 |
| C | 7.348994 | 0.303515 | -3.398847 |
| C | 7.731672 | -1.010114 | -3.113956 |
| C | 7.045733 | -2.081869 | -2.525185 |
| C | 7.665958 | -3.284006 | -2.125259 |
| H | 8.717457 | -3.477832 | -2.299867 |
| C | 6.813532 | -4.171273 | -1.482787 |
| H | 7.121749 | -5.142681 | -1.119695 |
| C | 5.494063 | -3.708541 | -1.408110 |
| C | 0.731160 | -5.395042 | 0.213515 |
| C | 0.221670 | -5.516739 | 1.558834 |
| C | -0.736596 | -6.421563 | 2.034145 |
| H | -1.193014 | -7.175618 | 1.406619 |
| C | -0.958621 | -6.320685 | 3.404238 |
| H | -1.626847 | -6.969665 | 3.956842 |
| C | -0.253692 | -5.268803 | 4.013932 |
| C | -3.345462 | -4.958235 | -3.140856 |
| C | -3.926561 | -5.626694 | -4.226333 |
| H | -3.522407 | -6.538301 | -4.645915 |
| C | -5.101903 | -5.033356 | -4.663859 |
| H | -5.705848 | -5.419306 | -5.476355 |
| C | -5.447402 | -3.858123 | -3.961300 |
| C | -6.546402 | -3.051751 | -4.278820 |
| C | -6.946073 | -1.791816 | -3.816127 |
| C | -8.155929 | -1.163569 | -4.186035 |
| H | -8.861158 | -1.623028 | -4.868178 |
| C | -8.351362 | 0.073684 | -3.593996 |
| H | -9.219704 | 0.692156 | -3.776993 |
| C | -7.287225 | 0.463228 | -2.764911 |
| C | -7.191981 | 1.678781 | -2.012445 |
| C | -8.263447 | 2.517649 | -1.662983 |
| H | -9.282879 | 2.339852 | -1.977772 |
| C | -7.894323 | 3.547013 | -0.815925 |
| H | -8.589839 | 4.271505 | -0.409775 |
| C | -6.513714 | 3.574361 | -0.510864 |
| C | -5.940786 | 4.474585 | 0.391021 |
| S | 5.823229 | 1.061698 | -2.953339 |
| S | 5.331431 | -2.126393 | -2.124445 |
| S | 0.797753 | -4.483191 | 2.855118 |
| S | -4.271097 | -3.552821 | -2.686374 |
| S | -6.041656 | -0.757695 | -2.716025 |
| S | -5.689808 | 2.247729 | -1.326825 |
| H | -0.818077 | -5.677034 | 6.012178 |
| H | 8.761200 | -1.239024 | -3.381990 |
| H | -7.204959 | -3.475965 | -5.034181 |
| H | 2.036615 | 4.347594 | 6.307515 |
| H | 2.299239 | 6.971900 | -3.785232 |
| H | -6.674352 | 5.058814 | 0.943595 |
| C | -2.125960 | -5.307992 | -2.461337 |
| C | -1.019986 | -5.951045 | -3.027345 |
| S | -1.818770 | -4.875913 | -0.802390 |
| C | 0.076768 | -6.018765 | -2.170763 |
| H | -1.008771 | -6.321464 | -4.043908 |
| C | -0.169288 | -5.484726 | -0.897827 |
| H | 1.012257 | -6.492091 | -2.437407 |
| C | 4.379630 | -4.387731 | -0.801856 |
| C | 4.448216 | -5.255968 | 0.293958 |
| S | 2.730765 | -4.148346 | -1.310096 |
| C | 3.199085 | -5.651160 | 0.763332 |
| H | 5.381698 | -5.550892 | 0.754689 |
| C | 2.127527 | -5.153755 | 0.006407 |
| H | 3.062802 | -6.325773 | 1.598059 |

$11^{6+}$
State = S0
Symmetry = $D_3$
E = -8584.39974949 a.u.

| | | | |
|---|---|---|---|
| C | -2.471940 | 7.119873 | -1.913112 |
| C | -3.443098 | 7.714916 | -2.730828 |
| H | -3.367896 | 8.729521 | -3.097839 |
| C | -4.468617 | 6.850273 | -3.078945 |
| H | -5.289216 | 7.121806 | -3.731475 |
| C | -4.354929 | 5.566794 | -2.507667 |
| C | -5.250303 | 4.518646 | -2.774494 |
| C | -5.415672 | 3.242527 | -2.235848 |
| C | -6.231358 | 2.255242 | -2.840733 |
| H | -6.795969 | 2.454521 | -3.743311 |
| C | -6.244366 | 1.047052 | -2.176539 |
| H | -6.840246 | 0.200730 | -2.488202 |
| C | -5.510629 | 1.054483 | -0.972453 |
| C | -5.461804 | -0.002410 | 0.002989 |
| C | -5.509458 | -1.375747 | -0.424496 |
| C | -6.242285 | -2.415324 | 0.184134 |

| | | | |
|---|---|---|---|
| H | -6.838435 | -2.262384 | 1.072777 |
| C | -6.228057 | -3.594805 | -0.529791 |
| H | -6.791844 | -4.476576 | -0.250916 |
| C | -5.412286 | -3.564101 | -1.687162 |
| C | -5.508844 | 0.313829 | 1.406162 |
| C | -6.242393 | 1.359710 | 2.002980 |
| H | -6.839726 | 2.052368 | 1.426892 |
| C | -6.227113 | 1.330803 | 3.381403 |
| H | -6.791179 | 2.012425 | 4.006195 |
| C | -5.409883 | 0.313847 | 3.932606 |
| C | -5.242130 | 0.142387 | 5.306838 |
| C | -4.345266 | -0.612408 | 6.079731 |
| C | -4.456644 | -0.759232 | 7.477109 |
| H | -5.276349 | -0.329866 | 8.039820 |
| C | -3.430163 | -1.492932 | 8.050259 |
| H | -3.353211 | -1.682316 | 9.112332 |
| C | -2.460510 | -1.903597 | 7.124521 |
| C | -1.231575 | -2.568204 | 7.436473 |
| C | -0.939652 | -3.266742 | 8.620440 |
| H | -1.654524 | -3.400426 | 9.420848 |
| C | 0.335674 | -3.801756 | 8.637196 |
| H | 0.733850 | -4.385298 | 9.458457 |
| C | 1.095535 | -3.516422 | 7.479222 |
| C | 2.412948 | -3.938210 | 7.290643 |
| S | -2.900941 | 5.470002 | -1.522284 |
| S | -4.711580 | 2.603546 | -0.749821 |
| S | -4.709754 | -1.957020 | -1.877150 |
| S | -4.707388 | -0.653184 | 2.635042 |
| S | -2.892105 | -1.417264 | 5.500943 |
| S | 0.131814 | -2.568902 | 6.348046 |
| C | -1.243512 | 7.724008 | -1.494572 |
| C | -0.953049 | 9.098971 | -1.483046 |
| H | -1.668858 | 9.857939 | -1.767916 |
| C | 0.322278 | 9.382775 | -1.029282 |
| H | 0.719516 | 10.386280 | -0.935925 |
| C | 1.083622 | 8.238389 | -0.696848 |
| C | 2.401311 | 8.287803 | -0.238340 |
| C | 3.334423 | 7.281741 | 0.051105 |
| C | 4.579141 | 7.518464 | 0.667528 |
| H | 4.915651 | 8.515168 | 0.925910 |
| C | 5.315178 | 6.366388 | 0.913880 |
| H | 6.297590 | 6.367700 | 1.366654 |
| C | 4.682993 | 5.204395 | 0.460000 |
| C | 5.616131 | 0.004004 | -0.003666 |
| C | 4.690789 | -2.998761 | 4.269094 |
| C | 5.323854 | -3.973679 | 5.046704 |
| H | 6.305863 | -4.366444 | 4.819701 |
| C | 4.589268 | -4.337284 | 6.168247 |
| H | 4.926591 | -5.060203 | 6.901062 |
| C | 3.344779 | -3.684961 | 6.273727 |
| C | 4.683696 | -2.197173 | -4.738104 |
| C | 5.315402 | -2.384277 | -5.971725 |
| H | 6.296923 | -1.991033 | -6.199998 |
| C | 4.580334 | -3.175455 | -6.845463 |
| H | 4.916706 | -3.449765 | -7.837951 |
| C | 3.336755 | -3.593134 | -6.331255 |
| C | 2.404550 | -4.348725 | -7.056978 |
| C | 1.087636 | -4.723231 | -6.784046 |
| C | 0.327200 | -5.584724 | -7.608251 |
| H | 0.724610 | -6.005320 | -8.524055 |
| C | -0.947609 | -5.837393 | -7.134989 |
| H | -1.662795 | -6.464646 | -7.649411 |
| C | -1.238519 | -5.159875 | -5.938607 |
| C | -2.466689 | -5.221524 | -5.205782 |
| C | -3.436482 | -6.228601 | -5.311431 |
| H | -3.360189 | -7.054147 | -6.006004 |
| C | -4.462132 | -6.098431 | -4.388617 |
| H | -5.281785 | -6.800341 | -4.296927 |
| C | -4.349860 | -4.961250 | -3.563520 |
| C | -5.245574 | -4.668800 | -2.522525 |
| S | 0.121169 | 6.783748 | -0.949541 |
| S | 3.133695 | 5.559838 | -0.256659 |
| S | 3.142474 | -2.556179 | 4.937704 |
| S | 3.135937 | -2.998394 | -4.686278 |
| S | 0.125119 | -4.215824 | -5.397598 |
| S | -2.897085 | -4.057852 | -3.973109 |
| H | 2.784443 | -4.593639 | 8.075744 |
| H | 2.771946 | 9.295994 | -0.064687 |
| H | 2.775220 | -4.702025 | -8.017060 |
| H | -5.932515 | 0.733726 | 5.904890 |
| H | -5.941481 | 4.739827 | -3.585256 |
| H | -5.935776 | -5.482268 | -2.308340 |
| C | 5.179068 | -1.432433 | -3.622392 |
| C | 6.014984 | -0.314882 | -3.694237 |
| S | 4.726309 | -1.771901 | -1.975956 |
| C | 6.223513 | 0.298680 | -2.459823 |
| H | 6.424510 | 0.056876 | -4.624006 |
| C | 5.601153 | -0.359438 | -1.391851 |
| H | 6.852606 | 1.168354 | -2.325493 |
| C | 5.185148 | -2.413161 | 3.049390 |
| C | 6.022389 | -3.032070 | 2.117278 |
| S | 4.728871 | -0.818430 | 2.520591 |
| C | 6.229108 | -2.269084 | 0.968857 |
| H | 6.434073 | -4.022301 | 2.259902 |
| C | 5.603914 | -1.016587 | 1.005162 |
| H | 6.858786 | -2.586001 | 0.148363 |
| C | 5.179107 | 3.855962 | 0.563013 |
| C | 6.017561 | 3.359583 | 1.564762 |
| S | 4.723939 | 2.599760 | -0.553099 |
| C | 6.226368 | 1.983861 | 1.478248 |
| H | 6.428691 | 3.978955 | 2.350718 |
| C | 5.601511 | 1.387897 | 0.375810 |
| H | 6.857326 | 1.432848 | 2.162652 |

$12^{6+}$
State = S0
Symmetry = $C_1$
E = -7480.53455160 a.u.

| | | | |
|---|---|---|---|
| C | 6.171279 | 2.956170 | -3.061852 |
| C | 6.511746 | 4.107804 | -3.794544 |

| | | | |
|---|---|---|---|
| H | 7.358245 | 4.156382 | -4.466340 |
| C | 5.628680 | 5.154453 | -3.603581 |
| H | 5.715600 | 6.113767 | -4.099852 |
| C | 4.580318 | 4.867008 | -2.693561 |
| C | 3.548798 | 5.754289 | -2.388087 |
| C | 2.467627 | 5.679423 | -1.488923 |
| C | 1.388422 | 6.588456 | -1.500191 |
| H | 1.351606 | 7.431346 | -2.179785 |
| C | 0.397713 | 6.299863 | -0.573654 |
| H | -0.489420 | 6.903871 | -0.439569 |
| C | 0.702021 | 5.201394 | 0.245870 |
| C | -0.075261 | 4.680337 | 1.342143 |
| C | -1.536642 | 4.818094 | 1.322304 |
| C | -2.277841 | 5.471609 | 2.307441 |
| H | -1.827841 | 5.888698 | 3.198675 |
| C | -3.622295 | 5.628222 | 1.975624 |
| H | -4.337755 | 6.158153 | 2.592834 |
| C | -3.980292 | 5.012007 | 0.766851 |
| C | 0.545752 | 4.133503 | 2.487397 |
| C | 1.763513 | 4.555803 | 3.094720 |
| H | 2.363668 | 5.359603 | 2.690644 |
| C | 2.014174 | 3.957307 | 4.302962 |
| H | 2.848194 | 4.212589 | 4.945215 |
| C | 1.088790 | 2.928453 | 4.644721 |
| C | 1.199084 | 2.129956 | 5.786561 |
| C | 0.659583 | 0.866419 | 6.077328 |
| C | 0.559599 | 0.308239 | 7.382706 |
| H | 0.855126 | 0.858508 | 8.267576 |
| C | 0.011340 | -0.948317 | 7.404729 |
| H | -0.174855 | -1.514784 | 8.309448 |
| C | -0.239477 | -1.491388 | 6.107878 |
| C | -0.701719 | -2.773537 | 5.865357 |
| S | 4.748033 | 3.222074 | -2.086517 |
| S | 2.231604 | 4.480035 | -0.227531 |
| S | -2.559710 | 4.313711 | -0.005612 |
| S | -0.204282 | 2.861320 | 3.454336 |
| S | 0.128543 | -0.286459 | 4.865814 |
| C | 6.839314 | 1.688876 | -3.093479 |
| C | 8.095442 | 1.406273 | -3.653337 |
| H | 8.715430 | 2.149940 | -4.135954 |
| C | 8.479288 | 0.083072 | -3.488896 |
| H | 9.423239 | -0.317501 | -3.839121 |
| C | 7.524608 | -0.710327 | -2.816314 |
| C | 7.702093 | -2.067267 | -2.504671 |
| C | 6.852263 | -2.996073 | -1.895143 |
| C | 7.271461 | -4.260235 | -1.422396 |
| H | 8.278132 | -4.630997 | -1.576005 |
| C | 6.289157 | -4.956131 | -0.729872 |
| H | 6.436529 | -5.938199 | -0.299928 |
| C | 5.063915 | -4.280164 | -0.693315 |
| C | 0.098567 | -4.918720 | 1.085127 |
| C | -0.380793 | -4.568724 | 2.398189 |
| C | -1.440874 | -5.138167 | 3.120048 |
| H | -2.022362 | -5.972329 | 2.749506 |
| C | -1.588985 | -4.602218 | 4.395326 |
| H | -2.315060 | -4.954543 | 5.117743 |
| C | -0.721004 | -3.527747 | 4.659244 |
| C | -3.975066 | -4.784754 | -2.274315 |
| C | -4.649518 | -5.549455 | -3.237305 |
| H | -4.362064 | -6.557986 | -3.503785 |
| C | -5.751542 | -4.897802 | -3.772297 |
| H | -6.407651 | -5.334908 | -4.515906 |
| C | -5.946596 | -3.590051 | -3.268482 |
| C | -6.939507 | -2.710086 | -3.707099 |
| C | -7.172087 | -1.357127 | -3.405966 |
| C | -8.280702 | -0.615962 | -3.866573 |
| H | -9.040954 | -1.045906 | -4.507952 |
| C | -8.305969 | 0.697381 | -3.414058 |
| H | -9.082203 | 1.403338 | -3.678117 |
| C | -7.205763 | 1.021348 | -2.607122 |
| C | -6.936339 | 2.275750 | -1.963843 |
| C | -7.861747 | 3.311109 | -1.734890 |
| H | -8.885215 | 3.280906 | -2.084059 |
| C | -7.350713 | 4.329946 | -0.954479 |
| H | -7.925104 | 5.192640 | -0.637859 |
| C | -5.994102 | 4.146257 | -0.580036 |
| C | -5.315652 | 4.980208 | 0.300721 |
| S | 6.127968 | 0.263965 | -2.381029 |
| S | 5.150391 | -2.745026 | -1.509723 |
| S | 0.385583 | -3.290009 | 3.325479 |
| S | -4.721271 | -3.226976 | -2.056842 |
| S | -6.141050 | -0.344660 | -2.406714 |
| S | -5.382294 | 2.638250 | -1.261824 |
| H | -1.112706 | -3.290742 | 6.730545 |
| H | 8.688483 | -2.456088 | -2.750845 |
| H | -7.648812 | -3.142163 | -4.410465 |
| H | 1.832961 | 2.541791 | 6.569501 |
| H | 3.565886 | 6.669962 | -2.975985 |
| H | -5.950641 | 5.731218 | 0.768208 |
| C | -2.790053 | -5.137640 | -1.536491 |
| C | -1.779443 | -6.019275 | -1.938853 |
| S | -2.395306 | -4.378165 | -0.021325 |
| C | -0.677409 | -6.018638 | -1.086023 |
| H | -1.834665 | -6.610508 | -2.843558 |
| C | -0.828748 | -5.179046 | 0.028805 |
| H | 0.191816 | -6.645274 | -1.236007 |
| C | 3.854938 | -4.702327 | -0.023741 |
| C | 3.796173 | -5.343224 | 1.214972 |
| S | 2.261085 | -4.328111 | -0.619405 |
| C | 2.499080 | -5.439642 | 1.721686 |
| H | 4.675953 | -5.675750 | 1.750303 |
| C | 1.518947 | -4.945467 | 0.853633 |
| H | 2.262439 | -5.901299 | 2.671430 |

$13^{6+}$
State = S0
Symmetry = $C_1$
E = -6928.51554229 a.u.

| | | | |
|---|---|---|---|
| C | 7.564244 | 1.475112 | -1.902800 |
| C | 8.451291 | 2.529004 | -2.166150 |

| | | | |
|---|---|---|---|
| H | 9.368066 | 2.411533 | -2.729859 |
| C | 8.036115 | 3.734852 | -1.614975 |
| H | 8.589388 | 4.662116 | -1.712959 |
| C | 6.805696 | 3.654811 | -0.912379 |
| C | 6.113015 | 4.741959 | -0.380340 |
| C | 4.859455 | 4.815338 | 0.274143 |
| C | 4.164227 | 6.015971 | 0.511002 |
| H | 4.585817 | 6.987794 | 0.281734 |
| C | 2.884948 | 5.833078 | 1.042900 |
| H | 2.216744 | 6.649044 | 1.286547 |
| C | 2.582664 | 4.494018 | 1.293174 |
| C | 1.355611 | 3.895160 | 1.814284 |
| C | 0.058220 | 4.368828 | 1.363278 |
| C | -1.101112 | 4.441607 | 2.151302 |
| H | -1.099950 | 4.237496 | 3.213199 |
| C | -2.231275 | 4.838649 | 1.447661 |
| H | -3.204406 | 4.922583 | 1.908819 |
| C | -1.988324 | 5.085503 | 0.088334 |
| C | 1.435351 | 2.829451 | 2.732311 |
| C | 2.405759 | 2.656480 | 3.760200 |
| H | 3.215676 | 3.357365 | 3.915121 |
| C | 2.136050 | 1.611003 | 4.609183 |
| H | 2.722337 | 1.380153 | 5.490099 |
| C | 1.022541 | 0.817054 | 4.217289 |
| C | -0.135474 | -1.445117 | 4.548350 |
| C | -0.741553 | -2.355678 | 5.488787 |
| C | -1.398372 | -3.395941 | 4.919070 |
| C | -1.253551 | -3.470302 | 3.480323 |
| S | 6.206347 | 2.000590 | -0.947770 |
| S | 3.901273 | 3.449077 | 0.815787 |
| S | -0.275154 | 4.879623 | -0.268893 |
| S | 0.233109 | 1.522325 | 2.825287 |
| S | -0.352351 | -2.037183 | 2.900067 |
| C | 7.600734 | 0.110118 | -2.377189 |
| C | 8.228577 | -0.388237 | -3.533720 |
| H | 8.884585 | 0.203695 | -4.159128 |
| C | 7.839634 | -1.685747 | -3.843417 |
| H | 8.175721 | -2.221407 | -4.724055 |
| C | 6.942431 | -2.248980 | -2.904274 |
| C | 6.199782 | -3.426197 | -3.011692 |
| C | 5.422094 | -3.968245 | -1.956714 |
| C | 5.757376 | -4.005655 | -0.595275 |
| H | 6.733370 | -3.724002 | -0.222152 |
| C | 4.748822 | -4.529829 | 0.203244 |
| H | 4.833295 | -4.630788 | 1.276305 |
| C | 3.587282 | -4.851560 | -0.512118 |
| C | -1.404147 | -4.737861 | 1.275294 |
| C | -5.778696 | -3.565022 | -1.386631 |
| C | -6.063673 | -3.272379 | -2.737532 |
| H | -5.527277 | -3.716353 | -3.565616 |
| C | -7.022750 | -2.284427 | -2.892455 |
| H | -7.334738 | -1.910904 | -3.858462 |
| C | -7.549073 | -1.818681 | -1.671600 |
| C | -8.211186 | -0.642248 | -1.287359 |
| C | -8.180594 | 0.618276 | -1.920869 |
| C | -7.839156 | 1.046348 | -3.218328 |
| H | -7.921464 | 0.419871 | -4.096278 |
| C | -7.374432 | 2.352332 | -3.258771 |
| H | -7.041865 | 2.841707 | -4.166368 |
| C | -7.320834 | 2.962718 | -1.990036 |
| C | -6.462069 | 4.034443 | -1.552341 |
| C | -6.457062 | 4.657251 | -0.278262 |
| H | -7.314337 | 4.647303 | 0.384087 |
| C | -5.250052 | 5.240133 | 0.038302 |
| H | -5.071594 | 5.769925 | 0.964750 |
| C | -4.278344 | 5.124743 | -0.988859 |
| C | -2.891002 | 5.227726 | -1.001978 |
| S | 6.626549 | -1.093984 | -1.604083 |
| S | 3.825135 | -4.663026 | -2.224029 |
| S | -6.883153 | -2.767451 | -0.326703 |
| S | -8.071388 | 1.967449 | -0.784890 |
| S | -5.021714 | 4.399091 | -2.435971 |
| H | 6.164461 | -3.897035 | -3.993116 |
| H | -8.576729 | -0.632084 | -0.262488 |
| H | 6.591951 | 5.707063 | -0.537407 |
| H | -2.429659 | 5.253267 | -1.986680 |
| C | -4.585830 | -4.223763 | -0.905323 |
| C | -3.719073 | -5.058450 | -1.626757 |
| S | -3.931109 | -3.845846 | 0.661483 |
| C | -2.535062 | -5.339000 | -0.952965 |
| H | -3.962027 | -5.464763 | -2.600058 |
| C | -2.450672 | -4.734151 | 0.316499 |
| H | -1.778528 | -6.008498 | -1.341064 |
| C | 2.322829 | -5.125732 | 0.133091 |
| C | 2.191339 | -5.576160 | 1.454846 |
| S | 0.774993 | -4.700998 | -0.545515 |
| C | 0.897260 | -5.489639 | 1.937979 |
| H | 3.021150 | -5.959596 | 2.033064 |
| C | -0.038115 | -5.010587 | 0.997619 |
| H | 0.613494 | -5.804025 | 2.933117 |
| C | 0.582009 | -0.322100 | 4.931879 |
| H | 0.876510 | -0.317271 | 5.979854 |
| C | -1.734234 | -4.474336 | 2.694023 |
| H | -2.415711 | -5.182884 | 3.163069 |
| H | -0.693172 | -2.178206 | 6.556430 |
| H | -1.939542 | -4.153408 | 5.473626 |

$1^{3+}$
State = Q
Symmetry = $D_3$
E = -6930.09936079 a.u.

| | | | |
|---|---|---|---|
| C | -0.657291 | -4.985413 | -3.847136 |
| C | -1.404708 | -6.141884 | -4.151997 |
| H | -1.016436 | -6.942255 | -4.767088 |
| C | -2.635053 | -6.181622 | -3.530247 |
| H | -3.329725 | -7.007066 | -3.617432 |
| C | -2.931299 | -5.036144 | -2.748980 |
| C | -4.070805 | -4.845291 | -1.973973 |
| C | -4.497873 | -3.694384 | -1.281845 |
| C | -5.395481 | -3.687713 | -0.197809 |

| | | | |
|---|---|---|---|
| H | -5.869152 | -4.592634 | 0.160442 |
| C | -5.581489 | -2.437574 | 0.372900 |
| H | -6.235545 | -2.250052 | 1.213422 |
| C | -4.907548 | -1.404927 | -0.297076 |
| C | -4.921148 | -0.001939 | -0.001827 |
| C | -4.909123 | 0.443702 | 1.360858 |
| C | -5.584054 | 1.539826 | 1.919768 |
| H | -6.237764 | 2.173837 | 1.336560 |
| C | -5.399438 | 1.670397 | 3.288004 |
| H | -5.873995 | 2.432769 | 3.892300 |
| C | -4.502023 | 0.735111 | 3.836445 |
| C | -4.908452 | 0.955180 | -1.069142 |
| C | -5.581214 | 0.889935 | -2.299036 |
| H | -6.233556 | 0.067020 | -2.557277 |
| C | -5.396455 | 2.009434 | -3.096318 |
| H | -5.869464 | 2.150662 | -4.059602 |
| C | -4.500936 | 2.953225 | -2.559399 |
| C | -4.075113 | 4.128635 | -3.209834 |
| C | -2.936743 | 4.896706 | -2.986716 |
| C | -2.641403 | 6.146392 | -3.587835 |
| H | -3.336013 | 6.633799 | -4.259753 |
| C | -1.411931 | 6.666342 | -3.241501 |
| H | -1.024290 | 7.599570 | -3.626873 |
| C | -0.664252 | 5.824886 | -2.391837 |
| C | 0.658924 | 5.994640 | -1.928787 |
| C | 1.406255 | 7.185997 | -1.823143 |
| H | 1.018205 | 8.147413 | -2.131032 |
| C | 2.636009 | 7.012770 | -1.223479 |
| H | 3.330509 | 7.818934 | -1.025247 |
| C | 2.931983 | 5.670318 | -0.876278 |
| C | 4.071038 | 5.227888 | -0.211069 |
| S | -1.577800 | -3.907751 | -2.817478 |
| S | -3.971629 | -2.055813 | -1.633997 |
| S | -3.974224 | -0.388683 | 2.593717 |
| S | -3.975371 | 2.440171 | -0.963650 |
| S | -1.583582 | 4.393418 | -1.973999 |
| S | 1.578907 | 4.631883 | -1.324349 |
| C | 0.666212 | -4.669088 | -4.224394 |
| C | 1.414584 | -5.173134 | -5.308304 |
| H | 1.027414 | -5.920808 | -5.987110 |
| C | 2.644126 | -4.566575 | -5.457370 |
| H | 3.339297 | -4.797741 | -6.254129 |
| C | 2.938843 | -3.594260 | -4.468402 |
| C | 4.077205 | -2.796076 | -4.417456 |
| C | 4.502456 | -1.910514 | -3.407047 |
| C | 5.398441 | -0.840942 | -3.591824 |
| H | 5.872230 | -0.647640 | -4.545636 |
| C | 5.582668 | -0.057814 | -2.462237 |
| H | 6.235259 | 0.803734 | -2.425973 |
| C | 4.908987 | -0.535697 | -1.327690 |
| C | 4.921223 | 0.002802 | 0.001020 |
| C | 4.907720 | 1.422679 | 0.199157 |
| C | 5.581074 | 2.166664 | 1.180232 |
| H | 6.234757 | 1.704905 | 1.907491 |
| C | 5.394988 | 3.536276 | 1.067705 |
| H | 5.868272 | 4.265917 | 1.712087 |
| C | 4.497858 | 3.910444 | 0.049786 |
| C | 4.908584 | -0.878789 | 1.131584 |
| C | 5.582394 | -2.100195 | 1.285153 |
| H | 6.235798 | -2.499105 | 0.521360 |
| C | 5.396938 | -2.687510 | 2.527652 |
| H | 5.870505 | -3.610290 | 2.837186 |
| C | 4.499984 | -1.993253 | 3.360940 |
| C | 4.073495 | -2.426447 | 4.632328 |
| C | 2.934238 | -2.072522 | 5.348132 |
| C | 2.638130 | -2.444528 | 6.683900 |
| H | 3.332779 | -3.019488 | 7.282575 |
| C | 1.407872 | -2.013248 | 7.133701 |
| H | 1.019548 | -2.228696 | 8.119828 |
| C | 0.660356 | -1.325691 | 6.155175 |
| C | -0.663415 | -0.841440 | 6.239603 |
| C | -1.412128 | -0.528703 | 7.393152 |
| H | -1.024832 | -0.662315 | 8.394087 |
| C | -2.642231 | 0.029793 | 7.116052 |
| H | -3.337770 | 0.365976 | 7.874155 |
| C | -2.937102 | 0.135072 | 5.733184 |
| C | -4.076340 | 0.710702 | 5.179672 |
| S | 1.584887 | -3.463531 | -3.346053 |
| S | 3.975703 | -1.968239 | -1.732219 |
| S | 3.972348 | 2.488635 | -0.838026 |
| S | 3.973690 | -0.513709 | 2.573691 |
| S | 1.580799 | -1.165469 | 4.673292 |
| S | -1.582491 | -0.487114 | 4.790939 |
| H | 4.701128 | 6.009553 | 0.205395 |
| H | 4.708016 | -2.826214 | -5.302118 |
| H | 4.703948 | -3.177824 | 5.100747 |
| H | -4.705251 | 4.456604 | -4.032523 |
| H | -4.701179 | -5.721741 | -1.847779 |
| H | -4.707708 | 1.257421 | 5.875322 |

$9^{3+}$
State = Q
Symmetry = $C_2$
E = -7481.99526731 a.u.

| | | | |
|---|---|---|---|
| C | 2.688437 | -1.175541 | 5.854547 |
| C | 2.359658 | -1.635579 | 7.138968 |
| H | 2.921726 | -1.359049 | 8.020725 |
| C | 1.210162 | -2.409354 | 7.170656 |
| H | 0.785891 | -2.821913 | 8.076793 |
| C | 0.623847 | -2.625516 | 5.907406 |
| C | -0.565434 | -3.327455 | 5.654133 |
| C | -1.108744 | -3.741897 | 4.439893 |
| C | -2.450283 | -4.161410 | 4.248688 |
| H | -3.148557 | -4.223905 | 5.073529 |
| C | -2.772153 | -4.457297 | 2.944187 |
| H | -3.746515 | -4.803008 | 2.628884 |
| C | -1.678686 | -4.370969 | 2.049552 |
| C | -1.645829 | -4.678484 | 0.666951 |
| C | -2.808929 | -4.511774 | -0.162819 |
| C | -3.223728 | -5.340033 | -1.213578 |

| | | | |
|---|---|---|---|
| H | -2.678921 | -6.230501 | -1.495111 |
| C | -4.425824 | -4.956351 | -1.788178 |
| H | -4.918434 | -5.502522 | -2.582236 |
| C | -4.965725 | -3.772159 | -1.254815 |
| C | -0.434896 | -5.200161 | 0.074400 |
| C | 0.392097 | -6.209899 | 0.581096 |
| H | 0.191042 | -6.700926 | 1.523356 |
| C | 1.419069 | -6.575818 | -0.278483 |
| H | 2.125207 | -7.370208 | -0.073662 |
| C | 1.484715 | -5.804527 | -1.452428 |
| C | 2.465932 | -5.940684 | -2.452016 |
| C | 2.873872 | -5.035483 | -3.429129 |
| C | 3.757190 | -5.310347 | -4.501186 |
| H | 4.155794 | -6.302053 | -4.671603 |
| C | 4.054403 | -4.215035 | -5.286372 |
| H | 4.729806 | -4.246575 | -6.130491 |
| C | 3.459658 | -3.020789 | -4.833145 |
| C | 3.658252 | -1.707656 | -5.313841 |
| C | 4.117336 | -1.295370 | -6.583387 |
| H | 4.381031 | -1.996449 | -7.363479 |
| C | 4.128199 | 0.072611 | -6.747118 |
| H | 4.420602 | 0.569526 | -7.663186 |
| C | 3.725309 | 0.802936 | -5.597893 |
| C | 3.625756 | 2.182878 | -5.497763 |
| S | 1.558155 | -1.797954 | 4.669220 |
| S | -0.240942 | -3.823027 | 2.907972 |
| S | -3.938258 | -3.179559 | 0.043349 |
| S | 0.153295 | -4.654299 | -1.485602 |
| S | 2.441593 | -3.326871 | -3.440016 |
| S | 3.301658 | -0.322486 | -4.304380 |
| C | 3.725705 | -0.291659 | 5.466539 |
| C | 4.943349 | -0.023676 | 6.120317 |
| H | 5.214225 | -0.485691 | 7.059884 |
| C | 5.777629 | 0.815276 | 5.408047 |
| H | 6.768663 | 1.101958 | 5.735588 |
| C | 5.231748 | 1.281870 | 4.187158 |
| C | 5.875766 | 2.088968 | 3.254339 |
| C | 5.357646 | 2.703779 | 2.098098 |
| C | 6.124173 | 3.154350 | 1.005432 |
| H | 7.204495 | 3.088513 | 0.997483 |
| C | 5.371247 | 3.648245 | -0.046427 |
| H | 5.796562 | 4.036618 | -0.961584 |
| C | 3.989766 | 3.686064 | 0.212668 |
| C | 2.952195 | 4.178825 | -0.636086 |
| C | 3.036165 | 3.966714 | -2.057830 |
| C | 2.665840 | 4.824883 | -3.097830 |
| H | 2.294381 | 5.824807 | -2.919905 |
| C | 2.894376 | 4.295989 | -4.365245 |
| H | 2.700964 | 4.830434 | -5.286365 |
| C | 3.376665 | 2.978584 | -4.354062 |
| C | -1.917519 | 5.893539 | 0.580512 |
| C | -2.556401 | 7.007146 | 1.124529 |
| H | -2.016416 | 7.859014 | 1.514525 |
| C | -3.944726 | 6.934785 | 1.057818 |
| H | -4.607142 | 7.715008 | 1.409719 |
| C | -4.430193 | 5.746332 | 0.491733 |
| C | -5.792278 | 5.425261 | 0.339531 |
| C | -6.410671 | 4.238102 | -0.029168 |
| C | -7.802425 | 4.085064 | -0.257758 |
| H | -8.490039 | 4.913945 | -0.150050 |
| C | -8.180611 | 2.815283 | -0.627703 |
| H | -9.203489 | 2.528776 | -0.828663 |
| C | -7.108322 | 1.897210 | -0.677294 |
| C | -7.170469 | 0.526445 | -0.999066 |
| C | -8.237536 | -0.153415 | -1.624435 |
| H | -9.161781 | 0.337135 | -1.896541 |
| C | -7.961601 | -1.471673 | -1.907581 |
| H | -8.648686 | -2.139103 | -2.411282 |
| C | -6.679442 | -1.903910 | -1.484770 |
| C | -6.158102 | -3.174598 | -1.701353 |
| S | 3.618567 | 0.602725 | 3.965652 |
| S | 3.654227 | 3.016814 | 1.805209 |
| S | 3.630298 | 2.446722 | -2.701628 |
| S | -3.082463 | 4.723820 | 0.007113 |
| S | -5.598123 | 2.691101 | -0.274514 |
| S | -5.825290 | -0.565014 | -0.717905 |
| H | 3.725183 | 2.730417 | -6.431376 |
| H | 6.939475 | 2.237976 | 3.420663 |
| H | -6.473227 | 6.244364 | 0.555651 |
| H | 3.014329 | -6.879072 | -2.432999 |
| H | -1.165106 | -3.551201 | 6.532461 |
| H | -6.760815 | -3.806062 | -2.348970 |
| C | -0.505331 | 5.656844 | 0.487323 |
| C | 0.477842 | 6.129075 | 1.354380 |
| S | 0.197012 | 4.657410 | -0.760305 |
| C | 1.756850 | 5.657494 | 1.055612 |
| H | 0.255536 | 6.768805 | 2.197556 |
| C | 1.808046 | 4.836223 | -0.072965 |
| H | 2.640314 | 5.927351 | 1.617317 |

$10^{3+}$
State = Q
Symmetry = $C_2$
E = -8033.88904462 a.u.

| | | | |
|---|---|---|---|
| C | 5.128959 | 3.749601 | -3.826457 |
| C | 5.152119 | 4.866056 | -4.685723 |
| H | 5.907231 | 4.999726 | -5.447914 |
| C | 4.094429 | 5.729972 | -4.498348 |
| H | 3.934630 | 6.626135 | -5.083601 |
| C | 3.212295 | 5.353804 | -3.458384 |
| C | 2.063351 | 6.056005 | -3.094541 |
| C | 1.181522 | 5.885535 | -2.020446 |
| C | -0.053445 | 6.555491 | -1.892123 |
| H | -0.396211 | 7.264578 | -2.634639 |
| C | -0.760472 | 6.231306 | -0.749644 |
| H | -1.711472 | 6.672364 | -0.486819 |
| C | -0.089266 | 5.336030 | 0.101288 |
| C | -0.515080 | 4.862911 | 1.383267 |
| C | -1.926311 | 4.726449 | 1.664820 |
| C | -2.584609 | 5.122936 | 2.834409 |

| | | | |
|---|---|---|---|
| H | -2.058386 | 5.561485 | 3.670728 |
| C | -3.963834 | 4.990779 | 2.774693 |
| H | -4.635502 | 5.296052 | 3.566637 |
| C | -4.435711 | 4.423791 | 1.577872 |
| C | 0.430885 | 4.598148 | 2.414389 |
| C | 1.638680 | 5.282236 | 2.681204 |
| H | 1.974731 | 6.111413 | 2.074844 |
| C | 2.273716 | 4.869310 | 3.830628 |
| H | 3.178834 | 5.319986 | 4.217235 |
| C | 1.646971 | 3.779587 | 4.486602 |
| C | 2.122186 | 3.163442 | 5.640339 |
| C | 1.762237 | 1.930520 | 6.215159 |
| C | 2.121910 | 1.473134 | 7.495335 |
| H | 2.693987 | 2.082944 | 8.182407 |
| C | 1.680236 | 0.188232 | 7.785133 |
| H | 1.889556 | -0.323422 | 8.714845 |
| C | 1.002766 | -0.420502 | 6.722298 |
| C | 0.524922 | -1.755243 | 6.619897 |
| C | 0.127257 | -2.631244 | 7.644134 |
| H | 0.151633 | -2.354889 | 8.689412 |
| C | -0.363304 | -3.837687 | 7.177370 |
| H | -0.746793 | -4.621580 | 7.817794 |
| C | -0.326616 | -3.975921 | 5.770167 |
| C | -0.786754 | -5.066322 | 5.035503 |
| S | 3.760616 | 3.846195 | -2.731124 |
| S | 1.441502 | 4.850098 | -0.620984 |
| S | -3.083226 | 4.099032 | 0.498996 |
| S | 0.169796 | 3.345056 | 3.623981 |
| S | 0.861864 | 0.681487 | 5.367899 |
| S | 0.335743 | -2.506487 | 5.052330 |
| C | 6.015895 | 2.653026 | -3.827951 |
| C | 7.277393 | 2.572224 | -4.456966 |
| H | 7.703555 | 3.393806 | -5.015652 |
| C | 7.922134 | 1.374795 | -4.248119 |
| H | 8.904701 | 1.140855 | -4.637113 |
| C | 7.187444 | 0.441154 | -3.473298 |
| C | 7.621962 | -0.834894 | -3.143135 |
| C | 6.961065 | -1.893732 | -2.491567 |
| C | 7.574578 | -3.077397 | -2.056574 |
| H | 8.629835 | -3.265153 | -2.206692 |
| C | 6.710022 | -3.968078 | -1.425967 |
| H | 7.012821 | -4.935657 | -1.049896 |
| C | 5.390886 | -3.520031 | -1.379589 |
| C | 0.549640 | -5.266893 | -0.079914 |
| C | -0.035151 | -5.454797 | 1.210316 |
| C | -1.113965 | -6.280961 | 1.569046 |
| H | -1.608773 | -6.925838 | 0.856050 |
| C | -1.415797 | -6.249736 | 2.921131 |
| H | -2.190771 | -6.849023 | 3.381373 |
| C | -0.638158 | -5.338982 | 3.662496 |
| C | -3.285532 | -4.406441 | -3.629577 |
| C | -3.828479 | -4.919739 | -4.806254 |
| H | -3.407645 | -5.771468 | -5.322808 |
| C | -4.990075 | -4.265807 | -5.209598 |
| H | -5.562768 | -4.539758 | -6.086184 |
| C | -5.371229 | -3.205357 | -4.373834 |
| C | -6.502029 | -2.389267 | -4.562456 |
| C | -6.912104 | -1.242171 | -3.894920 |
| C | -8.159651 | -0.594846 | -4.081626 |
| H | -8.884570 | -0.955199 | -4.800049 |
| C | -8.359341 | 0.498507 | -3.269593 |
| H | -9.253155 | 1.106346 | -3.290274 |
| C | -7.262520 | 0.786610 | -2.428967 |
| C | -7.155463 | 1.833664 | -1.489423 |
| C | -8.212755 | 2.587550 | -0.940211 |
| H | -9.242485 | 2.451267 | -1.240315 |
| C | -7.815585 | 3.450044 | 0.057242 |
| H | -8.493343 | 4.082273 | 0.615943 |
| C | -6.421728 | 3.449298 | 0.308240 |
| C | -5.794925 | 4.202947 | 1.295773 |
| S | 5.647142 | 1.157191 | -2.992250 |
| S | 5.243741 | -1.938692 | -2.108688 |
| S | 0.558073 | -4.586360 | 2.619310 |
| S | -4.232240 | -3.061501 | -3.040664 |
| S | -5.977399 | -0.379311 | -2.671145 |
| S | -5.625873 | 2.297227 | -0.763745 |
| H | -1.363990 | -5.798065 | 5.594691 |
| H | 8.646451 | -1.055318 | -3.431548 |
| H | -7.166655 | -2.716284 | -5.357840 |
| H | 2.910630 | 3.700267 | 6.161295 |
| H | 1.800943 | 6.868780 | -3.766685 |
| H | -6.477210 | 4.695574 | 1.983887 |
| C | -2.108802 | -4.843685 | -2.933378 |
| C | -0.985908 | -5.462395 | -3.475858 |
| S | -1.903425 | -4.582036 | -1.219884 |
| C | 0.037097 | -5.673882 | -2.546924 |
| H | -0.905525 | -5.715133 | -4.524346 |
| C | -0.279295 | -5.257298 | -1.253790 |
| H | 0.979148 | -6.144921 | -2.791036 |
| C | 4.256681 | -4.201196 | -0.823659 |
| C | 4.263136 | -5.121437 | 0.221685 |
| S | 2.631043 | -3.931354 | -1.400733 |
| C | 2.988861 | -5.564225 | 0.581687 |
| H | 5.170007 | -5.431294 | 0.722818 |
| C | 1.961421 | -5.027037 | -0.195665 |
| H | 2.806256 | -6.286911 | 1.364800 |

$11^{3+}$
State = Q
Symmetry = $D_3$
E = -8585.78045109 a.u.

| | | | |
|---|---|---|---|
| C | 6.566732 | -3.151264 | -2.325257 |
| C | 6.941052 | -4.176767 | -3.210089 |
| H | 7.880123 | -4.706019 | -3.125248 |
| C | 5.970550 | -4.482854 | -4.144597 |
| H | 6.071329 | -5.263752 | -4.886991 |
| C | 4.811248 | -3.682093 | -4.060812 |
| C | 3.689316 | -3.812893 | -4.884814 |
| C | 2.558107 | -3.007422 | -5.041017 |
| C | 1.425214 | -3.372729 | -5.800214 |

| | | | |
|---|---|---|---|
| H | 1.365563 | -4.325799 | -6.309842 |
| C | 0.435139 | -2.411218 | -5.844709 |
| H | -0.479012 | -2.519147 | -6.410576 |
| C | 0.761892 | -1.221126 | -5.170043 |
| C | -0.000085 | -0.006799 | -5.134573 |
| C | -1.432660 | -0.059633 | -5.171675 |
| C | -2.299148 | 0.816971 | -5.849179 |
| H | -1.934865 | 1.661475 | -6.416318 |
| C | -3.626904 | 0.440249 | -5.805624 |
| H | -4.421861 | 0.967338 | -6.317328 |
| C | -3.877849 | -0.721990 | -5.044375 |
| C | 0.670435 | 1.260223 | -5.172877 |
| C | 1.863896 | 1.571251 | -5.849036 |
| H | 2.414131 | 0.832596 | -6.413972 |
| C | 2.201271 | 2.909593 | -5.807382 |
| H | 3.055965 | 3.333689 | -6.318520 |
| C | 1.318906 | 3.709314 | -5.048977 |
| C | 1.450699 | 5.092021 | -4.895556 |
| C | 0.777292 | 5.999716 | -4.072463 |
| C | 0.890726 | 7.403927 | -4.159174 |
| H | 1.515446 | 7.880317 | -4.903408 |
| C | 0.141703 | 8.093068 | -3.224865 |
| H | 0.130470 | 9.171120 | -3.142123 |
| C | -0.557774 | 7.257765 | -2.337346 |
| C | -1.322509 | 7.653608 | -1.214668 |
| C | -1.862014 | 8.927586 | -0.950145 |
| H | -1.752543 | 9.759745 | -1.631587 |
| C | -2.577785 | 8.990147 | 0.227144 |
| H | -3.077541 | 9.881458 | 0.583986 |
| C | -2.603790 | 7.779562 | 0.959553 |
| C | -3.228439 | 7.600667 | 2.189910 |
| S | 4.977305 | -2.532243 | -2.738037 |
| S | 2.338184 | -1.380983 | -4.398854 |
| S | -2.360114 | -1.343273 | -4.398988 |
| S | 0.021124 | 2.706540 | -4.403771 |
| S | -0.299490 | 5.571008 | -2.747234 |
| S | -1.711054 | 6.538492 | 0.077753 |
| C | 7.290940 | -2.684427 | -1.202996 |
| C | 8.663753 | -2.853678 | -0.936913 |
| H | 9.330221 | -3.366197 | -1.616609 |
| C | 9.074853 | -2.262417 | 0.239375 |
| H | 10.096314 | -2.274576 | 0.597144 |
| C | 8.038849 | -1.632956 | 0.969525 |
| C | 8.195181 | -1.000051 | 2.198721 |
| C | 7.239891 | -0.476847 | 3.085313 |
| C | 7.526442 | 0.274605 | 4.236926 |
| H | 8.541738 | 0.507986 | 4.530487 |
| C | 6.402061 | 0.676205 | 4.949837 |
| H | 6.444586 | 1.239937 | 5.871604 |
| C | 5.202914 | 0.225949 | 4.394304 |
| C | 0.001116 | 0.006536 | 5.375740 |
| C | -2.790987 | 4.400096 | 4.390378 |
| C | -3.779884 | 5.214296 | 4.945779 |
| H | -4.288230 | 4.970718 | 5.868554 |
| C | -3.995248 | 6.387636 | 4.231200 |

| | | | |
|---|---|---|---|
| H | -4.704739 | 7.150629 | 4.524348 |
| C | -3.202656 | 6.513327 | 3.078365 |
| C | -2.410498 | -4.609149 | 4.401116 |
| C | -2.621548 | -5.871311 | 4.959412 |
| H | -2.155705 | -6.188166 | 5.882363 |
| C | -3.531472 | -6.645258 | 4.247517 |
| H | -3.838010 | -7.640295 | 4.543164 |
| C | -4.037484 | -6.023713 | 3.093968 |
| C | -4.967874 | -6.590772 | 2.207871 |
| C | -5.436321 | -6.141474 | 0.977165 |
| C | -6.499109 | -6.724979 | 0.247346 |
| H | -7.021379 | -7.602246 | 0.606739 |
| C | -6.803828 | -6.076075 | -0.930911 |
| H | -7.580430 | -6.398116 | -1.610633 |
| C | -5.969792 | -4.973053 | -1.198828 |
| C | -6.010349 | -4.115170 | -2.323234 |
| C | -7.084608 | -3.928365 | -3.209660 |
| H | -8.012777 | -4.476459 | -3.124464 |
| C | -6.863110 | -2.937196 | -4.146342 |
| H | -7.588919 | -2.635551 | -4.890190 |
| C | -5.589913 | -2.333726 | -4.062587 |
| C | -5.141193 | -1.298591 | -4.888396 |
| S | 6.518458 | -1.787314 | 0.086678 |
| S | 5.498017 | -0.684204 | 2.932454 |
| S | -2.152139 | 5.108387 | 2.926589 |
| S | -3.344772 | -4.412410 | 2.937934 |
| S | -4.807875 | -4.750214 | 0.091569 |
| S | -4.678744 | -3.049637 | -2.737157 |
| H | -3.830746 | 8.442206 | 2.522230 |
| H | 9.224832 | -0.898673 | 2.531796 |
| H | -5.395896 | -7.532083 | 2.542727 |
| H | 2.213377 | 5.541429 | -5.526230 |
| H | 3.697669 | -4.699333 | -5.513741 |
| H | -5.912307 | -0.863936 | -5.519157 |
| C | -1.567164 | -3.559262 | 4.888432 |
| C | -0.434452 | -3.684693 | 5.690161 |
| S | -1.851377 | -1.884565 | 4.480259 |
| C | 0.214521 | -2.472539 | 5.932482 |
| H | -0.079914 | -4.637929 | 6.058060 |
| C | -0.413753 | -1.366845 | 5.354710 |
| H | 1.102693 | -2.381439 | 6.542019 |
| C | -2.303245 | 3.146070 | 4.880738 |
| C | -2.978769 | 2.228924 | 5.683264 |
| S | -0.709883 | 2.555066 | 4.475840 |
| C | -2.253262 | 1.061586 | 5.928470 |
| H | -3.982159 | 2.398598 | 6.049501 |
| C | -0.980896 | 1.052375 | 5.352175 |
| H | -2.618903 | 0.247636 | 6.538783 |
| C | 3.872635 | 0.431433 | 4.883271 |
| C | 3.415533 | 1.476349 | 5.683730 |
| S | 2.564385 | -0.653534 | 4.479057 |
| C | 2.041636 | 1.432150 | 5.927934 |
| H | 4.063913 | 2.261021 | 6.049243 |
| C | 1.397882 | 0.333993 | 5.352842 |
| H | 1.519101 | 2.156750 | 6.536702 |

**14**

State = S0

Symmetry = $T_D$

E = -1065.96552438 a.u.

| | | | |
|---|---|---|---|
| C | 1.255303 | 1.255303 | -2.948287 |
| C | 0.491684 | 0.491684 | -3.544479 |
| C | -0.491684 | -0.491684 | -3.544479 |
| C | -1.255303 | -1.255303 | -2.948287 |
| C | -1.255303 | 2.948287 | 1.255303 |
| C | -0.491684 | 3.544479 | 0.491684 |
| C | 0.491684 | 3.544479 | -0.491684 |
| C | 1.255303 | 2.948287 | -1.255303 |
| C | 1.255303 | -2.948287 | 1.255303 |
| C | 0.491684 | -3.544479 | 0.491684 |
| C | -0.491684 | -3.544479 | -0.491684 |
| C | -1.255303 | -2.948287 | -1.255303 |
| C | -2.948287 | 1.255303 | 1.255303 |
| C | -3.544479 | 0.491684 | 0.491684 |
| C | -3.544479 | -0.491684 | -0.491684 |
| C | -2.948287 | -1.255303 | -1.255303 |
| C | 1.255303 | -1.255303 | 2.948287 |
| C | 0.491684 | -0.491684 | 3.544479 |
| C | -0.491684 | 0.491684 | 3.544479 |
| C | -1.255303 | 1.255303 | 2.948287 |
| C | 2.948287 | 1.255303 | -1.255303 |
| C | 3.544479 | 0.491684 | -0.491684 |
| C | 3.544479 | -0.491684 | 0.491684 |
| C | 2.948287 | -1.255303 | 1.255303 |
| C | 2.021882 | -2.021882 | 2.021882 |
| C | -2.021882 | 2.021882 | 2.021882 |
| C | -2.021882 | -2.021882 | -2.021882 |
| C | 2.021882 | 2.021882 | -2.021882 |

**15**

State = S0

Symmetry = $T_D$

E = -1523.31619852 a.u.

| | | | |
|---|---|---|---|
| C | -2.071264 | 3.775879 | -2.071264 |
| C | 0.434673 | 4.809887 | 0.434673 |
| C | 1.378645 | 4.525598 | 1.378645 |
| C | 2.071264 | 3.775879 | 2.071264 |
| C | 2.071264 | -2.071264 | -3.775879 |
| C | 1.378645 | -1.378645 | -4.525598 |
| C | 0.434673 | -0.434673 | -4.809887 |
| C | -2.071264 | 2.071264 | -3.775879 |
| C | 0.434673 | 0.434673 | 4.809887 |
| C | 1.378645 | 1.378645 | 4.525598 |
| C | 2.071264 | 2.071264 | 3.775879 |
| C | 3.775879 | -2.071264 | -2.071264 |
| C | 4.525598 | -1.378645 | -1.378645 |
| C | 4.809887 | -0.434673 | -0.434673 |
| C | 3.775879 | 2.071264 | 2.071264 |
| C | -2.071264 | -3.775879 | 2.071264 |
| C | 0.434673 | -4.809887 | -0.434673 |
| C | 1.378645 | -4.525598 | -1.378645 |
| C | 2.071264 | -3.775879 | -2.071264 |
| C | -3.775879 | 2.071264 | -2.071264 |
| C | -4.525598 | 1.378645 | -1.378645 |
| C | -4.809887 | 0.434673 | -0.434673 |
| C | -3.775879 | -2.071264 | 2.071264 |
| C | -1.378645 | 4.525598 | -1.378645 |
| C | -0.434673 | 4.809887 | -0.434673 |
| C | 4.525598 | 1.378645 | 1.378645 |
| C | 4.809887 | 0.434673 | 0.434673 |
| C | -0.434673 | -0.434673 | 4.809887 |
| C | -1.378645 | -1.378645 | 4.525598 |
| C | -4.809887 | -0.434673 | 0.434673 |
| C | -4.525598 | -1.378645 | 1.378645 |
| C | -1.378645 | 1.378645 | -4.525598 |
| C | -0.434673 | 0.434673 | -4.809887 |
| C | -1.378645 | -4.525598 | 1.378645 |
| C | -0.434673 | -4.809887 | 0.434673 |
| C | -2.071264 | -2.071264 | 3.775879 |
| C | 2.778053 | 2.778053 | 2.778053 |
| C | -2.778053 | -2.778053 | 2.778053 |
| C | -2.778053 | 2.778053 | -2.778053 |
| C | 2.778053 | -2.778053 | -2.778053 |

**16**

State = S0

Symmetry = $T_D$

E = -1980.57617953 a.u.

| | | | |
|---|---|---|---|
| C | -2.879023 | 4.587034 | -2.879023 |
| C | -2.210739 | 5.378773 | -2.210739 |
| C | -1.324302 | 5.873372 | -1.324302 |
| C | 1.324302 | 5.873372 | 1.324302 |
| C | 2.210739 | 5.378773 | 2.210739 |
| C | 2.879023 | 4.587034 | 2.879023 |
| C | 2.879023 | -2.879023 | -4.587034 |
| C | 2.210739 | -2.210739 | -5.378773 |
| C | 1.324302 | -1.324302 | -5.873372 |
| C | -1.324302 | 1.324302 | -5.873372 |
| C | -2.210739 | 2.210739 | -5.378773 |
| C | -2.879023 | 2.879023 | -4.587034 |
| C | 1.324302 | 1.324302 | 5.873372 |
| C | 2.210739 | 2.210739 | 5.378773 |
| C | 2.879023 | 2.879023 | 4.587034 |
| C | 4.587034 | -2.879023 | -2.879023 |
| C | 5.378773 | -2.210739 | -2.210739 |
| C | 5.873372 | -1.324302 | -1.324302 |
| C | 5.873372 | 1.324302 | 1.324302 |
| C | 4.587034 | 2.879023 | 2.879023 |
| C | 5.378773 | 2.210739 | 2.210739 |
| C | -2.879023 | -4.587034 | 2.879023 |
| C | -2.210739 | -5.378773 | 2.210739 |
| C | -1.324302 | -5.873372 | 1.324302 |
| C | 1.324302 | -5.873372 | -1.324302 |
| C | 2.210739 | -5.378773 | -2.210739 |
| C | 2.879023 | -4.587034 | -2.879023 |
| C | -4.587034 | 2.879023 | -2.879023 |

| | | | |
|---|---|---|---|
| C | -5.378773 | 2.210739 | -2.210739 |
| C | -5.873372 | 1.324302 | -1.324302 |
| C | -5.873372 | -1.324302 | 1.324302 |
| C | -5.378773 | -2.210739 | 2.210739 |
| C | -4.587034 | -2.879023 | 2.879023 |
| C | -0.475952 | 6.165163 | -0.475952 |
| C | 0.475952 | 6.165163 | 0.475952 |
| C | 6.165163 | 0.475952 | 0.475952 |
| C | 6.165163 | -0.475952 | -0.475952 |
| C | 0.475952 | 0.475952 | 6.165163 |
| C | -0.475952 | -0.475952 | 6.165163 |
| C | -6.165163 | 0.475952 | -0.475952 |
| C | -6.165163 | -0.475952 | 0.475952 |
| C | -0.475952 | 0.475952 | -6.165163 |
| C | 0.475952 | -0.475952 | -6.165163 |
| C | -0.475952 | -6.165163 | 0.475952 |
| C | 0.475952 | -6.165163 | -0.475952 |
| C | -1.324302 | -1.324302 | 5.873372 |
| C | -2.210739 | -2.210739 | 5.378773 |
| C | -2.879023 | -2.879023 | 4.587034 |
| C | 3.549344 | 3.549344 | 3.549344 |
| C | -3.549344 | -3.549344 | 3.549344 |
| C | -3.549344 | 3.549344 | -3.549344 |
| C | 3.549344 | -3.549344 | -3.549344 |

**17**

State = $S_0$

Symmetry = $O_H$

E = -2132.84884214 a.u.

| | | | |
|---|---|---|---|
| C | 3.078031 | 3.078031 | 3.078031 |
| C | 3.562974 | 3.562974 | 1.863114 |
| C | 3.763737 | 3.763737 | 0.667656 |
| C | 3.763737 | 3.763737 | -0.667656 |
| C | 3.562974 | 3.562974 | -1.863114 |
| C | 1.863114 | 3.562974 | 3.562974 |
| C | 0.667656 | 3.763737 | 3.763737 |
| C | -0.667656 | 3.763737 | 3.763737 |
| C | -1.863114 | 3.562974 | 3.562974 |
| C | 3.562974 | 1.863114 | 3.562974 |
| C | 3.763737 | 0.667656 | 3.763737 |
| C | 3.763737 | -0.667656 | 3.763737 |
| C | 3.562974 | -1.863114 | 3.562974 |
| C | -3.078031 | 3.078031 | 3.078031 |
| C | 3.078031 | 3.078031 | -3.078031 |
| C | 3.078031 | -3.078031 | 3.078031 |
| C | 1.863114 | 3.562974 | -3.562974 |
| C | 0.667656 | 3.763737 | -3.763737 |
| C | -0.667656 | 3.763737 | -3.763737 |
| C | -1.863114 | 3.562974 | -3.562974 |
| C | 3.562974 | 1.863114 | -3.562974 |
| C | 3.763737 | 0.667656 | -3.763737 |
| C | 3.763737 | -0.667656 | -3.763737 |
| C | 3.562974 | -1.863114 | -3.562974 |
| C | -3.562974 | 3.562974 | 1.863114 |
| C | -3.763737 | 3.763737 | 0.667656 |
| C | -3.763737 | 3.763737 | -0.667656 |
| C | -3.562974 | 3.562974 | -1.863114 |
| C | -3.562974 | 1.863114 | 3.562974 |
| C | -3.763737 | 0.667656 | 3.763737 |
| C | -3.763737 | -0.667656 | 3.763737 |
| C | -3.562974 | -1.863114 | 3.562974 |
| C | 3.562974 | -3.562974 | 1.863114 |
| C | 3.763737 | -3.763737 | 0.667656 |
| C | 3.763737 | -3.763737 | -0.667656 |
| C | 3.562974 | -3.562974 | -1.863114 |
| C | 3.078031 | -3.078031 | -3.078031 |
| C | -3.078031 | 3.078031 | -3.078031 |
| C | 1.863114 | -3.562974 | 3.562974 |
| C | 0.667656 | -3.763737 | 3.763737 |
| C | -0.667656 | -3.763737 | 3.763737 |
| C | -1.863114 | -3.562974 | 3.562974 |
| C | -3.078031 | -3.078031 | 3.078031 |
| C | 1.863114 | -3.562974 | -3.562974 |
| C | 0.667656 | -3.763737 | -3.763737 |
| C | -0.667656 | -3.763737 | -3.763737 |
| C | -1.863114 | -3.562974 | -3.562974 |
| C | -3.562974 | 1.863114 | -3.562974 |
| C | -3.763737 | 0.667656 | -3.763737 |
| C | -3.763737 | -0.667656 | -3.763737 |
| C | -3.562974 | -1.863114 | -3.562974 |
| C | -3.562974 | -3.562974 | 1.863114 |
| C | -3.763737 | -3.763737 | 0.667656 |
| C | -3.763737 | -3.763737 | -0.667656 |
| C | -3.562974 | -3.562974 | -1.863114 |
| C | -3.078031 | -3.078031 | -3.078031 |

**18**

State = 1

Symmetry = $O_H$

E = -3047.02011833 a.u.

| | | | |
|---|---|---|---|
| C | 4.281349 | 4.281349 | 4.281349 |
| C | 4.789408 | 4.789408 | 3.086795 |
| C | 5.078314 | 5.078314 | 1.927170 |
| C | 5.239408 | 5.239408 | 0.617076 |
| C | 5.239408 | 5.239408 | -0.617076 |
| C | 5.078314 | 5.078314 | -1.927170 |
| C | 4.789408 | 4.789408 | -3.086795 |
| C | 3.086795 | 4.789408 | 4.789408 |
| C | 1.927170 | 5.078314 | 5.078314 |
| C | 0.617076 | 5.239408 | 5.239408 |
| C | -0.617076 | 5.239408 | 5.239408 |
| C | -1.927170 | 5.078314 | 5.078314 |
| C | -3.086795 | 4.789408 | 4.789408 |
| C | 4.789408 | 3.086795 | 4.789408 |
| C | 5.078314 | 1.927170 | 5.078314 |
| C | 5.239408 | 0.617076 | 5.239408 |
| C | 5.239408 | -0.617076 | 5.239408 |
| C | 5.078314 | -1.927170 | 5.078314 |
| C | 4.789408 | -3.086795 | 4.789408 |

| | | | |
|---|---|---|---|
| C | -4.281349 | 4.281349 | 4.281349 |
| C | 4.281349 | 4.281349 | -4.281349 |
| C | 4.281349 | -4.281349 | 4.281349 |
| C | 3.086795 | 4.789408 | -4.789408 |
| C | 1.927170 | 5.078314 | -5.078314 |
| C | 0.617076 | 5.239408 | -5.239408 |
| C | -0.617076 | 5.239408 | -5.239408 |
| C | -1.927170 | 5.078314 | -5.078314 |
| C | -3.086795 | 4.789408 | -4.789408 |
| C | 4.789408 | 3.086795 | -4.789408 |
| C | 5.078314 | 1.927170 | -5.078314 |
| C | 5.239408 | 0.617076 | -5.239408 |
| C | 5.239408 | -0.617076 | -5.239408 |
| C | 5.078314 | -1.927170 | -5.078314 |
| C | 4.789408 | -3.086795 | -4.789408 |
| C | -4.789408 | 4.789408 | 3.086795 |
| C | -5.078314 | 5.078314 | 1.927170 |
| C | -5.239408 | 5.239408 | 0.617076 |
| C | -5.239408 | 5.239408 | -0.617076 |
| C | -5.078314 | 5.078314 | -1.927170 |
| C | -4.789408 | 4.789408 | -3.086795 |
| C | -4.789408 | 3.086795 | 4.789408 |
| C | -5.078314 | 1.927170 | 5.078314 |
| C | -5.239408 | 0.617076 | 5.239408 |
| C | -5.239408 | -0.617076 | 5.239408 |
| C | -5.078314 | -1.927170 | 5.078314 |
| C | -4.789408 | -3.086795 | 4.789408 |
| C | 4.789408 | -4.789408 | 3.086795 |
| C | 5.078314 | -5.078314 | 1.927170 |
| C | 5.239408 | -5.239408 | 0.617076 |
| C | 5.239408 | -5.239408 | -0.617076 |
| C | 5.078314 | -5.078314 | -1.927170 |
| C | 4.789408 | -4.789408 | -3.086795 |
| C | 4.281349 | -4.281349 | -4.281349 |
| C | -4.281349 | 4.281349 | -4.281349 |
| C | 3.086795 | -4.789408 | 4.789408 |
| C | 1.927170 | -5.078314 | 5.078314 |
| C | 0.617076 | -5.239408 | 5.239408 |
| C | -0.617076 | -5.239408 | 5.239408 |
| C | -1.927170 | -5.078314 | 5.078314 |
| C | -3.086795 | -4.789408 | 4.789408 |
| C | -4.281349 | -4.281349 | 4.281349 |
| C | 3.086795 | -4.789408 | -4.789408 |
| C | 1.927170 | -5.078314 | -5.078314 |
| C | 0.617076 | -5.239408 | -5.239408 |
| C | -0.617076 | -5.239408 | -5.239408 |
| C | -1.927170 | -5.078314 | -5.078314 |
| C | -3.086795 | -4.789408 | -4.789408 |
| C | -4.789408 | 3.086795 | -4.789408 |
| C | -5.078314 | 1.927170 | -5.078314 |
| C | -5.239408 | 0.617076 | -5.239408 |
| C | -5.239408 | -0.617076 | -5.239408 |
| C | -5.078314 | -1.927170 | -5.078314 |
| C | -4.789408 | -3.086795 | -4.789408 |
| C | -4.789408 | -4.789408 | 3.086795 |
| C | -5.078314 | -5.078314 | 1.927170 |
| C | -5.239408 | -5.239408 | 0.617076 |
| C | -5.239408 | -5.239408 | -0.617076 |
| C | -5.078314 | -5.078314 | -1.927170 |
| C | -4.789408 | -4.789408 | -3.086795 |
| C | -4.281349 | -4.281349 | -4.281349 |

**19**

State = $Q_1$

Symmetry = $O_H$

E = -2131.82556431 a.u.

| | | | |
|---|---|---|---|
| C | 3.072090 | 3.072090 | 3.072090 |
| C | 3.563466 | 3.563466 | 1.861845 |
| C | 3.765958 | 3.765958 | 0.667595 |
| C | 3.765958 | 3.765958 | -0.667595 |
| C | 3.563466 | 3.563466 | -1.861845 |
| C | 1.861845 | 3.563466 | 3.563466 |
| C | 0.667595 | 3.765958 | 3.765958 |
| C | -0.667595 | 3.765958 | 3.765958 |
| C | -1.861845 | 3.563466 | 3.563466 |
| C | 3.563466 | 1.861845 | 3.563466 |
| C | 3.765958 | 0.667595 | 3.765958 |
| C | 3.765958 | -0.667595 | 3.765958 |
| C | 3.563466 | -1.861845 | 3.563466 |
| C | -3.072090 | 3.072090 | 3.072090 |
| C | 3.072090 | 3.072090 | -3.072090 |
| C | 3.072090 | -3.072090 | 3.072090 |
| C | 1.861845 | 3.563466 | -3.563466 |
| C | 0.667595 | 3.765958 | -3.765958 |
| C | -0.667595 | 3.765958 | -3.765958 |
| C | -1.861845 | 3.563466 | -3.563466 |
| C | 3.563466 | 1.861845 | -3.563466 |
| C | 3.765958 | 0.667595 | -3.765958 |
| C | 3.765958 | -0.667595 | -3.765958 |
| C | 3.563466 | -1.861845 | -3.563466 |
| C | -3.563466 | 3.563466 | 1.861845 |
| C | -3.765958 | 3.765958 | 0.667595 |
| C | -3.765958 | 3.765958 | -0.667595 |
| C | -3.563466 | 3.563466 | -1.861845 |
| C | -3.563466 | 1.861845 | 3.563466 |
| C | -3.765958 | 0.667595 | 3.765958 |
| C | -3.765958 | -0.667595 | 3.765958 |
| C | -3.563466 | -1.861845 | 3.563466 |
| C | 3.563466 | -3.563466 | 1.861845 |
| C | 3.765958 | -3.765958 | 0.667595 |
| C | 3.765958 | -3.765958 | -0.667595 |
| C | 3.563466 | -3.563466 | -1.861845 |
| C | 3.072090 | -3.072090 | -3.072090 |
| C | -3.072090 | 3.072090 | -3.072090 |
| C | 1.861845 | -3.563466 | 3.563466 |
| C | 0.667595 | -3.765958 | 3.765958 |
| C | -0.667595 | -3.765958 | 3.765958 |
| C | -1.861845 | -3.563466 | 3.563466 |
| C | -3.072090 | -3.072090 | 3.072090 |
| C | 1.861845 | -3.563466 | -3.563466 |

| | | | |
|---|---|---|---|
| C | 0.667595 | -3.765958 | -3.765958 |
| C | -0.667595 | -3.765958 | -3.765958 |
| C | -1.861845 | -3.563466 | -3.563466 |
| C | -3.563466 | 1.861845 | -3.563466 |
| C | -3.765958 | 0.667595 | -3.765958 |
| C | -3.765958 | -0.667595 | -3.765958 |
| C | -3.563466 | -1.861845 | -3.563466 |
| C | -3.563466 | -3.563466 | 1.861845 |
| C | -3.765958 | -3.765958 | 0.667595 |
| C | -3.765958 | -3.765958 | -0.667595 |
| C | -3.563466 | -3.563466 | -1.861845 |
| C | -3.072090 | -3.072090 | -3.072090 |

**20**

State = $Q_1$

Symmetry = $O_H$

E = -3046.09741861 a.u.

| | | | |
|---|---|---|---|
| C | 4.275871 | 4.275871 | 4.275871 |
| C | 4.788398 | 4.788398 | 3.085387 |
| C | 5.075911 | 5.075911 | 1.925713 |
| C | 5.240297 | 5.240297 | 0.616615 |
| C | 5.240297 | 5.240297 | -0.616615 |
| C | 5.075911 | 5.075911 | -1.925713 |
| C | 4.788398 | 4.788398 | -3.085387 |
| C | 3.085387 | 4.788398 | 4.788398 |
| C | 1.925713 | 5.075911 | 5.075911 |
| C | 0.616615 | 5.240297 | 5.240297 |
| C | -0.616615 | 5.240297 | 5.240297 |
| C | -1.925713 | 5.075911 | 5.075911 |
| C | -3.085387 | 4.788398 | 4.788398 |
| C | 4.788398 | 3.085387 | 4.788398 |
| C | 5.075911 | 1.925713 | 5.075911 |
| C | 5.240297 | 0.616615 | 5.240297 |
| C | 5.240297 | -0.616615 | 5.240297 |
| C | 5.075911 | -1.925713 | 5.075911 |
| C | 4.788398 | -3.085387 | 4.788398 |
| C | -4.275871 | 4.275871 | 4.275871 |
| C | 4.275871 | 4.275871 | -4.275871 |
| C | 4.275871 | -4.275871 | 4.275871 |
| C | 3.085387 | 4.788398 | -4.788398 |
| C | 1.925713 | 5.075911 | -5.075911 |
| C | 0.616615 | 5.240297 | -5.240297 |
| C | -0.616615 | 5.240297 | -5.240297 |
| C | -1.925713 | 5.075911 | -5.075911 |
| C | -3.085387 | 4.788398 | -4.788398 |
| C | 4.788398 | 3.085387 | -4.788398 |
| C | 5.075911 | 1.925713 | -5.075911 |
| C | 5.240297 | 0.616615 | -5.240297 |
| C | 5.240297 | -0.616615 | -5.240297 |
| C | 5.075911 | -1.925713 | -5.075911 |
| C | 4.788398 | -3.085387 | -4.788398 |
| C | -4.788398 | 4.788398 | 3.085387 |
| C | -5.075911 | 5.075911 | 1.925713 |
| C | -5.240297 | 5.240297 | 0.616615 |
| C | -5.240297 | 5.240297 | -0.616615 |
| C | -5.075911 | 5.075911 | -1.925713 |
| C | -4.788398 | 4.788398 | -3.085387 |
| C | -4.788398 | 3.085387 | 4.788398 |
| C | -5.075911 | 1.925713 | 5.075911 |
| C | -5.240297 | 0.616615 | 5.240297 |
| C | -5.240297 | -0.616615 | 5.240297 |
| C | -5.075911 | -1.925713 | 5.075911 |
| C | -4.788398 | -3.085387 | 4.788398 |
| C | 4.788398 | -4.788398 | 3.085387 |
| C | 5.075911 | -5.075911 | 1.925713 |
| C | 5.240297 | -5.240297 | 0.616615 |
| C | 5.240297 | -5.240297 | -0.616615 |
| C | 5.075911 | -5.075911 | -1.925713 |
| C | 4.788398 | -4.788398 | -3.085387 |
| C | 4.275871 | -4.275871 | -4.275871 |
| C | -4.275871 | 4.275871 | -4.275871 |
| C | 3.085387 | -4.788398 | 4.788398 |
| C | 1.925713 | -5.075911 | 5.075911 |
| C | 0.616615 | -5.240297 | 5.240297 |
| C | -0.616615 | -5.240297 | 5.240297 |
| C | -1.925713 | -5.075911 | 5.075911 |
| C | -3.085387 | -4.788398 | 4.788398 |
| C | -4.275871 | -4.275871 | 4.275871 |
| C | 3.085387 | -4.788398 | -4.788398 |
| C | 1.925713 | -5.075911 | -5.075911 |
| C | 0.616615 | -5.240297 | -5.240297 |
| C | -0.616615 | -5.240297 | -5.240297 |
| C | -1.925713 | -5.075911 | -5.075911 |
| C | -3.085387 | -4.788398 | -4.788398 |
| C | -4.788398 | 3.085387 | -4.788398 |
| C | -5.075911 | 1.925713 | -5.075911 |
| C | -5.240297 | 0.616615 | -5.240297 |
| C | -5.240297 | -0.616615 | -5.240297 |
| C | -5.075911 | -1.925713 | -5.075911 |
| C | -4.788398 | -3.085387 | -4.788398 |
| C | -4.788398 | -4.788398 | 3.085387 |
| C | -5.075911 | -5.075911 | 1.925713 |
| C | -5.240297 | -5.240297 | 0.616615 |
| C | -5.240297 | -5.240297 | -0.616615 |
| C | -5.075911 | -5.075911 | -1.925713 |
| C | -4.788398 | -4.788398 | -3.085387 |
| C | -4.275871 | -4.275871 | -4.275871 |

**21**

State = S0

Symmetry = T

E = -2475.06260249 a.u.

| | | | |
|---|---|---|---|
| C | 4.162751 | 4.162751 | 4.162751 |
| C | 5.207245 | 3.227563 | 3.847953 |
| C | 3.097086 | 2.708654 | 5.970851 |
| C | 3.847953 | 5.207245 | 3.227563 |
| H | 5.314870 | 2.408540 | 4.556889 |
| C | 3.227563 | 3.847953 | 5.207245 |
| H | 3.848916 | 1.925420 | 5.901101 |

| | | | |
|---|---|---|---|
| H | 4.556889 | 5.314870 | 2.408540 |
| C | 1.888750 | 2.354189 | 6.640268 |
| H | 2.408540 | 4.556889 | 5.314870 |
| C | -4.162751 | -4.162751 | 4.162751 |
| C | -3.227563 | -3.847953 | 5.207245 |
| C | -3.847953 | -5.207245 | 3.227563 |
| C | -5.207245 | -3.227563 | 3.847953 |
| H | -2.408540 | -4.556889 | 5.314870 |
| H | -4.556889 | -5.314870 | 2.408540 |
| H | -5.314870 | -2.408540 | 4.556889 |
| C | 4.162751 | -4.162751 | -4.162751 |
| C | 3.847953 | -5.207245 | -3.227563 |
| C | 5.207245 | -3.227563 | -3.847953 |
| C | 3.227563 | -3.847953 | -5.207245 |
| C | 2.708654 | -5.970851 | -3.097086 |
| H | 4.556889 | -5.314870 | -2.408540 |
| C | 5.970851 | -3.097086 | -2.708654 |
| H | 5.314870 | -2.408540 | -4.556889 |
| C | 3.097086 | -2.708654 | -5.970851 |
| H | 2.408540 | -4.556889 | -5.314870 |
| C | 2.354189 | -6.640268 | -1.888750 |
| H | 1.925420 | -5.901101 | -3.848916 |
| C | 6.640268 | -1.888750 | -2.354189 |
| H | 5.901101 | -3.848916 | -1.925420 |
| C | 1.888750 | -2.354189 | -6.640268 |
| H | 3.848916 | -1.925420 | -5.901101 |
| C | -4.162751 | 4.162751 | -4.162751 |
| C | -3.847953 | 5.207245 | -3.227563 |
| C | -5.970851 | 3.097086 | -2.708654 |
| C | -3.227563 | 3.847953 | -5.207245 |
| C | -2.708654 | 5.970851 | -3.097086 |
| H | -4.556889 | 5.314870 | -2.408540 |
| C | -5.207245 | 3.227563 | -3.847953 |
| H | -5.901101 | 3.848916 | -1.925420 |
| H | -2.408540 | 4.556889 | -5.314870 |
| H | -1.925420 | 5.901101 | -3.848916 |
| C | -6.640268 | 1.888750 | -2.354189 |
| H | -5.314870 | 2.408540 | -4.556889 |
| C | -2.354189 | 6.640268 | -1.888750 |
| C | 5.970851 | 3.097086 | 2.708654 |
| C | 6.640268 | 1.888750 | 2.354189 |
| C | 7.302294 | -0.207081 | -0.654019 |
| C | 7.073492 | -1.547449 | -1.095661 |
| C | 1.547449 | 1.095661 | 7.073492 |
| C | 0.207081 | 0.654019 | 7.302294 |
| C | -1.888750 | -2.354189 | 6.640268 |
| C | -3.097086 | -2.708654 | 5.970851 |
| C | -7.073492 | 1.547449 | -1.095661 |
| C | -7.302294 | 0.207081 | -0.654019 |
| C | -6.640268 | -1.888750 | 2.354189 |
| C | -5.970851 | -3.097086 | 2.708654 |
| C | -2.708654 | -5.970851 | 3.097086 |
| C | -2.354189 | -6.640268 | 1.888750 |
| C | 0.654019 | -7.302294 | -0.207081 |
| C | 1.095661 | -7.073492 | -1.547449 |
| C | 2.708654 | 5.970851 | 3.097086 |
| C | 2.354189 | 6.640268 | 1.888750 |
| C | -0.654019 | 7.302294 | -0.207081 |
| C | -1.095661 | 7.073492 | -1.547449 |
| C | 1.547449 | -1.095661 | -7.073492 |
| C | -1.888750 | 2.354189 | -6.640268 |
| C | -3.097086 | 2.708654 | -5.970851 |
| C | 0.207081 | -0.654019 | -7.302294 |
| C | 0.654019 | 7.302294 | 0.207081 |
| C | 1.095661 | 7.073492 | 1.547449 |
| C | -7.302294 | -0.207081 | 0.654019 |
| C | -7.073492 | -1.547449 | 1.095661 |
| C | -0.654019 | -7.302294 | 0.207081 |
| C | -1.095661 | -7.073492 | 1.547449 |
| C | 7.302294 | 0.207081 | 0.654019 |
| C | 7.073492 | 1.547449 | 1.095661 |
| C | -1.547449 | 1.095661 | -7.073492 |
| C | -0.207081 | 0.654019 | -7.302294 |
| C | -1.547449 | -1.095661 | 7.073492 |
| C | -0.207081 | -0.654019 | 7.302294 |
| H | -3.848916 | 1.925420 | -5.901101 |
| H | -1.101024 | 3.110445 | -6.640527 |
| H | -2.313325 | 0.320127 | -7.027259 |
| H | 0.565470 | 1.424547 | -7.273010 |
| H | -0.565470 | -1.424547 | -7.273010 |
| H | 2.313325 | -0.320127 | -7.027259 |
| H | 1.101024 | -3.110445 | -6.640527 |
| H | -3.110445 | 6.640527 | -1.101024 |
| H | -0.320127 | 7.027259 | -2.313325 |
| H | -1.424547 | 7.273010 | 0.565470 |
| H | 1.424547 | 7.273010 | -0.565470 |
| H | 0.320127 | 7.027259 | 2.313325 |
| H | 3.110445 | 6.640527 | 1.101024 |
| H | 1.925420 | 5.901101 | 3.848916 |
| H | -6.640527 | 1.101024 | -3.110445 |
| H | -7.027259 | 2.313325 | -0.320127 |
| H | -7.273010 | -0.565470 | -1.424547 |
| H | -7.273010 | 0.565470 | 1.424547 |
| H | -7.027259 | -2.313325 | 0.320127 |
| H | -6.640527 | -1.101024 | 3.110445 |
| H | -5.901101 | -3.848916 | 1.925420 |
| H | -3.848916 | -1.925420 | 5.901101 |
| H | -1.101024 | -3.110445 | 6.640527 |
| H | -2.313325 | -0.320127 | 7.027259 |
| H | 0.565470 | -1.424547 | 7.273010 |
| H | -0.565470 | 1.424547 | 7.273010 |
| H | 2.313325 | 0.320127 | 7.027259 |
| H | 1.101024 | 3.110445 | 6.640527 |
| H | 6.640527 | -1.101024 | -3.110445 |
| H | 7.027259 | -2.313325 | -0.320127 |
| H | 7.273010 | 0.565470 | -1.424547 |
| H | 7.273010 | -0.565470 | 1.424547 |
| H | 7.027259 | 2.313325 | 0.320127 |
| H | 6.640527 | 1.101024 | 3.110445 |
| H | 5.901101 | 3.848916 | 1.925420 |

| | | | |
|---|---|---|---|
| H | -1.925420 | -5.901101 | 3.848916 |
| H | -3.110445 | -6.640527 | 1.101024 |
| H | -0.320127 | -7.027259 | 2.313325 |
| H | -1.424547 | -7.273010 | -0.565470 |
| H | 1.424547 | -7.273010 | 0.565470 |
| H | 0.320127 | -7.027259 | -2.313325 |
| H | 3.110445 | -6.640527 | -1.101024 |

**22**

State = S0

Symmetry = T

E = -2939.73094741 a.u.

| | | | |
|---|---|---|---|
| C | 4.920813 | -4.920813 | 4.920813 |
| C | 6.722647 | -3.487688 | 3.846208 |
| C | 3.995692 | -5.980391 | 4.640690 |
| C | 3.487688 | -3.846208 | 6.722647 |
| C | 5.980391 | -4.640690 | 3.995692 |
| H | 6.590693 | -2.674917 | 4.556859 |
| C | 3.846208 | -6.722647 | 3.487688 |
| H | 3.223421 | -6.136557 | 5.391116 |
| C | 4.640690 | -3.995692 | 5.980391 |
| H | 2.674917 | -4.556859 | 6.590693 |
| C | 7.493459 | -3.184746 | 2.696292 |
| H | 6.136557 | -5.391116 | 3.223421 |
| C | 2.696292 | -7.493459 | 3.184746 |
| H | 4.556859 | -6.590693 | 2.674917 |
| C | 3.184746 | -2.696292 | 7.493459 |
| H | 5.391116 | -3.223421 | 6.136557 |
| C | -4.920813 | 4.920813 | 4.920813 |
| C | -4.640690 | 3.995692 | 5.980391 |
| C | -5.980391 | 4.640690 | 3.995692 |
| C | -3.995692 | 5.980391 | 4.640690 |
| C | -3.487688 | 3.846208 | 6.722647 |
| H | -5.391116 | 3.223421 | 6.136557 |
| C | -6.722647 | 3.487688 | 3.846208 |
| H | -6.136557 | 5.391116 | 3.223421 |
| C | -3.846208 | 6.722647 | 3.487688 |
| H | -4.556859 | 6.590693 | 2.674917 |
| H | -2.674917 | 4.556859 | 6.590693 |
| C | -7.493459 | 3.184746 | 2.696292 |
| H | -6.590693 | 2.674917 | 4.556859 |
| C | -2.696292 | 7.493459 | 3.184746 |
| H | -3.223421 | 6.136557 | 5.391116 |
| C | -4.920813 | -4.920813 | -4.920813 |
| C | -5.980391 | -4.640690 | -3.995692 |
| C | -3.995692 | -5.980391 | -4.640690 |
| C | -4.640690 | -3.995692 | -5.980391 |
| C | -6.722647 | -3.487688 | -3.846208 |
| H | -6.136557 | -5.391116 | -3.223421 |
| C | -3.846208 | -6.722647 | -3.487688 |
| H | -3.223421 | -6.136557 | -5.391116 |
| C | -3.487688 | -3.846208 | -6.722647 |
| H | -5.391116 | -3.223421 | -6.136557 |
| C | -7.493459 | -3.184746 | -2.696292 |
| H | -6.590693 | -2.674917 | -4.556859 |
| C | -2.696292 | -7.493459 | -3.184746 |
| H | -4.556859 | -6.590693 | -2.674917 |
| C | -3.184746 | -2.696292 | -7.493459 |
| H | -2.674917 | -4.556859 | -6.590693 |
| C | 4.920813 | 4.920813 | -4.920813 |
| C | 5.980391 | 4.640690 | -3.995692 |
| C | 3.846208 | 6.722647 | -3.487688 |
| C | 4.640690 | 3.995692 | -5.980391 |
| C | 6.722647 | 3.487688 | -3.846208 |
| H | 6.136557 | 5.391116 | -3.223421 |
| C | 3.995692 | 5.980391 | -4.640690 |
| H | 4.556859 | 6.590693 | -2.674917 |
| C | 3.487688 | 3.846208 | -6.722647 |
| H | 5.391116 | 3.223421 | -6.136557 |
| H | 6.590693 | 2.674917 | -4.556859 |
| C | 2.696292 | 7.493459 | -3.184746 |
| H | 3.223421 | 6.136557 | -5.391116 |
| C | 7.493459 | 3.184746 | -2.696292 |
| H | 2.674917 | 4.556859 | -6.590693 |
| C | 2.375078 | -7.986628 | 1.936086 |
| C | 1.078944 | -8.388214 | 1.528913 |
| C | -1.078944 | -8.388214 | -1.528913 |
| C | -2.375078 | -7.986628 | -1.936086 |
| C | 1.936086 | -2.375078 | 7.986628 |
| C | 1.528913 | -1.078944 | 8.388214 |
| C | -1.528913 | 1.078944 | 8.388214 |
| C | -1.936086 | 2.375078 | 7.986628 |
| C | 2.375078 | 7.986628 | -1.936086 |
| C | 1.078944 | 8.388214 | -1.528913 |
| C | -1.078944 | 8.388214 | 1.528913 |
| C | -2.375078 | 7.986628 | 1.936086 |
| C | -7.986628 | 1.936086 | 2.375078 |
| C | -8.388214 | 1.528913 | 1.078944 |
| C | -8.388214 | -1.528913 | -1.078944 |
| C | -7.986628 | -1.936086 | -2.375078 |
| C | 7.986628 | -1.936086 | 2.375078 |
| C | 8.388214 | -1.528913 | 1.078944 |
| C | 8.388214 | 1.528913 | -1.078944 |
| C | 7.986628 | 1.936086 | -2.375078 |
| C | -1.936086 | -2.375078 | -7.986628 |
| C | 1.528913 | 1.078944 | -8.388214 |
| C | 1.936086 | 2.375078 | -7.986628 |
| C | 3.184746 | 2.696292 | -7.493459 |
| C | -3.184746 | 2.696292 | 7.493459 |
| C | -1.528913 | -1.078944 | -8.388214 |
| C | 8.560564 | 0.221266 | -0.673095 |
| C | 8.560564 | -0.221266 | 0.673095 |
| C | 0.673095 | 8.560564 | -0.221266 |
| C | -0.673095 | 8.560564 | 0.221266 |
| C | -8.560564 | -0.221266 | -0.673095 |
| C | -8.560564 | 0.221266 | 0.673095 |
| C | -0.673095 | -8.560564 | -0.221266 |
| C | 0.673095 | -8.560564 | 0.221266 |
| C | 0.221266 | 0.673095 | -8.560564 |
| C | -0.221266 | -0.673095 | -8.560564 |

| | | | |
|---|---|---|---|
| C | -0.221266 | 0.673095 | 8.560564 |
| C | 0.221266 | -0.673095 | 8.560564 |
| H | 3.962687 | 1.934000 | -7.561709 |
| H | 1.151948 | 3.127813 | -7.896838 |
| H | 2.299317 | 0.306369 | -8.398290 |
| H | -0.552080 | 1.441442 | -8.520147 |
| H | 0.552080 | -1.441442 | -8.520147 |
| H | -2.299317 | -0.306369 | -8.398290 |
| H | -1.151948 | -3.127813 | -7.896838 |
| H | -3.962687 | -1.934000 | -7.561709 |
| H | 7.561709 | 3.962687 | -1.934000 |
| H | 7.896838 | 1.151948 | -3.127813 |
| H | 8.398290 | 2.299317 | -0.306369 |
| H | 8.520147 | -0.552080 | -1.441442 |
| H | 8.520147 | 0.552080 | 1.441442 |
| H | 8.398290 | -2.299317 | 0.306369 |
| H | 7.896838 | -1.151948 | 3.127813 |
| H | 7.561709 | -3.962687 | 1.934000 |
| H | 1.934000 | 7.561709 | -3.962687 |
| H | 3.127813 | 7.896838 | -1.151948 |
| H | 0.306369 | 8.398290 | -2.299317 |
| H | 1.441442 | 8.520147 | 0.552080 |
| H | -1.441442 | 8.520147 | -0.552080 |
| H | -0.306369 | 8.398290 | 2.299317 |
| H | -3.127813 | 7.896838 | 1.151948 |
| H | -1.934000 | 7.561709 | 3.962687 |
| H | -3.962687 | 1.934000 | 7.561709 |
| H | -1.151948 | 3.127813 | 7.896838 |
| H | -2.299317 | 0.306369 | 8.398290 |
| H | 0.552080 | 1.441442 | 8.520147 |
| H | -0.552080 | -1.441442 | 8.520147 |
| H | 2.299317 | -0.306369 | 8.398290 |
| H | 1.151948 | -3.127813 | 7.896838 |
| H | 3.962687 | -1.934000 | 7.561709 |
| H | -1.934000 | -7.561709 | -3.962687 |
| H | -3.127813 | -7.896838 | -1.151948 |
| H | -0.306369 | -8.398290 | -2.299317 |
| H | -1.441442 | -8.520147 | 0.552080 |
| H | 1.441442 | -8.520147 | -0.552080 |
| H | 0.306369 | -8.398290 | 2.299317 |
| H | 3.127813 | -7.896838 | 1.151948 |
| H | 1.934000 | -7.561709 | 3.962687 |
| H | -7.561709 | 3.962687 | 1.934000 |
| H | -7.896838 | 1.151948 | 3.127813 |
| H | -8.398290 | 2.299317 | 0.306369 |
| H | -8.520147 | -0.552080 | 1.441442 |
| H | -8.520147 | 0.552080 | -1.441442 |
| H | -8.398290 | -2.299317 | -0.306369 |
| H | -7.896838 | -1.151948 | -3.127813 |
| H | -7.561709 | -3.962687 | -1.934000 |

**23**

State = Q
Symmetry = T
E = -2939.80752266 a.u.

| | | | |
|---|---|---|---|
| C | 4.899464 | -4.899464 | 4.899464 |
| C | 6.686093 | -3.487956 | 3.812378 |
| C | 3.965769 | -5.955014 | 4.643525 |
| C | 3.487956 | -3.812378 | 6.686093 |
| C | 5.955014 | -4.643525 | 3.965769 |
| H | 6.562362 | -2.675141 | 4.522510 |
| C | 3.812378 | -6.686093 | 3.487956 |
| H | 3.206153 | -6.115021 | 5.403322 |
| C | 4.643525 | -3.965769 | 5.955014 |
| H | 2.675141 | -4.522510 | 6.562362 |
| C | 7.451786 | -3.194798 | 2.658835 |
| H | 6.115021 | -5.403322 | 3.206153 |
| C | 2.658835 | -7.451786 | 3.194798 |
| H | 4.522510 | -6.562362 | 2.675141 |
| C | 3.194798 | -2.658835 | 7.451786 |
| H | 5.403322 | -3.206153 | 6.115021 |
| C | -4.899464 | 4.899464 | 4.899464 |
| C | -4.643525 | 3.965769 | 5.955014 |
| C | -5.955014 | 4.643525 | 3.965769 |
| C | -3.965769 | 5.955014 | 4.643525 |
| C | -3.487956 | 3.812378 | 6.686093 |
| H | -5.403322 | 3.206153 | 6.115021 |
| C | -6.686093 | 3.487956 | 3.812378 |
| H | -6.115021 | 5.403322 | 3.206153 |
| C | -3.812378 | 6.686093 | 3.487956 |
| H | -4.522510 | 6.562362 | 2.675141 |
| H | -2.675141 | 4.522510 | 6.562362 |
| C | -7.451786 | 3.194798 | 2.658835 |
| H | -6.562362 | 2.675141 | 4.522510 |
| C | -2.658835 | 7.451786 | 3.194798 |
| H | -3.206153 | 6.115021 | 5.403322 |
| C | -4.899464 | -4.899464 | -4.899464 |
| C | -5.955014 | -4.643525 | -3.965769 |
| C | -3.965769 | -5.955014 | -4.643525 |
| C | -4.643525 | -3.965769 | -5.955014 |
| C | -6.686093 | -3.487956 | -3.812378 |
| H | -6.115021 | -5.403322 | -3.206153 |
| C | -3.812378 | -6.686093 | -3.487956 |
| H | -3.206153 | -6.115021 | -5.403322 |
| C | -3.487956 | -3.812378 | -6.686093 |
| H | -5.403322 | -3.206153 | -6.115021 |
| C | -7.451786 | -3.194798 | -2.658835 |
| H | -6.562362 | -2.675141 | -4.522510 |
| C | -2.658835 | -7.451786 | -3.194798 |
| H | -4.522510 | -6.562362 | -2.675141 |
| C | -3.194798 | -2.658835 | -7.451786 |
| H | -2.675141 | -4.522510 | -6.562362 |
| C | 4.899464 | 4.899464 | -4.899464 |
| C | 5.955014 | 4.643525 | -3.965769 |
| C | 3.812378 | 6.686093 | -3.487956 |
| C | 4.643525 | 3.965769 | -5.955014 |
| C | 6.686093 | 3.487956 | -3.812378 |
| H | 6.115021 | 5.403322 | -3.206153 |
| C | 3.965769 | 5.955014 | -4.643525 |
| H | 4.522510 | 6.562362 | -2.675141 |

| | | | |
|---|---|---|---|
| C | 3.487956 | 3.812378 | -6.686093 |
| H | 5.403322 | 3.206153 | -6.115021 |
| H | 6.562362 | 2.675141 | -4.522510 |
| C | 2.658835 | 7.451786 | -3.194798 |
| H | 3.206153 | 6.115021 | -5.403322 |
| C | 7.451786 | 3.194798 | -2.658835 |
| H | 2.675141 | 4.522510 | -6.562362 |
| C | 2.349026 | -7.926730 | 1.940134 |
| C | 1.054056 | -8.325722 | 1.534080 |
| C | -1.054056 | -8.325722 | -1.534080 |
| C | -2.349026 | -7.926730 | -1.940134 |
| C | 1.940134 | -2.349026 | 7.926730 |
| C | 1.534080 | -1.054056 | 8.325722 |
| C | -1.534080 | 1.054056 | 8.325722 |
| C | -1.940134 | 2.349026 | 7.926730 |
| C | 2.349026 | 7.926730 | -1.940134 |
| C | 1.054056 | 8.325722 | -1.534080 |
| C | -1.054056 | 8.325722 | 1.534080 |
| C | -2.349026 | 7.926730 | 1.940134 |
| C | -7.926730 | 1.940134 | 2.349026 |
| C | -8.325722 | 1.534080 | 1.054056 |
| C | -8.325722 | -1.534080 | -1.054056 |
| C | -7.926730 | -1.940134 | -2.349026 |
| C | 7.926730 | -1.940134 | 2.349026 |
| C | 8.325722 | -1.534080 | 1.054056 |
| C | 8.325722 | 1.534080 | -1.054056 |
| C | 7.926730 | 1.940134 | -2.349026 |
| C | -1.940134 | -2.349026 | -7.926730 |
| C | 1.534080 | 1.054056 | -8.325722 |
| C | 1.940134 | 2.349026 | -7.926730 |
| C | 3.194798 | 2.658835 | -7.451786 |
| C | -3.194798 | 2.658835 | 7.451786 |
| C | -1.534080 | -1.054056 | -8.325722 |
| C | 8.489793 | 0.222099 | -0.671649 |
| C | 8.489793 | -0.222099 | 0.671649 |
| C | 0.671649 | 8.489793 | -0.222099 |
| C | -0.671649 | 8.489793 | 0.222099 |
| C | -8.489793 | -0.222099 | -0.671649 |
| C | -8.489793 | 0.222099 | 0.671649 |
| C | -0.671649 | -8.489793 | -0.222099 |
| C | 0.671649 | -8.489793 | 0.222099 |
| C | 0.222099 | 0.671649 | -8.489793 |
| C | -0.222099 | -0.671649 | -8.489793 |
| C | -0.222099 | 0.671649 | 8.489793 |
| C | 0.222099 | -0.671649 | 8.489793 |
| H | 3.974170 | 1.902130 | -7.532979 |
| H | 1.162368 | 3.106749 | -7.841728 |
| H | 2.299025 | 0.279006 | -8.350380 |
| H | -0.542634 | 1.446615 | -8.458616 |
| H | 0.542634 | -1.446615 | -8.458616 |
| H | -2.299025 | -0.279006 | -8.350380 |
| H | -1.162368 | -3.106749 | -7.841728 |
| H | -3.974170 | -1.902130 | -7.532979 |
| H | 7.532979 | 3.974170 | -1.902130 |
| H | 7.841728 | 1.162368 | -3.106749 |
| H | 8.350380 | 2.299025 | -0.279006 |
| H | 8.458616 | -0.542634 | -1.446615 |
| H | 8.458616 | 0.542634 | 1.446615 |
| H | 8.350380 | -2.299025 | 0.279006 |
| H | 7.841728 | -1.162368 | 3.106749 |
| H | 7.532979 | -3.974170 | 1.902130 |
| H | 1.902130 | 7.532979 | -3.974170 |
| H | 3.106749 | 7.841728 | -1.162368 |
| H | 0.279006 | 8.350380 | -2.299025 |
| H | 1.446615 | 8.458616 | 0.542634 |
| H | -1.446615 | 8.458616 | -0.542634 |
| H | -0.279006 | 8.350380 | 2.299025 |
| H | -3.106749 | 7.841728 | 1.162368 |
| H | -1.902130 | 7.532979 | 3.974170 |
| H | -3.974170 | 1.902130 | 7.532979 |
| H | -1.162368 | 3.106749 | 7.841728 |
| H | -2.299025 | 0.279006 | 8.350380 |
| H | 0.542634 | 1.446615 | 8.458616 |
| H | -0.542634 | -1.446615 | 8.458616 |
| H | 2.299025 | -0.279006 | 8.350380 |
| H | 1.162368 | -3.106749 | 7.841728 |
| H | 3.974170 | -1.902130 | 7.532979 |
| H | -1.902130 | -7.532979 | -3.974170 |
| H | -3.106749 | -7.841728 | -1.162368 |
| H | -0.279006 | -8.350380 | -2.299025 |
| H | -1.446615 | -8.458616 | 0.542634 |
| H | 1.446615 | -8.458616 | -0.542634 |
| H | 0.279006 | -8.350380 | 2.299025 |
| H | 3.106749 | -7.841728 | 1.162368 |
| H | 1.902130 | -7.532979 | 3.974170 |
| H | -7.532979 | 3.974170 | 1.902130 |
| H | -7.841728 | 1.162368 | 3.106749 |
| H | -8.350380 | 2.299025 | 0.279006 |
| H | -8.458616 | -0.542634 | 1.446615 |
| H | -8.458616 | 0.542634 | -1.446615 |
| H | -8.350380 | -2.299025 | -0.279006 |
| H | -7.841728 | -1.162368 | -3.106749 |
| H | -7.532979 | -3.974170 | -1.902130 |

State = $D_1$
Symmetry = $C_1$
E = -1980.65977477 a.u

| | | | |
|---|---|---|---|
| C | 6.046323 | 1.130284 | -0.240878 |
| C | 5.695892 | 2.236751 | -0.647723 |
| C | 5.061377 | 3.336813 | -1.060039 |
| C | 2.087992 | 5.452832 | -1.900992 |
| C | 0.796943 | 5.759163 | -2.046200 |
| C | -0.432445 | 5.762594 | -2.080981 |
| C | -0.943686 | -1.064746 | 5.974859 |
| C | 0.282739 | -1.107872 | 6.056364 |
| C | 1.597562 | -1.096388 | 5.826523 |
| C | 4.751083 | -0.805450 | 3.827375 |
| C | 5.481767 | -0.621830 | 2.725055 |
| C | 5.929659 | -0.426901 | 1.596339 |
| C | -3.129818 | 2.791132 | -4.491334 |

| | | | |
|---|---|---|---|
| C | -2.816351 | 3.911530 | -3.837731 |
| C | -2.394956 | 4.760845 | -3.054263 |
| C | -2.851649 | 0.301793 | 5.432170 |
| C | -3.229790 | 1.409398 | 5.054083 |
| C | -3.475318 | 2.538387 | 4.386355 |
| C | -3.294307 | 4.955249 | 1.534629 |
| C | -2.456816 | 5.574449 | -0.789193 |
| C | -2.930233 | 5.409817 | 0.333973 |
| C | -2.696473 | -4.824912 | -2.679936 |
| C | -3.171157 | -5.045045 | -1.566861 |
| C | -3.524391 | -5.026302 | -0.280142 |
| C | -3.620605 | -3.761816 | 3.240941 |
| C | -3.330412 | -2.952082 | 4.261179 |
| C | -2.906325 | -2.066324 | 5.001655 |
| C | 5.991666 | -1.241366 | -0.672289 |
| C | 5.595518 | -2.117107 | -1.439587 |
| C | 4.915893 | -2.972292 | -2.207380 |
| C | 1.858042 | -4.522532 | -3.714763 |
| C | 0.555852 | -4.700378 | -3.947979 |
| C | -0.672151 | -4.636611 | -3.971779 |
| C | 4.254362 | 4.209216 | -1.395301 |
| C | 3.209678 | 4.977011 | -1.699528 |
| C | -3.506838 | 4.347402 | 2.588127 |
| C | -3.571187 | 3.487584 | 3.602558 |
| C | -3.301713 | 1.648625 | -4.926813 |
| C | -3.331895 | 0.339078 | -5.164865 |
| C | 4.073513 | -3.634031 | -2.821600 |
| C | 2.998139 | -4.198434 | -3.367896 |
| C | 3.868644 | -0.940253 | 4.680549 |
| C | 2.758534 | -1.047097 | 5.408320 |
| C | -3.718153 | -4.819148 | 0.921541 |
| C | -3.752379 | -4.369316 | 2.174133 |
| C | -3.214752 | -0.890055 | -5.160670 |
| C | -2.956864 | -2.181553 | -4.945537 |
| C | -2.580151 | -3.270117 | -4.514436 |
| C | -1.803484 | 5.493146 | -2.021649 |
| C | -2.030241 | -4.344445 | -3.810293 |
| C | 6.120495 | -0.183275 | 0.232730 |
| C | -2.287133 | -0.965423 | 5.599180 |

**24**

State = $S_0$

Symmetry = $C_1$

E = -2162.88051173 a.u.

| | | | |
|---|---|---|---|
| C | -2.872995 | 0.069032 | -3.989372 |
| C | -3.043735 | -1.329562 | -3.605983 |
| C | -3.914180 | -1.881988 | -2.718209 |
| C | -3.529276 | -3.046291 | -1.968143 |
| C | -4.050561 | -3.379650 | -0.756638 |
| C | -3.664944 | 1.051093 | -3.389119 |
| C | -3.384927 | 2.430804 | -3.264033 |
| C | -3.729803 | 3.107630 | -2.109650 |
| C | -3.027609 | 4.239078 | -1.636236 |
| C | -1.613919 | 0.437264 | -4.594141 |
| C | -0.592430 | -0.381869 | -4.993194 |
| C | 0.762705 | 0.060876 | -4.948176 |
| C | 1.828737 | -0.791136 | -4.854171 |
| C | -2.807268 | 4.531172 | -0.289178 |
| C | -3.354110 | -4.146428 | 0.269853 |
| C | 3.100816 | -0.472300 | -4.250129 |
| C | -3.544661 | -3.828332 | 1.615398 |
| C | -4.103175 | -2.617797 | 2.084922 |
| C | -3.682805 | -1.988764 | 3.241172 |
| C | -3.789929 | -0.584906 | 3.365816 |
| C | -2.213137 | -4.951973 | -0.139266 |
| C | -1.042084 | -5.007074 | 0.562856 |
| C | 0.254483 | -5.233894 | 0.005070 |
| C | 1.352509 | -4.735760 | 0.652758 |
| C | -3.600869 | 3.857767 | 0.732117 |
| C | -3.134480 | 3.463318 | 1.947703 |
| C | -3.666574 | 2.355622 | 2.693401 |
| C | -2.879227 | 1.700209 | 3.588732 |
| C | -1.577325 | 5.187816 | 0.127683 |
| C | -0.404208 | 5.097504 | -0.567457 |
| C | 0.906815 | 5.160582 | -0.001098 |
| C | 1.938854 | 4.530581 | -0.642473 |
| C | 3.831704 | -1.459021 | -3.583088 |
| C | 3.300824 | -2.692437 | -3.143388 |
| C | 3.684448 | -3.308738 | -1.967469 |
| C | 2.775378 | -4.139282 | -1.270689 |
| C | 2.598125 | -4.227826 | 0.111511 |
| C | -2.885888 | 0.291105 | 3.971595 |
| C | 3.399336 | 0.943451 | -4.014722 |
| C | 3.898471 | 1.411517 | -2.840843 |
| C | 3.677485 | 2.726080 | -2.300214 |
| C | 3.802191 | 2.927543 | -0.958831 |
| C | 3.107917 | 3.871236 | -0.093727 |
| C | 3.398686 | -3.377056 | 0.981169 |
| C | 3.292731 | -3.162039 | 2.322062 |
| C | 3.671211 | -1.884566 | 2.864457 |
| C | 3.228827 | -1.357906 | 4.036172 |
| C | -1.686025 | -0.230729 | 4.584014 |
| C | -0.572758 | 0.454967 | 4.988878 |
| C | 0.717004 | -0.153232 | 4.953130 |
| C | 1.880989 | 0.559769 | 4.864457 |
| C | 3.263998 | 3.761691 | 1.289502 |
| C | 4.057216 | 2.823949 | 1.991847 |
| C | 3.592126 | 2.261666 | 3.165372 |
| C | 3.960976 | 0.971394 | 3.608118 |
| C | 3.107698 | 0.084390 | 4.269650 |
| H | -1.982766 | 2.237014 | 3.878221 |
| H | -4.597588 | 1.922565 | 2.337902 |
| H | -2.165724 | 3.824204 | 2.280726 |
| H | -4.560059 | 3.459938 | 0.416122 |
| H | -1.569335 | -1.308057 | 4.530706 |
| H | -0.600975 | 1.531858 | 5.123913 |
| H | 0.732139 | -1.219586 | 4.749508 |
| H | 1.816338 | 1.625544 | 5.059805 |
| H | -4.536607 | -0.132135 | 2.720250 |

| | | | |
|---|---|---|---|
| H | -3.045740 | -2.547854 | 3.919251 |
| H | -4.693403 | -2.019526 | 1.397969 |
| H | -2.984967 | -4.408903 | 2.343241 |
| H | 2.707481 | -2.012952 | 4.728114 |
| H | 4.853179 | 0.547542 | 3.155595 |
| H | 2.717704 | 2.728942 | 3.607522 |
| H | 4.147734 | -1.216144 | 2.152772 |
| H | 2.726482 | -3.833612 | 2.961073 |
| H | 4.030609 | -2.668196 | 0.456439 |
| H | 4.898605 | 2.347588 | 1.498816 |
| H | 2.534848 | 4.287382 | 1.899216 |
| H | 1.738908 | 4.281683 | -1.679807 |
| H | 4.339230 | 2.146764 | -0.430540 |
| H | 3.201269 | 3.461643 | -2.942202 |
| H | 0.996702 | 5.483416 | 1.032499 |
| H | -0.440569 | 4.695588 | -1.575920 |
| H | -1.533170 | 5.537592 | 1.155147 |
| H | 4.285985 | 0.689747 | -2.127238 |
| H | 2.966478 | 1.658333 | -4.708438 |
| H | 1.633951 | -1.840390 | -5.052586 |
| H | 4.766953 | -1.150327 | -3.124337 |
| H | 2.377207 | -3.046034 | -3.591033 |
| H | 4.576019 | -2.941929 | -1.468977 |
| H | 1.989867 | -4.569496 | -1.885252 |
| H | 1.178217 | -4.461837 | 1.688410 |
| H | 0.310297 | -5.567404 | -1.027536 |
| H | -1.034269 | -4.602042 | 1.570694 |
| H | -2.207060 | -5.306310 | -1.166100 |
| H | -4.955519 | -2.866117 | -0.447545 |
| H | -2.609932 | -3.524490 | -2.293309 |
| H | -4.788184 | -1.337404 | -2.371449 |
| H | -2.217330 | -1.972909 | -3.886783 |
| H | -1.363913 | 1.491557 | -4.538817 |
| H | -0.754148 | -1.446645 | -5.130396 |
| H | 0.908930 | 1.116681 | -4.741553 |
| H | -4.465611 | 0.694940 | -2.747739 |
| H | -2.679628 | 2.905959 | -3.938601 |
| H | -4.393324 | 2.587339 | -1.426121 |
| H | -2.395444 | 4.745181 | -2.360313 |

**25**

State = $S_0$

Symmetry = $C_1$

E = -3092.07811072 a.u.

| | | | |
|---|---|---|---|
| C | -4.014494 | 5.656521 | -0.424742 |
| C | -2.829397 | 6.337356 | 0.068026 |
| C | -1.799858 | 6.888260 | -0.636236 |
| C | -0.509388 | 7.058690 | -0.046620 |
| C | 0.663757 | 7.217847 | -0.727387 |
| C | 1.921633 | 6.882759 | -0.133351 |
| C | 3.077683 | 6.647096 | -0.811034 |
| C | -4.203882 | 5.465044 | -1.790180 |
| C | -5.104994 | 4.575769 | -2.422862 |
| C | -4.861893 | 4.056437 | -3.673264 |
| C | -5.463230 | 2.875048 | -4.176351 |
| C | -4.844547 | 2.084853 | -5.116491 |
| C | -5.153316 | 0.720228 | -5.330741 |
| C | -4.801385 | 4.889374 | 0.526568 |
| C | -4.710690 | 4.854429 | 1.884407 |
| C | -5.228505 | 3.747592 | 2.633915 |
| C | -4.929787 | 3.448978 | 3.928583 |
| C | -5.078170 | 2.118456 | 4.445229 |
| C | -4.499166 | 1.648575 | 5.579468 |
| C | -4.301666 | -0.240523 | -5.867284 |
| C | 4.221789 | 5.917031 | -0.275392 |
| C | -4.289735 | 0.231191 | 5.875933 |
| C | 4.966886 | 5.051848 | -1.171552 |
| C | 4.654739 | 4.711439 | -2.452053 |
| C | 5.213122 | 3.559284 | -3.095723 |
| C | 4.738907 | 3.007741 | -4.247463 |
| C | 4.964503 | 1.637696 | -4.605867 |
| C | 4.261481 | 0.980266 | -5.563725 |
| C | 4.430702 | 5.847327 | 1.096637 |
| C | 5.198518 | 4.883359 | 1.797691 |
| C | 4.969983 | 4.548408 | 3.109908 |
| C | 5.433952 | 3.338406 | 3.690984 |
| C | 4.799078 | 2.707386 | 4.733046 |
| C | 5.002426 | 1.339810 | 5.042014 |
| C | -3.059060 | 0.144870 | -6.496763 |
| C | -1.956548 | -0.639638 | -6.674094 |
| C | -0.658356 | -0.112029 | -6.941821 |
| C | 0.493750 | -0.836589 | -6.825416 |
| C | 1.784501 | -0.254082 | -6.650481 |
| C | 2.876316 | -0.971290 | -6.256138 |
| C | -4.507277 | -1.658425 | -5.570449 |
| C | -5.082347 | -2.129530 | -4.434735 |
| C | -4.930104 | -3.459781 | -3.918478 |
| C | -5.225158 | -3.758902 | -2.623094 |
| C | -4.703713 | -4.864750 | -1.874648 |
| C | -4.791217 | -4.899616 | -0.516600 |
| C | -3.044923 | -0.151401 | 6.502748 |
| C | -1.943753 | 0.635485 | 6.677745 |
| C | -0.643853 | 0.110605 | 6.942593 |
| C | 0.506458 | 0.837646 | 6.824084 |
| C | 1.798083 | 0.257938 | 6.646279 |
| C | 2.887653 | 0.977598 | 6.250266 |
| C | 4.120979 | 0.477054 | 5.683958 |
| C | 4.109641 | -0.467993 | -5.692310 |
| C | -5.140334 | -0.731549 | 5.341229 |
| C | -4.829051 | -2.095505 | 5.126472 |
| C | -5.447916 | -2.887046 | 4.187563 |
| C | -4.845075 | -4.067153 | 3.683316 |
| C | -5.089619 | -4.587070 | 2.433424 |
| C | -4.187816 | -5.474293 | 1.798886 |
| C | -4.000823 | -5.665250 | 0.433022 |
| C | 4.275793 | -0.970891 | 5.555175 |
| C | 4.977984 | -1.626750 | 4.595637 |
| C | 4.754767 | -2.997338 | 4.237800 |
| C | 5.227640 | -3.547777 | 3.084977 |
| C | 4.670446 | -4.701184 | 2.442511 |

| | | | |
|---|---|---|---|
| C | 4.980579 | -5.040914 | 1.161347 |
| C | 4.994295 | -1.328804 | -5.052106 |
| C | 4.794691 | -2.696861 | -4.742880 |
| C | 5.433080 | -3.326494 | -3.702107 |
| C | 4.973206 | -4.537641 | -3.120188 |
| C | 5.205197 | -4.871951 | -1.808398 |
| C | 4.441373 | -5.837818 | -1.105649 |
| C | -2.815250 | -6.343433 | -0.062186 |
| C | -1.783231 | -6.892437 | 0.639951 |
| C | -0.493514 | -7.059727 | 0.047826 |
| C | 0.681335 | -7.216592 | 0.726189 |
| C | 1.937215 | -6.878469 | 0.129683 |
| C | 3.094206 | -6.640442 | 0.804937 |
| C | 4.235491 | -5.907819 | 0.266849 |
| H | 2.753551 | -2.049163 | -6.248110 |
| H | 1.827132 | 0.829666 | -6.694757 |
| H | 0.415136 | -1.914799 | -6.696190 |
| H | -0.580150 | 0.964896 | -7.081195 |
| H | -2.006821 | -1.700612 | -6.448006 |
| H | -2.942251 | 1.197537 | -6.733282 |
| H | 5.870193 | -0.878102 | -4.594086 |
| H | 3.982637 | -3.236658 | -5.220046 |
| H | 6.209812 | -2.780889 | -3.168726 |
| H | 4.244534 | -5.119218 | -3.682310 |
| H | 5.887274 | -4.233785 | -1.255296 |
| H | 3.828899 | -6.485843 | -1.726344 |
| H | 3.616169 | 1.587235 | -6.190360 |
| H | 5.607939 | 1.077455 | -3.932717 |
| H | 4.009977 | 3.563249 | -4.834783 |
| H | 5.951621 | 2.996883 | -2.526423 |
| H | 3.839340 | 5.208683 | -2.969023 |
| H | 5.794337 | 4.508628 | -0.727294 |
| H | 5.805894 | -4.495914 | 0.715304 |
| H | 3.106742 | -6.854181 | 1.869752 |
| H | 1.888835 | -6.616372 | -0.923986 |
| H | 3.857222 | -5.200176 | 2.961220 |
| H | 5.963576 | -2.983682 | 2.514037 |
| H | 0.658846 | -7.415674 | 1.795762 |
| H | 4.028487 | -3.554531 | 4.826799 |
| H | 5.618379 | -1.065002 | 3.920846 |
| H | 3.633459 | -1.579254 | 6.183525 |
| H | -0.430383 | -6.856918 | -1.020374 |
| H | -1.857935 | -7.046380 | 1.713112 |
| H | -2.641888 | -6.238505 | -1.128295 |
| H | -5.442812 | -4.151397 | -0.077822 |
| H | -3.454068 | -5.925080 | 2.460494 |
| H | -5.911768 | -4.161046 | 1.866981 |
| H | -4.000082 | -4.465428 | 4.243018 |
| H | -4.106772 | -5.592093 | -2.417258 |
| H | -5.764382 | -3.008642 | -2.046410 |
| H | -4.393694 | -4.184780 | -4.527356 |
| H | -5.541535 | -1.418874 | -3.752288 |
| H | -4.005751 | -2.372678 | -6.217091 |
| H | -6.317587 | -2.495936 | 3.662874 |
| H | -3.930676 | -2.500808 | 5.581851 |
| H | -6.058310 | -0.376446 | 4.880995 |
| H | -3.997503 | 2.363972 | 6.224737 |
| H | -2.925276 | -1.203807 | 6.738992 |
| H | -1.996808 | 1.696384 | 6.451962 |
| H | -0.563059 | -0.966194 | 7.081471 |
| H | 0.425288 | 1.915741 | 6.695484 |
| H | 1.843060 | -0.825742 | 6.689854 |
| H | 2.762650 | 2.055220 | 6.242969 |
| H | 5.878505 | 0.891093 | 4.582388 |
| H | 3.986708 | 3.245357 | 5.211733 |
| H | 6.210925 | 2.794606 | 3.156119 |
| H | 4.240975 | 5.128176 | 3.673464 |
| H | 5.881139 | 4.247035 | 1.243144 |
| H | 3.817825 | 6.493638 | 1.718722 |
| H | 3.087445 | 6.860700 | -1.875904 |
| H | 1.876047 | 6.620906 | 0.920502 |
| H | 0.638656 | 7.416387 | -1.797004 |
| H | -0.443738 | 6.856522 | 1.021547 |
| H | -1.876832 | 7.041516 | -1.709339 |
| H | -2.653838 | 6.233236 | 1.133849 |
| H | -5.452721 | 4.140136 | 0.089162 |
| H | -4.113678 | 5.582624 | 2.425791 |
| H | -5.767719 | 2.996398 | 2.058448 |
| H | -4.393269 | 4.174999 | 4.536147 |
| H | -5.537695 | 1.406889 | 3.763964 |
| H | -3.472511 | 5.917415 | -2.453339 |
| H | -5.925026 | 4.147913 | -1.854740 |
| H | -4.018930 | 4.456561 | -4.234704 |
| H | -6.331090 | 2.482136 | -3.650010 |
| H | -3.947912 | 2.492041 | -5.573614 |
| H | -6.069541 | 0.363032 | -4.868650 |

**26**

State = S0
Symmetry = $C_2$
E = -2629.95744123 a.u.

| | | | |
|---|---|---|---|
| C | 5.270776 | -3.798653 | 3.729395 |
| C | 4.010547 | -3.017510 | 5.785536 |
| C | 6.008058 | -3.305683 | 2.595746 |
| C | 4.304139 | -5.830053 | 2.527656 |
| C | 5.023662 | -2.936576 | 4.850714 |
| H | 3.344921 | -3.877328 | 5.760561 |
| C | 6.426086 | -2.022140 | 2.322016 |
| H | 6.132497 | -4.015246 | 1.779797 |
| C | 4.553425 | -5.032454 | 3.629570 |
| H | 4.792078 | -5.605501 | 1.581647 |
| C | 3.591445 | -1.954366 | 6.622233 |
| H | 5.615374 | -2.023603 | 4.883161 |
| C | 6.821723 | -1.564433 | 1.035181 |
| H | 6.296491 | -1.250706 | 3.078399 |
| C | 3.248875 | -6.772498 | 2.481697 |
| H | 4.001677 | -5.317500 | 4.522972 |
| C | -5.270589 | -3.799294 | -3.729622 |
| C | -4.553318 | -5.033128 | -3.629572 |

| | | | |
|---|---|---|---|
| C | -5.023408 | -2.937378 | -4.851022 |
| C | -6.008102 | -3.306224 | -2.596151 |
| C | -4.303927 | -5.830350 | -2.527405 |
| H | -4.001763 | -5.318508 | -4.522990 |
| C | -4.010026 | -3.018239 | -5.785574 |
| H | -5.615401 | -2.024598 | -4.883859 |
| C | -6.425586 | -2.022514 | -2.322428 |
| H | -6.295081 | -1.251049 | -3.078620 |
| H | -4.791535 | -5.605229 | -1.581361 |
| C | -3.591122 | -1.955220 | -6.622508 |
| H | -3.344077 | -3.877806 | -5.760161 |
| C | -6.821836 | -1.564748 | -1.035793 |
| H | -6.133294 | -4.015905 | -1.780417 |
| C | 5.270589 | 3.799294 | -3.729622 |
| C | 5.023408 | 2.937378 | -4.851022 |
| C | 6.008102 | 3.306224 | -2.596151 |
| C | 4.553318 | 5.033128 | -3.629572 |
| C | 4.010026 | 3.018239 | -5.785574 |
| H | 5.615401 | 2.024598 | -4.883859 |
| C | 6.425586 | 2.022514 | -2.322428 |
| H | 6.133294 | 4.015905 | -1.780417 |
| C | 4.303927 | 5.830350 | -2.527405 |
| H | 4.001763 | 5.318508 | -4.522990 |
| C | 3.591122 | 1.955220 | -6.622508 |
| H | 3.344077 | 3.877806 | -5.760161 |
| C | 6.821836 | 1.564748 | -1.035793 |
| H | 6.295081 | 1.251049 | -3.078620 |
| C | 3.248875 | 6.773035 | -2.481262 |
| H | 4.791535 | 5.605229 | -1.581361 |
| C | -5.270776 | 3.798653 | 3.729395 |
| C | -5.023662 | 2.936576 | 4.850714 |
| C | -6.426086 | 2.022140 | 2.322016 |
| C | -4.553425 | 5.032454 | 3.629570 |
| C | -4.010547 | 3.017510 | 5.785536 |
| H | -5.615374 | 2.023603 | 4.883161 |
| C | -6.008058 | 3.305683 | 2.595746 |
| H | -6.296491 | 1.250706 | 3.078399 |
| C | -4.304139 | 5.830053 | 2.527656 |
| H | -4.001677 | 5.317500 | 4.522972 |
| H | -3.344921 | 3.877328 | 5.760561 |
| C | -6.821723 | 1.564433 | 1.035181 |
| H | -6.132497 | 4.015246 | 1.779797 |
| C | -3.591445 | 1.954366 | 6.622233 |
| H | -4.792078 | 5.605501 | 1.581647 |
| C | 6.966656 | -0.243662 | 0.667010 |
| C | 6.966258 | 0.243922 | -0.667643 |
| C | 2.716679 | -7.340160 | 1.337988 |
| C | 1.398727 | -7.848011 | 1.238547 |
| C | -1.398711 | -7.848287 | -1.237768 |
| C | -2.716543 | -7.340159 | -1.337348 |
| C | -6.966656 | 0.243662 | 0.667010 |
| C | -6.966258 | -0.243922 | -0.667643 |
| C | -2.363986 | -1.903083 | -7.257776 |
| C | -1.720406 | -0.725744 | -7.708863 |
| C | 1.720406 | 0.725744 | -7.708863 |
| C | 2.363986 | 1.903083 | -7.257776 |
| C | 2.364458 | -1.902483 | 7.257777 |
| C | 1.720583 | -0.725255 | 7.708788 |
| C | -1.720583 | 0.725255 | 7.708788 |
| C | -2.364458 | 1.902483 | 7.257777 |
| C | 2.716543 | 7.340159 | -1.337348 |
| C | -1.398727 | 7.848011 | 1.238547 |
| C | -2.716679 | 7.340160 | 1.337988 |
| C | -3.248875 | 6.772498 | 2.481697 |
| C | -3.248875 | -6.773035 | -2.481262 |
| C | 1.398711 | 7.848287 | -1.237768 |
| C | -0.361627 | 0.608642 | 7.934576 |
| C | 0.361627 | -0.608642 | 7.934576 |
| C | 0.361449 | 0.608737 | -7.934558 |
| C | -0.361449 | -0.608737 | -7.934558 |
| C | -0.705755 | 8.053187 | 0.059940 |
| C | 0.705757 | 8.053023 | -0.059075 |
| C | -0.705757 | -8.053023 | -0.059075 |
| C | 0.705755 | -8.053187 | 0.059940 |
| H | -2.687809 | 6.910618 | 3.408054 |
| H | -3.257392 | 7.192014 | 0.402322 |
| H | -0.823280 | 7.881190 | 2.165432 |
| H | -1.272249 | 7.993801 | -0.870763 |
| H | 1.272227 | 7.992978 | 0.871598 |
| H | 0.823309 | 7.882104 | -2.164659 |
| H | 3.257084 | 7.191382 | -0.401681 |
| H | 2.688085 | 6.911771 | -3.407693 |
| H | -4.194816 | 1.045014 | 6.610615 |
| H | -1.751812 | 2.804597 | 7.222010 |
| H | -2.305381 | -0.195733 | 7.682779 |
| H | 0.228359 | 1.526014 | 7.902135 |
| H | -0.228359 | -1.526014 | 7.902135 |
| H | 2.305381 | 0.195733 | 7.682779 |
| H | 1.751812 | -2.804597 | 7.222010 |
| H | 4.194816 | -1.045014 | 6.610615 |
| H | -6.855227 | 2.312987 | 0.241588 |
| H | -6.915085 | -0.505959 | 1.458150 |
| H | -6.913605 | 0.505704 | -1.458707 |
| H | -6.856369 | -2.313361 | -0.242301 |
| H | -2.688085 | -6.911771 | -3.407693 |
| H | -3.257084 | -7.191382 | -0.401681 |
| H | -0.823309 | -7.882104 | -2.164659 |
| H | -1.272227 | -7.992978 | 0.871598 |
| H | 1.272249 | -7.993801 | -0.870763 |
| H | 0.823280 | -7.881190 | 2.165432 |
| H | 3.257392 | -7.192014 | 0.402322 |
| H | 2.687809 | -6.910618 | 3.408054 |
| H | 6.856369 | 2.313361 | -0.242301 |
| H | 6.913605 | -0.505704 | -1.458707 |
| H | 6.915085 | 0.505959 | 1.458150 |
| H | 6.855227 | -2.312987 | 0.241588 |
| H | -4.194804 | -1.046068 | -6.611319 |
| H | -1.751072 | -2.804997 | -7.221657 |
| H | -2.305480 | 0.195073 | -7.682996 |
| H | 0.228800 | -1.525938 | -7.902057 |

| | | | |
|---|---|---|---|
| H | -0.228800 | 1.525938 | -7.902057 |
| H | 2.305480 | -0.195073 | -7.682996 |
| H | 1.751072 | 2.804997 | -7.221657 |
| H | 4.194804 | 1.046068 | -6.611319 |

**27**
State = $S_0$
Symmetry = $C_S$
E = -2437.59607580 a.u.

| | | | |
|---|---|---|---|
| C | -4.423592 | 3.583062 | 0.000000 |
| C | -3.840599 | 4.011043 | 1.200882 |
| C | -3.113917 | 4.189205 | 2.170743 |
| C | -2.166544 | 4.188346 | 3.118261 |
| C | -1.196866 | 4.008767 | 3.844813 |
| C | -3.840599 | 4.011043 | -1.200882 |
| C | -3.113917 | 4.189205 | -2.170743 |
| C | -2.166544 | 4.188346 | -3.118261 |
| C | -1.196866 | 4.008767 | -3.844813 |
| C | -5.293387 | 2.511610 | 0.000000 |
| C | -5.815645 | 1.392599 | 0.000000 |
| C | -6.118622 | 0.103768 | 0.000000 |
| C | -6.145741 | -1.130824 | 0.000000 |
| C | 0.003529 | 3.578930 | -4.427501 |
| C | 0.003529 | 3.578930 | 4.427501 |
| C | -5.840620 | -2.478575 | 0.000000 |
| C | 1.204937 | 4.006718 | 3.845581 |
| C | 2.174694 | 4.185875 | 3.118975 |
| C | 3.121900 | 4.185428 | 2.171328 |
| C | 3.847634 | 4.006155 | 1.200944 |
| C | 0.002231 | 2.506779 | 5.296455 |
| C | 0.001077 | 1.387427 | 5.817925 |
| C | -0.000354 | 0.098347 | 6.119600 |
| C | -0.001338 | -1.136243 | 6.146465 |
| C | 1.204937 | 4.006718 | -3.845581 |
| C | 2.174694 | 4.185875 | -3.118975 |
| C | 3.121900 | 4.185428 | -2.171328 |
| C | 3.847634 | 4.006155 | -1.200944 |
| C | 0.002231 | 2.506779 | -5.296455 |
| C | 0.001077 | 1.387427 | -5.817925 |
| C | -0.000354 | 0.098347 | -6.119600 |
| C | -0.001338 | -1.136243 | -6.146465 |
| C | -5.516027 | -3.089736 | 1.217760 |
| C | -4.986088 | -3.436158 | 2.268452 |
| C | -4.208724 | -3.636865 | 3.335177 |
| C | -3.338987 | -3.636153 | 4.206814 |
| C | -2.271621 | -3.438052 | 4.983932 |
| C | -1.220695 | -3.093422 | 5.514593 |
| C | -0.002430 | -2.483787 | 5.839957 |
| C | 4.429235 | 3.576726 | -0.000000 |
| C | -5.516027 | -3.089736 | -1.217760 |
| C | -4.986088 | -3.436158 | -2.268452 |
| C | -4.208724 | -3.636865 | -3.335177 |
| C | -3.338987 | -3.636153 | -4.206814 |
| C | -2.271621 | -3.438052 | -4.983932 |
| C | -1.220695 | -3.093422 | -5.514593 |
| C | -0.002430 | -2.483787 | -5.839957 |
| C | 1.215083 | -3.095198 | 5.515164 |
| C | 2.265419 | -3.441102 | 4.984105 |
| C | 3.332280 | -3.641321 | 4.206887 |
| C | 4.202907 | -3.641235 | 3.336077 |
| C | 4.980097 | -3.442680 | 2.268907 |
| C | 5.510191 | -3.097653 | 1.217798 |
| C | 5.297107 | 2.503658 | -0.000000 |
| C | 5.817754 | 1.383944 | -0.000000 |
| C | 6.118317 | 0.094573 | -0.000000 |
| C | 6.143713 | -1.140032 | -0.000000 |
| C | 1.215083 | -3.095198 | -5.515164 |
| C | 2.265419 | -3.441102 | -4.984105 |
| C | 3.332280 | -3.641321 | -4.206887 |
| C | 4.202907 | -3.641235 | -3.336077 |
| C | 4.980097 | -3.442680 | -2.268907 |
| C | 5.510191 | -3.097653 | -1.217798 |
| C | 5.835903 | -2.487337 | -0.000000 |

**28**
State = $Q_1$
Symmetry = $C_1$
E = -2784.94069291 a.u.

| | | | |
|---|---|---|---|
| C | -8.152684 | -0.219132 | -2.127679 |
| C | -7.306188 | -2.404815 | -3.039857 |
| C | -8.103540 | 1.197185 | -2.297675 |
| C | -8.380803 | -0.100419 | 0.376779 |
| C | -7.705419 | -1.088787 | -3.165197 |
| H | -7.430177 | -2.909197 | -2.085752 |
| C | -7.434917 | 1.903541 | -3.277319 |
| H | -8.514563 | 1.791371 | -1.486301 |
| C | -8.427974 | -0.764332 | -0.815981 |
| H | -8.226548 | 0.974926 | 0.385044 |
| C | -6.553459 | -3.099620 | -4.010915 |
| H | -7.534968 | -0.632478 | -4.136323 |
| C | -7.108129 | 3.272175 | -3.168221 |
| H | -6.977105 | 1.365866 | -4.103264 |
| C | -8.289887 | -0.749838 | 1.645145 |
| H | -8.549408 | -1.842139 | -0.760070 |
| C | 1.219187 | -0.267672 | 7.603600 |
| C | -0.142414 | 0.163444 | 7.800348 |
| C | 2.231499 | 0.705266 | 7.341262 |
| C | 1.500889 | -1.658535 | 7.431585 |
| C | -1.286018 | -0.580969 | 7.691992 |
| H | -0.287075 | 1.231837 | 7.930885 |
| C | 2.069096 | 2.044515 | 7.053974 |
| H | 3.240419 | 0.322882 | 7.217316 |
| C | 2.620945 | -2.228545 | 6.864189 |
| H | 3.475427 | -1.602890 | 6.621636 |
| H | -1.221998 | -1.655397 | 7.545734 |
| C | 3.075472 | 2.835918 | 6.455173 |
| H | 1.084727 | 2.497575 | 7.131537 |
| C | 2.663863 | -3.551431 | 6.368741 |
| H | 0.683045 | -2.342170 | 7.639243 |
| C | 3.639230 | 6.868857 | -1.949817 |

| | | | |
|---|---|---|---|
| C | 3.409426 | 7.197210 | -0.565304 |
| C | 2.571138 | 7.023615 | -2.893732 |
| C | 4.822456 | 6.175283 | -2.319699 |
| C | 4.081623 | 6.708568 | 0.525690 |
| H | 2.518463 | 7.780319 | -0.351072 |
| C | 1.263118 | 7.372438 | -2.653272 |
| H | 2.794382 | 6.722857 | -3.913654 |
| C | 5.049466 | 5.399733 | -3.447069 |
| H | 5.603497 | 6.130986 | -1.565640 |
| C | 3.582684 | 6.759163 | 1.853487 |
| H | 4.960584 | 6.087840 | 0.375644 |
| C | 0.218424 | 7.164450 | -3.586676 |
| H | 0.960468 | 7.683890 | -1.657622 |
| C | 6.095600 | 4.469658 | -3.534976 |
| H | 4.302616 | 5.366910 | -4.235671 |
| C | 4.203661 | -6.399617 | -2.445898 |
| C | 2.922853 | -7.034914 | -2.611042 |
| C | 4.209431 | -6.653250 | 0.071661 |
| C | 4.763705 | -5.637492 | -3.505126 |
| C | 2.005567 | -6.832529 | -3.611045 |
| H | 2.587173 | -7.650926 | -1.781419 |
| C | 4.785485 | -6.334817 | -1.133998 |
| H | 3.231420 | -7.125287 | 0.099241 |
| C | 5.768036 | -4.682886 | -3.429750 |
| H | 4.263077 | -5.712134 | -4.466552 |
| H | 2.262428 | -6.198619 | -4.455296 |
| C | 4.724368 | -6.197468 | 1.311831 |
| H | 5.754773 | -5.849319 | -1.069516 |
| C | 0.645249 | -7.223254 | -3.514916 |
| H | 6.309554 | -4.553422 | -2.496892 |
| C | -6.160839 | 3.898985 | -3.952980 |
| C | -5.499689 | 5.096955 | -3.605097 |
| C | -2.148303 | 6.657916 | -4.061191 |
| C | -1.122189 | 7.243540 | -3.283639 |
| C | -7.908200 | -0.110376 | 2.791070 |
| C | -7.382414 | -0.762907 | 3.946305 |
| C | -4.844117 | -0.089355 | 6.564095 |
| C | -3.683820 | -0.700592 | 7.123165 |
| C | 4.031926 | -6.212380 | 2.500684 |
| C | 3.515002 | -5.135896 | 4.668565 |
| C | 3.647321 | -4.015635 | 5.520813 |
| C | 2.849413 | 4.058052 | 5.858371 |
| C | 3.722436 | 4.652061 | 4.917905 |
| C | 3.375133 | 5.679166 | 4.069228 |
| C | 4.076543 | 5.986435 | 2.878098 |
| C | -5.894143 | -4.285201 | -3.756845 |
| C | -4.831961 | -4.801700 | -4.531261 |
| C | -1.731925 | -6.799577 | -4.016490 |
| C | -0.352951 | -6.751928 | -4.334687 |
| C | 6.150953 | 3.432564 | -4.458540 |
| C | 6.674794 | -1.390430 | -4.943524 |
| C | 6.764803 | -2.570225 | -4.195215 |
| C | 6.002850 | -3.712470 | -4.413891 |
| C | -2.581558 | -0.006849 | 7.543254 |
| C | 6.918198 | 2.274471 | -4.282141 |

| | | | |
|---|---|---|---|
| C | -2.702654 | -6.054728 | -4.646710 |
| C | -3.978654 | -5.792370 | -4.095844 |
| C | 4.354218 | -5.389509 | 3.606288 |
| C | -3.441333 | 6.463905 | -3.627421 |
| C | -4.339132 | 5.537831 | -4.205229 |
| C | 7.157649 | -0.163030 | -4.502533 |
| C | 6.720893 | 1.082179 | -4.969981 |
| C | -5.790813 | -0.755272 | 5.836802 |
| C | -6.686349 | -0.116102 | 4.928171 |
| H | 5.424069 | -3.772692 | -5.334759 |
| H | 7.343631 | -2.526595 | -3.273277 |
| H | 6.044519 | -1.401257 | -5.831796 |
| H | 7.786310 | -0.164869 | -3.612747 |
| H | 6.041282 | 1.092141 | -5.821066 |
| H | 7.596366 | 2.253659 | -3.429696 |
| H | 5.427443 | 3.438013 | -5.272658 |
| H | 6.819691 | 4.455739 | -2.720966 |
| H | 0.356789 | -7.808960 | -2.642718 |
| H | -0.064698 | -6.159784 | -5.202025 |
| H | -2.013192 | -7.344246 | -3.115891 |
| H | -2.422570 | -5.500655 | -5.541451 |
| H | -4.231118 | -6.306946 | -3.169417 |
| H | -4.581747 | -4.276880 | -5.452121 |
| H | -6.097587 | -4.781600 | -2.808699 |
| H | -6.355844 | -2.592775 | -4.954429 |
| H | 5.667862 | -5.653401 | 1.282402 |
| H | 3.087865 | -6.753755 | 2.539715 |
| H | 5.262136 | -4.792805 | 3.525790 |
| H | 2.605002 | -5.727473 | 4.757492 |
| H | 4.523244 | -3.385588 | 5.370792 |
| H | 1.786406 | -4.176441 | 6.529031 |
| H | -2.653023 | 1.077817 | 7.613469 |
| H | -3.611466 | -1.784393 | 7.046170 |
| H | -4.881492 | 0.999109 | 6.579481 |
| H | -5.750184 | -1.843104 | 5.808786 |
| H | -6.672906 | 0.972874 | 4.912948 |
| H | -7.389970 | -1.851870 | 3.947825 |
| H | -7.831485 | 0.975783 | 2.767730 |
| H | -8.361955 | -1.836489 | 1.655708 |
| H | 0.497694 | 6.774654 | -4.565016 |
| H | -1.402951 | 7.637441 | -2.307903 |
| H | -1.853199 | 6.196633 | -5.003011 |
| H | -3.739018 | 6.924308 | -2.686414 |
| H | -4.007808 | 5.024997 | -5.107433 |
| H | -5.833919 | 5.606897 | -2.702646 |
| H | -5.776145 | 3.354588 | -4.814497 |
| H | -7.497765 | 3.813123 | -2.306864 |
| H | 4.052523 | 2.375230 | 6.315051 |
| H | 1.871541 | 4.519068 | 5.991069 |
| H | 4.674209 | 4.151663 | 4.744339 |
| H | 2.421269 | 6.179453 | 4.231227 |
| H | 4.996314 | 5.432507 | 2.694938 |
| H | 2.660884 | 7.314272 | 2.022967 |

**29**

State = $S_0$
Symmetry = $C_1$
E = -2629.95959356 a.u.

| | | | |
|---|---|---|---|
| C | -3.777294 | -5.749608 | -3.320917 |
| C | -4.557533 | -3.759348 | -4.716332 |
| C | -3.903103 | -6.306180 | -2.002258 |
| C | -1.439909 | -6.667262 | -3.727754 |
| C | -4.681449 | -4.710401 | -3.726954 |
| H | -3.717126 | -3.802057 | -5.405633 |
| C | -4.738213 | -5.939970 | -0.967993 |
| H | -3.128141 | -7.018097 | -1.726400 |
| C | -2.593344 | -5.999872 | -4.089378 |
| H | -1.405956 | -7.189827 | -2.774241 |
| C | -5.327390 | -2.563105 | -4.729346 |
| H | -5.529845 | -4.556871 | -3.063374 |
| C | -4.484058 | -6.248600 | 0.395393 |
| H | -5.526425 | -5.212058 | -1.147696 |
| C | -0.195506 | -6.522576 | -4.392399 |
| H | -2.540311 | -5.477112 | -5.042581 |
| C | 7.900736 | -0.330769 | 0.109017 |
| C | 7.772138 | -1.709989 | -0.255294 |
| C | 7.728546 | 0.063509 | 1.477309 |
| C | 7.928532 | 0.668473 | -0.926341 |
| C | 7.459267 | -2.263406 | -1.482022 |
| H | 7.786662 | -2.413773 | 0.575430 |
| C | 7.217788 | -0.672167 | 2.530642 |
| H | 7.872401 | 1.123247 | 1.680653 |
| C | 7.620581 | 2.007550 | -0.837277 |
| H | 7.407506 | 2.432340 | 0.141191 |
| H | 7.366687 | -1.611886 | -2.348052 |
| C | 6.724321 | -0.118213 | 3.735936 |
| H | 6.999475 | -1.726892 | 2.374576 |
| C | 7.314118 | 2.841549 | -1.947108 |
| H | 8.084260 | 0.291837 | -1.935752 |
| C | -2.087742 | -0.249741 | 7.065659 |
| C | -0.777193 | -0.779921 | 7.290513 |
| C | -3.159958 | -1.127370 | 6.679053 |
| C | -2.275774 | 1.167974 | 6.935892 |
| C | 0.445544 | -0.131175 | 7.291799 |
| H | -0.716755 | -1.866151 | 7.324881 |
| C | -3.113445 | -2.450082 | 6.297690 |
| H | -4.121557 | -0.643182 | 6.522828 |
| C | -3.323236 | 1.861681 | 6.366112 |
| H | -1.409806 | 1.776234 | 7.189518 |
| C | 1.690911 | -0.782135 | 7.135762 |
| H | 0.468652 | 0.955177 | 7.230432 |
| C | -4.143444 | -3.087529 | 5.553691 |
| H | -2.176077 | -2.995984 | 6.379436 |
| C | -3.234448 | 3.206511 | 5.920532 |
| H | -4.211422 | 1.319330 | 6.050177 |
| C | -3.242099 | 6.360859 | -2.825008 |
| C | -3.582311 | 5.500236 | -3.930256 |
| C | -0.867951 | 6.844697 | -3.618791 |
| C | -4.066417 | 6.337754 | -1.643064 |
| C | -4.540577 | 4.516891 | -3.988618 |
| H | -2.902249 | 5.539747 | -4.779146 |
| C | -1.967101 | 6.993104 | -2.784541 |
| H | -0.958834 | 6.246359 | -4.522766 |
| C | -3.720629 | 6.629652 | -0.343346 |
| H | -5.048385 | 5.885344 | -1.767451 |
| H | -5.223870 | 4.396504 | -3.151252 |
| C | 0.440380 | 7.214997 | -3.239479 |
| H | -1.776075 | 7.585313 | -1.891030 |
| C | -4.566045 | 3.465624 | -4.951557 |
| H | -2.728227 | 7.025206 | -0.137194 |
| C | -5.062874 | -5.617419 | 1.475814 |
| C | -4.834796 | -4.637337 | 3.746041 |
| C | -4.005974 | -4.255936 | 4.836030 |
| C | 1.031677 | -6.871763 | -3.865249 |
| C | 2.281183 | -6.351512 | -4.291929 |
| C | 5.507149 | -5.220743 | -2.784781 |
| C | 6.343653 | -4.075365 | -2.769749 |
| C | 1.619198 | 6.809180 | -3.844611 |
| C | 2.874728 | 6.796382 | -3.192639 |
| C | 5.963846 | 4.709595 | -2.896461 |
| C | 6.682563 | 4.063420 | -1.855516 |
| C | 5.911700 | -0.783922 | 4.638982 |
| C | 4.002068 | -0.805732 | 6.226249 |
| C | 2.886491 | -0.162571 | 6.807425 |
| C | -5.045910 | -1.415408 | -5.434599 |
| C | -5.561830 | -0.131679 | -5.094321 |
| C | -5.057500 | 1.081457 | -5.497279 |
| C | -5.304653 | 2.313849 | -4.824056 |
| C | -4.113183 | 3.812378 | 5.046500 |
| C | -4.465318 | 6.210063 | 0.793475 |
| C | 6.997482 | -3.592338 | -1.652992 |
| C | -3.812527 | 4.947560 | 4.247981 |
| C | 3.992698 | 6.080214 | -3.577088 |
| C | 5.041807 | 5.715775 | -2.694659 |
| C | 5.052201 | -0.173070 | 5.579874 |
| C | -4.501779 | -5.562042 | 2.780350 |
| C | -3.973152 | 6.144340 | 2.078755 |
| C | -4.499805 | 5.326658 | 3.114956 |
| C | 4.550025 | -5.499967 | -3.738459 |
| C | 3.441972 | -6.366678 | -3.546902 |
| H | -5.425722 | 5.729819 | 0.597616 |
| H | -2.995789 | 6.592294 | 2.263704 |
| H | -5.435008 | 4.807909 | 2.898237 |
| H | -2.856826 | 5.437691 | 4.440689 |
| H | -5.024692 | 3.268312 | 4.794989 |
| H | -2.306257 | 3.734949 | 6.145101 |
| H | -3.821217 | 3.508635 | -5.748047 |
| H | -6.022810 | 2.273542 | -4.003835 |
| H | -4.277979 | 1.083398 | -6.260410 |
| H | -6.312112 | -0.115605 | -4.301974 |
| H | -4.235870 | -1.447046 | -6.163979 |
| H | -6.116236 | -2.496923 | -3.977655 |
| H | 0.534516 | 7.727293 | -2.279742 |
| H | 1.545788 | 6.273392 | -4.791732 |
| H | 2.901917 | 7.251129 | -2.200441 |

| | | | |
|---|---|---|---|
| H | 3.971792 | 5.588994 | -4.550912 |
| H | 4.992146 | 6.143308 | -1.691618 |
| H | 6.002480 | 4.246278 | -3.883645 |
| H | 6.555231 | 4.480364 | -0.855319 |
| H | 7.424173 | 2.400240 | -2.939426 |
| H | 7.004689 | -4.228089 | -0.765542 |
| H | 6.300155 | -3.426605 | -3.645582 |
| H | 5.496535 | -5.825464 | -1.876183 |
| H | 4.526161 | -4.865841 | -4.626060 |
| H | 3.433898 | -6.946544 | -2.622498 |
| H | 2.270607 | -5.732906 | -5.191001 |
| H | 1.031844 | -7.425353 | -2.925168 |
| H | -0.197255 | -5.933139 | -5.311235 |
| H | -5.051966 | -2.505179 | 5.389119 |
| H | -3.084902 | -4.824535 | 4.970808 |
| H | -5.704597 | -4.006432 | 3.555531 |
| H | -3.609779 | -6.166790 | 2.951452 |
| H | -5.893505 | -4.939176 | 1.275194 |
| H | -3.631429 | -6.900258 | 0.594669 |
| H | 6.841170 | 0.959422 | 3.865302 |
| H | 5.782281 | -1.857325 | 4.490503 |
| H | 5.106477 | 0.914087 | 5.659544 |
| H | 3.937031 | -1.890265 | 6.121620 |
| H | 2.904186 | 0.928441 | 6.822658 |
| H | 1.669291 | -1.872955 | 7.098073 |

**30**
State = $Q_1$
Symmetry = $C_1$
E = -2741.34710761 a.u.

| | | | |
|---|---|---|---|
| C | 4.707262 | 4.423247 | -0.439947 |
| C | 5.094294 | 3.936882 | 0.807688 |
| C | 5.255342 | 3.299839 | 1.845648 |
| C | 5.253686 | 2.456218 | 2.877216 |
| C | 5.089759 | 1.565528 | 3.707229 |
| C | 5.095964 | 3.699139 | -1.565786 |
| C | 5.258416 | 2.868916 | -2.456551 |
| C | 5.258082 | 1.837469 | -3.300321 |
| C | 5.095200 | 0.799841 | -3.937441 |
| C | 3.716813 | 5.399720 | -0.538445 |
| C | 2.675341 | 6.048089 | -0.604074 |
| C | 1.438659 | 6.533377 | -0.653463 |
| C | 0.226474 | 6.765963 | -0.677482 |
| C | -1.100398 | 6.746975 | -0.676328 |
| C | -2.311137 | 6.520100 | -0.654351 |
| C | 4.706255 | -0.447166 | -4.423892 |
| C | 4.700016 | 0.439690 | 4.430782 |
| C | -3.573445 | 5.948086 | -0.597887 |
| C | 5.087818 | -0.807931 | 3.944996 |
| C | 5.249942 | -1.845851 | 3.308160 |
| C | 5.249758 | -2.877283 | 2.464371 |
| C | 5.087300 | -3.707169 | 1.573294 |
| C | 3.707846 | 0.538139 | 5.405512 |
| C | 2.665244 | 0.603732 | 6.052064 |
| C | 1.427729 | 0.653144 | 6.535223 |
| C | 0.215146 | 0.677296 | 6.765703 |
| C | -1.111692 | 0.676423 | 6.744393 |
| C | -2.322073 | 0.654542 | 6.515594 |
| C | 5.093171 | -1.573627 | -3.699794 |
| C | 5.254290 | -2.464616 | -2.869552 |
| C | 5.252788 | -3.308197 | -1.837951 |
| C | 5.088988 | -3.944887 | -0.800204 |
| C | 3.715394 | -0.544054 | -5.400108 |
| C | 2.673644 | -0.608031 | -6.048194 |
| C | 1.436755 | -0.655554 | -6.533142 |
| C | 0.224470 | -0.677735 | -6.765382 |
| C | -1.102394 | -0.674515 | -6.746024 |
| C | -2.313078 | -0.650732 | -6.519020 |
| C | -4.133446 | 5.650600 | 0.648722 |
| C | -4.443095 | 5.166345 | 1.731848 |
| C | -4.626087 | 4.476541 | 2.860700 |
| C | -4.627772 | 3.697829 | 3.812358 |
| C | -4.448326 | 2.727624 | 4.712429 |
| C | -4.141446 | 1.761568 | 5.402432 |
| C | -3.583765 | 0.598061 | 5.942212 |
| C | 4.699145 | -4.430583 | 0.446818 |
| C | -4.131804 | 5.409073 | -1.761431 |
| C | -4.440040 | 4.719538 | -2.727392 |
| C | -4.621572 | 3.819601 | -3.697332 |
| C | -4.622046 | 2.867708 | -4.475757 |
| C | -4.441472 | 1.738402 | -5.165460 |
| C | -4.133768 | 0.654813 | -5.649911 |
| C | -3.575581 | -0.592556 | -5.947598 |
| C | -4.143332 | -0.648550 | 5.643939 |
| C | -4.452169 | -1.731747 | 5.159325 |
| C | -4.633653 | -2.860836 | 4.469512 |
| C | -4.633744 | -3.812621 | 3.690956 |
| C | -4.452538 | -4.712653 | 2.721041 |
| C | -4.143978 | -5.402558 | 1.755451 |
| C | 3.707054 | -5.405544 | 0.543750 |
| C | 2.664459 | -6.052263 | 0.607781 |
| C | 1.426881 | -6.535438 | 0.655373 |
| C | 0.214245 | -6.765834 | 0.677673 |
| C | -1.112588 | -6.744432 | 0.674678 |
| C | -2.322921 | -6.515562 | 0.650968 |
| C | -4.135659 | -1.755325 | -5.408705 |
| C | -4.445253 | -2.720939 | -4.719295 |
| C | -4.627869 | -3.690835 | -3.819532 |
| C | -4.629197 | -4.469346 | -2.867711 |
| C | -4.449484 | -5.159173 | -1.738344 |
| C | -4.142355 | -5.643895 | -0.654713 |
| C | -3.584457 | -5.942021 | 0.592681 |

**31**
State = $Q_1$
Symmetry = $C_S$
E = -2436.59189408 a.u.

| | | | |
|---|---|---|---|
| C | 3.591519 | 4.408492 | -0.000000 |
| C | 4.021073 | 3.835825 | 1.195768 |
| C | 4.201129 | 3.112300 | 2.172623 |

| | | | |
|---|---|---|---|
| C | 4.200508 | 2.169844 | 3.114591 |
| C | 4.019277 | 1.193010 | 3.837837 |
| C | 4.021073 | 3.835825 | -1.195768 |
| C | 4.201129 | 3.112300 | -2.172623 |
| C | 4.200508 | 2.169844 | -3.114591 |
| C | 4.019277 | 1.193010 | -3.837837 |
| C | 2.504622 | 5.284410 | -0.000000 |
| C | 1.393516 | 5.805929 | -0.000000 |
| C | 0.094525 | 6.106620 | -0.000000 |
| C | -1.134426 | 6.147282 | -0.000000 |
| C | 3.588845 | -0.002458 | -4.410492 |
| C | 3.588845 | -0.002458 | 4.410492 |
| C | -2.487833 | 5.829994 | -0.000000 |
| C | 4.018279 | -1.198599 | 3.838526 |
| C | 4.199303 | -2.175426 | 3.115216 |
| C | 4.199134 | -3.117510 | 2.172871 |
| C | 4.018182 | -3.840539 | 1.195817 |
| C | 2.500983 | -0.001782 | 5.285212 |
| C | 1.389362 | -0.001190 | 5.805704 |
| C | 0.090127 | -0.000184 | 6.105344 |
| C | -1.138826 | 0.000527 | 6.145777 |
| C | 4.018279 | -1.198599 | -3.838526 |
| C | 4.199303 | -2.175426 | -3.115216 |
| C | 4.199134 | -3.117510 | -2.172871 |
| C | 4.018182 | -3.840539 | -1.195817 |
| C | 2.500983 | -0.001782 | -5.285212 |
| C | 1.389362 | -0.001190 | -5.805704 |
| C | 0.090127 | -0.000184 | -6.105344 |
| C | -1.138826 | 0.000527 | -6.145777 |
| C | -3.100415 | 5.518247 | 1.217526 |
| C | -3.439057 | 4.983336 | 2.268139 |
| C | -3.642438 | 4.211652 | 3.339651 |
| C | -3.641969 | 3.342778 | 4.209967 |
| C | -3.440718 | 2.270572 | 4.981243 |
| C | -3.103576 | 1.219668 | 5.516524 |
| C | -2.492153 | 0.001520 | 5.828059 |
| C | 3.587965 | -4.412627 | 0.000000 |
| C | -3.100415 | 5.518247 | -1.217526 |
| C | -3.439057 | 4.983336 | -2.268139 |
| C | -3.642438 | 4.211652 | -3.339651 |
| C | -3.641969 | 3.342778 | -4.209967 |
| C | -3.440718 | 2.270572 | -4.981243 |
| C | -3.103576 | 1.219668 | -5.516524 |
| C | -2.492153 | 0.001520 | -5.828059 |
| C | -3.105226 | -1.215623 | 5.515855 |
| C | -3.444010 | -2.265943 | 4.980442 |
| C | -3.647040 | -3.337381 | 4.208588 |
| C | -3.647097 | -4.207334 | 3.339338 |
| C | -3.444277 | -4.979172 | 2.267830 |
| C | -3.105783 | -5.514884 | 1.217572 |
| C | 2.499948 | -5.287156 | 0.000000 |
| C | 1.388065 | -5.807072 | 0.000000 |
| C | 0.088662 | -6.106086 | 0.000000 |
| C | -1.140328 | -6.145352 | 0.000000 |
| C | -3.105226 | -1.215623 | -5.515855 |
| C | -3.444010 | -2.265943 | -4.980442 |
| C | -3.647040 | -3.337381 | -4.208588 |
| C | -3.647097 | -4.207334 | -3.339338 |
| C | -3.444277 | -4.979172 | -2.267830 |
| C | -3.105783 | -5.514884 | -1.217572 |
| C | -2.493474 | -5.826950 | 0.000000 |

$N_4(C_8)_6$
State = S0
Symmetry = $T_D$
E = -2046.96944081 a.u.

| | | | |
|---|---|---|---|
| C | -2.902128 | -2.902128 | 4.546826 |
| C | -2.198101 | -2.198101 | 5.239032 |
| C | -1.322611 | -1.322611 | 5.775458 |
| C | 1.322611 | 1.322611 | 5.775458 |
| C | 2.198101 | 2.198101 | 5.239032 |
| C | 2.902128 | 2.902128 | 4.546826 |
| C | -4.546826 | 2.902128 | -2.902128 |
| C | -5.239032 | 2.198101 | -2.198101 |
| C | -5.775458 | 1.322611 | -1.322611 |
| C | -5.775458 | -1.322611 | 1.322611 |
| C | -5.239032 | -2.198101 | 2.198101 |
| C | -4.546826 | -2.902128 | 2.902128 |
| C | 5.775458 | 1.322611 | 1.322611 |
| C | 5.239032 | 2.198101 | 2.198101 |
| C | 4.546826 | 2.902128 | 2.902128 |
| C | -2.902128 | 4.546826 | -2.902128 |
| C | -2.198101 | 5.239032 | -2.198101 |
| C | -1.322611 | 5.775458 | -1.322611 |
| C | 1.322611 | 5.775458 | 1.322611 |
| C | 2.902128 | 4.546826 | 2.902128 |
| C | 2.198101 | 5.239032 | 2.198101 |
| C | 2.902128 | -2.902128 | -4.546826 |
| C | 2.198101 | -2.198101 | -5.239032 |
| C | 1.322611 | -1.322611 | -5.775458 |
| C | -1.322611 | 1.322611 | -5.775458 |
| C | -2.198101 | 2.198101 | -5.239032 |
| C | -2.902128 | 2.902128 | -4.546826 |
| C | -2.902128 | -4.546826 | 2.902128 |
| C | -2.198101 | -5.239032 | 2.198101 |
| C | -1.322611 | -5.775458 | 1.322611 |
| C | 1.322611 | -5.775458 | -1.322611 |
| C | 2.198101 | -5.239032 | -2.198101 |
| C | 2.902128 | -4.546826 | -2.902128 |
| N | 3.581399 | 3.581399 | 3.581399 |
| N | -3.581399 | -3.581399 | 3.581399 |
| N | 3.581399 | -3.581399 | -3.581399 |
| N | -3.581399 | 3.581399 | -3.581399 |
| C | -0.475226 | -0.475226 | 6.024396 |
| C | 0.475226 | 0.475226 | 6.024396 |
| C | 0.475226 | 6.024396 | 0.475226 |
| C | -0.475226 | 6.024396 | -0.475226 |
| C | 6.024396 | 0.475226 | 0.475226 |
| C | 6.024396 | -0.475226 | -0.475226 |
| C | -0.475226 | -6.024396 | 0.475226 |

| | | | |
|---|---|---|---|
| C | 0.475226 | -6.024396 | -0.475226 |
| C | -6.024396 | -0.475226 | 0.475226 |
| C | -6.024396 | 0.475226 | -0.475226 |
| C | 0.475226 | -0.475226 | -6.024396 |
| C | -0.475226 | 0.475226 | -6.024396 |
| C | 5.775458 | -1.322611 | -1.322611 |
| C | 5.239032 | -2.198101 | -2.198101 |
| C | 4.546826 | -2.902128 | -2.902128 |

$N_4(C_{10}H_{10})_6$
State = S0
Symmetry = T
E = -2541.69947360 a.u.

| | | | |
|---|---|---|---|
| C | 2.717737 | -5.730164 | -3.060530 |
| C | 5.250824 | -3.319696 | -3.957822 |
| C | 3.060530 | -2.717737 | -5.730164 |
| C | 3.957822 | -5.250824 | -3.319696 |
| H | 1.882430 | -5.400999 | -3.669196 |
| C | 5.730164 | -3.060530 | -2.717737 |
| H | 5.547281 | -2.706589 | -4.799698 |
| C | 3.319696 | -3.957822 | -5.250824 |
| H | 3.669196 | -1.882430 | -5.400999 |
| C | 2.378443 | -6.476690 | -1.882401 |
| H | 4.799698 | -5.547281 | -2.706589 |
| C | 6.476690 | -1.882401 | -2.378443 |
| H | 5.400999 | -3.669196 | -1.882430 |
| C | 1.882401 | -2.378443 | -6.476690 |
| H | 2.706589 | -4.799698 | -5.547281 |
| C | -3.319696 | 3.957822 | -5.250824 |
| C | -3.957822 | 5.250824 | -3.319696 |
| C | -5.250824 | 3.319696 | -3.957822 |
| C | -3.060530 | 2.717737 | -5.730164 |
| H | -2.706589 | 4.799698 | -5.547281 |
| C | -2.717737 | 5.730164 | -3.060530 |
| H | -4.799698 | 5.547281 | -2.706589 |
| C | -5.730164 | 3.060530 | -2.717737 |
| H | -5.400999 | 3.669196 | -1.882430 |
| H | -3.669196 | 1.882430 | -5.400999 |
| C | -2.378443 | 6.476690 | -1.882401 |
| H | -1.882430 | 5.400999 | -3.669196 |
| C | -6.476690 | 1.882401 | -2.378443 |
| H | -5.547281 | 2.706589 | -4.799698 |
| C | 3.957822 | 5.250824 | 3.319696 |
| C | 5.250824 | 3.319696 | 3.957822 |
| C | 3.319696 | 3.957822 | 5.250824 |
| C | 2.717737 | 5.730164 | 3.060530 |
| H | 4.799698 | 5.547281 | 2.706589 |
| C | 5.730164 | 3.060530 | 2.717737 |
| H | 5.547281 | 2.706589 | 4.799698 |
| C | 3.060530 | 2.717737 | 5.730164 |
| H | 2.706589 | 4.799698 | 5.547281 |
| C | 2.378443 | 6.476690 | 1.882401 |
| H | 1.882430 | 5.400999 | 3.669196 |
| C | 6.476690 | 1.882401 | 2.378443 |
| H | 5.400999 | 3.669196 | 1.882430 |
| C | 1.882401 | 2.378443 | 6.476690 |
| H | 3.669196 | 1.882430 | 5.400999 |
| C | -3.957822 | -5.250824 | 3.319696 |
| C | -5.730164 | -3.060530 | 2.717737 |
| C | -3.319696 | -3.957822 | 5.250824 |
| C | -2.717737 | -5.730164 | 3.060530 |
| H | -4.799698 | -5.547281 | 2.706589 |
| C | -5.250824 | -3.319696 | 3.957822 |
| H | -5.400999 | -3.669196 | 1.882430 |
| C | -3.060530 | -2.717737 | 5.730164 |
| H | -2.706589 | -4.799698 | 5.547281 |
| H | -1.882430 | -5.400999 | 3.669196 |
| C | -6.476690 | -1.882401 | 2.378443 |
| H | -5.547281 | -2.706589 | 4.799698 |
| C | -2.378443 | -6.476690 | 1.882401 |
| H | -3.669196 | -1.882430 | 5.400999 |
| C | 6.753626 | -1.528164 | -1.092666 |
| C | 7.013770 | -0.191055 | -0.654507 |
| C | 6.753626 | 1.528164 | 1.092666 |
| C | 1.528164 | -1.092666 | -6.753626 |
| C | 0.191055 | -0.654507 | -7.013770 |
| C | -0.191055 | 0.654507 | -7.013770 |
| C | -1.528164 | 1.092666 | -6.753626 |
| C | -6.753626 | -1.528164 | 1.092666 |
| C | -7.013770 | 0.191055 | -0.654507 |
| C | -6.753626 | 1.528164 | -1.092666 |
| C | 0.654507 | 7.013770 | 0.191055 |
| C | 1.092666 | 6.753626 | 1.528164 |
| C | 1.092666 | -6.753626 | -1.528164 |
| C | 0.654507 | -7.013770 | -0.191055 |
| C | 1.528164 | 1.092666 | 6.753626 |
| C | -0.191055 | -0.654507 | 7.013770 |
| C | -1.528164 | -1.092666 | 6.753626 |
| C | -1.882401 | -2.378443 | 6.476690 |
| C | -1.882401 | 2.378443 | -6.476690 |
| C | 0.191055 | 0.654507 | 7.013770 |
| C | -1.092666 | -6.753626 | 1.528164 |
| C | -0.654507 | -7.013770 | 0.191055 |
| C | -7.013770 | -0.191055 | 0.654507 |
| C | -0.654507 | 7.013770 | -0.191055 |
| C | -1.092666 | 6.753626 | -1.528164 |
| C | 7.013770 | 0.191055 | 0.654507 |
| H | -1.163106 | -3.173238 | 6.666867 |
| H | -2.268789 | -0.311843 | 6.587408 |
| H | 0.583930 | -1.419258 | 7.025825 |
| H | -0.583930 | 1.419258 | 7.025825 |
| H | 2.268789 | 0.311843 | 6.587408 |
| H | 1.163106 | 3.173238 | 6.666867 |
| H | -3.173238 | -6.666867 | 1.163106 |
| H | -0.311843 | -6.587408 | 2.268789 |
| H | -1.419258 | -7.025825 | -0.583930 |
| H | 1.419258 | -7.025825 | 0.583930 |
| H | 0.311843 | -6.587408 | -2.268789 |
| H | 3.173238 | -6.666867 | -1.163106 |
| H | -6.666867 | -1.163106 | 3.173238 |

| | | | |
|---|---|---|---|
| H | -6.587408 | -2.268789 | 0.311843 |
| H | -7.025825 | 0.583930 | 1.419258 |
| H | -7.025825 | -0.583930 | -1.419258 |
| H | -6.587408 | 2.268789 | -0.311843 |
| H | -6.666867 | 1.163106 | -3.173238 |
| H | -1.163106 | 3.173238 | -6.666867 |
| H | -2.268789 | 0.311843 | -6.587408 |
| H | 0.583930 | 1.419258 | -7.025825 |
| H | -0.583930 | -1.419258 | -7.025825 |
| H | 2.268789 | -0.311843 | -6.587408 |
| H | 1.163106 | -3.173238 | -6.666867 |
| H | 6.666867 | 1.163106 | 3.173238 |
| H | 6.587408 | 2.268789 | 0.311843 |
| H | 7.025825 | -0.583930 | 1.419258 |
| H | 7.025825 | 0.583930 | -1.419258 |
| H | 6.587408 | -2.268789 | -0.311843 |
| H | 6.666867 | -1.163106 | -3.173238 |
| H | -3.173238 | 6.666867 | -1.163106 |
| H | -0.311843 | 6.587408 | -2.268789 |
| H | -1.419258 | 7.025825 | 0.583930 |
| H | 1.419258 | 7.025825 | -0.583930 |
| H | 0.311843 | 6.587408 | 2.268789 |
| H | 3.173238 | 6.666867 | 1.163106 |
| N | -4.279892 | -4.279892 | 4.279892 |
| N | 4.279892 | 4.279892 | 4.279892 |
| N | 4.279892 | -4.279892 | -4.279892 |
| N | -4.279892 | 4.279892 | -4.279892 |

$N_4(C_8H_8)_6$ (2C removed from 2 linkers)
State = S0
Symmetry = C1
E = -2851.42470302 a.u.

| | | | |
|---|---|---|---|
| C | -8.282249 | -0.285590 | 0.599256 |
| C | -7.972263 | -1.165026 | -2.884436 |
| C | -7.546149 | 1.698620 | -2.844632 |
| C | -8.578657 | -0.911170 | -0.565111 |
| H | -7.955224 | 0.748709 | 0.575225 |
| C | -7.343570 | -2.354172 | -2.721957 |
| H | -8.083524 | -0.724122 | -3.867453 |
| C | -8.333134 | 1.037072 | -1.961517 |
| H | -6.972070 | 1.135078 | -3.572705 |
| C | -8.161283 | -0.954364 | 1.862167 |
| H | -8.870134 | -1.954219 | -0.573769 |
| C | -6.650746 | -3.059005 | -3.760817 |
| H | -7.216537 | -2.748377 | -1.719140 |
| C | -7.253057 | 3.098801 | -2.751036 |
| H | -8.887278 | 1.580464 | -1.206020 |
| C | 2.352462 | 7.398464 | -2.770962 |
| C | 4.569101 | 6.556454 | -2.319782 |
| C | 3.236236 | 7.447890 | -0.523726 |
| C | 1.047165 | 7.441856 | -2.409259 |
| H | 2.633437 | 7.394440 | -3.816777 |
| C | 4.616556 | 5.677471 | -3.350765 |
| H | 5.430972 | 6.694446 | -1.678523 |
| C | 3.842134 | 6.733515 | 0.454906 |
| H | 4.583614 | 5.989809 | 0.182483 |
| H | 0.785848 | 7.420491 | -1.356683 |
| C | 5.706608 | 4.772328 | -3.562711 |
| H | 3.732012 | 5.519199 | -3.959029 |
| C | 3.435590 | 6.735891 | 1.830663 |
| H | 2.469798 | 8.174562 | -0.285264 |
| C | 4.782328 | -5.579577 | -3.874983 |
| C | 3.071347 | -7.028506 | -2.977956 |
| C | 4.946450 | -6.380942 | -1.607567 |
| C | 5.551186 | -4.480320 | -3.681239 |
| H | 4.389751 | -5.815010 | -4.856257 |
| C | 2.077839 | -6.604095 | -3.795792 |
| H | 2.899915 | -7.834689 | -2.275175 |
| C | 4.319989 | -6.533085 | -0.415636 |
| H | 5.990569 | -6.096703 | -1.642609 |
| C | 5.781393 | -3.462235 | -4.663393 |
| H | 5.905397 | -4.254427 | -2.681162 |
| C | 0.720808 | -7.057482 | -3.706529 |
| H | 2.260457 | -5.769084 | -4.464149 |
| C | 4.907987 | -6.156613 | 0.837402 |
| H | 3.266616 | -6.792069 | -0.394253 |
| C | 0.293624 | -0.134739 | 7.900295 |
| C | 2.262520 | 1.738876 | 6.916114 |
| C | 1.922433 | -1.835783 | 7.368689 |
| C | -0.797682 | -0.863561 | 7.563958 |
| H | 0.182336 | 0.859521 | 8.314676 |
| C | 2.557694 | 0.491815 | 7.356140 |
| H | 1.224869 | 2.047253 | 6.843281 |
| C | 2.915545 | -2.260113 | 6.550209 |
| H | 1.221241 | -2.544868 | 7.789838 |
| H | -0.668176 | -1.845062 | 7.120488 |
| C | 3.229122 | 2.626298 | 6.337080 |
| H | 3.586506 | 0.157820 | 7.404495 |
| C | -2.135421 | -0.346824 | 7.568188 |
| H | 3.600414 | -1.536529 | 6.121418 |
| C | -5.875553 | -4.151399 | -3.510717 |
| C | -4.842470 | -4.650313 | -4.362639 |
| C | -1.684946 | -6.586715 | -4.040481 |
| C | -0.304251 | -6.477384 | -4.391731 |
| C | -6.301258 | 3.715111 | -3.507012 |
| C | -5.656706 | 4.942167 | -3.159522 |
| C | -2.404964 | 6.658119 | -3.709453 |
| C | -1.342672 | 7.204429 | -2.925102 |
| C | 3.306897 | 5.498407 | 3.975933 |
| C | 3.941754 | 5.865239 | 2.748077 |
| C | 5.655481 | 3.727511 | -4.437187 |
| C | 6.477274 | 2.563380 | -4.345994 |
| C | 6.290531 | -1.069057 | -5.094389 |
| C | 6.406630 | -2.288984 | -4.361132 |
| C | -7.643872 | -0.349840 | 2.968094 |
| C | -7.064833 | -1.034012 | 4.081449 |
| C | -4.416963 | -0.423766 | 6.612015 |
| C | -3.194047 | -1.024882 | 7.043264 |
| C | 4.215011 | -6.112597 | 2.009640 |
| C | 3.782325 | -5.090068 | 4.216348 |

| | | | |
|---|---|---|---|
| C | 3.917674 | -3.963273 | 5.086772 |
| C | 3.012137 | -3.595965 | 6.036073 |
| C | -0.046398 | 7.321207 | -3.328798 |
| C | 4.585549 | -5.314546 | 3.137170 |
| C | -5.354264 | -1.072031 | 5.861609 |
| C | -6.317062 | -0.412239 | 5.038648 |
| C | 2.898067 | 3.793052 | 5.716545 |
| C | 3.723946 | 4.464637 | 4.761611 |
| C | 6.727543 | 0.130694 | -4.610139 |
| C | 6.245065 | 1.405514 | -5.031799 |
| C | -2.672385 | -5.817379 | -4.584106 |
| C | -3.942616 | -5.598284 | -3.970094 |
| C | -3.635215 | 6.354262 | -3.202568 |
| C | -4.545524 | 5.423645 | -3.789083 |
| H | 2.223540 | -4.295876 | 6.307211 |
| H | 4.723132 | -3.268123 | 4.854390 |
| H | 2.888659 | -5.700868 | 4.333931 |
| H | 5.494293 | -4.722454 | 3.040256 |
| H | 3.229683 | -6.574922 | 2.042356 |
| H | 5.908089 | -5.727153 | 0.807861 |
| H | -2.272046 | 0.691869 | 7.864252 |
| H | -3.049444 | -2.071157 | 6.778151 |
| H | -4.517096 | 0.651330 | 6.752065 |
| H | -5.261578 | -2.150191 | 5.740741 |
| H | -6.319961 | 0.676327 | 5.065801 |
| H | -7.082385 | -2.122387 | 4.060033 |
| H | -7.500753 | 0.729302 | 2.938077 |
| H | -8.336548 | -2.028576 | 1.882520 |
| H | 4.253364 | 2.265690 | 6.257674 |
| H | 1.879550 | 4.162369 | 5.825861 |
| H | 4.690506 | 4.012010 | 4.545463 |
| H | 2.352746 | 5.971545 | 4.202114 |
| H | 4.815934 | 5.280579 | 2.465142 |
| H | 2.581761 | 7.353236 | 2.104275 |
| H | 0.193204 | 7.169320 | -4.379837 |
| H | -1.567285 | 7.394303 | -1.876491 |
| H | -2.160002 | 6.326058 | -4.716981 |
| H | -3.880817 | 6.713818 | -2.204424 |
| H | -4.237576 | 4.955546 | -4.722714 |
| H | -5.979782 | 5.425876 | -2.239036 |
| H | -5.879896 | 3.165976 | -4.347657 |
| H | -7.703035 | 3.649681 | -1.926729 |
| H | 0.488141 | -7.799277 | -2.944365 |
| H | -0.056512 | -5.756141 | -5.169086 |
| H | -1.929016 | -7.211620 | -3.182841 |
| H | -2.432692 | -5.207492 | -5.453683 |
| H | -4.127435 | -6.121174 | -3.032873 |
| H | -4.680259 | -4.134668 | -5.307634 |
| H | -5.935314 | -4.597270 | -2.518831 |
| H | -6.631044 | -2.611391 | -4.752852 |
| H | 6.543785 | 4.829546 | -2.868910 |
| H | 4.828836 | 3.688678 | -5.144826 |
| H | 7.223606 | 2.550185 | -3.553152 |
| H | 5.513732 | 1.417476 | -5.838253 |
| H | 7.357535 | 0.120988 | -3.721841 |
| H | 5.678204 | -1.080051 | -5.994391 |
| H | 6.905727 | -2.218955 | -3.395565 |
| H | 5.301957 | -3.573422 | -5.634253 |
| N | 1.628491 | -0.503499 | 7.687112 |
| N | 4.344084 | -6.453384 | -2.870392 |
| N | -8.448615 | -0.353390 | -1.845229 |
| N | 3.442176 | 7.273722 | -1.900528 |
